# The genome of an underwater architect, the caddisfly *Stenopsyche tienmushanensis* Hwang (Insecta: Trichoptera)

**DOI:** 10.1093/gigascience/giy143

**Published:** 2018-11-23

**Authors:** Shiqi Luo, Min Tang, Paul B Frandsen, Russell J Stewart, Xin Zhou

**Affiliations:** 1 Beijing Advanced Innovation Center for Food Nutrition and Human Health, College of Plant Protection, China Agricultural University, 2 Yuanmingyuan West Road, Haidian District, Beijing 100193, China; 2 Department of Plant and Wildlife Sciences, Brigham Young University, 701 E University Parkway Drive, Provo, UT 84602, USA; 3 Data Science Lab, Smithsonian Institution, 600 Maryland Ave SW, Washington, DC 20002, USA; 4 Department of Biomedical Engineering, University of Utah, 20 South 2030 East, Salt Lake City, UT 84112, USA

**Keywords:** caddisworm, caddisfly, aquatic insect, freshwater adaptation, silk, H-fibroin, PacBio

## Abstract

**Background:**

Caddisflies (Insecta: Trichoptera) are a highly adapted freshwater group of insects split from a common ancestor with Lepidoptera. They are the most diverse (>16,000 species) of the strictly aquatic insect orders and are widely employed as bio-indicators in water quality assessment and monitoring. Among the numerous adaptations to aquatic habitats, caddisfly larvae use silk and materials from the environment (e.g., stones, sticks, leaf matter) to build composite structures such as fixed retreats and portable cases. Understanding how caddisflies have adapted to aquatic habitats will help explain the evolution and subsequent diversification of the group.

**Findings:**

We sequenced a retreat-builder caddisfly *Stenopsyche tienmushanensis* Hwang and assembled a high-quality genome from both Illumina and Pacific Biosciences (PacBio) sequencing. In total, 601.2 M Illumina reads (90.2 Gb) and 16.9 M PacBio subreads (89.0 Gb) were generated. The 451.5 Mb assembled genome has a contig N50 of 1.29 M, has a longest contig of 4.76 Mb, and covers 97.65% of the 1,658 insect single-copy genes as assessed by Benchmarking Universal Single-Copy Orthologs. The genome comprises 36.76% repetitive elements. A total of 14,672 predicted protein-coding genes were identified. The genome revealed gene expansions in specific groups of the cytochrome P450 family and olfactory binding proteins, suggesting potential genomic features associated with pollutant tolerance and mate finding. In addition, the complete gene complex of the highly repetitive H-fibroin, the major protein component of caddisfly larval silk, was assembled.

**Conclusions:**

We report the draft genome of *Stenopsyche tienmushanensis*, the highest-quality caddisfly genome so far. The genome information will be an important resource for the study of caddisflies and may shed light on the evolution of aquatic insects.

## Data Description

Comprising >16,000 species and distributed worldwide except for Antarctica, caddisflies (Insecta: Trichoptera) are the most diverse of the strictly aquatic insect orders [1]. This highly adapted freshwater group split from a common ancestor with lepidopterans (moths and butterflies) more than 200 million years ago [2]. The transition between terrestrial and aquatic (freshwater) habitat has occurred multiple times independently within insects, with caddisflies representing one of the most recent examples [2]. Presumably, this radical transition required numerous adaptations in morphological, physiological, and molecular traits. Understanding these adaptations will help explain how insects, in general, have evolved as one of the most successful and abundant class of animals on the planet and how caddisflies, in particular, have adapted to a wide range of freshwater and marine habitats. Identifying the genomic underpinnings of the adaptive mechanisms of caddisflies will improve our knowledge of these thriving aquatic insects that, as major contributors to freshwater biodiversity, have been widely employed as bio-indicators in water quality assessment and monitoring [3].

In addition, caddisflies are of technological interest because, like their terrestrial moth and butterfly relatives, their larvae (caddisworms) spin silk. Unlike terrestrial silks, caddisworm silk is adapted to be spun into tough viscoelastic fibers while fully submerged in water. Caddisworms use their silk as an adhesive tape to construct a wide variety of composite structures using stones, sticks, leaf matter, and other sediment gathered from the benthos of freshwater rivers, lakes, streams, and marine tidal pools [4]. The larval architectures are suborder dependent and include transportable tube cases that provide camouflage and physical protection (suborder Integripalpia), stationary fixed retreats with silk nets for capturing food (suborder Annulipalpia), and rigid silk cases for pupation (suborder ‘Spicipalpia’) [5]. The distinct and varied deployments of their underwater silk are responsible, in large part, for the penetration of caddisworms into diverse aquatic habitats.

The major protein component of caddisworm silk is H-fibroin, a high-molecular-weight protein with a blocky, highly repetitive primary sequence. Caddisworm H-fibroins are extensively phosphorylated on repeating serine-rich motifs with the sequence (_p_SX)_n_, where _p_S is phosphoserine, X is a hydrophobic amino acid, and n = 2–6 [6, 7]. The (_p_SX)_n_ motifs form divalent metal ion-stabilized β domains that are responsible for the strength, toughness, and energy-dissipating self-recovery of caddisworm silk [8–10]. Currently, only incomplete caddisfly H-fibroin sequences are available through a GenBank search because it has not been possible to obtain full-length sequences from cDNAs [11, 12] or to assemble the highly repetitive sequence *de novo* from short-read RNA-sequencing (RNA-seq) data [13] in the absence of long-read sequences.

As both an underwater adhesive and a tough fully hydrated metallofiber, caddisworm silk may provide new insights into the mimetic design of tough adhesive materials for use in aquatic environments. The high-quality draft genome of a caddisfly, which includes the full assembly of the H-fibroin gene complex, will be invaluable for further identifying and characterizing the enzymes [14] and structural protein components of caddisworm silks.

### Sampling, taxonomy, and sample preparation

**Figure 1:**
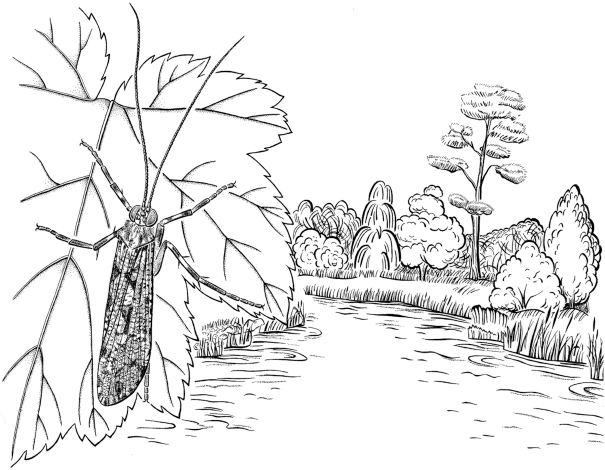
An illustration of the adult caddisfly *Stenopsyche tienmushanensis* in its typical habitat.

The caddisfly *Stenopsyche tienmushanensis* Hwang 1957 (Fig. 1, Supplementary Fig. S1, National Center for Biotechnology Information: txid1560151) is only found in China, representing one of the first caddisfly species described by Chinese taxonomists [15]. The distribution range of the species was recently reviewed and is confined to the central China region [16]. The larvae inhabit lotic environments (living in flowing waters) and are adapted to a wide range of micro-habitats, from pristine creeks to disturbed streams, displaying tolerance to various levels of pollutants.

Adult caddisfly specimens were collected using a light trap by the Yongding River, at Yanchi Town in Beijing, China (altitude 292 m, 40.03°N, 115.48°E) in 2017. This collection site is the most northern record for the species. All specimens used in this study were collected at the same site on the same night. Specimens were kept alive on ice, flash frozen, and transferred into a −80°C freezer until extraction. Two female *Stenopsyche* adults (Stie1, Stie2) were used for genome sequencing because the quantity of DNA from a single specimen was not sufficient for Pacific Biosciences (PacBio) sequencing. A third female individual (Stie3) was extracted for RNA and transcriptome sequencing. The guts were dissected, and the remaining whole bodies were used for DNA and RNA extractions. DNA was extracted with sodium dodecyl sulfate (SDS) and proteinase K using the protocol developed by Hu et al. [17, 18]. Total RNA was extracted with TRIzol following the manufacturer's instructions (Thermo Fisher). Taxonomic identification was conducted by X.Z. using male morphology and confirmed by Cytochrome *c* Oxidase Subunit I barcodes.

### Genome and transcriptome sequencing

Separate 400 bp insert libraries were created from Stie1 and Stie2 DNA. We generated ca. 270 million 150 bp paired-end (PE) reads, 80.45 Gb in total, using the Illumina HiSeq X Ten sequencing platform at WuXi AppTec (Shanghai, China) (Supplementary Table S1). We then combined and sequenced the remaining DNA from Stie1 and Stie2 using 12 PacBio Sequel single-molecule real-time (SMRT) cells 1M v2 (PacBio p/n101–008-000), with one movie of 600 minutes at the Genome Center of Nextomics (Wuhan, China). We produced 78.72 Gb of subreads, resulting in a mean subread length of 7.6 kb (Supplementary Table S1).

We sequenced RNA samples using the Illumina HiSeq X Ten platform (insert size of 180 bp, 150 PE reads) and the PacBio Sequel system (Iso-Seq, library size 0.5–6k), which produced 9.72 Gb and 10.31 Gb data, respectively (Supplementary Table S1). We used the PacBio RNA sequences to obtain full-length transcripts and the Illumina RNA sequences to polish/correct sequencing errors for the PacBio reads (see Transcriptome analysis section).

For DNA reads sequenced with Illumina, we trimmed three and two bases at the 5′ and 3′ ends, respectively, using fastp (v 0.18.0) [19]. These termini showed higher fluctuation in per base quality scores, which were reported in the fastq files. For RNA reads from Illumina, we filtered the raw data using fastp with default parameters. For the PacBio data, the subreads (basecalls from a single pass of the insert DNA template) of poor quality were filtered out based on the signal-to-noise ratio with default parameters. This analytical step is part of the integrative data processing procedure and is performed automatically when the raw data are produced during sequencing.

### Genome assembly and polishing

Before genome assembly, we estimated the genome size by *k*-mer analyses of the Illumina DNA data. The genome size is calculated using the formula: G = K_num_/K_depth_ [20], where K_num_ is the total counts of *k*-mer and K_depth_ is the *k*-mer depth. We generated a *k*-mer profile with Jellyfish (v2.1.3, RRID:SCR_005491) [21], which calculates the *k*-mer number and distribution. We then used two different models to generate estimates of genome size. The first method assumes a Poisson distribution for the *k*-mers. When multiple peaks are observed, the peak with lower *k*-mer frequencies is considered as the result of heterozygosity. The second method, which is integrated into the program GenomeScope, uses a mixed negative binomial model, granting more flexibility in genome size estimation [22]. Using the distribution frequency of 17-mers (Supplementary Fig. S2), the genome sizes were estimated as 453.2 Mb and 445.5 Mb for Stie1 and Stie2, respectively, when K_depth_ was calculated based on a Poisson distribution; while the genome sizes estimated in GenomeScope were 407.6 Mb and 406.8 Mb, respectively (Supplementary Fig. S3).

*De novo* genome assembly conducted with Falcon on the PacBio data (v1.8.7, length_cutoff = 8 kb, length_cutoff_pr = 10 kb, max_diff = 60, max_cov = 75) [23] produced an initial assembly of 510.7 Mb, with a contig N50 of 1.16 Mb (Supplementary Table S2). After the *de novo* assembly, a first round of genome polishing using PacBio subreads improved the accuracy of the assembly. BLASR in SMRT Link [24] mapped all subreads to the initial assembly with the following parameters: "–bestn 5 –minMatch 18 –minSubreadLength 1000 –minAlnLength 500 –minPctSimilarity 70 –minPctAccuracy 70 –hitPolicy randombest." Then Arrow (a function of the SMRT analysis suite) [25] defined consensus sequences. Arrow reaches improved consensus when compared with the legacy Quiver algorithm and is based on a more straightforward hidden Markov model approach [25]. This analysis corrected 2,556,035 insertions, 519,440 deletions, and 1,302,397 substitutions in the draft genome assembly.

To further correct errors in the PacBio-only assembly, the Arrow-corrected genome was polished for two additional rounds with Illumina data using Pilon (v1.20, RRID:SCR_014731) [26]. First, we mapped reads from each individual, separately, to the Arrow-corrected assembly with bwa-mem using default parameters (Version 0.7.12-r1039, RRID:SCR_010910 [27]). Then, we used the output bam file from the individual with higher coverage (99.20% in Stie2 compared to 97.07% in Stie1) for the first round of polishing with Pilon (–mindepth 20), which corrected 87,535 insertions, 44,308 deletions, and 46,678 substitutions. For the second round of polishing, we mapped all Illumina reads (from both Stie1 and Stie2) to the Pilon- and Arrow-corrected assembly with bwa-mem using default parameters. We then ran the resulting bam file through Pilon (–mindepth 20) again, producing an assembly of 512.7 Mb and correcting an additional 71,259 insertions, 123,506 deletions, and 223,395 substitutions.

### Transcriptome analysis

We identified full-length transcripts from the PacBio RNA-seq data following the PacBio IsoSeq analysis pipeline, which includes three steps: classifying, clustering, and mapping. After filtering the low-quality subreads based on signal-to-noise ratio with default parameters, we used SMRT Link to convert raw sequences into a BAM file and retained reads of insert with high quality (minimal full pass: 1, minimal predicted accuracy: 0.8), producing circular consensus sequences, which were then classified into two classes: full-length reads (those that contained both the 5′ primer and 3′ primer with poly-As, 76.43% of all subreads) and non-full-length reads. Next, we conducted isoform clustering with full-length and non-full-length reads using the Iteratively Clustering and Error Correction algorithm in the SMRT analysis software, followed by polishing using the Arrow function [25]. To correct sequencing errors from PacBio, we polished the consensus sequences with Illumina transcriptome sequences using LoRDEC [28] (v0.6, RRID:SCR_015814, with parameters: −k 19, −s 3), resulting in 272,511,198 bp of 118,776 full-length transcripts. Further, we retained only the transcripts that could be aligned to the intermediate genome assembly with GMAP (-n 1) [29], then collapsed them with the python script collapse_isoforms_by_sam.py from SMRT Link package, producing a final set with 22,347 non-redundant transcriptome isoforms. The mean length of all resulting transcripts was 2,881 bp, ranging from 274 to 13,820 bp.

### Heterozygosity estimation

We estimated the heterozygosity from the *k*-mer profile via a comparison to a series of simulated heterozygosities of a model genome (*Arabidopsis thaliana*) [30]. The estimated heterozygosities were 1.10% and 1.06% for Stie1 and Stie2, respectively (Supplementary Fig. S4), which were similar to the results obtained from GenomeScope (1.08% and 1.05%, respectively, Supplementary Fig. S3).

Although the estimated heterozygosity of *S. tienmushanensis* is within the normal range for non-model insects with a published genome, the pooling of DNA from two wild-caught caddisfly adults represents a potential source for inflated heterozygosity. To address this potential issue, we used LAST (v852, RRID:SCR_006119) [31] and Redundans (v 0.14a) [32] to identify redundant contigs in the intermediate assembly. Contigs of the corrected intermediate assembly were aligned against themselves using LAST (v852, RRID:SCR_006119) [31] and Redundans (v 0.14a) [32]. Those contigs with ≥50% of their length overlapping with others at a ≥80% identity were considered redundant; the shorter of the pair was removed from the genome assembly. As a result, 1,472 and 1,474 contigs were identified as redundant by LAST and Redundans, respectively, with 1,471 contigs identified by both programs. The distribution of identity of the redundant contigs identified by LAST (Supplementary Fig. S5) indicated that most had >90% similarity and overlaps with other contigs. We then compared candidate redundant contigs identified by either LAST or Redundans with the full-length transcripts. If a particular candidate was mapped with distinct full-length transcript sequences and also aligned with other contigs at ≤90% identity, it was considered a true contig with unique expressed transcripts and added back into the assembly. We additionally removed short contigs (<1,000 bp) from the genome assembly. In total, 1,498 redundant contigs were removed from the genome assembly in this step.

We screened for potential contamination in the genome assembly with taxon-annotated GC-coverage (TAGC) plots using Blobtools (v1.0) [33]. To identify contaminated contigs, we followed the process outlined by Fu et al. 2017 [17]. In short, we marked a contig as a contaminant if it had all three of the following characteristics: (1) had a best hit to a reference sequence from non-Arthropoda, (2) had no mapping of full-length transcripts, and (3) contained no homologous insect genes from the Benchmarking Universal Single-Copy Orthologs (BUSCO v3.0, RRID:SCR_015008) [34]. Four contigs met these characteristics, and were subsequently removed from the assembly (TAGC plots for the final assembly shown in Supplementary Fig. S6, Table S3).

The final genome assembly of *S. tienmushanensis* is 451.5 Mb, with a contig N50 of 1.29 Mb and a longest contig of 4.76 Mb (Supplementary Table S2). The size of the final assembly is very close to those estimated based on *k*-mer distributions using the Poisson distribution method (453.2 Mb and 445.5 Mb for Stie1 and Stie2, respectively) but larger than those estimated by GenomeScope (407.6 Mb and 406.8 Mb). This discrepancy may reflect the differences in the two algorithms or it may imply possible redundant contigs that were not identified by our filtering procedures. The comparisons among the five available Trichoptera genome assemblies (including *Glossosoma conforme* [35]*, Glyphotaelius pellucidus* [36], *Limnephilus lunatus* provided by i5K [37], and *Sericostoma* sp. HW-2014 [38]) are shown in Table 1.

**Table 1:** Comparison of genome assemblies among five caddisfly genomes

Species	*Stenopsyche tienmushanensis*	*Glossosoma conforme*	*Glyphotaelius pellucidus*	*Limnephilus lunatus*	*Sericostoma* sp. HW-2014
Platform	PacBio + Illumina	Illumina	Illumina	Illumina	Illumina
Assembly accession	v1	ASM334726v1	-	Llun_2.0	ASM300347v1
Sequencing depth	153× + 150×	53.0×	8.12×	80.1×	43.0×
Total length (bp)	451,494,475	604,293,666	757,289,448	1369,180,260	1015,727,762
Scaffold N50 (kb)	1297	16.7	1.47	69.1	3.1
BUSCO (n = 1658)	C:97.6% (S:94.0%,D:3.6%)	C:85.2% (S:84.2%,D:1.0%)	C:22.3% (S:22.1%,D:0.2%)	C:86.7% (S:80.9%,D:5.8%)	C:37.4% (S:37.0%,D:0.4%)
	F:1.1%	F:11.6%	F:39.9%	F:7.6%	F:38.8%
	M:1.3%	M:3.2%	M:37.8%	M:5.7%	M:23.8%

The genome source: *G. conforme* [35], *G. pellucidus* [36], *L. lunatus* from i5K project [37], and *Sericostoma* sp. HW-2014 [38]. BUSCO annotation: C: complete BUSCOs; S: complete and single-copy BUSCOs; D: complete and duplicated BUSCOs; F: fragmented BUSCOs; M: missing BUSCOs.

The completeness of the assembly was assessed using BUSCO (v3.0, RRID:SCR_015008) [34] and the insecta_odb9 gene set [39]. Overall, 97.65% of 1,658 single-copy genes were completely recovered in the full genome assembly, representing a significant improvement over existing caddisfly genomes (Table 1). The high completeness of the assembly is likely due to deep long-read sequencing, which enables the assembly of long and complex regions of the genome.

### Repeat analysis and non-coding RNA annotation

In total, we identified 91,564 simple sequence repeats (simple sequence repeat [SSR], 4,217 with compound format) with the MIcroSAtellite identification tool (MISA, v1.0, RRID:SCR_010765) [40] using default parameters (see Supplementary Table S4 for types of SSR). We identified 1,749,004 bp (0.39% of the genome size) of sequence as full-length long terminal repeat (LTR) transposons, using LTR_finder (v1.06, RRID:SCR_015247) [41] with the parameter "–a ps_scan." We also identified 3,579,704 tandem repeats, accounting for 0.79% of the genome size, using Tandem Repeats Finder (TRF, v4.09) [42] with the following parameters "Match = 2, Mismatch = 7, Delta = 7, PM = 80, PI = 10, Minscore = 20, MaxPeriod = 2000." Next, we used RepeatModeler (v1.0.4, RRID:SCR_015027, [43]) to generate a *de novo* repeat library from the genome (searching engine: rmblast, using default parameters), followed by RepeatMasker (v4.0.7, RRID:SCR_012954) [44] with parameters "-nolow –norna –q –no_is" to search for transposable elements (TEs) from the known Repbase TE library (Repbase21.08) [45] and the *de novo* repeat library we built. In total, we annotated 46,773,887 bp (10.36%) and 156,642,282 bp (34.69%) from RepeatMasker with the Repbase TE library and the *de novo* repeat library, respectively. We also annotated 30,030,332 bp (6.65%) of TE sequences in the genome by similarity using the TE protein reference libraries in RepeatProteinMasker (v4.0.7, *P* < 0.0001, RRID:SCR_012954) [44] using parameters "-noLowSimple −pvalue 0.0001." Overall, 36.76% of the genome was masked as repeats (Table 2, results from different programs in Supplementary Table S5), with those classified as DNA transposons as the most abundant type (17.81% of the genome size).

**Table 2:** Summary of annotated repeats

Type	Combined TEs length (bp)	% of genome
DNA	80,400,946	17.81
LINE	20,688,303	4.58
LTR	1,914,131	0.42
SINE	7,687	0.00
Other	15,267,487	3.38
Unknown	47,696,381	10.56
Total	165,974,935	36.76

"Other" represents a TE that is classified but does not belong to one of our chosen classes. "Unknown" represents TEs that could not be classified.

We annotated rRNA using RNAmmer (v1.2) [46] with default parameters. In addition, we aligned our RNA-seq data to all caddisfly rRNA sequences available in GenBank using Basic Local Alignment Search Tool N (BLASTN) (identity >90%, mapping length for 18S and 28S rRNA >400 bp). We predicted tRNA using tRNAscan-SE (v1.3.1, with default parameters) [47] and annotated snRNA and miRNA using Rfam 11.0 [48] and BLAST with default parameters. In total, we predicted 150 rRNAs (4 28S rRNA genes,1 18S rRNA gene, and 145 5S rRNA genes), 644 tRNAs, 75 snRNAs, and 89 miRNAs.

### Gene prediction

We predicted gene models using three different strategies: *ab initio*, homology-based, and RNA-seq-assisted predictions. We chose 1,000 non-redundant full-length transcripts, each of which contained more than one exon with translated amino acids at <80% identity from each other, for parameter training in the *ab initio* prediction (AUGUSTUS v3.2.2, RRID:SCR_008417) [49] with other parameters as "–UTR = off –gff3 = on –genemodel = complete –strand = both –min_intron_len = 15." For homology-based gene prediction, we aligned the genome to insect proteins obtained from the uniref90 database [50] using TBLASTN with an E-value cutoff of 1e-5, and defined gene structures using GeneWise (v2.4.1, RRID:SCR_015054) [51] with parameters "-genesf −gff −sum −trev/tfor." For RNA-seq-assisted predictions, we used the Program to Assemble Spliced Alignment (PASA) (v2.0.2, RRID:SCR_014656) [52] with the parameter "–ALIGNERS BLAT" to align the transcriptomes to genome sequences with BLAT. We then predicted open reading frames from the resulting PASA gff file using Transdecoder (v5.0.2) [53] with default parameters. Finally, we used EvidenceModeler (EVM, v1.1.1, RRID:SCR_014659) to combine gene models from all three methods (using the following weights for different types of evidence types: 3, 5, and 10 for *ab initio*, homology-based, and RNA-seq-assisted predictions, respectively), followed by PASA (running with the default parameters) to update the final results, including alternative splicing, untranslated regions, and additional genes missed but predicted by PASA (Supplementary Table S6) [54]. All predicted genes were aligned with known transposons by TransposonPSI [55] to remove putative transposon sequences (E-value ≤ 1e-5). In total, 14,672 genes were annotated for *S. tienmushanensis*. Comparisons of the new Trichoptera annotation with four sequenced lepidopterans (*B. mori:* ASM15162 v.1 [56], *D. plexippus* v.3 [57], *H. melpomene* Hmel2.5 [58, 59], *P. xylostella* DBM_FJ_V1.1 [60]) suggested that gene numbers and exon lengths were similar among all species (Supplementary Table S7).

### Functional annotation of protein-coding genes

**Figure 2:**
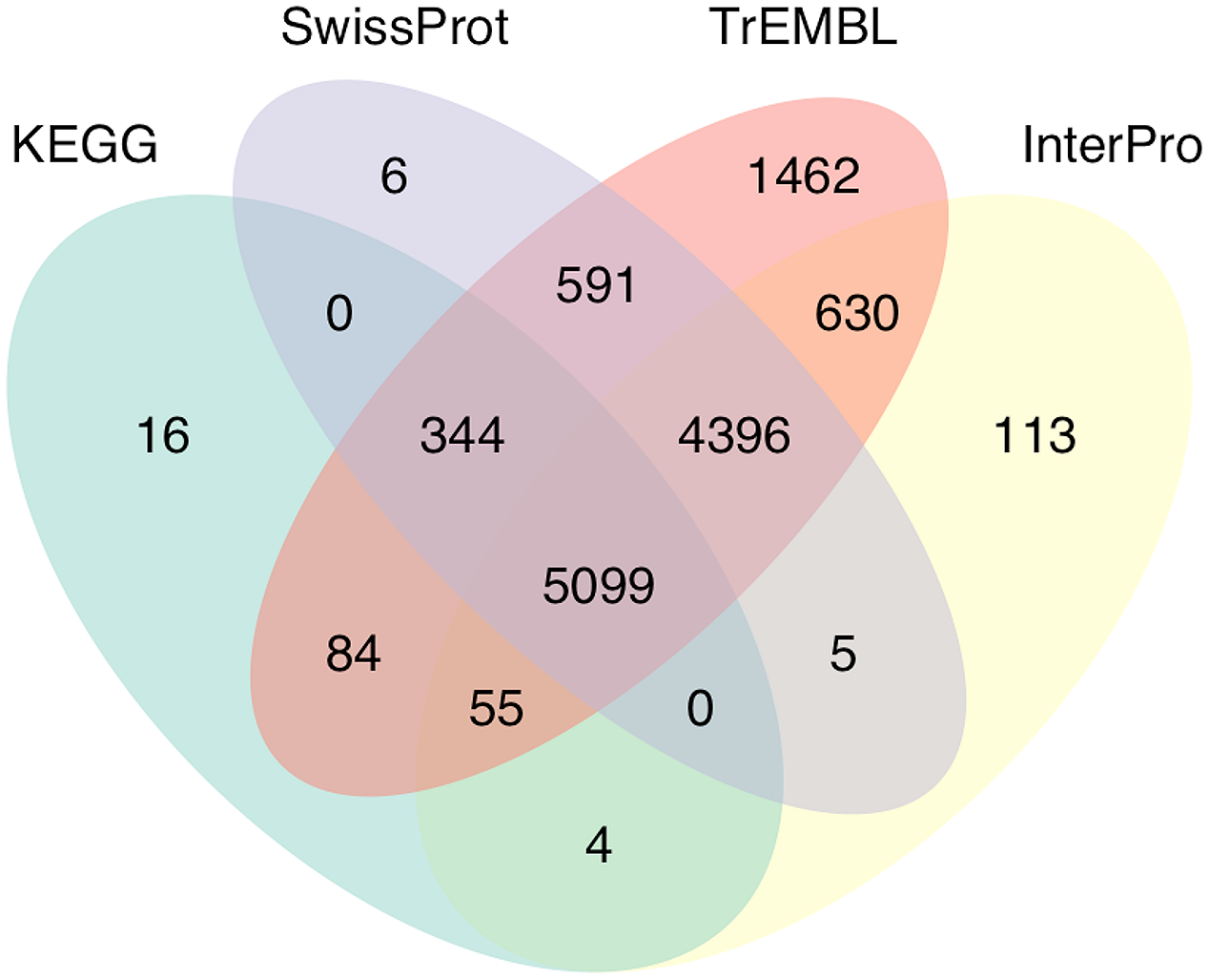
Functional gene annotations using four databases.

Gene functions were assigned based on best match of the predicted proteins to SwissProt and TrEMBL [50] using BLASTP (with E-value ≤ 1e-5), and Kyoto Encyclopedia of Genes and Genomes (KEGG) databases using KAAS [61]. Of the 15,658 annotated proteins encoded by 14,672 genes, including those from alternative splicing, 10,441 (66.68%), 12,661 (80.86%), and 5,602 (35.78%) had significant hits with proteins catalogued in SwissProt, TrEMBL, and KEGG, respectively. In total, 10,302 (65.79%) annotated proteins included motifs/domains identified by InterProScan (v5.21, RRID:SCR_005829) [62] when searched against InterPro databases. Of these, 7,842 genes were assigned to Gene Ontology (GO) [63] IDs with a corresponding InterPro entry (top 20 terms of GO pathway analysis shown in Supplementary Fig. S7). In summary, 12,805 annotated proteins encoded by 11,838 genes were assigned with at least one related function, accounting for 80.68% of the total identified genes in *S. tienmushanensis* (Fig. 2).

### Gene orthology analysis and phylogenetic tree reconstruction

**Figure 3:**
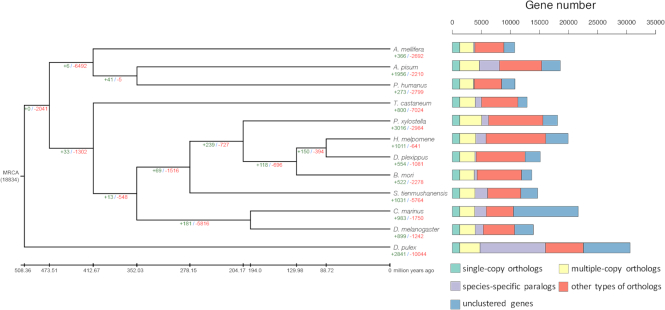
The phylogenetic tree and gene expansion/contraction of 12 arthropod taxa. Multiple-copy orthologs represent the gene groups present in all species with a gene number >1 in at least one species. Species-specific paralogs represent genes uniquely present in only one species. Other types of orthologs represent the gene groups that are absent in some species and not species-specific paralogs. Numbers of expanded gene families are marked in green, while numbers of contracted gene families are marked in red. MRCA: most recent common ancestor. The number below MRCA is the total group number from the OrthoMCL analysis. Note that only some of the gene expansions/contractions are significant.

We constructed a phylogeny using genome-scale orthologous genes from 12 species, including *S. tienmushanensis*, 10 additional insects (*Acyrthosiphon pisum, Apis mellifera, Bombyx mori, Clunio marinus, Danaus plexippus, Drosophila melanogaster, Heliconius melpomene, Tribolium castaneum, Pediculus humanus*, and *Plutella xylostella*), and a crustacean (*Daphnia pulex*) as the outgroup (see Supplementary Table S8 for additional details). Gene orthology was identified using OrthoMCL (version v2.0.9, RRID:SCR_007839) [64] with default parameters. We excluded transcripts from alternative splicing and retained only the longest transcript for each gene. Orthologous proteins from the 12 species were aligned against each other using BLASTP (E-value ≤ 1e-5). Then we used the Markov Clustering (MCL) algorithm to perform a graph clustering of protein orthologs from above. In total, 18,834 gene family clusters were identified, including 1,263 single-copy orthologous genes (Fig. 3).

We used these 1,263 orthologous single-copy genes from the 12 species to construct a phylogenetic tree. Multiple sequence alignments were conducted with MAFFT (version 7.058beta, RRID:SCR_011811) [65] with default parameters, and the protein alignment was transformed to a coding sequence (CDS) alignment. We used Gblocks (version 0.91b, with the parameter −b5 = h) [66, 67] to filter out poorly aligned positions. The phylogenetic tree was constructed using RAxML (version v8.0.19, RRID:SCR_006086) [68] with the GTRGAMMA model and 100 bootstrap replicates. The divergence times among different lineages were estimated with the MCMCTREE package from PAML (version 4.6, RRID:SCR_014932) [69], using parameters "clock = 2, RootAge ≤ 5.30, model = 7, BDparas = 110, kappa_gamma = 62, alpha_gamma = 11, rgene_gamma = 13.7, sigma2_gamma = 11.03." The phylogenetic tree (Fig. 3) confirmed that *S. tiemushanensis* was the sister lineage to Lepidoptera. The divergence time between *S. tienmushanensis* and the three representative Lepidoptera species was generally consistent with earlier results [2].

**Figure 4:**
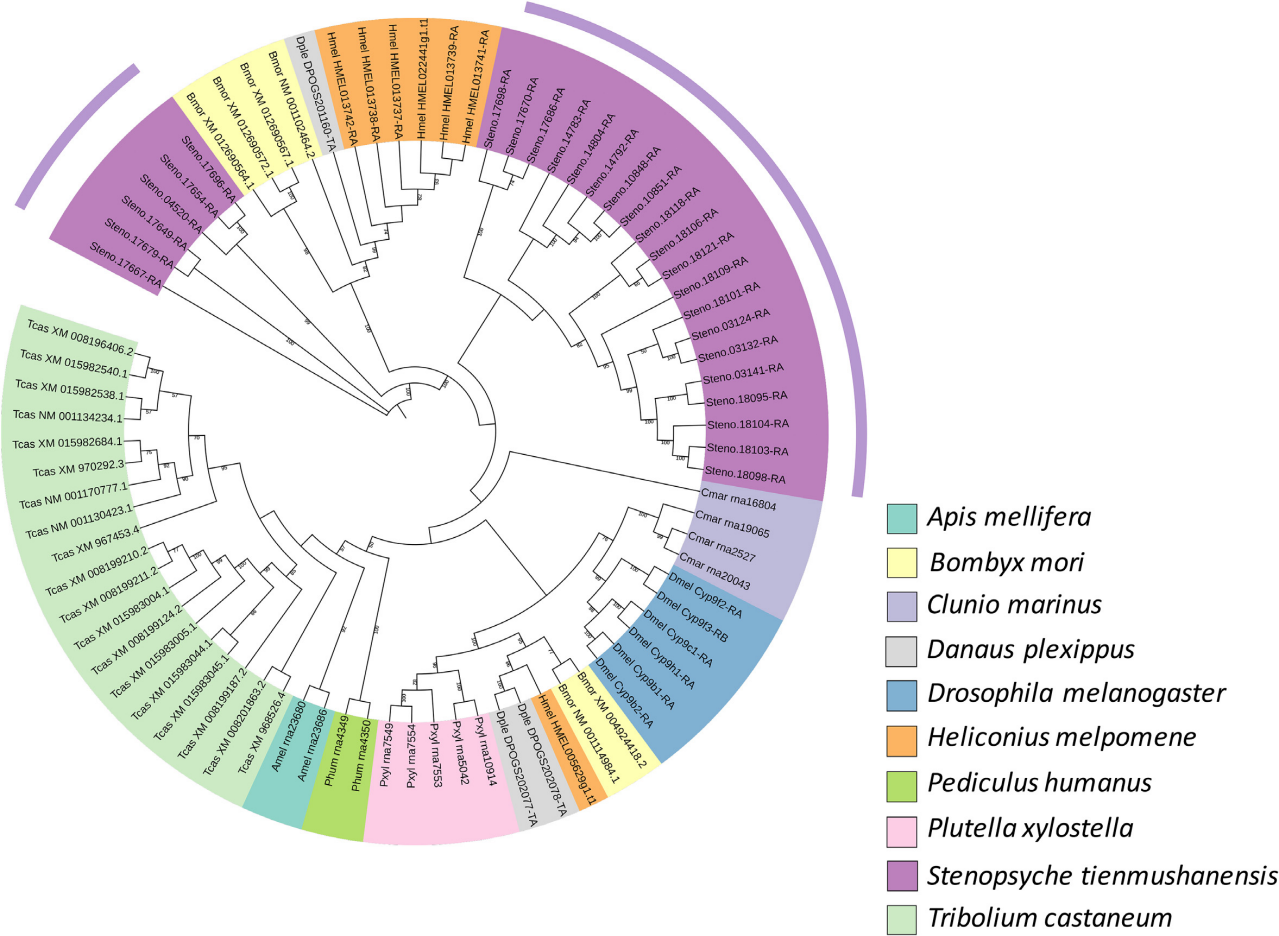
The phylogenetic relationship of the significantly expanded gene groups of cytochrome P450 family in 10 insect species. The phylogeny was constructed using maximum likelihood, showing significant expansions in *S. tienmushanensis*. The bootstrap values are marked on the nodes.

Based on the phylogeny, we conducted analyses on gene family expansions and contractions using CAFE (version 3.1) [70] with default parameters. Compared with sister taxa from Lepidoptera, *S. tienmushanensis* possessed a larger number of contracted gene families and lower number of expanded gene families from the common ancestor (Fig. 3). Among all expanded/contracted groups, 66 gene families showed a significant change in size in *S. tienmushanensis* (*P* < 0.05), in which 63 gene families were significantly expanded. These included cytochrome P450, HSP20, insect cuticle protein, and Histone-lysine N-methyltransferase SETMAR, which is related to DNA double-strand break repair [71, 72]. The expanded cytochrome P450 in the caddisfly was most closely related to the CYP9 family from *D. melanogaster* (Fig. 4), which is functional in the metabolism of insect hormones and in the breakdown of insecticides [73, 74]. We speculate that this expansion may play a role in the adaptation of *S. tienmushanensis* to a wide range of fresh waters with varied pollutants, although further investigations are needed to prove this hypothesis.

**Figure 5:**
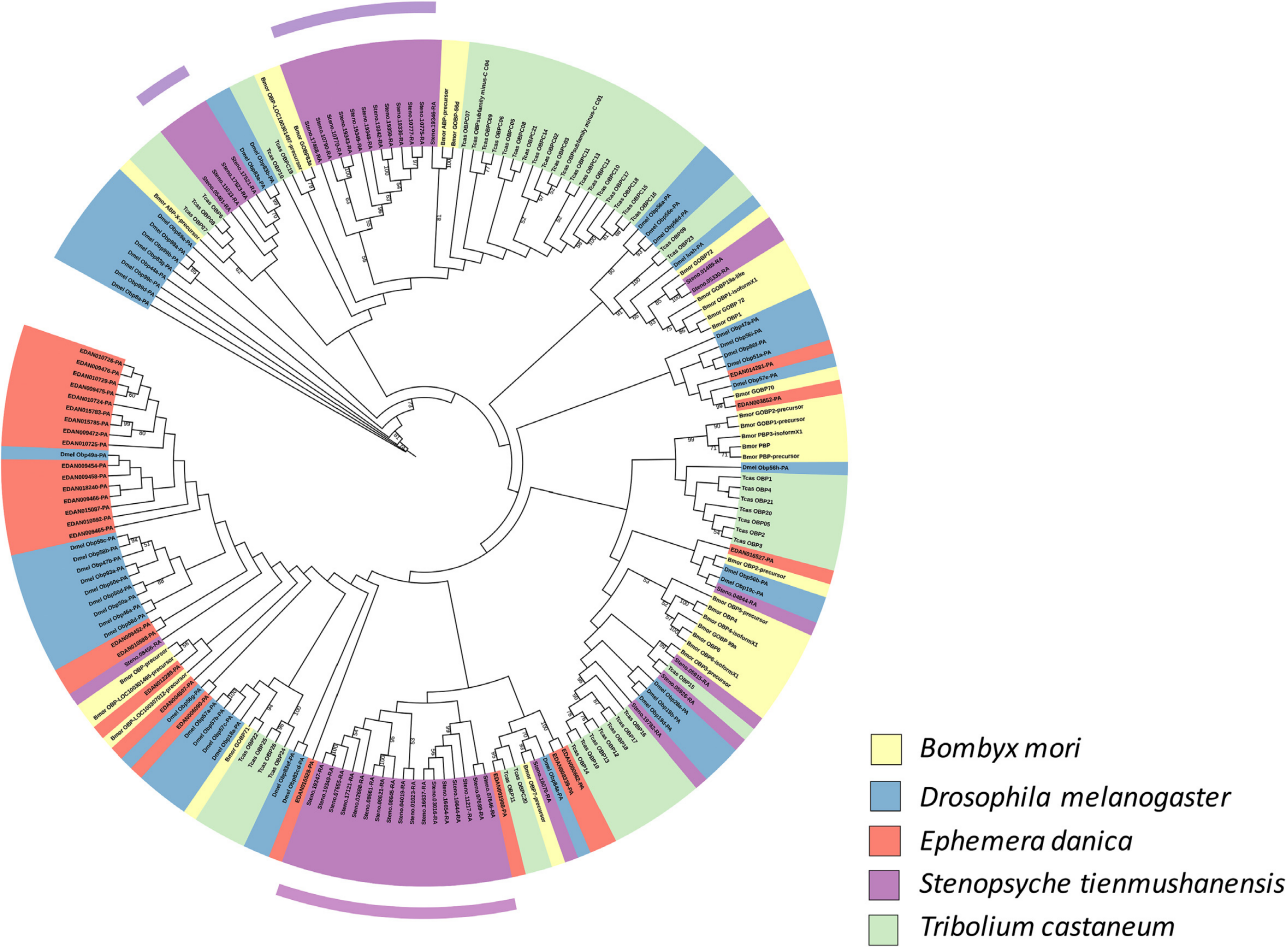
The maximum likelihood tree of odorant-binding proteins (OBPs) in five insect species. The bootstrap values are marked on the nodes. The expanded OBP groups in *S. tienmushanensis* are most closely related to those potentially responsible for pheromone detection in *Drosophila*.

For the species-specific paralogs of *S. tienmushanensis* revealed by the OrthoMCL analysis, GO enrichment (Supplementary Fig. S8) revealed gene expansions of the odorant-binding proteins (OBPs). A phylogeny of the OBPs from *S. tienmushanensis, D. melanogaster* [75], *T. castaneum* [76], and *B. mori* [77] (genome data sources shown in Supplementary Table S8) indicated potential functional relevance of these expansions in the caddisfly genome. Of the expanded OBP gene groups in *S. tienmushanensis*, one was most closely related to OBP83a and OBP83b from *D. melanogaster* (Fig. 5), which are also known as OS-F and OS-E with putative roles in detection of volatile pheromones [75, 78, 79]; another was most closely related to OBP84a from *D. melanogaster*, which is also known as PBPRP-4 (pheromone-binding protein related protein gene) [75]. These uniquely expanded OBPs in *S. tienmushanensis* may be an adaptive genomic feature associated with sex attraction. Because most adult caddisflies do not feed due to reduced mouthpart structures, they are obliged to complete reproduction in a more efficient way, in the relatively short adult stage. Therefore, the OBP expansions in *S. tienmushanensis* may reflect their adaptation in effective mate finding. It is worth noting that OBP expansion is probably not the only mechanism that helps to facilitate reproduction. We examined the mayfly (*Ephemera danica*) genome and did not find convergence on the OBPs. The PBP_GOBP family (PF01395 in Pfam), including pheromone binding proteins (PBP) and general odorant binding proteins (GOBP), was used to search for OBPs in the mayfly genome obtained from the i5K project [37] using HMMER (v3.1b2, RRID:SCR_005305) [80] with default parameters. Although the mayflies are also known to have short life-spans as adults, they may effectively increase their chances in finding mates by forming mating swarms. This behavioral adaptation may explain the discrepancy observed in the genomic features of their OBP genes when compared with the trichopteran genome.

### H-fibroin gene analysis

Previous research on caddisworm silk has revealed that phosphorylation of serines in the H-fibroin protein and the incorporation of multivalent metal ions are responsible for its unique mechanical properties [6, 10]. However, while these features have been revealed as important functional features of caddisworm silk, the genetic underpinnings of silk production have not been fully explored. For example, only partial sequences of the H-fibroin gene have been assembled in previously sequenced transcriptomes [13], presumably due to the inadequacy of short-read technologies in resolving complex genomic features rich in repeats. Here, using long-read PacBio sequencing, we report the first full assembly of the H-fibroin gene complex of a retreat-building caddisworm.

**Figure 6:**
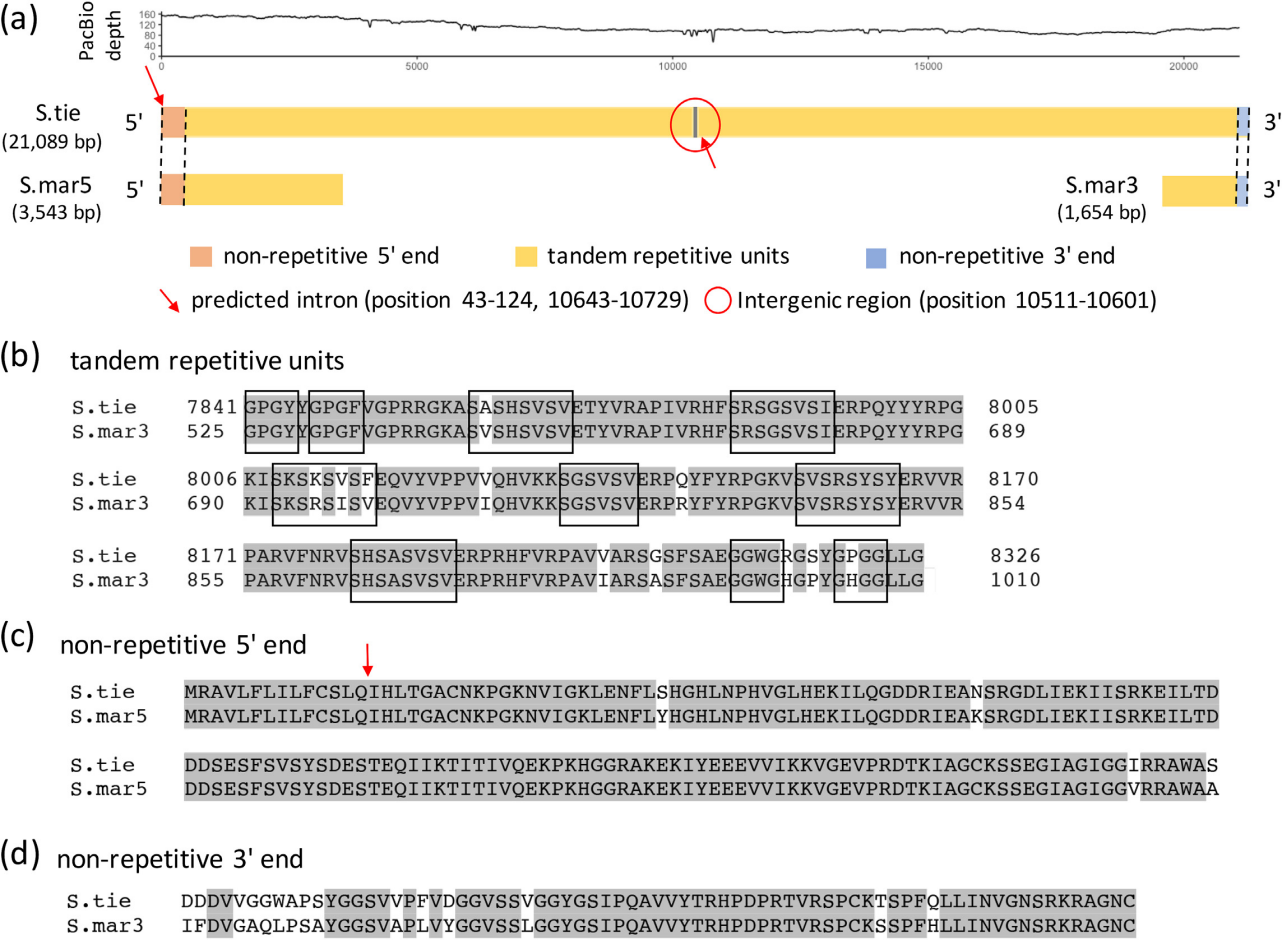
The H-fibroin gene complex in *S. tienmushanensis*. The sequences of H-fibroin gene fragments previously reported from *S. marmorata* are referred from [7, 12]. **(a)** The comparison of H-fibroin genes between *S. tienmushanensis* and *S. marmorata*. The depth of PacBio read coverage is shown in the line plot (smoothed by a sliding window average of 25 bps). The H-fibroin alignment of one representative tandem repetitive unit, non-repetitive 5′ end, and non-repetitive 3′ end between *S. tienmushanensis* and *S. marmorata* was shown in panels **(b-d)**. Identical amino acids in alignment between *S. tienmushanensis* and *S. marmorata* were marked in gray shadow. The start and end positions of the nucleotides were shown in the alignment of the repetitive units. Amino acids in the black box represent the typical motifs of short repeat unit. S.tie: H-fibroin gene complex in *S. tienmushanensis*; S.mar5/S.mar3: the 5′/3′ end nucleotides of H-fibroin mRNA fragments in *S. marmorata*. The marked intron near the 5′ end of the gene complex (position: 43–124) was inferred from the alignment between the non-repetitive 5′ end between *S. tienmushanensis* and *S. marmorata*, positioned between sequences coding for the 14th and 15th amino acids of the N-terminus of the first predicted protein. The other marked intron (position: 10 643–10 729) was identified near the 5′ end of the second predicted gene, positioned between the second and third position in the codon for the 14th amino acid of the second predicted protein.

The genome assembly included a 21 kb region, which was identified as the complete H-fibroin gene complex, including two similarly sized H-fibroin genes separated by a short intergenic region. PacBio sequencing results show a coverage depth of >100× with many reads spanning across large proportions of the gene range, including the intergenic region, ensuring the validity of the assembly (Fig. 6a). The coding regions harbor multiple conserved tandem units with high similarity to a previously reported H-fibroin gene fragment from *Stenopsyche marmorata*, a retreat-making caddisfly from the same genus [7] (GenBank accession number BAM84281, 479 aa in length). The conserved units code typical short H-fibroin repeats, including GGX, SXSXSX, and GPGX, with varied sequences and lengths (Fig. 6b). In addition, the identified region contained both non-repetitive N- and C-termini, homologous to the termini of *S. marmorata* H-fibroin [12] (Figs 6c and 6d), further confirming complete assembly of the gene complex. Interestingly, the N-terminus was found at the beginning of the first gene and the C-terminus was found at the end of the second gene, with the intergenic region occurring between the repetitive regions (Fig. 6a). Both genes encode proteins with the expected molecular mass of H-fibroin, ∼350 kg mol^−1^. This gene structure had not been previously reported and was only possible to determine with the full assembly using long-read sequencing. The assembly of the complete H-fibroin region in our study provides a significant expansion over existing genetic resources on caddisfly H-fibroin genes, which will be important for studying caddisworm silk structure and adaptation to aquatic environments. For future studies, transcriptome and gene expression analysis from larval silk glands will help elucidate additional structural details of H-fibroin.

## Conclusions

The genome presented here is the first high-quality draft genome of a retreat-building caddisfly. With a known diversity of more than 16,000 species, caddisflies are important members of freshwater ecological communities, and their species have been shown to be effective indicators of freshwater health [35, 81, 82]. The research of a host of researchers in freshwater biology and entomology will be positively impacted by the availability of a high-quality draft genome.

In addition to the genome, we present a set of 14,672 annotated genes. This will enable large-scale comparisons with existing genomes, especially those in Lepidoptera. While Trichoptera and Lepidoptera are reciprocally monophyletic and among the strongest supported ordinal level relationships within insects [2, 83], they have highly divergent life histories, with Lepidoptera being primarily terrestrial and the Trichoptera egg, larval, and pupal stages being entirely aquatic. The addition of a high-quality trichopteran genome has the potential to deliver insights into the genetic basis of diverse strategies of insects to adapt to divergent habitats and to uncover the genomic differences between aquatic and terrestrial lifestyles. In particular, the caddisfly genome may provide a deeper understanding of the evolution of the fascinating case-making behaviors and the underwater silk of these aquatic architects.

## Availability of supporting data

All raw sequencing reads have been deposited in the Short Read Archive under project PRJNA436868. The raw sequencing reads, genome assembly, gene models, and other supporting data are available via the *GigaScience* database, GigaDB [84].

## Additional files

**Figure S1:** An adult *Stenopsyche tienmushanensis* attracted to light trap (Photo credit: Mr. Jiahui Hu, China Agricultural University). By comparison, an adult Hydroptilidae is shown resting next to the left foreleg of *Stenopsyche*.

**Figure S2:** Distributions of Illumina 17-mers for samples Stie1 (a) and Stie2 (b). The first peak on the left (depth = 35) is a heterozygous peak, the second peak is the main peak (depth = 70).

**Figure S3:** GenomeScope 17-mer profile plots for Stie1 (a) and Stie2 (b), showing the fitting of the GenomeScope model (black) to the observed *k*-mer frequencies (blue).

**Figure S4:** Comparison of 17-mer depth distributions of samples Stie1 (a) and Stie2 (b) with a series of simulated heterozygosities of a model genome (*Arabidopsis thaliana*). The simulated genome data of *A. thaliana* with different heterozygosity (number before ‘X’ in the key) and appropriate depth (number after ‘X’ in the key) was used.

**Figure S5:** The distribution of the 1472 redundant contigs identified by LAST.

**Figure S6:** Taxon-annotated GC-coverage (TAGC) plots for the final genome assembly of *Stenopsyche tienmushanensis*. Each circle represents one contig in the assembly, with different colors based on the best match to the corresponding taxonomic annotation. The upper- and right- panels show the distribution of the total span (kb) of contigs for a given GC proportion or coverage.

**Figure S7:** Top 20 terms in the GO pathway analysis.

**Figure S8:** Top 10 terms in the GO pathway analysis of the species-specific paralog genes.

**Table S1:** Sequencing data counts.

**Table S2:** Statistics of the initial and final genome assemblies.

**Table S3:** Statistics of Taxon-annotated GC-coverage (TAGC) analysis (see Supplementary Table S3.xlsx). ‘Iso-Seq’ represents contigs mapped by full-length transcripts sequenced by PacBio Iso-Seq. ‘BUSCO’ represents contigs containing insect homologous genes categorized in BUSCO.

**Table S4:** Numbers of different types of Simple Sequence Repeat (SSR).

**Table S5:** Annotated repeat sequences from different methods.

**Table S6:** Statistics of gene prediction based on three methods.

**Table S7:** Comparison of gene annotations with representative lepidopterans.

**Table S8:** Genome data sources of the 11 arthropod species used in evolutionary analysis.

## Abbreviations

BLAST: Basic Local Alignment Search Tool; BUSCO: Benchmarking Universal Single-Copy Orthologs; GO: Gene Ontology; KEGG: Kyoto Encyclopedia of Genes and Genomes; LTR: long terminal repeat; MCL: Markov Clustering; OBP: odorant-binding protein; PacBio: Pacific Biosciences; PASA: Program to Assemble Spliced Alignment; PE: paired-end; RNA-seq: RNA sequencing; SMRT: single-molecule real-time; SSR: simple sequence repeat; TAGC: taxon-annotated GC-Coverage; TE: transposable element; TRF: tandem repeat finder.

## Competing interests

The authors declare that there have no competing interests.

## Funding

X.Z. is supported by the National Science Foundation of China (31772493), Beijing Advanced Innovation Center for Food Nutrition and Human Health, and the Chinese Universities Scientific Fund (2017QC114 and 2018QC133) through China Agricultural University.

## Author contributions

X.Z. designed the study. S.L., M.T., and P.B.F. conducted genome analysis and assembly. X.Z., S.L., P.B.F., and M.T. collected the specimens. P.B.F. and R.J.S. led analysis of the H-fibroin genes. All authors participated in writing and proofing of the manuscript.

## Supplementary Material

giga-d-18-00136_original_submission.pdf

giga-d-18-00136_revision_1.pdf

giga-d-18-00136_revision_2.pdf

response_to_reviewer_comments_original_submission.pdf

response_to_reviewer_comments_revision_1.pdf

reviewer_1_report_(original_submission) -- Scott Hotaling 7/19/2018 Reviewed

reviewer_1_report_revsion_1 -- Scott Hotaling 10/28/2018 Reviewed

reviewer_2_original_submission_(attachment).docx

reviewer_2_report_(original_submission) -- Reuben William Nowell, Ph.D. 7/24/2018 Reviewed

Supplemental Files
