# Supplementary material for: The genome of an underwater architect, the caddisfly Stenopsyche tienmushanensis Hwang (Insecta: Trichoptera)
Source: Gigascience. 2018 Nov 23;7(12):giy143. doi: 10.1093/gigascience/giy143 (PMC6302954; doi:10.1093/gigascience/giy143)
Supplement: giga-d-18-00136_original_submission.pdf [file giy143_giga-d-18-00136_original_submission.pdf]

GigaScience

The genome of an underwater architect, the caddisfly Stenopsyche tienmushanensis Hwang (Insecta: Trichoptera)

--Manuscript Draft--

**Manuscript Number:**	GIGA-D-18-00136
**Full Title:**	The genome of an underwater architect, the caddisfly Stenopsyche tienmushanensis Hwang (Insecta: Trichoptera)
**Article Type:**	Data Note
**Funding Information:**	National Natural Science Foundation of China (31772493) — Dr. Xin Zhou<br>Chinese Universities Scientific Fund (2017QC114) — Dr. Xin Zhou<br>Chinese Universities Scientific Fund (2018QC133) — Dr. Xin Zhou
**Abstract:**	Background<br><br>Caddisflies (Insecta: Trichoptera) are a highly adapted freshwater group of insects split from a common ancestor with Lepidoptera. They are the most diverse (with > 16,000 species) of the strictly aquatic insect orders and widely employed as bio-indicators in water quality assessment and monitoring. Among the numerous adaptations to the aquatic habitats, caddisfly larvae use their silk and materials from the environment (stones, sticks, leaf matter and etc.) to build architectures such as fixed retreats and cases. Understanding how caddisflies have adapted to the aquatic habitats will help explain the evolution of the group.<br><br>Findings<br><br>We sequenced a retreat-maker caddisfly Stenopsyche tienmushanensis Hwang and reported a high-quality genome assembly from both Illumina and PacBio sequencing. In total, 89.0 Gb of PacBio data, and 90.2 Gb of Illumina data were generated. The assembled genome is 453.1 Mb with a contig N50 of 1.29 Mb and a longest contig of 4.76 Mb, covering 97.65% of the 1,658 insect single-copy genes via BUSCO assessment. The genome is comprised of 36.73% repetitive elements and 14,687 protein-coding genes were predicted. The new genome sequences revealed gene expansions in specific groups of the cytochrome P450 family and olfactory binding proteins, suggesting potential genomic features associated with pollutant tolerance and mate finding. In addition, the fully resolved assembly of the highly repetitive H-fibroin gene was achieved, which is the major protein component of caddisfly larval silk.<br><br>Conclusions<br><br>We reported the draft genome of Stenopsyche tienmushanensis, the highest quality caddisfly genome so far. The genome information will be an important resource for the study of caddisflies, and may shed light on the evolution of aquatic insects.
**Corresponding Author:**	Xin Zhou<br><br>CHINA
**Corresponding Author Secondary Information:**	
**Corresponding Author's Institution:**	
**Corresponding Author's Secondary Institution:**	
**First Author:**	Shiqi Luo
**First Author Secondary Information:**	

Powered by Editorial Manager® and ProduXion Manager® from Aries Systems Corporation

**Order of Authors:**	Shiqi Luo
	Min Tang
	Paul B. Frandsen
	Russell J. Stewart
	Xin Zhou
**Order of Authors Secondary Information:**	
**Additional Information:**	
**Question**	**Response**
Are you submitting this manuscript to a special series or article collection?	No
**Experimental design and statistics**<br><br>Full details of the experimental design and statistical methods used should be given in the Methods section, as detailed in our Minimum Standards Reporting Checklist. Information essential to interpreting the data presented should be made available in the figure legends.<br><br>Have you included all the information requested in your manuscript?	Yes
**Resources**<br><br>A description of all resources used, including antibodies, cell lines, animals and software tools, with enough information to allow them to be uniquely identified, should be included in the Methods section. Authors are strongly encouraged to cite Research Resource Identifiers (RRIDs) for antibodies, model organisms and tools, where possible.<br><br>Have you included the information requested as detailed in our Minimum Standards Reporting Checklist?	Yes
**Availability of data and materials**<br><br>All datasets and code on which the conclusions of the paper rely must be	Yes

either included in your submission or deposited in publicly available repositories (where available and ethically appropriate), referencing such data using a unique identifier in the references and in the "Availability of Data and Materials" section of your manuscript.<br><br>Have you have met the above requirement as detailed in our Minimum Standards Reporting Checklist?	

Manuscript

Click here to view linked References

Click here to access/download;Manuscript;Main_text_20180702.docx

The genome of an underwater architect, the caddisfly *Stenopsyche tienmushanensis* Hwang (Insecta: Trichoptera)

Shiqi Luo[1], Min Tang[1], Paul B. Frandsen[2], Russell J. Stewart[3], and Xin Zhou[1*]

[1] Beijing Advanced Innovation Center for Food Nutrition and Human Health, Department of Entomology, College of Plant Protection, China Agricultural University, Beijing, China 100193

[2] Department of Plant and Wildlife Sciences, Brigham Young University, Provo, UT 84602 USA

[3] Department of Bioengineering, University of Utah, Salt Lake City, UT 84112 USA

Shiqi Luo: shiqi_luo@cau.edu.cn, http://orcid.org/0000-0002-0506-2230

Min Tang: mintang_bio@outlook.com, http://orcid.org/0000-0002-6021-7282

Paul B. Frandsen: paul_frandsen@byu.edu, http://orcid.org/0000-0002-4801-7579

Russell J. Stewart: russell.stewart@utah.edu, https://orcid.org/0000-0002-8389-8877

Xin Zhou: xinzhoucaddis@icloud.com, http://orcid.org/0000-0002-1407-7952

*Correspondence should be address to XZ (xinzhoucaddis@icloud.com)

Abstract

Background: Caddisflies (Insecta: Trichoptera) are a highly adapted freshwater group of insects split from a common ancestor with Lepidoptera. They are the most diverse (with > 16,000 species) of the strictly aquatic insect orders and widely employed as bio-indicators in water quality assessment and monitoring. Among the numerous adaptations to the aquatic habitats, caddisfly larvae use their silk and materials from the

environment (stones, sticks, leaf matter and etc.) to build architectures such as fixed retreats and cases. Understanding how caddisflies have adapted to the aquatic habitats will help explain the evolution of the group. **Findings:** We sequenced a retreat-maker caddisfly *Stenopsyche tienmushanensis* Hwang and reported a high-quality genome assembly from both Illumina and PacBio sequencing. In total, 89.0 Gb of PacBio data, and 90.2 Gb of Illumina data were generated. The assembled genome is 453.1 Mb with a contig N50 of 1.29 Mb and a longest contig of 4.76 Mb, covering 97.65% of the 1,658 insect single-copy genes via BUSCO assessment. The genome is comprised of 36.73% repetitive elements and 14,687 protein-coding genes were predicted. The new genome sequences revealed gene expansions in specific groups of the cytochrome P450 family and olfactory binding proteins, suggesting potential genomic features associated with pollutant tolerance and mate finding. In addition, the fully resolved assembly of the highly repetitive H-fibroin gene was achieved, which is the major protein component of caddisfly larval silk. **Conclusions:** We reported the draft genome of *Stenopsyche tienmushanensis*, the highest quality caddisfly genome so far. The genome information will be an important resource for the study of caddisflies, and may shed light on the evolution of aquatic insects.

Keywords

caddisworm, caddisfly, aquatic insect, freshwater adaptation, silk, H-fibroin, PacBio

Data Description

Comprising >16,000 species, caddisflies (Insecta: Trichoptera) are the most diverse of the strictly aquatic insect orders, distributed worldwide except for Antarctica [1]. This highly adapted freshwater group split from a common ancestor with lepidopterans

(moths and butterflies) more than 200 mya. The transition between terrestrial and aquatic (freshwater) habitat has occurred multiple times independently within insects, with caddisflies representing one of the most recent examples [2]. This radical transition would require numerous adaptations in morphological, physiological and molecular traits. Understanding these adaptations will help explain how insects, in general, have evolved as one of the most successful and abundant class of animals on the planet, and how caddisflies, in particular, have adapted to a wide range of freshwater and marine habitats. Identifying the genomic underpinnings of the adaptive mechanisms of caddisflies will improve our knowledge of these thriving aquatic insects that, as major contributors to freshwater biodiversity, have been widely employed as bio-indicators in water quality assessment and monitoring [3].

In addition, caddisflies are of technological interest because, like their terrestrial moth and butterfly relatives, their larvae spin silk. Unlike terrestrial silks, caddisfly larval silk is adapted to be spun from liquid silk dope into tough viscoelastic fibers while fully submerged in water. Caddisfly larvae (caddisworms) use their silk as an adhesive tape to construct a wide variety of composite structures using stones, sticks, leaf matter, and other sediment gathered from the benthos of freshwater rivers, lakes, streams, and marine tidal pools [4]. The larval architectures are sub-order dependent, and include transportable tube cases that provide camouflage and physical protection (suborder Integripalpia), stationary fixed retreats with silk nets for capturing food (suborder Annulipalpia), and rigid silk cases for pupation (suborder “Spicipalpia”) [5]. The distinct and varied deployments of their underwater silk are responsible, in large part, for the penetration of caddisworms into diverse aquatic habitats.

The major protein component of caddisworm silk is H-fibroin, a high molecular weight protein with a blocky, highly repetitive primary sequence. Caddisworm H-

fibroins are extensively phosphorylated on repeating serine-rich motifs with the sequence $({}_{p}SX)_n$, where pS is phosphoserine, X is a hydrophobic amino acid, and n=2-6 [6, 7]. The $({}_{p}SX)_n$ motifs form divalent metal ion-stabilized β-domains that are responsible for the strength, toughness, and energy-dissipating self-recovery of caddisworm silk [8-10]. Currently, only incomplete H-fibroins sequences are available through a GenBank search because it has not been possible to obtain the full-length sequence from cDNAs [11, 12] or to assemble the highly repetitive sequence *de novo* from short-read RNA seq data [13] in the absence of a complete caddisfly genome.

As both an underwater adhesive and a tough fully hydrated metallofiber, caddisworm silk may provide new insights into the mimetic design of tough adhesive materials for use in watery environments. The high-quality draft genome of a caddisfly, which includes the full assembly of the H-fibroin gene, will be invaluable for further identifying and characterizing the enzymes [14] and structural protein components of caddisworm silks.

Sampling, taxonomy and sample preparation

The caddisfly *Stenopsyche tienmushanensis* Hwang 1957 (Fig. 1, NCBI taxonomy ID: 1560151) is only known from China, representing one of the first caddisfly species described by Chinese taxonomists [15]. The distribution range of the species was recently reviewed as confined in the Central China Region [16]. The larvae are of typical lotic species adapted to a wide range of running waters, from prestine creeks to disturbed streams, displaying tolerance to various levels of pullutants.

Adult caddisfly specimens were collected using a light trap by the Yongding River, at Yanchi Town in Beijing, China (altitude 292m, 40.03° N, 115.48° E) in 2017, which is the most northern record for the species. All specimens used in this study were collected at the same site on the same night. Specimens were kept alive on ice then

transferred to -80 °C till extraction. Two female *Stenopsyche* adults (Stie1, Stie2) were used for genome sequencing because DNA quantity from a single specimen was not sufficient for PacBio sequencing. And a third female individual (Stie3) was extracted for RNA and transcriptome sequencing. DNA and RNA were extracted from whole bodies excluding guts. DNA was extracted using the same protocol by Fu *et al.* [17]. RNA was extracted with TRIzol. Taxonomic identification was made using male morphology by XZ and confirmed by COI barcodes.

Genome and transcriptome sequencing

For both Stie1 and Stie2 samples, about 270 millon 150 bp paired-end (PE) reads (80.45 Gb in total for the two samples, details shown in Table S1) were generated from a 400 bp insert-library, using Illumina's Hiseq X ten sequencing platform at WuXi AppTec (Shanghai, China). A genome survey was carried out using Jellyfish (v2.1.3, RRID:SCR_005491) [18]. Using the distribution frequency of 17-mers, the genome size was estimated as 453.2 Mb and 445.5 Mb for Stie1 and Stie2 respectively (Fig. S1). A 1~1.1% heterozygosity rate was estimated using the *Arabidopsis thaliana* genome (Fig. S2).

The remaining DNA from Stie1 and Stie2 were combined for PacBio sequencing on Sequel SMRT cells 1M v2 (PacBio p/n101-008-000), with one movie of 600 minutes at the Genome Center of Nextomics (Wuhan, China). 78.72 Gb of subreads were produced with a mean subread length of 7.6 Kb (Table S1).

RNA extracted from Stie3 was used for transcriptome sequencing on Illumina Hiseq X ten (insert-size of 180 bp, 150PE) and PacBio Sequel system (library size 0.5-6k), which produced 9.72 Gb and 10.31 Gb data, respectively (Table S1). Following PacBio's instruction, PacBio reads were corrected by SMRT link 5.0.1 (https://github.com/PacificBiosciences/SMRT-Link), excluding nearly half reads.

Reads containing both 5' primer and 3' primer with polyAs were identified as full-length transcripts (76.43%) and retained for further analysis. After clustering using the ICE algorithm and correction using Arrow [19] , a total of 118,776 consensus sequences were further revised by LoRDEC [20] using Illumina transcriptome sequences, resulting in 272,511,198 bp of full-length transcripts.

Genome assembly

De novo assembly was performed using PacBio data and Falcon (v1.8.7, length_cutoff = 8 Kb, length_cutoff_pr = 10 Kb, max_diff = 60, max_cov = 75) [21], producing an intermediate assembly of 510.7 Mb, with a contig N50 of 1.16 Mb (Table S2). This assembly was rectified by PacBio reads using Arrow [19], which corrected 2,556,035 insertions, 519,440 deletions and 1,302,397 substitutions. Illumina reads from samples Stie1 and Stie2 were mapped against the PacBio assembly using BWA (Version 0.7.12-r1039, RRID:SCR_010910) [22], covering 97.07% and 99.20% of corrected assemblies, respectively. The output bam files of Stie2 with higher coverage was applied for another revision using Pilon (v1.20, RRID:SCR_014731) [23], which corrected 87,535 insertions, 44,308 deletions and 46,678 substitutions. Then the assembly was corrected again with all Illumina reads, producing an intermediate assembly of 512.7 Mb, with 71,259 insertions, 123,506 deletions and 223,395 substitutions corrected.

After removing short contigs (< 1,000 bp), contigs of the corrected intermediate assembly were BLASTed against themselves using LAST (v852, RRID:SCR_006119) [24]. Those contigs with ≥ 50% of their length overlapping with others at a ≥ 80% identity were considered redundant and the shorter contig of the pair was removed from the genome assembly. If a redundant contig identified in the previous step was mapped with distinct full-length transcriptome sequences and aligned with other contigs at ≤ 90% identity, it was subsequently added back to the final genome assembly. The final

genome assembly of the *S. tienmushanensis* contains 453.1 Mb with a contig N50 of 1.29 Mb, with a longest contig of 4.76 Mb (Statistics of the genome assembly in Table S2). The comparisons among three available Trichoptera genome assemblies (the other two genome assemblies from *Limnephilus lunatus* provided by i5K [25] and *Glyphotaelius pellucidus* [26]) were shown in Table 1.

Genome quality and completeness

Of the full-length transcripts generated from PacBio, 94.55% were successfully mapped against the final genome assembly using GMAP (-n 1) [27]. The completeness of the assembly was assessed using Benchmarking Universal Single-Copy Orthologs (BUSCO v3.0, RRID:SCR_015008) [28] and the insecta_odb9 gene set [29]. 97.65% of 1,658 single-copy genes were completely recovered in the full genome assembly, representing a significant improvement over existing caddisfly genomes (Table 1). The high completeness of the assembly is likely due to deep long read sequencing, which enables the assembly of long and complex regions of the genome.

Repeat analysis and ncRNA annotation

In total, 91,871 simple sequence repeats (SSR) (Table S3, S4) were identified with the MIcroSAtellite identification tool (MISA, v1.0, RRID:SCR_010765) [30] using default parameters. To identify transposable elements (TEs) and tandem repeats, we used LTR_finder (v1.06, RRID: SCR_015247) [31] and Tandem Repeats Finder (TRF, v4.09), respectively [32]. When searching for full-length LTR transposons, 1,749,004 bp (0.39% of the genome size) sequences were identified. We also identified 3,587,745 tandem repeats, accounting for 0.79% of the genome size. Next, we used RepeatModeler (v1.0.4, RRID:SCR_015027, http://www.repeatmasker.org) to generate a *de novo* repeat library from the genome (searching engine: rmblast, using default parameters), followed by RepeatMasker (v4.0.7, RRID:SCR_012954) [33] to

search for TEs from the known Repbase TE library (Repbase21.08) [34] and the *de novo* repeat library we built. Totally, we annotated 46,896,120 bp (10.35%) and 157,078,944 bp (34.67%) from RepeatMasker with Repbase TE library and the *de novo* repeat library respectively. We also annotated 30,118,277 bp (6.65%) TE sequences in the genome by similarity using the TE protein reference libraries in RepeatProteinMasker (v4.0.7, *P* < 0.0001, RRID:SCR_012954) [33]. In total, 36.73% of the genome were masked as repeats (Table 2, results from different softwares in Table S5), and DNA trasnsposon was the most abundant type (17.79% of the genome size).

Non-coding RNA (ncRNA) was annotated with different methods. The rRNA was annotated with RNAmmer (v1.2) [35], and and by aligning to all the caddisfly rRNA sequences in NCBI Genbank with BLASTN (identity > 90%, mapping length for 18s and 28s rRNA > 400 bp). The tRNA was predicted using tRNAscan-SE (v1.3.1, with default parameters) [36]. The snRNA and miRNA were annotated by aligning to Rfam 11.0 [37] with BLAST and default parameters. In total, we predicted 151 rRNAs (four 28S rRNA genes, one 18s rRNA gene and 146 5S rRNA genes), 646 tRNAs, 150 snRNAs and 89 miRNAs.

Gene prediction

The gene models were predicted using three different prediction strategies: *ab initio*, homology-based and RNA-seq-assisted predictions. The *ab initio* predictions were conducted with AUGUSTUS (v3.2.2, RRID:SCR_008417) [38]. One thousand transcripts, which were highly complete and non-redundant, were chosen for training, each including more than one exon with translated amino acids at < 80% identity from each other. Using the model parameters from training, we conducted *ab initio* gene prediction with AUGUSTUS. For homology-based gene prediction, we aligned the

genome to insect proteins from the uniref90 database [39] using TBLASTN with an E-value cutoff of 1e-5. We used GeneWise (v2.4.1, RRID:SCR 015054) [40] to define gene structures and PASA (v2.0.2, RRID:SCR_014656) [41] to align the transcriptome to the genome sequences with BLAT. After successful alignment of the transcriptome, we used transdecoder (v5.0.2) [41] to predict ORFs from the resulting PASA gff file. Finally, we used EVM (EvidenceModeler, v1.1.1, RRID:SCR_014659) [42] to combine gene models concluded from the three different methods, followed by PASA to update the final results, including alternative splicing, UTRs and additional genes missed during earlier stages (details in Table S6). Finally, all predicted genes were aligned with known transposons by Transposon PSI (http://transposonpsi.sourceforge.net/). Candidate genes showing high identity to transposons (with E-value ≤ 1e-5) were removed from the final gene set. In summary, we annotated a total of 14,687 genes. We compared our Trichoptera annotation with four sequenced lepidopterans in Table S7 (The source of genome assemblies: *B. mori* : ASM15162 v.1 [43], *D. plexippus* v.3 [44], *H. melpomene* Hmel2.5 [45, 46] , *P. xylostella* DBM_FJ_V1.1 [47]).

Functional annotation of protein-coding genes

Gene functions were assigned according to the best match by aligning protein sequences predicted from the *S. tienmushanensis* genome to SwissProt, TrEMBL [39] using BLASTP (with E-value ≤ 1e-5), and Kyoto Encyclopedia of Genes and Genomes (KEGG) databases using KAAS [48]. In all the 15,673 annotated proteins (including proteins from alternative splicing) from the 14,687 genes, 10,444 (66.64%), 12,667 (80.82%) and 5,603 (35.75%) annotated proteins had significant hits with proteins in SwissProt, TrEMBL and KEGG, respectively. Totally, 10,304 (65.74%) annotated proteins included motifs/domains identified by InterProScan (v5.21, RRID:SCR

005829) [49] when searched against InterPro databases. Of these, 7,843 genes were assigned Gene Ontology (GO) [50] IDs with the corresponding InterPro entry (top 20 terms of GO pathway analysis shown in Fig. S3). In summary, 12,811 annotated proteins from 11,844 genes were assigned with at least one related function, accounting for about 87.23% of total identified genes in *S. tienmushanensis* (Fig. 2).

Gene orthology analysis and phylogenetic tree

We used predicted coding genes from *S. tienmushanensis*, 10 sequenced insect genomes (*Acyrthosiphon pisum*, *Apis mellifera*, *Bombyx mori*, *Clunio marinus*, *Danaus plexippus*, *Drosophila melanogaster*, *Heliconius melpomene*, *Tribolium castaneum*, *Pediculus humanus* and *Plutella xylostella*) and a crustacean (*Daphnia pulex*) to infer gene orthology and construct the phylogenetic tree (download address for each genome was shown in Table S8). Gene orthology was identified using OrthoMCL (version v2.0.9, RRID: SCR_007839) [51] with default parameters. Firstly, we filtered the transcripts from alternative splicing and only the longest transcript was retained for each gene. All proteins from the 12 species were aligned against each other using BLASTP (E-value $\leq$ 1e-5). Then the Markov Clustering Algorithm (MCL) was used to perform a graph clustering of protein orthologs from above. Totally, 18,834 gene family clusters were identified, with 1,263 single-copy orthologous genes (Fig. 3).

We used these 1,263 orthologous single-copy genes from the 12 species to construct the phylogenetic tree. Multiple sequence alignments were conducted with MAFFT (version 7.058beta, RRID: SCR_011811) [52], and the protein alignment was transformed to the alignment of CDS. The region with low quality was filtered with Gblocks (version 0.91b) [53, 54]. The phylogenetic tree was constructed using RaxML (version v8.0.19, RRID: SCR_006086) [55] with the GTRGAMMA model and 100 bootstrap replicates, and *Daphnia pulex* was used as the outgroup. The divergence times

among different lineages were estimated with the MCMCTREE package from PAML (version 4.6, RRID: SCR_014932) [56], using parameters "clock = 2, RootAge ≤ 5.30, model = 7, BDparas = 110, kappa_gamma = 62, alpha_gamma = 11, rgene_gamma = 13.7, sigma2_gamma = 11.03". The phylogenetic tree (Fig. 3) confirmed that *S. tiemushanesis* was the sister lineage to Lepidopera.

The analyses of gene family expansions and contractions were conducted using CAFE (version 3.1) [57] with default parameters. Compared with sister taxa from Lepidoptera, *S. tienmushanensis* possesses a larger number of contracted gene families and reduced number of expanded gene families from the common ancestor (Fig. 3). Among all expanded/contracted groups, 66 gene families showed significant changs in sizes in *S. tienmushanensis* ($P < 0.05$), in which 63 gene families were significantly expanded. These include cytochrome P450, HSP20, insect cuticle protein, and Histone-lysine N-methyltransferase SETMAR, which is related to DNA double-strand break repair [58, 59]. The expanded cytochrome P450 in the caddisfly is most closely related to the CYP9 family from *D. melanogaster* (Fig. 4), which are functional in the metabolism of insect hormones and in the breakdown of insecticides [60, 61]. We speculate that this expansion may play a role in the adaptation of *S. tienmushanensis* to a wide range of freshwaters with varied pollutants.

For the species-specific paralogs of *S. tienmushanensis* in the OrthoMCL analysis, GO enrichment (Fig. S4) showed species-specific expansion of the odorant binding proteins (OBPs). OBPs of *D. melanogaster*, *T. castaneum* and *B. mori* were used as references based on genomic annotations (genome data sources shown in Table S8). The PBP_GOBP (PF01395) family was used to search for OBPs in the mayfly *Ephemera danica* genome obtained from the *i*5K project [25], which is also an aquatic insect, using HMMER (v3.1b2, RRID: SCR_005305) [62]. Of the two expanded OBP

gene groups in *S. tienmushanensis*, one is closely related to OBP83a and OBP83b from *D. melanogaster* (Fig. 5), which are also known as OS-F and OS-E with putative roles in detection of volatile pheromones [63-65]; and the other is closely related to OBP84a from *D. melanogaster*, which is also known as PBPRP-4 (pheromone-binding protein related protein gene) [65]. Caddisflies are generally short-lived as adults, a life-stage with the main function of reproduction. Therefore, the uniquely expanded OBPs in *S. tienmushanensis* may be an adaptive genomic feature associated with their high efficacy in finding mates.

H-fibroin gene analysis

Previous research on caddisfly silk has revealed that extensive phosphorylation of serines in the H-fibroin protein and the incorporation of multivalent metal ions is responsible for its unique mechanical robustness in a freshwater environment [6, 10]. However, while these features have been revealed as important functional features of caddisfly silk, the genetic underpinnings of silk production has not been fully fleshed out. For example, only partial sequences of the H-fibroin gene have been assembled in previously sequenced transcriptomes [13], presumably due to the inadequacy of short read technologies in resolving complex genomic features rich in repeats. Here, using long read PacBio sequencing, we report the first fully resolved assembly of the H-fibroin gene in retreat-making caddisflies.

The genome assembly included a 21kb region on Contig 553, which was identified as the complete H-fibroin gene. PacBio sequencing results show a coverage depth of > 100 × with many reads spanning across large proportions of the gene range, assuring the validity of the assembly. The region harbors multiple conserved tandem units, showing high similarity to a previously reported H-fibroin gene fragment from *Stenopsyche marmorata*, a retreat-making caddisfly from the same genus [7] (GenBank

accession number BAM84281, 479aa in length, Fig. 6). The conserved units code typical short repeats of the H-fibroin sequence, including GGX, SXSXSX and GPGX, with varied sequences and lengths (Fig. 6). In addition, the identified region contained the conserved non-repetitive N- and C-termini of the *S. marmorata* H-fibroin [12] (Fig. 6), further confirming the gene assembly. The resolution of a complete H-fibroin gene in our study provides a significant expansion over existing genetic resources on caddisfly silk genes, which will be important for studying H-fibroin and silk adaptation to aquatic environments. For future studies, transcriptome and gene expression analysis from larval silk glands would help to elucidate the precise structure of the H-fibroin gene.

Concluding remarks

The genome presented here is the first high quality draft genome of a retreat-building caddisfly. With a known diversity of over 16,000 species, this is a welcome addition to the genomic resources available to the caddisfly research community. Because caddisflies are important members of freshwater ecological communities and their species have been shown to be effective indicators of freshwater health, there exists a host of researchers in freshwater biology and entomology whose research will be positively impacted by the availability of a high-quality draft genome.

In addition to the genome, we present an annotation with 14,687 genes annotated. This will enable large scale comparisons with existing genomes, especially those in Lepidoptera. While Trichoptera and Lepidoptera are reciprocally monophyletic and among the strongest supported ordinal level relationships within insects, they have highly divergent life histories with Lepidoptera primarily terrestrial, while the Trichoptera egg, larval, and pupal stages are entirely aquatic. The comparisons now possible with the addition of a high quality trichopteran genome have the potential to

deliver insights into the genetic basis of these divergent adaptation strategies to land habitats.

Availability of supporting data

All raw sequencing reads have been deposited in the Short Read Archive (SRA) under the project PRJNA436868. The raw sequencing reads, genome assembly, gene models and other supporting data are available via the GigaScience database, GigaDB.

Abbreviations

BUSCO: Benchmarking Universal Single-Copy Orthologs; GO: Gene Ontology; KEGG: Kyoto Encyclopedia of Genes and Genomes; OBP: odorant binding protein; SMRT: single molecular real time; SSR: simple sequence repeats; TE: transposable elements; TRF: tandem repeat finder.

Competing interests

The authors declare that there are no competing interests.

Funding

XZ is supported by the National Science Foundation of China (31772493) , Beijing Advanced Innovation Center for Food Nutrition and Human Health, and the Chinese Universities Scientific Fund (2017QC114 and 2018QC133) through China Agricultural University.

Author contributions

XZ designed the study. SL, MT and PBF conducted genome analysis and assembly. XZ, SL, PBF and MT collected the specimens. PBF and RJS led analysis of the H-fibroin gene. All authors participated in writing and proofed the manuscript.

Acknowledgements

XZ thank Dr. Tingting Zhang from Shandong Agricultural University for her contribution in preparing the illustration of the caddisfly. Drs. Meng Yang, Ruixue Li,

Hui Zhang and Hua Peng from NextOmics provided important expertise and assistance in genome sequencing and analysis.

References

1. Morse JC. The Trichoptera world checklist. Zoosymposia 2011;**5**(1):372-80.
2. Misof B, Liu S, Meusemann K, et al. Phylogenomics resolves the timing and pattern of insect evolution. Science 2014;**346**(6210):763-7.
3. Resh VH and Unzicker JD. Water quality monitoring and aquatic organisms: the importance of species identification. J Water Pollut Control Fed 1975;**47**(1):9-19.
4. Holzenthal R, Blahnik R, Kjer K, et al. An update on the phylogeny of caddisflies (Trichoptera). In: *Proceedings of the 12th International Symposium on Trichoptera The Caddis Press, Columbus, Ohio* 2007, pp.143-53.
5. Holzenthal RW, Thomson RE and Ríos-Touma B. Order Trichoptera. Thorp and Covich's Freshwater Invertebrates (Fourth Edition). Elsevier; 2015. p. 965-1002.
6. Stewart RJ and Wang CS. Adaptation of caddisfly larval silks to aquatic habitats by phosphorylation of H-fibroin serines. Biomacromolecules 2010;**11**(4):969-74.
7. Ohkawa K, Miura Y, Nomura T, et al. Long-range periodic sequence of the cement/silk protein of *Stenopsyche marmorata*: purification and biochemical characterisation. Biofouling 2013;**29**(4):357-67.
8. Addison JB, Ashton NN, Weber WS, et al. β-Sheet nanocrystalline domains formed from phosphorylated serine-rich motifs in caddisfly larval silk: a solid state NMR and XRD study. Biomacromolecules 2013;**14**(4):1140-8.
9. Ashton NN and Stewart RJ. Self-recovering caddisfly silk: energy dissipating, Ca^{2+}-dependent, double dynamic network fibers. Soft Matter 2015;**11**(9):1667-76.
10. Ashton NN, Pan H and Stewart RJ. Connecting caddisworm silk structure and mechanical properties: combined infrared spectroscopy and mechanical analysis. Open Biol 2016;**6**(6):160067.
11. Yonemura N, Mita K, Tamura T, et al. Conservation of silk genes in Trichoptera and Lepidoptera. J Mol Evol 2009;**68**(6):641-53.
12. Wang Y, Sanai K, Wen H, et al. Characterization of unique heavy chain fibroin filaments spun underwater by the caddisfly *Stenopsyche marmorata* (Trichoptera; Stenopsychidae). Mol Biol Rep 2010;**37**(6):2885-92.
13. Ashton NN, Roe DR, Weiss RB, et al. Self-tensioning aquatic caddisfly silk: Ca^{2+}-dependent structure, strength, and load cycle hysteresis. Biomacromolecules 2013;**14**(10):3668-81.
14. Wang CS, Ashton NN, Weiss RB, et al. Peroxinectin catalyzed dityrosine crosslinking in the adhesive underwater silk of a casemaker caddisfly larvae, *Hysperophylax occidentalis*. Insect Biochem Mol Biol 2014;**54**:69-79.

15. Hwang CL. Descriptions of Chinese caddis flies (Trichoptera). Acta Zool sin 1958;**10**:279-85.
16. Xu JH, Wang BX and Sun CH. The *Stenopsyche simplex* species group from China with descriptions of three new species (Trichoptera: Stenopsychidae). Zootaxa 2014;**3785**(2):217-30.
17. Fu X, Li J, Tian Y, et al. Long-read sequence assembly of the firefly *Pyrocoelia pectoralis* genome. Gigascience 2017;**6**(12):1-7.
18. Marçais G and Kingsford C. A fast, lock-free approach for efficient parallel counting of occurrences of k-mers. Bioinformatics 2011;**27**(6):764-70.
19. Chin CS, Alexander DH, Marks P, et al. Nonhybrid, finished microbial genome assemblies from long-read SMRT sequencing data. Nat Methods 2013;**10**(6):563-9.
20. Salmela L and Rivals E. LoRDEC: accurate and efficient long read error correction. Bioinformatics 2014;**30**(24):3506-14.
21. Chin CS, Peluso P, Sedlazeck FJ, et al. Phased diploid genome assembly with single-molecule real-time sequencing. Nat Methods 2016;**13**(12):1050-4.
22. Li H and Durbin R. Fast and accurate short read alignment with Burrows–Wheeler transform. Bioinformatics 2009;**25**(14):1754-60.
23. Walker BJ, Abeel T, Shea T, et al. Pilon: an integrated tool for comprehensive microbial variant detection and genome assembly improvement. PLoS One 2014;**9**(11):e112963.
24. Kiełbasa SM, Wan R, Sato K, et al. Adaptive seeds tame genomic sequence comparison. Genome Res 2011;**21**(3):487-93.
25. i5K Consortium. The i5K Initiative: advancing arthropod genomics for knowledge, human health, agriculture, and the environment. J Hered 2013;**104**(5):595-600.
26. Ferguson L, Marlétaz F, Carter J-M, et al. Ancient expansion of the Hox cluster in Lepidoptera generated four homeobox genes implicated in extra-embryonic tissue formation. PLoS Genet 2014;**10**(10):e1004698.
27. Wu TD and Watanabe CK. GMAP: a genomic mapping and alignment program for mRNA and EST sequences. Bioinformatics 2005;**21**(9):1859-75.
28. Simão FA, Waterhouse RM, Ioannidis P, et al. BUSCO: assessing genome assembly and annotation completeness with single-copy orthologs. Bioinformatics 2015;**31**(19):3210-2.
29. Zdobnov EM, Tegenfeldt F, Kuznetsov D, et al. OrthoDB v9.1: cataloging evolutionary and functional annotations for animal, fungal, plant, archaeal, bacterial and viral orthologs. Nucleic Acids Res 2016;**45**(D1):D744-9.
30. Thiel T, Michalek W, Varshney R, et al. Exploiting EST databases for the development and characterization of gene-derived SSR-markers in barley (*Hordeum vulgare* L.). Theor Appl Genet 2003;**106**(3):411-22.
31. Xu Z and Wang H. LTR_FINDER: an efficient tool for the prediction of full-length LTR retrotransposons. Nucleic Acids Res 2007;**35**(suppl_2):W265-8.
32. Benson G. Tandem repeats finder: a program to analyze DNA sequences. Nucleic Acids Res 1999;**27**(2):573-80.

33. Tarailo-Graovac M and Chen N. Using RepeatMasker to identify repetitive elements in genomic sequences. Curr Protoc Bioinformatics 2009:4.10.1-4.
34. Kapitonov VV and Jurka J. A universal classification of eukaryotic transposable elements implemented in Repbase. Nat Rev Genet 2008;**9**(5):411-2.
35. Lagesen K, Hallin P, Rødland EA, et al. RNAmmer: consistent and rapid annotation of ribosomal RNA genes. Nucleic Acids Res 2007;**35**(9):3100-8.
36. Lowe TM and Eddy SR. tRNAscan-SE: a program for improved detection of transfer RNA genes in genomic sequence. Nucleic Acids Res 1997;**25**(5):955.
37. Burge SW, Daub J, Eberhardt R, et al. Rfam 11.0: 10 years of RNA families. Nucleic Acids Res 2012;**41**(D1):D226-D32.
38. Fu H and Dooner HK. Intraspecific violation of genetic colinearity and its implications in maize. Proc Natl Acad Sci U S A 2002;**99**(14):9573-8.
39. UniProt Consortium. UniProt: a hub for protein information. Nucleic Acids Res 2015;**43**(D1):D204-12.
40. Birney E and Durbin R. Using GeneWise in the *Drosophila* annotation experiment. Genome Res 2000;**10**(4):547-8.
41. Haas BJ, Delcher AL, Mount SM, et al. Improving the *Arabidopsis* genome annotation using maximal transcript alignment assemblies. Nucleic Acids Res 2003;**31**(19):5654-66.
42. Haas BJ, Salzberg SL, Zhu W, et al. Automated eukaryotic gene structure annotation using EVidenceModeler and the program to assemble spliced alignments. Genome Biol 2008;**9**(1):R7.
43. Duan J, Li R, Cheng D, et al. SilkDB v2. 0: a platform for silkworm (*Bombyx mori*) genome biology. Nucleic Acids Res 2009;**38**(suppl_1):D453-6.
44. Zhan S, Merlin C, Boore JL, et al. The monarch butterfly genome yields insights into long-distance migration. Cell 2011;**147**(5):1171-85.
45. Dasmahapatra KK, Walters JR, Briscoe AD, et al. Butterfly genome reveals promiscuous exchange of mimicry adaptations among species. Nature 2012;**487**(7405):94-8.
46. Davey JW, Chouteau M, Barker SL, et al. Major improvements to the *Heliconius melpomene* genome assembly used to confirm 10 chromosome fusion events in 6 million years of butterfly evolution. G3 2016;**6**(3):695-708.
47. You M, Yue Z, He W, et al. A heterozygous moth genome provides insights into herbivory and detoxification. Nat Genet 2013;**45**(2):220-5.
48. Moriya Y, Itoh M, Okuda S, et al. KAAS: an automatic genome annotation and pathway reconstruction server. Nucleic Acids Res 2007;**35**(suppl_2):W182-5.
49. Jones P, Binns D, Chang HY, et al. InterProScan 5: genome-scale protein function classification. Bioinformatics 2014;**30**(9):1236-40.
50. Ashburner M, Ball CA, Blake JA, et al. Gene ontology: tool for the unification of biology. Nat Genet 2000;**25**(1):25-9.
51. Li L, Stoeckert CJ and Roos DS. OrthoMCL: identification of ortholog groups for eukaryotic genomes. Genome Res 2003;**13**(9):2178-89.

52. Katoh K and Standley DM. MAFFT multiple sequence alignment software version 7: improvements in performance and usability. Mol Biol Evol 2013;**30**(4):772-80.
53. Talavera G and Castresana J. Improvement of phylogenies after removing divergent and ambiguously aligned blocks from protein sequence alignments. Syst Biol 2007;**56**(4):564-77.
54. Castresana J. Selection of conserved blocks from multiple alignments for their use in phylogenetic analysis. Mol Biol Evol 2000;**17**(4):540-52.
55. Stamatakis A. RAxML version 8: a tool for phylogenetic analysis and post-analysis of large phylogenies. Bioinformatics 2014;**30**(9):1312-3.
56. Yang Z. PAML 4: phylogenetic analysis by maximum likelihood. Mol Biol Evol 2007;**24**(8):1586-91.
57. De Bie T, Cristianini N, Demuth JP, et al. CAFE: a computational tool for the study of gene family evolution. Bioinformatics 2006;**22**(10):1269-71.
58. Fnu S, Williamson EA, De Haro LP, et al. Methylation of histone H3 lysine 36 enhances DNA repair by nonhomologous end-joining. Proc Natl Acad Sci U S A 2011;**108**(2):540-5.
59. Lee SH, Oshige M, Durant ST, et al. The SET domain protein Metnase mediates foreign DNA integration and links integration to nonhomologous end-joining repair. Proc Natl Acad Sci U S A 2005;**102**(50):18075-80.
60. Feyereisen R. 8 - Insect CYP Genes and P450 Enzymes A2 - Gilbert, Lawrence I. Insect Molecular Biology and Biochemistry. San Diego: Academic Press; 2012. p. 236-316.
61. Li X, Schuler MA and Berenbaum MR. Molecular mechanisms of metabolic resistance to synthetic and natural xenobiotics. Annu Rev Entomol 2007;**52**:231-53.
62. Eddy SR. Accelerated profile HMM searches. PLoS Comp Biol 2011;**7**(10):e1002195.
63. Pikielny CW, Hasan G, Rouyer F, et al. Members of a family of *Drosophila* putative odorant-binding proteins are expressed in different subsets of olfactory hairs. Neuron 1994;**12**(1):35-49.
64. McKenna MP, Hekmat-Scafe DS, Gaines P, et al. Putative *Drosophila* pheromone-binding proteins expressed in a subregion of the olfactory system. J Biol Chem 1994;**269**(23):16340-7.
65. Hekmat-Scafe DS, Scafe CR, McKinney AJ, et al. Genome-wide analysis of the odorant-binding protein gene family in *Drosophila melanogaster*. Genome Res 2002;**12**(9):1357-69.

Figure legends

Figure 1: An illustration of the adult caddisfly *Stenopsyche tienmushanensis* in its typical habitat.

Figure 2: Functional gene annotations using four databases.

Figure 3: The phylogenetic tree and gene expansion/contraction of 12 arthropod taxa. Single-copy orthologs represent orthologous genes with a single copy in all species. Multiple-copy orthologs represent the gene groups present in all species with a gene number > 1 in at least one species. Species-specific paralogs represent genes uniquely present in only one species. Other types of orthologs represent the gene groups that are absent in some species and not species-specific paralogs. Numbers of expanded gene families are marked in green, while numbers of contracted gene families are marked in red. MRCA: most recent common ancestor. The number below MRCA is the total group numbers from the OrthoMCL analysis. Note that only some of the gene expansions/contractions are significant.

Figure 4: The phylogenetic relationship of the significant expanded gene group of cytochrome P450 family in ten insect species. The phylogeny was constructed using Maximum Likelihood, showing significant expansions in *S. tienmushanensis*. The bootstrap values are marked on the nodes.

Figure 5: The Maximum Likelihood tree of odorant-binding proteins (OBPs) in five insect species. The bootstrap values are marked on the nodes. Both expanded OBP groups in *S. tienmushanensis* are closely related to those potentially responsible for pheromone detection in *Drosophila*.

Figure 6: The H-fibroin gene in *S. tienmushanensis*. The sequences of H-fibroin gene fragments previously reported from *S. marmorata* are referred from [7, 12]. Identical amino acids in alignment between *S. tienmushanensis* and *S. marmorata* were marked in grey shadow. The start and end positions of the nucleotides were shown in the alignment of the repetitive units. Amino acids in the black box represent the typical motifs of short repeat unit. S.tie: H-fibroin gene in *S. tienmushanensis*; S.mar5/S.mar3: the 5'/3' end nucleotides of H-fibroin mRNA fragments in *S. marmorata*.

Tables

Table 1 Comparison of genome assemblies among three caddisfly genomes

Species	*Stenopsyche tienmushanensis*	*Limnephilus lunatus*	*Glyphotaelius pellucidus*
Platform	PacBio + Illumina	Illumina	Illumina
Sequencing depth	153 × + 150 ×	80.1 ×	8.12 ×
Scaffold number	557	69,094	1,617
Total length	453,094,794 bp	1,369,180,260 bp	757,289,448 bp
Contig N50 length	1,296,863 bp	5,895 bp	705 bp
BUSCO (n=1,658)	C:97.6%[S:94.0%,D:3.6%],F:1.2%,M:1.2%	C:88.2%[S:82.7%,D:5.5%],F:6.9%,M:4.9%	C:25.0%[S:24.9%,D:0.1%],F:44.8%,M:30.2%

The genome of *L. lunatus* was from i5K project [25]. The genome of *G. pellucidus* was from [26].

Table 2 Summary of annotated repeats

Type	Combined TEs Length (bp)	% of genome
DNA	80,609,112	17.79
LINE	20,766,162	4.58
LTR	1,920,232	0.42
SINE	7,831	0.00
Other	15,296,436	3.38
Unknown	47,831,952	10.56
Total	166,431,725	36.73

 Click here to access/download;Figure;Figure 1.pdf

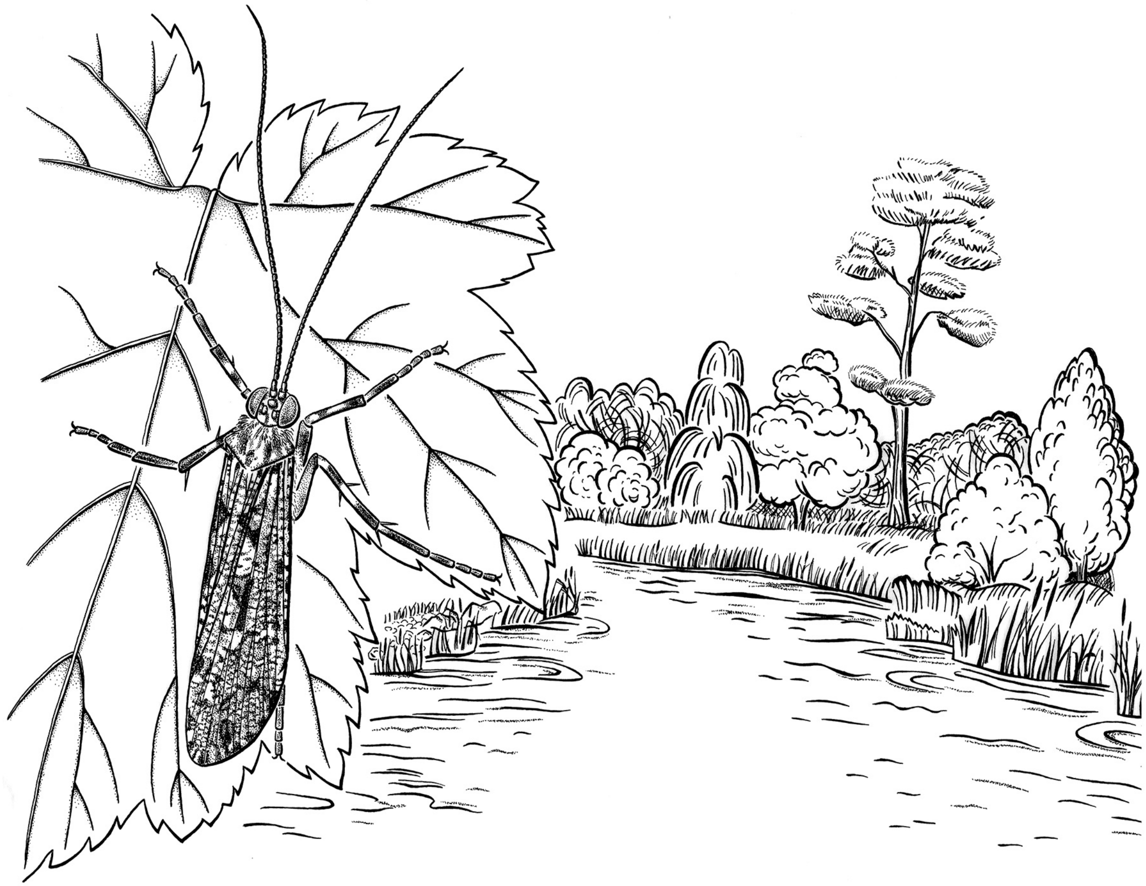

Click here to access/download;Figure;Figure 2.pdf

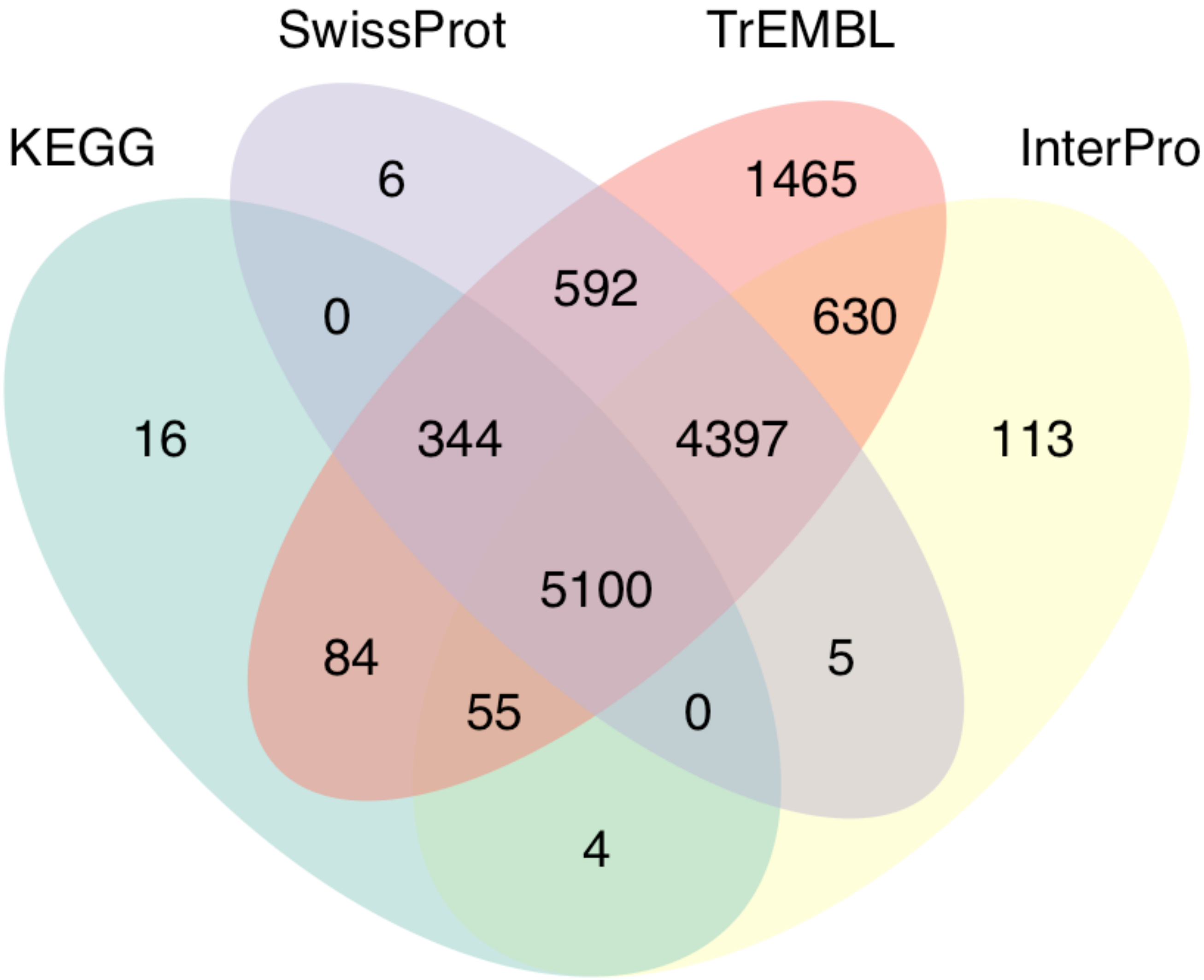

Figure

Click here to access/download;Figure;Figure 3.pdf

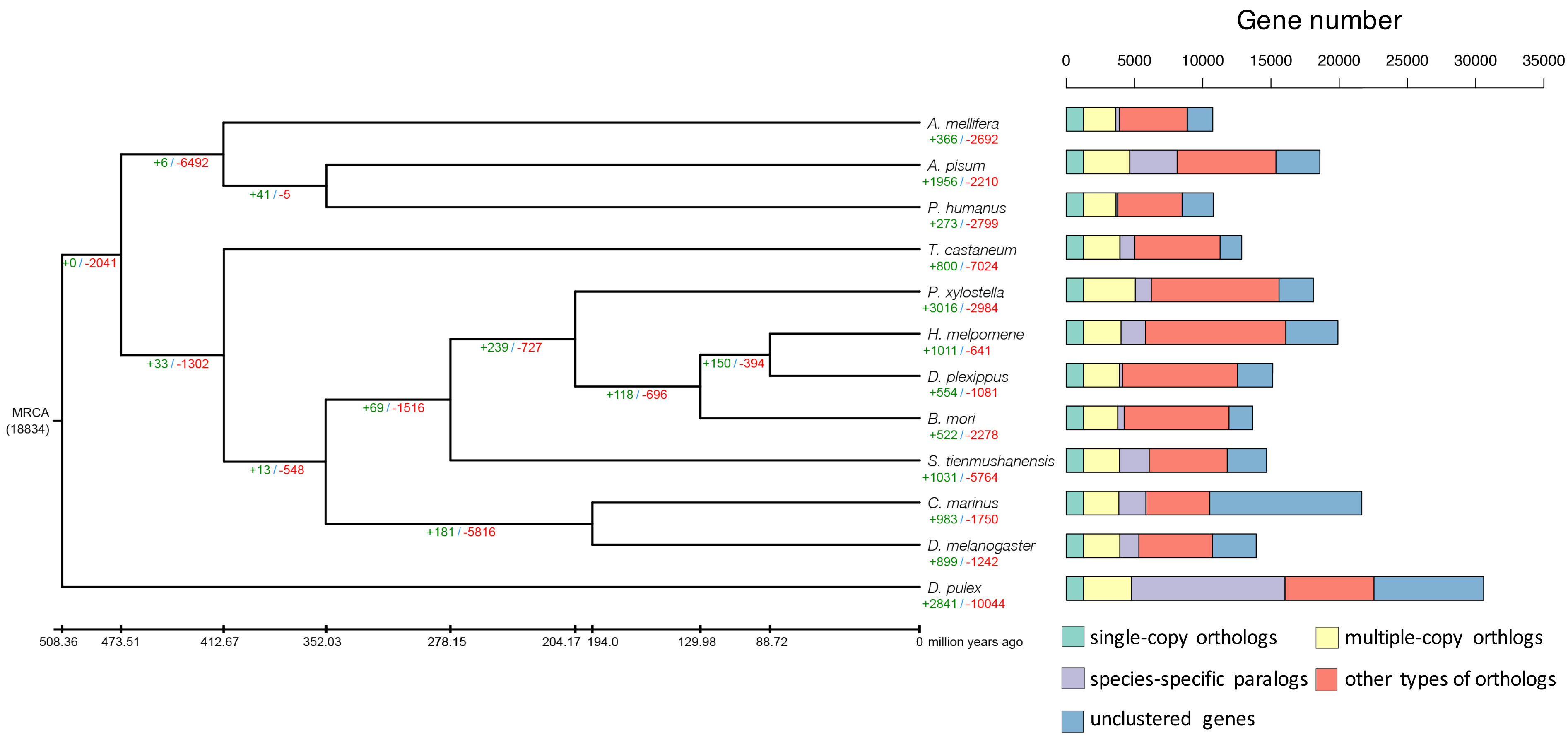

Figure
Click here to access/download;Figure;Figure 4.pdf

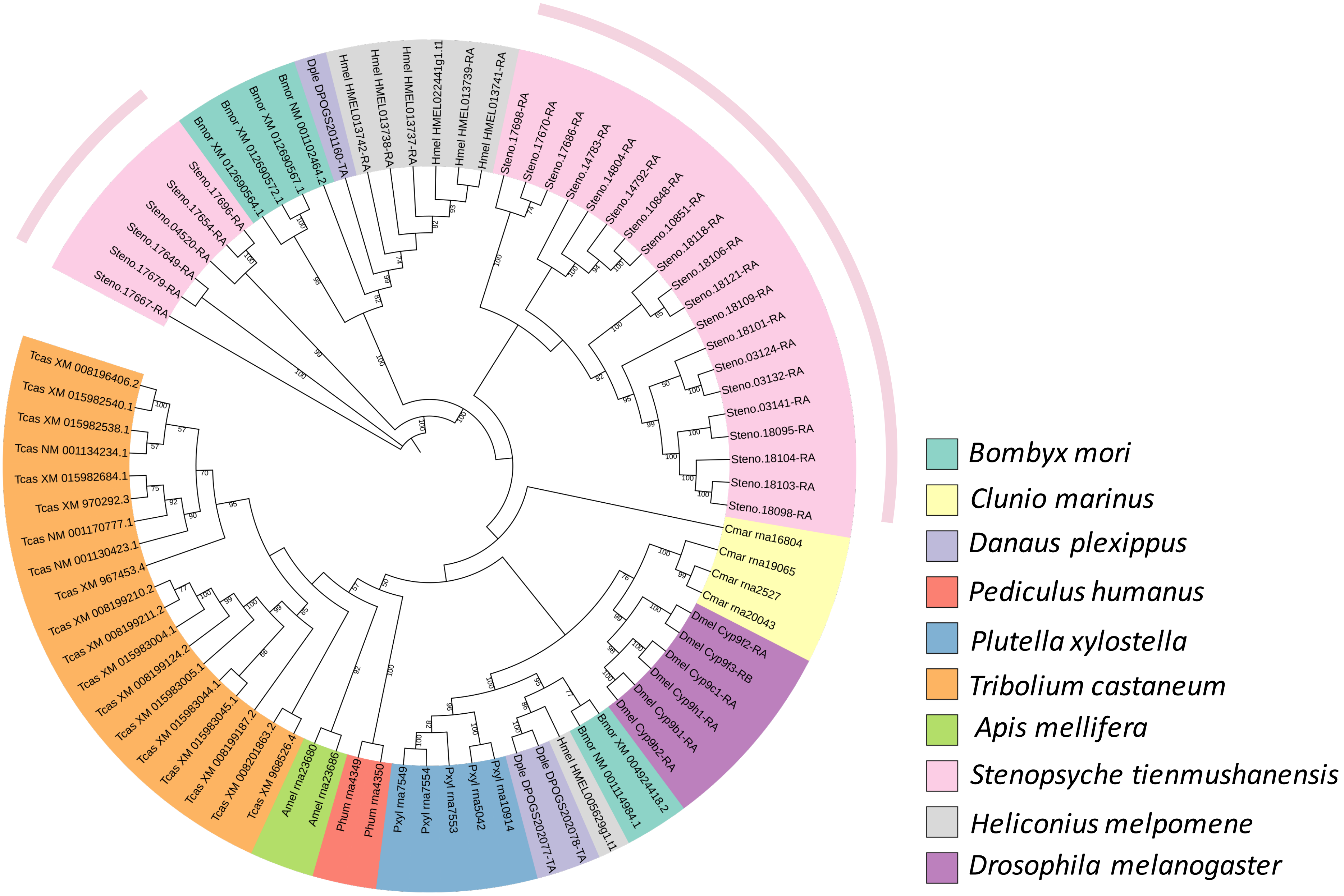

Figure

Click here to access/download;Figure;Figure 5.pdf

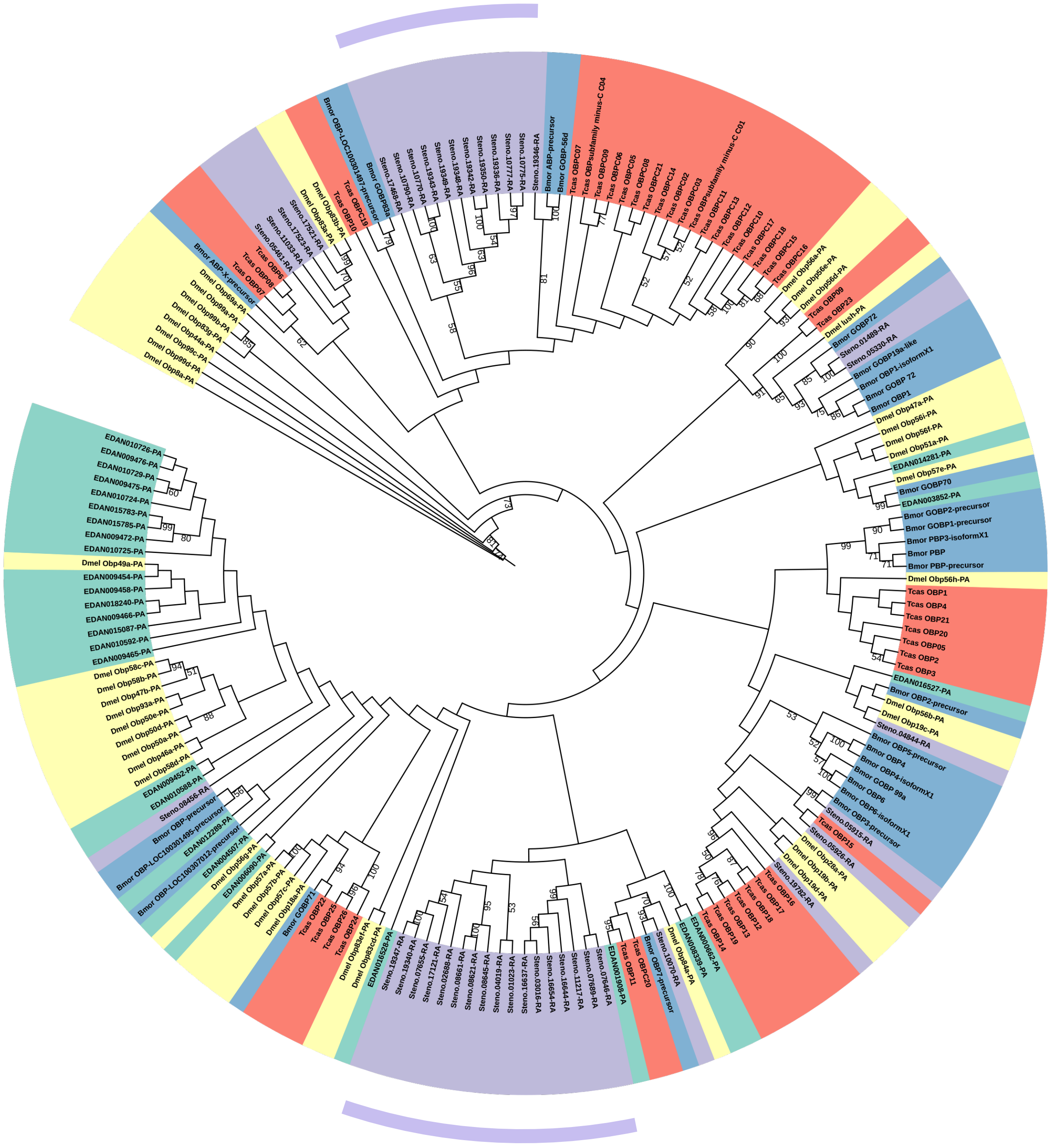

Ephemera danica

Drosophila melanogaster

Stenopsyche tienmushanensis

Tribolium castaneum

Bombyx mori

Figure

Click here to access/download;Figure;Figure 6.pdf

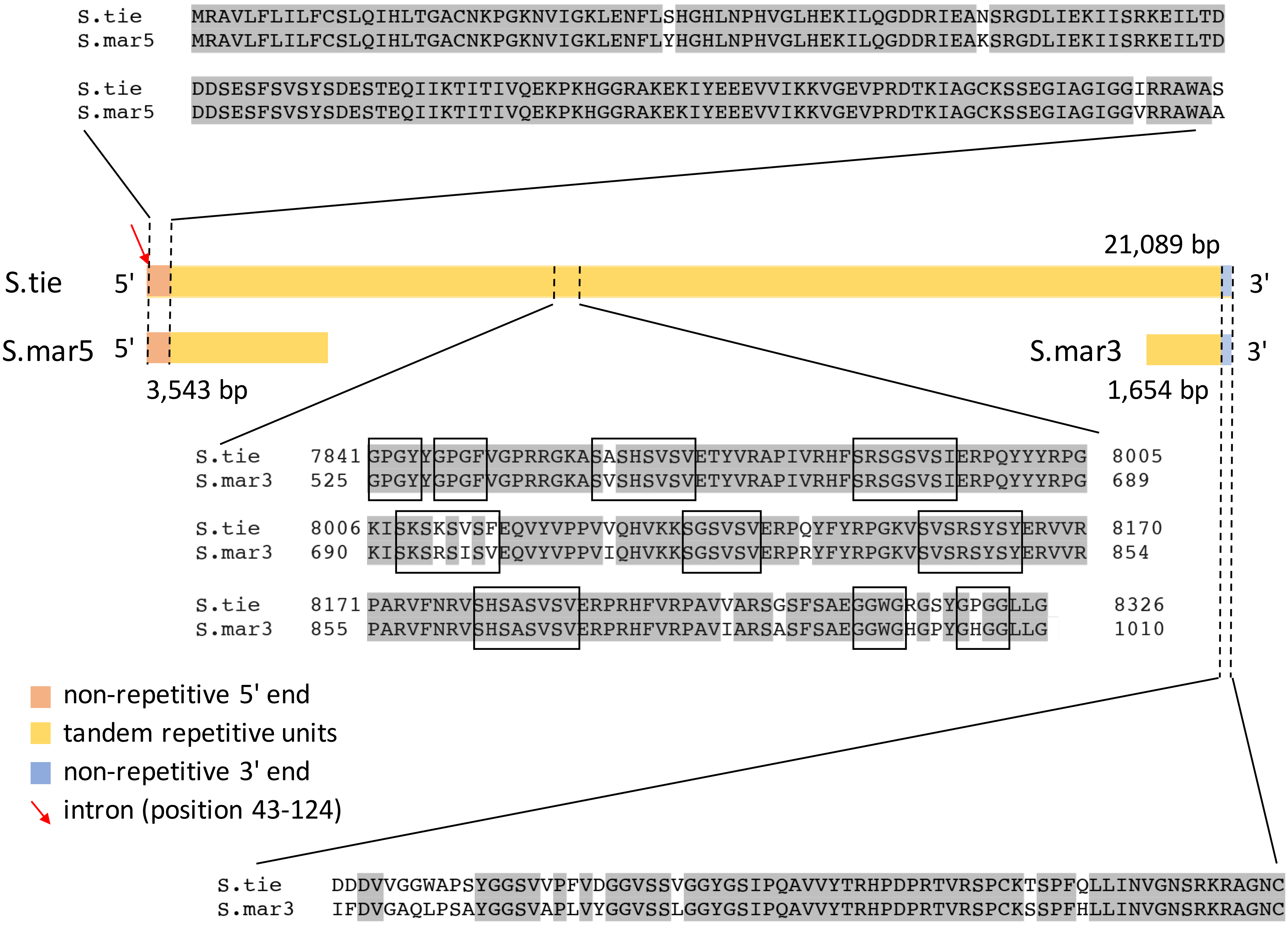

Click here to access/download;Figure;SOM_20180701.docx

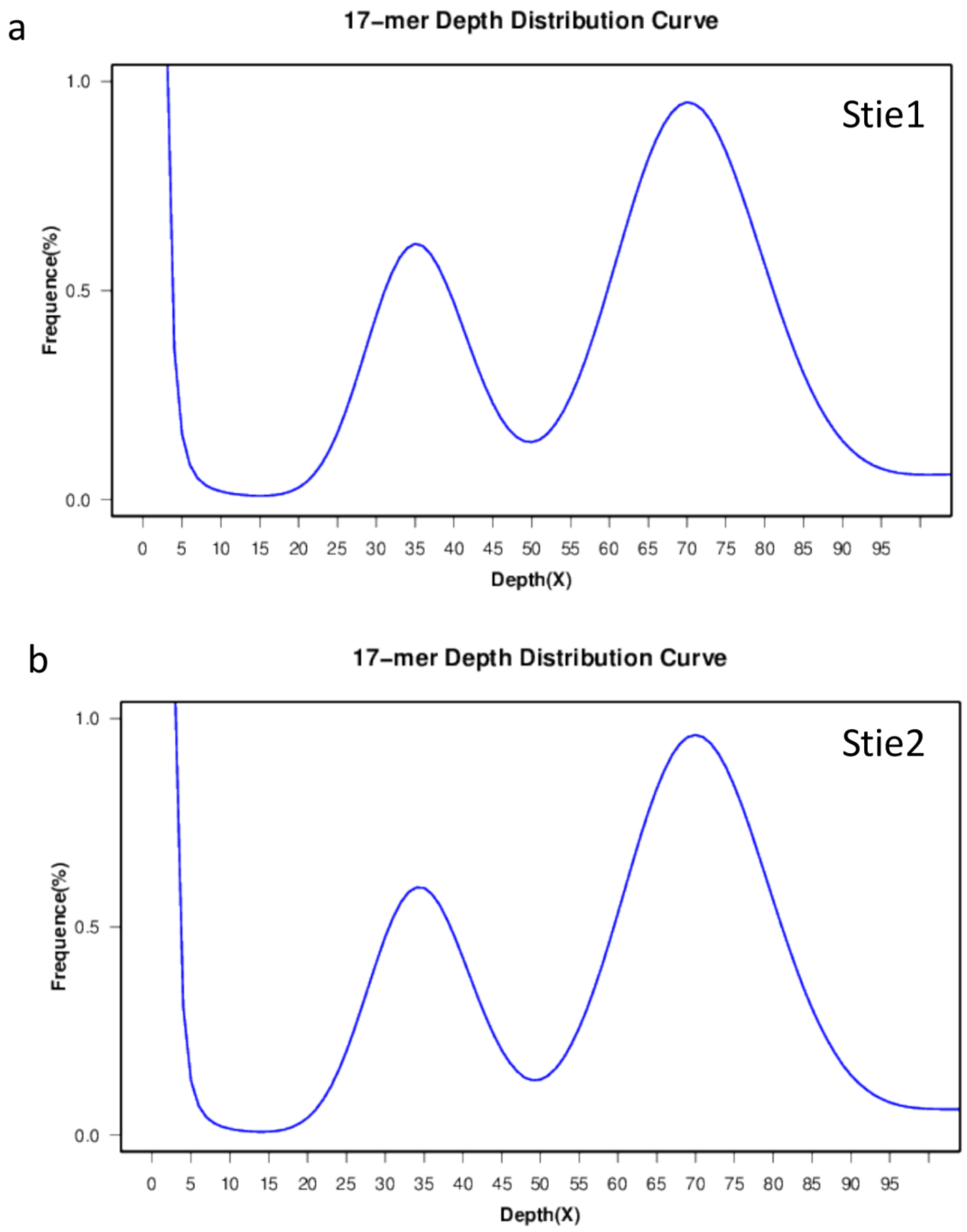

Figure S1 17-mer depth distribution curve of sample Stie1(a) and Stie2 (b) from Illumina sequencing data.

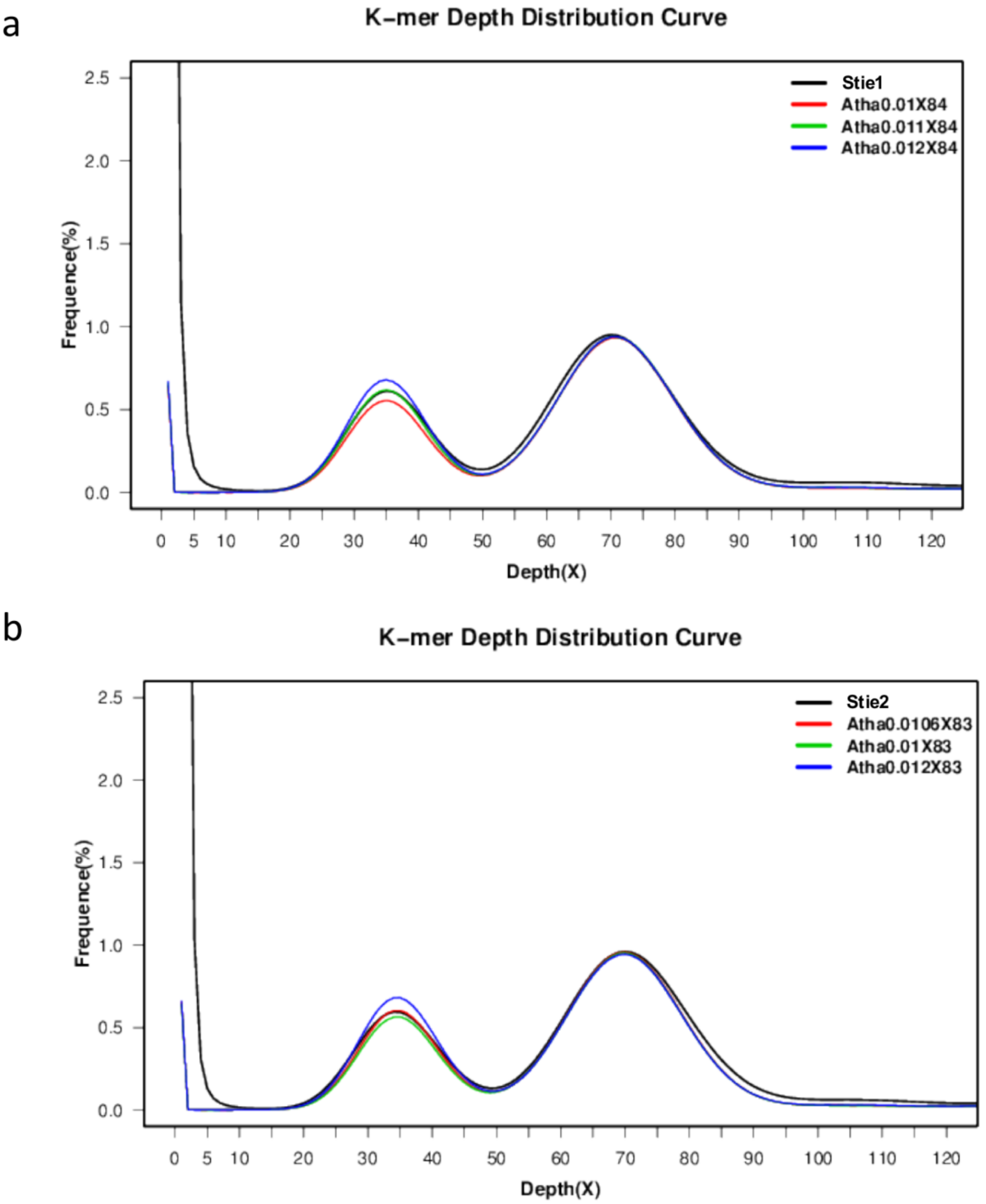

Figure S2 Comparison of K-mer depth distribution curve of sample Stie1(a) and Stie2 (b) with *Arabidopsis thaliana* genomes from Illumina sequencing data. The simulated genome data of *Arabidopsis thaliana* with different heterozygosity (number before "X" in the key) and appropriate depth (number after "X" in the key) was used.

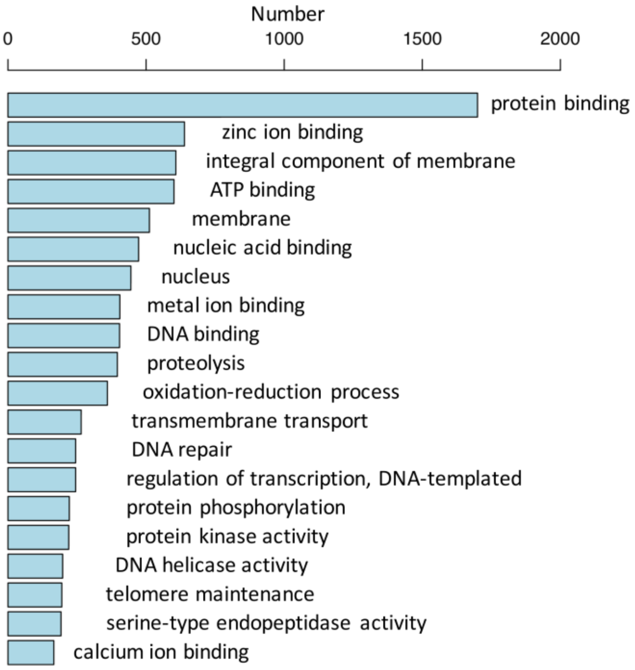

Figure S3 Top 20 terms in GO pathway analysis

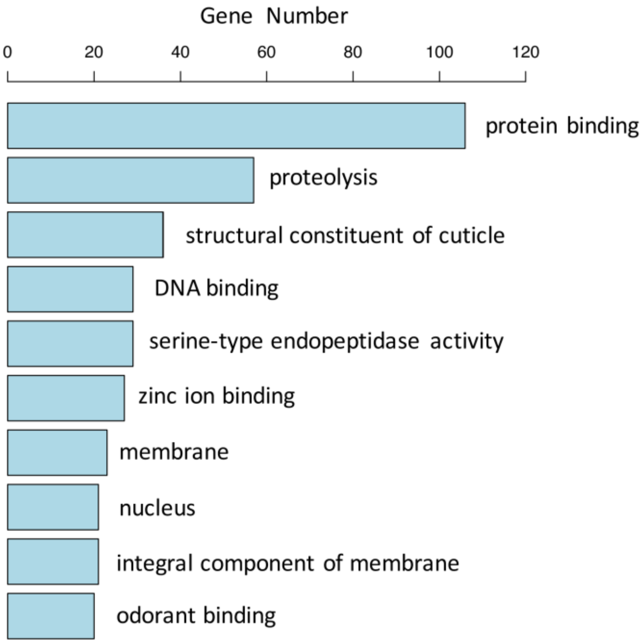

Figure S4 Top 10 terms in GO pathway analysis of the species-specific paralog genes in *Stenopsyche tienmushanensis*

Supplementary Tables

Table S1 Sequencing data counts

Sample	Source	Library type	Platform	Read length	Insert size (bp)	Total base (bp)	Number of reads
Stie1	DNA	short insert	Illumina HiSeq X ten	150 bp pair-end	400	40,679,119,800	271,194,132
Stie2	DNA	short insert	Illumina HiSeq X ten	150 bp pair-end	400	39,775,849,200	265,172,328
Stie1+Stie2	DNA	long read	PacBio Sequel	7.6 kb (mean)	20 k	78,720,333,957	10,321,897
Stie3	RNA	short insert	Illumina HiSeq X ten	150 bp pair-end	180	9,723,314,700	64,822,098
Stie3	RNA	long read	PacBio Sequel	1.1 kb (mean)	0.5-6 k	10,305,012,637	6,578,615

The Read length and Number of reads of PacBio sequencing results are from the statistics of subreads.

Table S2 Statistics of the initial and final genome assemblies

Stat Type	Initial		Final	
	Contig length	Contig number	Contig length	Contig number
N50	1,160,110	134	1,296,863	110
N60	933,486	183	1,109,078	148
N70	674,154	248	883,793	193
N80	411,166	344	655,011	254
N90	164,125	531	414,477	339
Longest	4,744,952	1	4,765,805	1
Total	510,726,241	2,054	453,094,794	557
Length ≥ 1kb	510,710,601	2005	453,094,794	557
Length ≥ 2kb	1,160,110	134	453,093,705	556
Length ≥ 5kb	933,486	183	453,089,922	555

Table S3 Summary of Simple Sequence Repeat (SSR)

Type	Number/Size
Total examined sequences	557/453,094,794
Total identified SSR	91,871
Compound format	4,232
Sequences contain SSR	556
Sequences contain SSR(>1)	554

Table S4 Numbers of different types of Simple Sequence Repeat (SSR)

Type	Unit size (repeat number)	Number
p1	1($\geq$ 10)	68,838
p2	2($\geq$ 6)	18,379
p3	3($\geq$ 5)	3,968
p4	4($\geq$ 5)	468
p5	5($\geq$ 5)	205
p6	6($\geq$ 5)	13

Type: p1: repeat of single nucleotide; p2: repeat of two-nucleotide unit, p3: repeat of three-nucleotide unit and similar in p4, p5 and p6. Unit size: the number of nucleotides in a repetitive unit of SSR.

Table S5 Annotated repeat sequences from different methods

Methods	Repeat Size(bp)	% of genome
LTR_finder	1,749,004	0.39
TRF	3,587,745	0.79
RepeatMasker + Repbase library	46,896,120	10.35
RepeatMasker + RepeatModeler *de novo* library	157,078,944	34.67
RepeatProteinMasker	30,118,277	6.65
Total	166,431,725	36.73

Table S6 Statistics of gene prediction from three methods

Method	Software	Total number of genes	Average gene length (bp)	Average CDS length (bp)	Average exon number per gene	Average exon length (bp)	Average intron number per gene	Average intron length (bp)
De novo	AUGUSTUS	20,508	8025.82	1,344.34	5.31	253.2	4.31	1,550.46
Homology	GeneWise	13,651	5,443.79	1,064.94	4.19	254.02	3.19	1,371.79
cDNA	PASA	9,499	13,776.29	1,490.47	6.87	216.95	5.87	2,092.92
Final set	EVM	14,687	11,211.85	1,509.99	6.43	234.9	4.89	1,787.26

Table S7 Comparison of gene annotations with representative lepidopterans

Species	Total number of genes	Average gene length (bp)	Average CDS length (bp)	Average exon number per gene	Average exon length (bp)	Average intron length (bp)
Stenopsyche tienmushanensis	14,687	11,211.85	1,509.99	6.43	234.90	1,787.26
Bombyx mori	13,663	14,654.48	1,429.25	6.79	210.52	2,284.46
Danaus plexippus	15,130	6,002.37	1,382.82	6.71	205.97	808.51
Heliconius melpomene	19,908	5,254.51	1,217.58	5.49	221.88	899.61
Plutella xylostella	18,106	9,657.24	1,406.53	7.07	198.87	1,358.68

The source of genome assemblies: *B. mori* : ASM15162 v.1 [1], *D. plexippus* v.3 [2], *H. melpomene* Hmel2.5 [3, 4]，*P. xylostella* DBM_FJ_V1.1 [5].

Table S8 Genome data sources of the 11 arthropod species used in evolutionary analysis

Species	Data source
Apis mellifera	ftp://ftp.ncbi.nlm.nih.gov/genomes/all/GCF/000/002/195/GCF_000002195.4_Amel_4.5
Acyrthosiphon pisum	ftp://ftp.ncbi.nlm.nih.gov/genomes/all/GCF/000/142/985/GCF_000142985.2_Acyr_2.0
Pediculus humanus	ftp://ftp.ncbi.nlm.nih.gov/genomes/all/GCF/000/006/295/GCF_000006295.1_JCVI_LOUSE_1.0
Tribolium castaneum	ftp://ftp.ncbi.nlm.nih.gov/genomes/all/GCF/000/002/335/GCF_000002335.3_Tcas5.2
Plutella xylostella	ftp://ftp.ncbi.nlm.nih.gov/genomes/all/GCF/000/330/985/GCF_000330985.1_DBM_FJ_V1.1
Heliconius melpomene	http://www.butterflygenome.org/sites/default/files/Hmel2.5_Release_2017-10-05.tar.gz
Danaus plexippus	http://monarchbase.umassmed.edu/download/Dp_geneset_OGS2*
Bombyx mori	http://download.lepbase.org/v4/sequence/Bombyx_mori_ASM15162v1*
Clunio marinus	ftp://ftp.ncbi.nlm.nih.gov/genomes/all/GCA/900/005/825/GCA_900005825.1_CLUMA_1.0/
Drosophila melanogaster	ftp://ftp.flybase.net/genomes/Drosophila_melanogaster/dmel_r6.18_FB2017_05
Daphnia pulex	ftp://ftp.ncbi.nlm.nih.gov/genomes/all/GCA/000/187/875/GCA_000187875.1_V1.0

References

1. Duan J, Li R, Cheng D, et al. SilkDB v2. 0: a platform for silkworm (*Bombyx mori*) genome biology. Nucleic Acids Res 2009;**38**(suppl_1):D453-6.
2. Zhan S, Merlin C, Boore JL, et al. The monarch butterfly genome yields insights into long-distance migration. Cell 2011;**147**(5):1171-85.
3. Dasmahapatra KK, Walters JR, Briscoe AD, et al. Butterfly genome reveals promiscuous exchange of mimicry adaptations among species. Nature 2012;**487**(7405):94-8.
4. Davey JW, Chouteau M, Barker SL, et al. Major improvements to the *Heliconius melpomene* genome assembly used to confirm 10 chromosome fusion events in 6 million years of butterfly evolution. G3 2016;**6**(3):695-708.
5. You M, Yue Z, He W, et al. A heterozygous moth genome provides insights into herbivory and detoxification. Nat Genet 2013;**45**(2):220-5.
