# Supplementary material for: The genome of an underwater architect, the caddisfly Stenopsyche tienmushanensis Hwang (Insecta: Trichoptera)
Source: Gigascience. 2018 Nov 23;7(12):giy143. doi: 10.1093/gigascience/giy143 (PMC6302954; doi:10.1093/gigascience/giy143)
Supplement: giga-d-18-00136_revision_1.pdf [file giy143_giga-d-18-00136_revision_1.pdf]

GigaScience

The genome of an underwater architect, the caddisfly Stenopsyche tienmushanensis Hwang (Insecta: Trichoptera)

--Manuscript Draft--

Manuscript Number:	GIGA-D-18-00136R1
Full Title:	The genome of an underwater architect, the caddisfly Stenopsyche tienmushanensis Hwang (Insecta: Trichoptera)
Article Type:	Data Note
Funding Information:	National Natural Science Foundation of China (31772493) — Dr. Xin Zhou<br>Chinese Universities Scientific Fund (2017QC114) — Dr. Xin Zhou<br>Chinese Universities Scientific Fund (2018QC133) — Dr. Xin Zhou
Abstract:	Background: Caddisflies (Insecta: Trichoptera) are a highly adapted freshwater group of insects split from a common ancestor with Lepidoptera. They are the most diverse (with > 16,000 species) of the strictly aquatic insect orders and widely employed as bio-indicators in water quality assessment and monitoring. Among the numerous adaptations to aquatic habitats, caddisfly larvae use their silk and materials from the environment (stones, sticks, leaf matter and etc.) to build composite structures such as fixed retreats and portable cases. Understanding how caddisflies have adapted to aquatic habitats will help explain the evolution and subsequent diversification of the group.<br><br>Findings: We sequenced a retreat-maker caddisfly Stenopsyche tienmushanensis Hwang and reported a high-quality genome assembly from both Illumina and PacBio sequencing. In total, 601.2 M Illumina reads (90.2 Gb), and 16.9 M PacBio subreads (89.0 Gb) were generated. The assembled genome is 451.5 Mb with a contig N50 of 1.29 Mb and a longest contig of 4.76 Mb, covering 97.65% of the 1,658 insect single-copy genes via BUSCO assessment. The genome comprises of 36.76% repetitive elements and 14,672 predicted protein-coding genes. The new genome sequences revealed gene expansions in specific groups of the cytochrome P450 family and olfactory binding proteins, suggesting potential genomic features associated with pollutant tolerance and mate finding. In addition, the complete gene complex of the highly repetitive H-fibroin, the major protein component of caddisfly larval silk, was assembled.<br><br>Conclusions: We reported the draft genome of Stenopsyche tienmushanensis, the highest quality caddisfly genome so far. The genome information will be an important resource for the study of caddisflies, and may shed light on the evolution of aquatic insects.
Corresponding Author:	Xin Zhou<br><br>CHINA
Corresponding Author Secondary Information:	
Corresponding Author's Institution:	
Corresponding Author's Secondary Institution:	
First Author:	Shiqi Luo
First Author Secondary Information:	
Order of Authors:	Shiqi Luo<br>Min Tang

Powered by Editorial Manager® and ProduXion Manager® from Aries Systems Corporation

	Paul B. Frandsen
	Russell J. Stewart
	Xin Zhou
**Order of Authors Secondary Information:**	
**Response to Reviewers:**	Please check the file "Response_to_reviewers_20180925.docx" uploaded as "Personal cover"
**Additional Information:**	
**Question**	**Response**
Are you submitting this manuscript to a special series or article collection?	No
**Experimental design and statistics**<br><br>Full details of the experimental design and statistical methods used should be given in the Methods section, as detailed in our Minimum Standards Reporting Checklist. Information essential to interpreting the data presented should be made available in the figure legends.<br><br>Have you included all the information requested in your manuscript?	Yes
**Resources**<br><br>A description of all resources used, including antibodies, cell lines, animals and software tools, with enough information to allow them to be uniquely identified, should be included in the Methods section. Authors are strongly encouraged to cite Research Resource Identifiers (RRIDs) for antibodies, model organisms and tools, where possible.<br><br>Have you included the information requested as detailed in our Minimum Standards Reporting Checklist?	Yes
**Availability of data and materials**<br><br>All datasets and code on which the conclusions of the paper rely must be either included in your submission or	Yes

Powered by Editorial Manager® and ProduXion Manager® from Aries Systems Corporation

deposited in publicly available repositories (where available and ethically appropriate), referencing such data using a unique identifier in the references and in the "Availability of Data and Materials" section of your manuscript.<br><br>Have you have met the above requirement as detailed in our Minimum Standards Reporting Checklist?	

Powered by Editorial Manager® and ProduXion Manager® from Aries Systems Corporation

Manuscript
Click here to access/download;Manuscript;Main_text_20180925.docx
Click here to view linked References

The genome of an underwater architect, the caddisfly *Stenopsyche tienmushanensis* Hwang (Insecta: Trichoptera)

Shiqi Luo[1], Min Tang[1], Paul B. Frandsen[2,3], Russell J. Stewart[4], and Xin Zhou[1*]

[1] Beijing Advanced Innovation Center for Food Nutrition and Human Health, College of Plant Protection, China Agricultural University, Beijing, China 100193

[2] Department of Plant and Wildlife Sciences, Brigham Young University, Provo, UT 84602 USA

[3] Data Science Lab, Smithsonian Institution, Washginton, DC, 20002 USA

[4] Department of Biomedical Engineering, University of Utah, Salt Lake City, UT 84112 USA

Shiqi Luo: shiqi_luo@cau.edu.cn, http://orcid.org/0000-0002-0506-2230

Min Tang: mintang_bio@outlook.com, http://orcid.org/0000-0002-6021-7282

Paul B. Frandsen: paul_frandsen@byu.edu, http://orcid.org/0000-0002-4801-7579

Russell J. Stewart: russell.stewart@utah.edu, https://orcid.org/0000-0002-8389-8877

Xin Zhou: xinzhoucaddis@icloud.com, http://orcid.org/0000-0002-1407-7952

*Correspondence should be addressed to XZ (xinzhoucaddis@icloud.com)

Abstract

Background: Caddisflies (Insecta: Trichoptera) are a highly adapted freshwater group of insects split from a common ancestor with Lepidoptera. They are the most diverse (with > 16,000 species) of the strictly aquatic insect orders and widely employed as bio-indicators in water quality assessment and monitoring. Among the numerous

adaptations to aquatic habitats, caddisfly larvae use their silk and materials from the environment (stones, sticks, leaf matter and etc.) to build composite structures such as fixed retreats and portable cases. Understanding how caddisflies have adapted to aquatic habitats will help explain the evolution and subsequent diversification of the group. **Findings:** We sequenced a retreat-maker caddisfly *Stenopsyche tienmushanensis* Hwang and reported a high-quality genome assembly from both Illumina and PacBio sequencing. In total, 601.2 M Illumina reads (90.2 Gb), and 16.9 M PacBio subreads (89.0 Gb) were generated. The assembled genome is 451.5 Mb with a contig N50 of 1.29 Mb and a longest contig of 4.76 Mb, covering 97.65% of the 1,658 insect single-copy genes via BUSCO assessment. The genome comprises of 36.76% repetitive elements and 14,672 predicted protein-coding genes. The new genome sequences revealed gene expansions in specific groups of the cytochrome P450 family and olfactory binding proteins, suggesting potential genomic features associated with pollutant tolerance and mate finding. In addition, the complete gene complex of the highly repetitive H-fibroin, the major protein component of caddisfly larval silk, was assembled. **Conclusions:** We reported the draft genome of *Stenopsyche tienmushanensis*, the highest quality caddisfly genome so far. The genome information will be an important resource for the study of caddisflies, and may shed light on the evolution of aquatic insects.

Keywords

caddisworm, caddisfly, aquatic insect, freshwater adaptation, silk, H-fibroin, PacBio

Data Description

Comprising >16,000 species and distributed worldwide except for Antarctica, caddisflies (Insecta: Trichoptera) are the most diverse of the strictly aquatic insect orders [1]. This highly adapted freshwater group split from a common ancestor with lepidopterans (moths and butterflies) more than 200 mya [2]. The transition between terrestrial and aquatic (freshwater) habitat has occurred multiple times independently within insects, with caddisflies representing one of the most recent examples [2]. Presumably, this radical transition required numerous adaptations in morphological, physiological and molecular traits. Understanding these adaptations will help explain how insects, in general, have evolved as one of the most successful and abundant class of animals on the planet, and how caddisflies, in particular, have adapted to a wide range of freshwater and marine habitats. Identifying the genomic underpinnings of the adaptive mechanisms of caddisflies will improve our knowledge of these thriving aquatic insects that, as major contributors to freshwater biodiversity, have been widely employed as bio-indicators in water quality assessment and monitoring [3].

In addition, caddisflies are of technological interest because, like their terrestrial moth and butterfly relatives, their larvae (caddisworms) spin silk. Unlike terrestrial silks, caddisworm silk is adapted to be spun from silk dope, the liquid form of the silk stored in silk glands, into tough viscoelastic fibers while fully submerged in water. Caddisworms use their silk as an adhesive tape to construct a wide variety of composite structures using stones, sticks, leaf matter, and other sediment gathered from the benthos of freshwater rivers, lakes, streams, and marine tidal pools [4]. The larval architectures are suborder dependent, and include transportable tube cases that provide camouflage and physical protection (suborder Integripalpia), stationary fixed retreats with silk nets for capturing food (suborder Annulipalpia), and rigid silk cases for pupation (suborder "Spicipalpia") [5]. The distinct and varied deployments of their

underwater silk are responsible, in large part, for the penetration of caddisworms into diverse aquatic habitats.

The major protein component of caddisworm silk is H-fibroin, a high molecular weight protein with a blocky, highly repetitive primary sequence. Caddisworm H-fibroins are extensively phosphorylated on repeating serine-rich motifs with the sequence $({}_{p}SX)_{n}$, where pS is phosphoserine, X is a hydrophobic amino acid, and n=2-6 [6, 7]. The $({}_{p}SX)_{n}$ motifs form divalent metal ion-stabilized β-domains that are responsible for the strength, toughness, and energy-dissipating self-recovery of caddisworm silk [8-10]. Currently, only incomplete caddisfly H-fibroin sequences are available through a GenBank search because it has not been possible to obtain full-length sequences from cDNAs [11, 12] or to assemble the highly repetitive sequence *de novo* from short-read RNA-seq data [13] in the absence of a complete caddisfly genome.

As both an underwater adhesive and a tough fully hydrated metallofiber, caddisworm silk may provide new insights into the mimetic design of tough adhesive materials for use in aquatic environments. The high-quality draft genome of a caddisfly, which includes the full assembly of the H-fibroin gene complex, will be invaluable for further identifying and characterizing the enzymes [14] and structural protein components of caddisworm silks.

Sampling, taxonomy and sample preparation

The caddisfly *Stenopsyche tienmushanensis* Hwang 1957 (Fig. 1, Fig. S1, NCBI taxonomy ID: 1560151) is only known from China, representing one of the first caddisfly species described by Chinese taxonomists [15]. The distribution range of the species was recently reviewed as confined in the Central China Region [16]. The larvae inhabit lotic environments (living in flowing waters) and are adapted to a wide range of

micro-habitats, from pristine creeks to disturbed streams, displaying tolerance to various levels of pollutants.

Adult caddisfly specimens were collected using a light trap by the Yongding River, at Yanchi Town in Beijing, China (altitude 292m, 40.03° N, 115.48° E) in 2017. This collecting site is the most northern record for the species. All specimens used in this study were collected at the same site on the same night. Specimens were kept alive on ice, flash frozen and transferred into a -80 °C freezer until extraction. Two female *Stenopsyche* adults (Stie1, Stie2) were used for genome sequencing because DNA quantity from a single specimen was not sufficient for PacBio sequencing. A third female individual (Stie3) was extracted for RNA and transcriptome sequencing. DNA and RNA were extracted from whole bodies excluding guts. DNA was extracted with SDS and proteinase K using the protocol developed by Hu *et al.* (https://www.protocols.io/view/dna-extraction-procedure-using-sds-jg4cjyw) [17]. RNA was extracted with TRIzol. Taxonomic identification was conducted by Xin Zhou using male morphology and confirmed by Cytochrome *c* Oxidase Subunit I (COI) barcodes.

Genome and transcriptome sequencing

We generated a total of ca. 270 millon 150 bp paired-end (PE) reads from two 400 bp insert-libraries for Stie1 and Stie2, respectively (80.45 Gb in total for the two samples, details shown in Table S1), using Illumina's HiSeq X Ten sequencing platform at WuXi AppTec (Shanghai, China). We combined and sequenced the remaining DNA from Stie1 and Stie2 using PacBio Sequel SMRT cells 1M v2 (PacBio p/n101-008-000), with one movie of 600 minutes at the Genome Center of Nextomics (Wuhan, China). We produced 78.72 Gb of subreads resulting in a mean subread length of 7.6 Kb (Table S1). We sequenced RNA samples using the Illumina HiSeq X Ten platform (insert-size of

180 bp, 150PE) and the PacBio Sequel system (Iso-Seq, library size 0.5-6k), which produced 9.72 Gb and 10.31 Gb data, respectively (Table S1). We used the PacBio RNA sequences to obtain full-length transcriptomes and the Illumina RNA sequences to polish/correct sequencing errors for the PacBio reads (see "Transcriptome analysis" section).

For the Illumina data, we filtered the raw data with fastp (v 0.18.0) [18] and trimmed the three low-quality bases at 5' end and two low-quality bases at 3' end of Illumina DNA reads. For the PacBio data, we filtered the subreads based on the Signal Noise Ratio (SNR) with default parameters.

Genome assembly and polishing

Before genome assembly, we estimated the genome size with the Illumina DNA data based on *k*-mer analyses. To estimate the genome size, we used the following formula: $G=K_{num}/K_{depth}$ [19], where K_{num} is the total counts of *k*-mer and K_{depth} is the *k*-mer depth. We generated a *k*-mer profile with Jellyfish (v2.1.3, RRID:SCR_005491) [20], which calculates the *k*-mer number and distribution. Then we used two different models in the genome size estimation. The first method assumes a Poisson distribution for the *k*-mers. When multiple peaks are observed, the one with lower *k*-mer frequencies is considered as a result of heterozygosity. The second method, which is integrated in the program GenomeScope, uses a mixed negative binomial model, granting more flexibility in genome size estimation [21]. Using the distribution frequency of 17-mers (Fig. S2), the genome sizes were estimated as 453.2 Mb and 445.5 Mb for Stie1 and Stie2, respectively, when K_{depth} was calculated based on a Poisson distribution; while the genome sizes estimated in GenomeScope were 407.6 Mb and 406.8 Mb, respectively (Fig. S3).

We conducted *de novo* genome assembly with Falcon on the PacBio data (v1.8.7, length_cutoff = 8 kb, length_cutoff_pr = 10 kb, max_diff = 60, max_cov = 75) [22], producing an initial assembly of 510.7 Mb, with a contig N50 of 1.16 Mb (Table S2). After the *de novo* assembly, we conducted a first round of genome polishing using PacBio subreads to improve the accuracy of the assembly. We used BLASR in SMRTlink 4.0 (https://github.com/PacificBiosciences/SMRT-Link) to map all subreads to the initial assembly, then used Arrow (part of the SMRT analysis suite) [23] to define consensus sequences. As an improved consensus model from the legacy Quiver algorithm, Arrow is based on a more straightforward hidden Markov model approach. This procedure corrected 2,556,035 insertions, 519,440 deletions and 1,302,397 substitutions.

To further correct errors in the PacBio only assembly, we polished the genome for two additional rounds with Illumina data using Pilon (v1.20, RRID:SCR_014731) [24]. First, we mapped reads from each individual, separately, to the Arrow-corrected assembly with bwa-mem using default parameters (Version 0.7.12-r1039, RRID:SCR_010910 [25]). Then we used the output bam file from the individual with higher coverage (Stie2, 99.20%, compared to 97.07% in Stie1) for the first round of polishing with Pilon, which corrected 87,535 insertions, 44,308 deletions and 46,678 substitutions. For the second round of polishing, we mapped all Illumina reads (from both Stie1 and Stie2) to the Pilon and Arrow corrected assembly using bwa-mem. We then ran the resulting bam file through Pilon again, producing an assembly of 512.7 Mb and correcting an additional 71,259 insertions, 123,506 deletions and 223,395 substitutions.

Transcriptome analysis

We identified full-length transcriptomes from the PacBio RNA-sequencing data following the PacBio IsoSeq analysis pipeline, which includes three steps: classifying, clustering and mapping. After filtering the low-quality reads, we used SMRT Link to convert raw sequences into a BAM file, producing circular consensus sequences (CCS), which were then classified into two classes: full-length reads (those that contained both the 5' primer and 3' primer with poly-As--76.43% of all subreads) and non-full-length reads. Next, we conducted isoform clustering with full-length and non-full length reads using the Iteratively Clustering and Error Correction (ICE) algorithm in the SMRT analysis software, followed by polishing using Arrow [23]. To correct sequencing errors from PacBio, we polished the consensus sequences using LoRDEC [26] with Illumina transcriptome sequences, resulting in 272,511,198 bp of 118,776 full-length transcripts. Further, we retained only the transcripts that could be aligned to the intermediate genome assembly with GMAP (-n 1) [27], then collapsed them with the python script from SMRT Link package, producing a final set with 22,347 non-redundant transcriptome isoforms. The mean length of all resulting transcripts was 2,881 bp, ranging from 274 to 13,820 bp.

Heterozygosity estimation

We estimated the heterozygosity from the *k*-mer profile by comparisons to a series of simulated heterozygosities of a model genome (*Arabidopsis thaliana*) [28]. The estimated heterozygosities were 1.10% and 1.06% for Stie1 and Stie2, respectively (Fig. S4), which were similar to the results concluded from GenomeScope (1.08% and 1.05%, respectively, Fig. S3).

Although the estimated heterozygosity of *S. tienmushanensis* is within the normal range for non-model insects with a published genome, the pooling of DNA from two wild-caught caddisfly adults represents a potential source for inflated heterozygosity.

To address this potential issue, we used LAST (v852, RRID:SCR_006119) [29] and Redundans (v 0.14a) [30] to identify redundant contigs in the intermediate assembly. Contigs of the corrected intermediate assembly were aligned against themselves using LAST (v852, RRID:SCR_006119) [29] and Redundans (v 0.14a) [30]. Those contigs with ≥ 50% of their length overlapping with others at a ≥ 80% identity were considered redundant and the shorter of the pair was removed from the genome assembly. As a result, a total of 1,472 and 1,474 contigs were identified as redundant by LAST and Redundans, respectively, with 1,471 contigs shared between the two programs. The distribution of identity of the redundant contigs identified by LAST (Fig. S5) indicated that most had > 90% similarity and overlaps with other contigs. We then compared candidate redundant contigs identified by either LAST or Redundans with the full-length transcriptomes. If a particular candidate was mapped with distinct full-length transcriptome sequences and aligned with other contigs at ≤ 90% identity, it was considered a true contig with expressed transcripts and added back into the assembly. Also we removed the short contigs (< 1,000 bp) from the genome assembly. In total, 1,498 redundant contigs were removed from the genome assembly in this step.

We screened for potential contamination in the genome assembly with Taxon-Annotated GC-Coverage (TAGC) plots using Blobtools (v1.0) [31]. To identify contaminated contigs, we followed the process outlined in Fu et al. 2017 [17]. In short, we marked a contig as a contaminant if it had all three of the following characteristics: (1) had a best hit to a reference sequence from non-Arthropoda, (2) had no mapping of full-length transcripts, and (3) contained no homologous insect genes from the Benchmarking Universal Single-Copy Orthologs (BUSCO v3.0, RRID:SCR_015008) [32]. Four contigs met these characteristics, and were subsequently removed from the assembly (TAGC plots for the final assembly shown in Fig. S6, Table S3).

The final genome assembly of *S. tienmushanensis* is 451.5 Mb, with a contig N50 of 1.29 Mb and a longest contig of 4.76 Mb (Statistics of the genome assembly in Table S2). The size of the final assembly is very close to those estimated based on *k*-mer distributions (453.2 Mb and 445.5 Mb for Stie1 and Stie2, respectively), but larger than those by GenomeScope (407.6 Mb and 406.8 Mb). This discrepancy may reflect the differences in the two algorithms, or it may imply possible redundant contigs that cannot be identified by our filtering procedures. The comparisons among the five available Trichoptera genome assemblies (including: *Glossosoma conforme* [33], *Glyphotaelius pellucidus* [34], *Limnephilus lunatus* provided by i5K [35] and *Sericostoma* sp. HW-2014 [36]) are shown in Table 1.

The completeness of the assembly was assessed using Benchmarking Universal Single-Copy Orthologs (BUSCO v3.0, RRID:SCR_015008) [32] and the insecta_odb9 gene set [37]. Overall, 97.65% of 1,658 single-copy genes were completely recovered in the full genome assembly, representing a significant improvement over existing caddisfly genomes (Table 1). The high completeness of the assembly is likely due to deep long-read sequencing, which enables the assembly of long and complex regions of the genome.

Repeat analysis and non-coding RNA (ncRNA) annotation

In total, we identified 91,564 simple sequence repeats (SSR, 4,217 with compound format) with the MIcroSAtellite identification tool (MISA, v1.0, RRID:SCR_010765) [38] using default parameters (see Table S4 for types of SSR). We identified 1,749,004 bp (0.39% of the genome size) of sequence as full-length long terminal repeat (LTR) transposons, using LTR_finder (v1.06, RRID: SCR_015247) [39]. We also identified 3,579,704 tandem repeats, accounting for 0.79% of the genome size, using Tandem Repeats Finder (TRF, v4.09) [40]. Next, we used RepeatModeler (v1.0.4,

RRID:SCR_015027, http://www.repeatmasker.org) to generate a *de novo* repeat library from the genome (searching engine: rmblast, using default parameters), followed by RepeatMasker (v4.0.7, RRID:SCR_012954) [41] to search for TEs from the known Repbase TE library (Repbase21.08) [42] and the *de novo* repeat library we built. In total, we annotated 46,773,887 bp (10.36%) and 156,642,282 bp (34.69%) from RepeatMasker with the Repbase TE library and the *de novo* repeat library, respectively. We also annotated 30,030,332 bp (6.65%) of TE sequences in the genome by similarity using the TE protein reference libraries in RepeatProteinMasker (v4.0.7, $P < 0.0001$, RRID:SCR_012954) [41]. Overall, 36.76% of the genome were masked as repeats (Table 2, results from different softwares in Table S5), with those classified as DNA transposons as the most abundant type (17.81% of the genome size).

We annotated rRNA using RNAmmer (v1.2) [43]. In addition, we aligned our RNA-seq data to all caddisfly rRNA sequences available in Genbank using BLASTN (identity > 90%, mapping length for 18s and 28s rRNA > 400 bp). We predicted tRNA using tRNAscan-SE (v1.3.1, with default parameters) [44] and annotated snRNA and miRNA using Rfam 11.0 [45] and BLAST with default parameters. In total, we predicted 150 rRNAs (four 28S rRNA genes, one 18s rRNA gene and 145 5S rRNA genes), 644 tRNAs, 75 snRNAs and 89 miRNAs.

Gene prediction

We predicted gene models using three different strategies: *ab initio*, homology-based, and RNA-seq-assisted predictions. We chose one thousand non-redundant full-length transcripts, each of which contained more than one exon with translated amino acids at < 80% identity from each other, for parameter training in the *ab initio* prediction (AUGUSTUS v3.2.2, RRID:SCR_008417, default parameters) [46]. For homology-based gene prediction, we aligned the genome to insect proteins obtained from the

uniref90 database [47] using TBLASTN with an E-value cutoff of 1e-5, and defined gene structures using GeneWise (v2.4.1, RRID:SCR 015054) [48]. For RNA-seq-assisted predictions, we used Program to Assemble Spliced Alignment (PASA) (v2.0.2, RRID:SCR_014656) [49] to align the transcriptomes to genome sequences with BLAT. We then predicted ORFs from the resulting PASA gff file using Transdecoder (v5.0.2) [50]. Finally, we used EvidenceModeler (EVM, v1.1.1, RRID:SCR_014659) to combine gene models from all three different methods, followed by PASA to update the final results, including alternative splicing, UTRs, and additional genes missed but predicted by PASA (details in Table S6) [51]. All predicted genes were aligned with known transposons by Transposon PSI (http://transposonpsi.sourceforge.net/) to remove putative transposon sequences (E-value ≤ 1e-5). In total, 14,672 genes were annotated for *S. tienmushanensis*. Comparisons of the new Trichoptera annotation with four sequenced lepidopterans (*B. mori* : ASM15162 v.1 [52], *D. plexippus* v.3 [53], *H. melpomene* Hmel2.5 [54, 55] , *P. xylostella* DBM_FJ_V1.1 [56]) suggested that gene numbers and exon lengths were similar among the species (Table S7).

Functional annotation of protein-coding genes

Gene functions were assigned based on best match of the predicted proteins to SwissProt and TrEMBL [47] using BLASTP (with E-value ≤ 1e-5), and Kyoto Encyclopedia of Genes and Genomes (KEGG) databases using KAAS [57]. In all of the 15,658 annotated proteins (including those from alternative splicing) encoded by 14,672 genes, 10,441 (66.68%), 12,661 (80.86%) and 5,602 (35.78%) had significant hits with proteins catalogued in SwissProt, TrEMBL and KEGG, respectively. In total, 10,302 (65.79%) annotated proteins included motifs/domains identified by InterProScan (v5.21, RRID:SCR 005829) [58] when searched against InterPro databases. Of these, 7,842 genes were assigned to Gene Ontology (GO) [59] IDs with

a corresponding InterPro entry (top 20 terms of GO pathway analysis shown in Fig. S7). In summary, 12,805 annotated proteins encoded by 11,838 genes were assigned with at least one related function, accounting for 80.68% of the total identified genes in *S. tienmushanensis* (Fig. 2).

Gene orthology analysis and phylogenetic tree reconstruction

We constructed a phylogeny using genome-scale orthologous genes from 12 species, including *S. tienmushanensis*, 10 additional insects (*Acyrthosiphon pisum*, *Apis mellifera*, *Bombyx mori*, *Clunio marinus*, *Danaus plexippus*, *Drosophila melanogaster*, *Heliconius melpomene*, *Tribolium castaneum*, *Pediculus humanus* and *Plutella xylostella*) and a crustacean (*Daphnia pulex*) as the outgroup (see Table S8 for additional details). Gene orthology was identified using OrthoMCL (version v2.0.9, RRID: SCR_007839) [60] with default parameters. We excluded transcripts from alternative splicing and retained only the longest transcript for each gene. Orthologous proteins from the 12 species were aligned against each other using BLASTP (E-value ≤ 1e-5). Then we used the Markov Clustering Algorithm (MCL) to perform a graph clustering of protein orthologs from above. In total, 18,834 gene family clusters were identified, including 1,263 single-copy orthologous genes (Fig. 3).

We used these 1,263 orthologous single-copy genes from the 12 species to construct a phylogenetic tree. Multiple sequence alignments were conducted with MAFFT (version 7.058beta, RRID: SCR_011811) [61], and the protein alignment was transformed to a coding sequence (CDS) alignment. We used Gblocks (version 0.91b, with parameters -b5=h) [62, 63] to filter out poorly aligned positions. The phylogenetic tree was constructed using RaxML (version v8.0.19, RRID: SCR_006086) [64] with the GTRGAMMA model and 100 bootstrap replicates. The divergence times among different lineages were estimated with the MCMCTREE package from PAML (version

4.6, RRID: SCR_014932) [65], using parameters "clock = 2, RootAge ≤ 5.30, model = 7, BDparas = 110, kappa_gamma = 62, alpha_gamma = 11, rgene_gamma = 13.7, sigma2_gamma = 11.03". The phylogenetic tree (Fig. 3) confirmed that *S. tiemushanesis* was the sister lineage to Lepidoptera. The divergence time between *S. tienmushanensis* and the three representative Lepidoptera species was generally consistent with earlier results [2].

Based on the phylogeny, we conducted analyses on gene family expansions and contractions using CAFE (version 3.1) [66] with default parameters. Compared with sister taxa from Lepidoptera, *S. tienmushanensis* possessed a larger number of contracted gene families and lower number of expanded gene families from the common ancestor (Fig. 3). Among all expanded/contracted groups, 66 gene families showed a significant change in size in *S. tienmushanensis* ($P < 0.05$), in which 63 gene families were significantly expanded. These included cytochrome P450, HSP20, insect cuticle protein, and Histone-lysine N-methyltransferase SETMAR, which is related to DNA double-strand break repair [67, 68]. The expanded cytochrome P450 in the caddisfly was most closely related to the CYP9 family from *D. melanogaster* (Fig. 4), which are functional in the metabolism of insect hormones and in the breakdown of insecticides [69, 70]. We speculate that this expansion may play a role in the adaptation of *S. tienmushanensis* to a wide range of freshwaters with varied pollutants, although further investigations are needed to prove this hypothesis.

For the species-specific paralogs of *S. tienmushanensis* revealed by the OrthoMCL analysis, GO enrichment (Fig. S8) revealed gene expansions of the odorant binding proteins (OBPs). A phylogeny of the OBPs from *S. tienmushanensis*, *D. melanogaster* [71], *T. castaneum* [72], and *B. mori* [73] (genome data sources shown in Table S8) indicated potential functional relevance of these expansions in the caddisfly genome.

Of the expanded OBP gene groups in *S. tienmushanensis*, one was most closely related to OBP83a and OBP83b from *D. melanogaster* (Fig. 5), which are also known as OS-F and OS-E with putative roles in detection of volatile pheromones [71, 74, 75]; and another was most closely related to OBP84a from *D. melanogaster*, which is also known as PBPRP-4 (pheromone-binding protein related protein gene) [71]. These uniquely expanded OBPs in *S. tienmushanensis* may be an adaptive genomic feature associated with sex attraction. Because most adult caddisflies do not feed due to reduced mouthpart structures, they are obliged to complete reproduction in a more efficient way, in the relatively short adult-stage. Therefore, the OBP expansions in *S. tienmushanensis* may reflect their adaptation in effective mate finding. It is worth noting that OBP expansion is probably not the only mechanism that helps to facilitate reproduction. We examined the mayfly (*Ephemera danica*) genome and did not find convergence on the OBPs. The PBP_GOBP family (PF01395 in Pfam), including pheromone binding proteins (PBP) and general odorant binding proteins (GOBP), was used to search for OBPs in the mayfly genome obtained from the i5K project [35] using HMMER (v3.1b2, RRID: SCR_005305) [76]. Although the mayflies are also known to have short life-span as adults, they may effectively increase their chances in finding mates by forming mating swarms. This behavioral adaptation may explain the discrepancy observed in the genomic features of their OBP genes.

H-fibroin gene analysis

Previous research on caddisworm silk has revealed that extensive phosphorylation of serines in the H-fibroin protein and the incorporation of multivalent metal ions is responsible for its unique mechanical properties in a freshwater environment [6, 10]. However, while these features have been revealed as important functional features of caddisworm silk, the genetic underpinnings of silk production have not been fully

explored. For example, only partial sequences of the H-fibroin gene have been assembled in previously sequenced transcriptomes [13], presumably due to the inadequacy of short read technologies in resolving complex genomic features rich in repeats. Here, using long read PacBio sequencing, we report the first full assembly of the H-fibroin gene complex of retreat-making caddisworms.

The genome assembly included a 21 kb region, which was identified as the complete H-fibroin gene complex, which includes two similarly sized genes with a short intergenic region from the homology-based prediction. PacBio sequencing results show a coverage depth of > 100x with many reads spanning across large proportions of the gene range, including the intergenic region, assuring the validity of the assembly (Fig. 6a). The coding regions harbor multiple conserved tandem units with high similarity to a previously reported H-fibroin gene fragment from *Stenopsyche marmorata*, a retreat-making caddisfly from the same genus [7] (GenBank accession number BAM84281, 479 aa in length). The conserved units code typical short H-fibroin repeats, including GGX, SXSXSX and GPGX, with varied sequences and lengths (Fig. 6b). In addition, the identified region contained both non-repetitive N- and C-termini homologous to the termini of *S. marmorata* H-fibroin [12] (Fig. 6c, 6d), further confirming complete assembly of the gene complex. Interestingly, the N-terminus was found at the beginning of the first gene and the C-terminus was found at the end of the second gene with the intergenic region occurring in the middle of the repetitive region (Fig. 6a). Both genes encode proteins with the expected M_m of H-fibroin, ~350 kg mol^{-1}. This structure had not been previously reported, but was only possible to elucidate with the full assembly using long-read sequencing. The assembly of the complete H-fibroin region in our study provides a significant expansion over existing genetic resources on caddisfly H-fibroin genes, which will be important for studying

caddisworm silk structure and adaptation to aquatic environments. For future studies, transcriptome and gene expression analysis from larval silk glands will help elucidate additional structural details of H-fibroin.

Concluding remarks

The genome presented here is the first high quality draft genome of a retreat-building caddisfly. With a known diversity of over 16,000 species, caddisflies are important members of freshwater ecological communities and their species have been shown to be effective indicators of freshwater health [33, 77, 78]. There exists a host of researchers in freshwater biology and entomology whose research will be positively impacted by the availability of a high-quality draft genome.

In addition to the genome, we present a set of 14,672 annotated genes. This will enable large scale comparisons with existing genomes, especially those in Lepidoptera. While Trichoptera and Lepidoptera are reciprocally monophyletic and among the strongest supported ordinal level relationships within insects [2, 79], they have highly divergent life histories with Lepidoptera being primarily terrestrial, while the Trichoptera egg, larval, and pupal stages are entirely aquatic. The addition of a high-quality trichopteran genome has the potential to deliver insights into the genetic basis of diverse strategies of insects to adapt to divergent habitats and to uncover the genomic differences between aquatic and terrestrial lifestyles. In particular, the caddisfly genome may help to gain deeper understanding of the evolution of the fascinating case-making behaviors and the robust aquatic silk of these underwater architects.

Availability of supporting data

All raw sequencing reads have been deposited in the Short Read Archive (SRA) under the project PRJNA436868. The raw sequencing reads, genome assembly, gene models and other supporting data are available via the GigaScience database, GigaDB.

Abbreviations

BUSCO: Benchmarking Universal Single-Copy Orthologs; GO: Gene Ontology; KEGG: Kyoto Encyclopedia of Genes and Genomes; OBP: odorant binding protein; SMRT: single molecular real time; SSR: simple sequence repeats; TE: transposable elements; TRF: tandem repeat finder.

Competing interests

The authors declare that there are no competing interests.

Funding

XZ is supported by the National Science Foundation of China (31772493) , Beijing Advanced Innovation Center for Food Nutrition and Human Health, and the Chinese Universities Scientific Fund (2017QC114 and 2018QC133) through China Agricultural University.

Author contributions

XZ designed the study. SL, MT and PBF conducted genome analysis and assembly. XZ, SL, PBF and MT collected the specimens. PBF and RJS led analysis of the H-fibroin genes. All authors participated in writing and proofed the manuscript.

Acknowledgements

XZ thank Dr. Tingting Zhang from Shandong Agricultural University for her contribution in preparing the illustration of the caddisfly. Drs. Meng Yang, Ruixue Li, Hui Zhang and Hua Peng from NextOmics provided important expertise and assistance in genome sequencing and analysis.

References

1. Morse JC. The Trichoptera world checklist. Zoosymposia 2011;**5**(1):372-80.
2. Misof B, Liu S, Meusemann K, et al. Phylogenomics resolves the timing and pattern of insect evolution. Science 2014;**346**(6210):763-7.

3. Resh VH and Unzicker JD. Water quality monitoring and aquatic organisms: the importance of species identification. J Water Pollut Control Fed 1975;**47**(1):9-19.
4. Holzenthal R, Blahnik R, Kjer K, et al. An update on the phylogeny of caddisflies (Trichoptera). In: *Proceedings of the 12th International Symposium on Trichoptera The Caddis Press, Columbus, Ohio* 2007, pp.143-53.
5. Holzenthal RW, Thomson RE and Ríos-Touma B. Order Trichoptera. Thorp and Covich's Freshwater Invertebrates (Fourth Edition). Elsevier; 2015. p. 965-1002.
6. Stewart RJ and Wang CS. Adaptation of caddisfly larval silks to aquatic habitats by phosphorylation of H-fibroin serines. Biomacromolecules 2010;**11**(4):969-74.
7. Ohkawa K, Miura Y, Nomura T, et al. Long-range periodic sequence of the cement/silk protein of *Stenopsyche marmorata*: purification and biochemical characterisation. Biofouling 2013;**29**(4):357-67.
8. Addison JB, Ashton NN, Weber WS, et al. β-Sheet nanocrystalline domains formed from phosphorylated serine-rich motifs in caddisfly larval silk: a solid state NMR and XRD study. Biomacromolecules 2013;**14**(4):1140-8.
9. Ashton NN and Stewart RJ. Self-recovering caddisfly silk: energy dissipating, Ca^{2+}-dependent, double dynamic network fibers. Soft Matter 2015;**11**(9):1667-76.
10. Ashton NN, Pan H and Stewart RJ. Connecting caddisworm silk structure and mechanical properties: combined infrared spectroscopy and mechanical analysis. Open Biol 2016;**6**(6):160067.
11. Yonemura N, Mita K, Tamura T, et al. Conservation of silk genes in Trichoptera and Lepidoptera. J Mol Evol 2009;**68**(6):641-53.
12. Wang Y, Sanai K, Wen H, et al. Characterization of unique heavy chain fibroin filaments spun underwater by the caddisfly *Stenopsyche marmorata* (Trichoptera; Stenopsychidae). Mol Biol Rep 2010;**37**(6):2885-92.
13. Ashton NN, Roe DR, Weiss RB, et al. Self-tensioning aquatic caddisfly silk: Ca^{2+}-dependent structure, strength, and load cycle hysteresis. Biomacromolecules 2013;**14**(10):3668-81.
14. Wang CS, Ashton NN, Weiss RB, et al. Peroxinectin catalyzed dityrosine crosslinking in the adhesive underwater silk of a casemaker caddisfly larvae, *Hysperophylax occidentalis*. Insect Biochem Mol Biol 2014;**54**:69-79.
15. Hwang CL. Descriptions of Chinese caddis flies (Trichoptera). Acta Zool sin 1958;**10**:279-85.
16. Xu JH, Wang BX and Sun CH. The *Stenopsyche simplex* species group from China with descriptions of three new species (Trichoptera: Stenopsychidae). Zootaxa 2014;**3785**(2):217-30.
17. Fu X, Li J, Tian Y, et al. Long-read sequence assembly of the firefly *Pyrocoelia pectoralis* genome. Gigascience 2017;**6**(12):1-7.

18. Chen S, Zhou Y, Chen Y, et al. fastp: an ultra-fast all-in-one FASTQ preprocessor. Bioinformatics 2018;**34**(17):i884-90.

19. Lander ES and Waterman MS. Genomic mapping by fingerprinting random clones: a mathematical analysis. Genomics 1988;**2**(3):231-9.

20. Marçais G and Kingsford C. A fast, lock-free approach for efficient parallel counting of occurrences of k-mers. Bioinformatics 2011;**27**(6):764-70.

21. Vurture GW, Sedlazeck FJ, Nattestad M, et al. GenomeScope: fast reference-free genome profiling from short reads. Bioinformatics 2017;**33**(14):2202-4.

22. Chin CS, Peluso P, Sedlazeck FJ, et al. Phased diploid genome assembly with single-molecule real-time sequencing. Nat Methods 2016;**13**(12):1050-4.

23. Chin CS, Alexander DH, Marks P, et al. Nonhybrid, finished microbial genome assemblies from long-read SMRT sequencing data. Nat Methods 2013;**10**(6):563-9.

24. Walker BJ, Abeel T, Shea T, et al. Pilon: an integrated tool for comprehensive microbial variant detection and genome assembly improvement. PLoS One 2014;**9**(11):e112963.

25. Li H and Durbin R. Fast and accurate short read alignment with Burrows–Wheeler transform. Bioinformatics 2009;**25**(14):1754-60.

26. Salmela L and Rivals E. LoRDEC: accurate and efficient long read error correction. Bioinformatics 2014;**30**(24):3506-14.

27. Wu TD and Watanabe CK. GMAP: a genomic mapping and alignment program for mRNA and EST sequences. Bioinformatics 2005;**21**(9):1859-75.

28. Kajitani R, Toshimoto K, Noguchi H, et al. Efficient *de novo* assembly of highly heterozygous genomes from whole-genome shotgun short reads. Genome Res 2014;**24**(8):1384-95.

29. Kiełbasa SM, Wan R, Sato K, et al. Adaptive seeds tame genomic sequence comparison. Genome Res 2011;**21**(3):487-93.

30. Pryszcz LP and Gabaldón T. Redundans: an assembly pipeline for highly heterozygous genomes. Nucleic Acids Res 2016;**44**(12):e113.

31. Kumar S, Jones M, Koutsovoulos G, et al. Blobology: exploring raw genome data for contaminants, symbionts and parasites using taxon-annotated GC-coverage plots. Front Genet 2013;**4**:237.

32. Simão FA, Waterhouse RM, Ioannidis P, et al. BUSCO: assessing genome assembly and annotation completeness with single-copy orthologs. Bioinformatics 2015;**31**(19):3210-2.

33. Weigand H, Weiss M, Cai H, et al. Fishing in troubled waters: Revealing genomic signatures of local adaptation in response to freshwater pollutants in two macroinvertebrates. Sci Total Environ 2018;**633**:875-91.

34. Ferguson L, Marlétaz F, Carter JM, et al. Ancient expansion of the Hox cluster in Lepidoptera generated four homeobox genes implicated in extra-embryonic tissue formation. PLoS Genet 2014;**10**(10):e1004698.

35. i5K Consortium. The i5K Initiative: advancing arthropod genomics for knowledge, human health, agriculture, and the environment. J Hered 2013;**104**(5):595-600.

36. Weigand H, Weiss M, Cai H, et al. Deciphering the origin of mito-nuclear discordance in two sibling caddisfly species. Mol Ecol 2017;**26**(20):5705-15.

37. Zdobnov EM, Tegenfeldt F, Kuznetsov D, et al. OrthoDB v9.1: cataloging evolutionary and functional annotations for animal, fungal, plant, archaeal, bacterial and viral orthologs. Nucleic Acids Res 2016;**45**(D1):D744-9.

38. Thiel T, Michalek W, Varshney R, et al. Exploiting EST databases for the development and characterization of gene-derived SSR-markers in barley (*Hordeum vulgare* L.). Theor Appl Genet 2003;**106**(3):411-22.

39. Xu Z and Wang H. LTR_FINDER: an efficient tool for the prediction of full-length LTR retrotransposons. Nucleic Acids Res 2007;**35**(suppl_2):W265-8.

40. Benson G. Tandem repeats finder: a program to analyze DNA sequences. Nucleic Acids Res 1999;**27**(2):573-80.

41. Tarailo-Graovac M and Chen N. Using RepeatMasker to identify repetitive elements in genomic sequences. Curr Protoc Bioinformatics 2009:4.10.1-4.

42. Kapitonov VV and Jurka J. A universal classification of eukaryotic transposable elements implemented in Repbase. Nat Rev Genet 2008;**9**(5):411-2.

43. Lagesen K, Hallin P, Rødland EA, et al. RNAmmer: consistent and rapid annotation of ribosomal RNA genes. Nucleic Acids Res 2007;**35**(9):3100-8.

44. Lowe TM and Eddy SR. tRNAscan-SE: a program for improved detection of transfer RNA genes in genomic sequence. Nucleic Acids Res 1997;**25**(5):955.

45. Burge SW, Daub J, Eberhardt R, et al. Rfam 11.0: 10 years of RNA families. Nucleic Acids Res 2012;**41**(D1):D226-32.

46. Fu H and Dooner HK. Intraspecific violation of genetic colinearity and its implications in maize. Proc Natl Acad Sci U S A 2002;**99**(14):9573-8.

47. UniProt Consortium. UniProt: a hub for protein information. Nucleic Acids Res 2015;**43**(D1):D204-12.

48. Birney E and Durbin R. Using GeneWise in the *Drosophila* annotation experiment. Genome Res 2000;**10**(4):547-8.

49. Haas BJ, Delcher AL, Mount SM, et al. Improving the *Arabidopsis* genome annotation using maximal transcript alignment assemblies. Nucleic Acids Res 2003;**31**(19):5654-66.

50. Haas BJ, Papanicolaou A, Yassour M, et al. *De novo* transcript sequence reconstruction from RNA-Seq: reference generation and analysis with Trinity. Nat Protoc 2013;**8**(8):1494.

51. Haas BJ, Salzberg SL, Zhu W, et al. Automated eukaryotic gene structure annotation using EVidenceModeler and the program to assemble spliced alignments. Genome Biol 2008;**9**(1):R7.

52. Duan J, Li R, Cheng D, et al. SilkDB v2. 0: a platform for silkworm (*Bombyx mori*) genome biology. Nucleic Acids Res 2009;**38**(suppl_1):D453-6.

53. Zhan S, Merlin C, Boore JL, et al. The monarch butterfly genome yields insights into long-distance migration. Cell 2011;**147**(5):1171-85.

54. Dasmahapatra KK, Walters JR, Briscoe AD, et al. Butterfly genome reveals promiscuous exchange of mimicry adaptations among species. Nature 2012;**487**(7405):94-8.
55. Davey JW, Chouteau M, Barker SL, et al. Major improvements to the *Heliconius melpomene* genome assembly used to confirm 10 chromosome fusion events in 6 million years of butterfly evolution. G3 2016;**6**(3):695-708.
56. You M, Yue Z, He W, et al. A heterozygous moth genome provides insights into herbivory and detoxification. Nat Genet 2013;**45**(2):220-5.
57. Moriya Y, Itoh M, Okuda S, et al. KAAS: an automatic genome annotation and pathway reconstruction server. Nucleic Acids Res 2007;**35**(suppl_2):W182-5.
58. Jones P, Binns D, Chang HY, et al. InterProScan 5: genome-scale protein function classification. Bioinformatics 2014;**30**(9):1236-40.
59. Ashburner M, Ball CA, Blake JA, et al. Gene ontology: tool for the unification of biology. Nat Genet 2000;**25**(1):25-9.
60. Li L, Stoeckert CJ and Roos DS. OrthoMCL: identification of ortholog groups for eukaryotic genomes. Genome Res 2003;**13**(9):2178-89.
61. Katoh K and Standley DM. MAFFT multiple sequence alignment software version 7: improvements in performance and usability. Mol Biol Evol 2013;**30**(4):772-80.
62. Talavera G and Castresana J. Improvement of phylogenies after removing divergent and ambiguously aligned blocks from protein sequence alignments. Syst Biol 2007;**56**(4):564-77.
63. Castresana J. Selection of conserved blocks from multiple alignments for their use in phylogenetic analysis. Mol Biol Evol 2000;**17**(4):540-52.
64. Stamatakis A. RAxML version 8: a tool for phylogenetic analysis and post-analysis of large phylogenies. Bioinformatics 2014;**30**(9):1312-3.
65. Yang Z. PAML 4: phylogenetic analysis by maximum likelihood. Mol Biol Evol 2007;**24**(8):1586-91.
66. De Bie T, Cristianini N, Demuth JP, et al. CAFE: a computational tool for the study of gene family evolution. Bioinformatics 2006;**22**(10):1269-71.
67. Fnu S, Williamson EA, De Haro LP, et al. Methylation of histone H3 lysine 36 enhances DNA repair by nonhomologous end-joining. Proc Natl Acad Sci U S A 2011;**108**(2):540-5.
68. Lee SH, Oshige M, Durant ST, et al. The SET domain protein Metnase mediates foreign DNA integration and links integration to nonhomologous end-joining repair. Proc Natl Acad Sci U S A 2005;**102**(50):18075-80.
69. Li X, Schuler MA and Berenbaum MR. Molecular mechanisms of metabolic resistance to synthetic and natural xenobiotics. Annu Rev Entomol 2007;**52**:231-53.
70. Feyereisen R. Insect CYP genes and P450 enzymes. Insect molecular biology and biochemistry. Elsevier; 2012. p. 236-316.
71. Hekmat-Scafe DS, Scafe CR, McKinney AJ, et al. Genome-wide analysis of the odorant-binding protein gene family in *Drosophila melanogaster*. Genome Res 2002;**12**(9):1357-69.

72. Dippel S, Oberhofer G, Kahnt J, et al. Tissue-specific transcriptomics, chromosomal localization, and phylogeny of chemosensory and odorant binding proteins from the red flour beetle *Tribolium castaneum* reveal subgroup specificities for olfaction or more general functions. BMC Genomics 2014;**15**:1141.
73. Gong DP, Zhang HJ, Zhao P, et al. The odorant binding protein gene family from the genome of silkworm, *Bombyx mori*. BMC Genomics 2009;**10**:332.
74. Pikielny CW, Hasan G, Rouyer F, et al. Members of a family of drosophila putative odorant-binding proteins are expressed in different subsets of olfactory hairs. Neuron 1994;**12**(1):35-49.
75. McKenna MP, Hekmat-Scafe DS, Gaines P, et al. Putative *Drosophila* pheromone-binding proteins expressed in a subregion of the olfactory system. J Biol Chem 1994;**269**(23):16340-7.
76. Eddy SR. Accelerated profile HMM searches. PLoS Comp Biol 2011;**7**(10):e1002195.
77. Jehamalar EE, Gloda D, Kiruba S, et al. Trichopterans as a bioindicators of a stream ecosystem. J Basic Applied Biol 2010;**4**:86-90.
78. Schmidt-Kloiber A, Neu PJ, Malicky M, et al. Aquatic biodiversity in Europe: a unique dataset on the distribution of Trichoptera species with important implications for conservation. Hydrobiologia 2017;**797**(1):11-27.
79. Kristensen NP. Phylogeny of endopterygote insects, the most successful lineage of living organisms. Eur J Entomol 1999;**96**:237-54.

Figure legends

Figure 1: An illustration of the adult caddisfly *Stenopsyche tienmushanensis* in its typical habitat.

Figure 2: Functional gene annotations using four databases.

Figure 3: The phylogenetic tree and gene expansion/contraction of 12 arthropod taxa. Multiple-copy orthologs represent the gene groups present in all species with a gene number > 1 in at least one species. Species-specific paralogs represent genes uniquely present in only one species. Other types of orthologs represent the gene groups that are absent in some species and not species-specific paralogs. Numbers of expanded gene families are marked in green, while numbers of contracted gene families are marked in red. MRCA: most recent common ancestor. The number below MRCA is the total group numbers from the OrthoMCL analysis. Note that only some of the gene expansions/contractions are significant.

Figure 4: The phylogenetic relationship of the significantly expanded gene groups of cytochrome P450 family in ten insect species. The phylogeny was constructed using Maximum Likelihood, showing significant expansions in *S. tienmushanensis*. The bootstrap values are marked on the nodes.

Figure 5: The Maximum Likelihood tree of odorant-binding proteins (OBPs) in five insect species. The bootstrap values are marked on the nodes. The expanded OBP groups in *S. tienmushanensis* are most closely related to those potentially responsible for pheromone detection in *Drosophila*.

Figure 6: The H-fibroin gene complex in *S. tienmushanensis*. The sequences of H-fibroin gene fragments previously reported from *S. marmorata* are referred from [7, 12]. (a) The comparison of H-fibroin genes between *S. tienmushanensis* and *S. marmorata*. The depth of PacBio read coverage is shown in the line plot (smoothed by a sliding

window average of 25 bps). The H-fibroin alignment of one representative tandem repetitive unit, non-repetitive 5' end, and non-repetitive 3' end between *S. tienmushanensis* and *S. marmorata* was shown in panels (b-d). Identical amino acids in alignment between *S. tienmushanensis* and *S. marmorata* were marked in grey shadow. The start and end positions of the nucleotides were shown in the alignment of the repetitive units. Amino acids in the black box represent the typical motifs of short repeat unit. S.tie: H-fibroin gene complex in *S. tienmushanensis*; S.mar5/S.mar3: the 5'/3' end nucleotides of H-fibroin mRNA fragments in *S. marmorata*. The marked intron near the 5' end of the gene complex (position: 43-124) was inferred from the alignment between the non-repetitive 5' end between *S. tienmushanensis* and *S. marmorata*, positioned between sequences coding for the 14th and 15th amino acids of the N-terminus of the first predicted protein. The other marked intron (position: 10643-10729) was identified near the 5' end of the second predicted gene, positioned between the second and third position in the codon for the 14th amino acid of the second predicted protein.

Tables

Table 1 Comparison of genome assemblies among five caddisfly genomes

Species	*Stenopsyche tienmushanensis*	*Glossosoma conforme*	*Glyphotaelius pellucidus*	*Limnephilus lunatus*	*Sericostoma* sp. HW-2014
Platform	PacBio + Illumina	Illumina	Illumina	Illumina	Illumina
Aseembly accession	v1	ASM334726v1	-	Llun_2.0	ASM300347v1
Sequencing depth	153 × + 150 ×	53.0 ×	8.12 ×	80.1 ×	43.0 ×
Total length (bp)	451,494,475	604,293,666	757,289,448	1,369,180,260	1,015,727,762
Scaffold N50 (kb)	1,297	16.7	1.47	69.1	3.1
BUSCO (n=1,658)	C:97.6%<br>(S:94.0%,D:3.6%)<br>F:1.1%<br>M:1.3%	C:85.2%<br>(S:84.2%,D:1.0%)<br>F:11.6%<br>M:3.2%	C:22.3%<br>(S:22.1%,D:0.2%)<br>F:39.9%<br>M:37.8%	C:86.7%<br>(S:80.9%,D:5.8%)<br>F:7.6%<br>M:5.7%	C:37.4%<br>(S:37.0%,D:0.4%)<br>F:38.8%<br>M:23.8%

The genome source: *G. conforme* [33], *G. pellucidus* [34], *L. lunatus* from i5K project [35], and *Sericostoma* sp. HW-2014 [36]. BUSCO annotation: C: complete BUSCOs; S: Complete and single-copy BUSCOs; D: Complete and duplicated BUSCOs; F: Fragmented BUSCOs; M: Missing BUSCOs.

Table 2 Summary of annotated repeats

Type	Combined TEs Length (bp)	% of genome
DNA	80,400,946	17.81
LINE	20,688,303	4.58
LTR	1,914,131	0.42
SINE	7,687	0.00
Other	15,267,487	3.38
Unknown	47,696,381	10.56
Total	165,974,935	36.76

"Other" represents a TE that is classified, but does not belong to one of our chosen classes. "Unknown" represents TE that could not be classified.

Figure 1

Click here to access/download;Figure;Figure_1_20180925.pdf

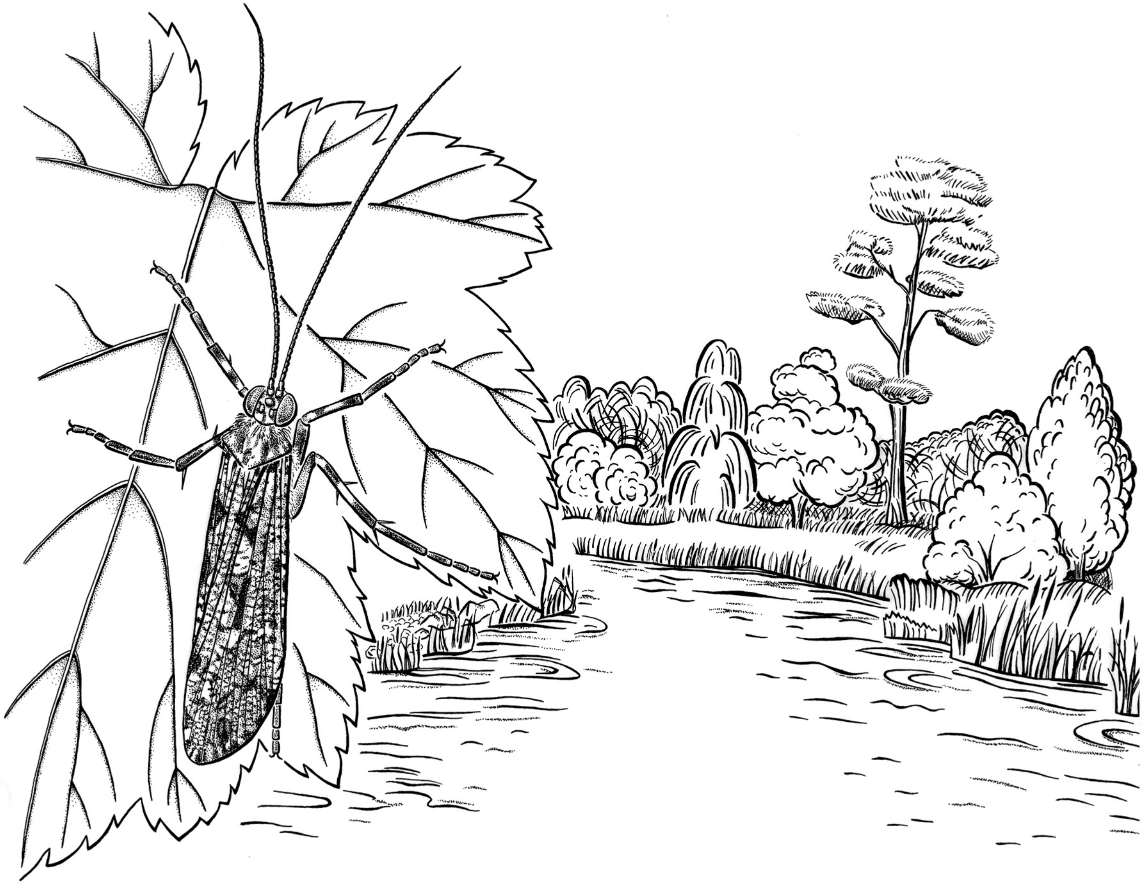

Figure 2

Click here to access/download;Figure;Figure_2_20180925.pdf

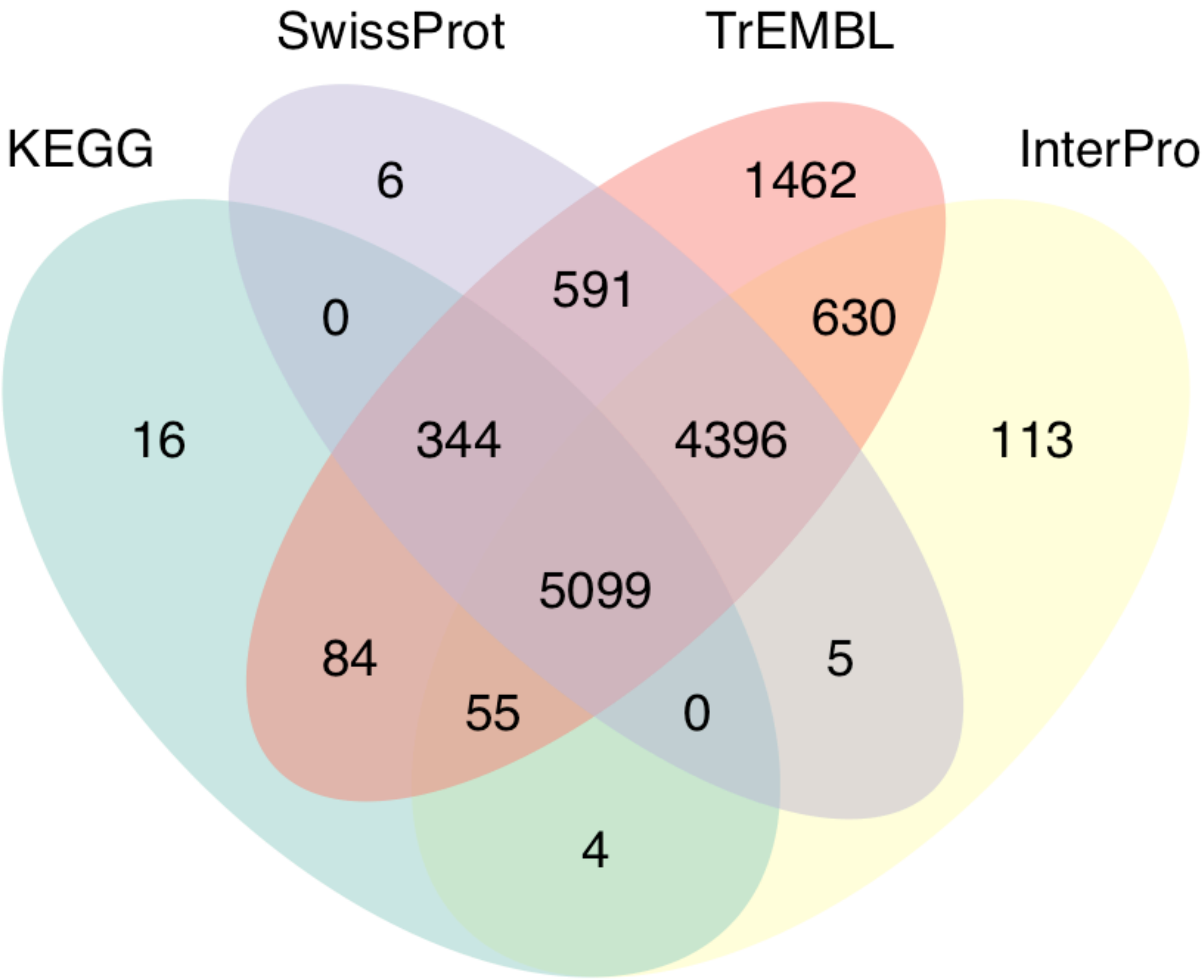

Figure 3 Click here to access/download;Figure;Figure_3_20180925.pdf

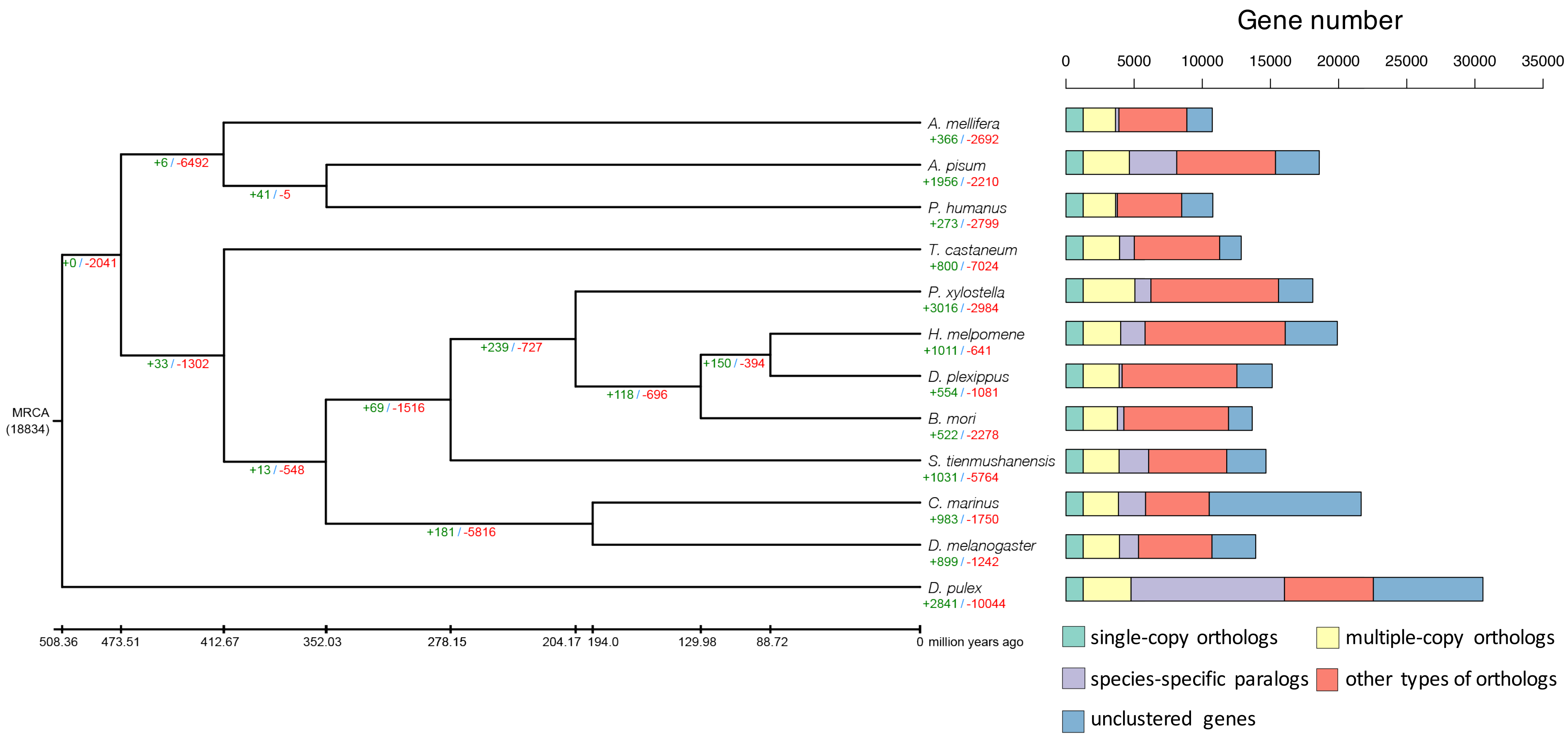

Figure 4

Click here to access/download;Figure;Figure_4_20180925.pdf

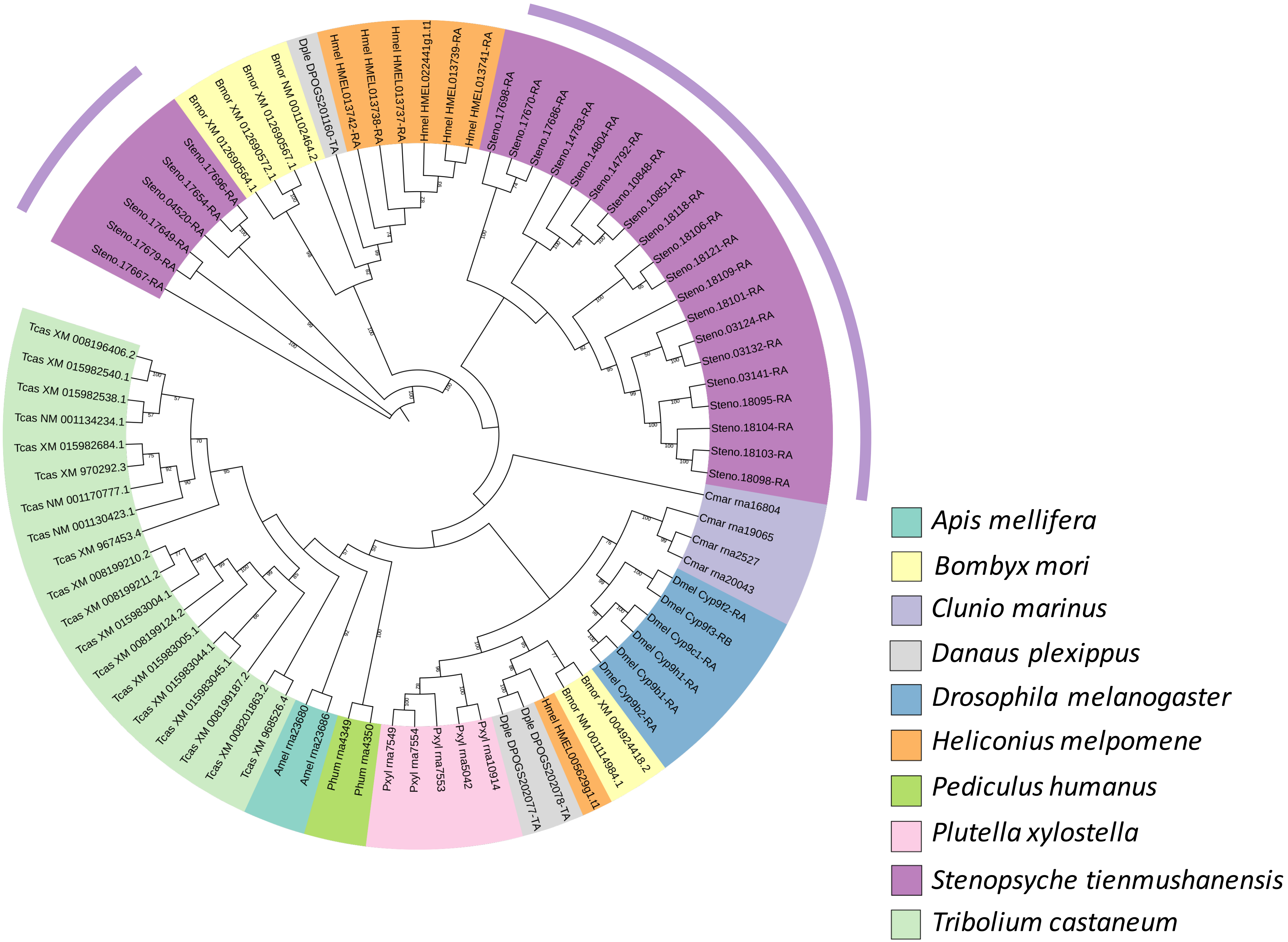

Figure 5

Click here to access/download;Figure;Figure_5_20180925.pdf

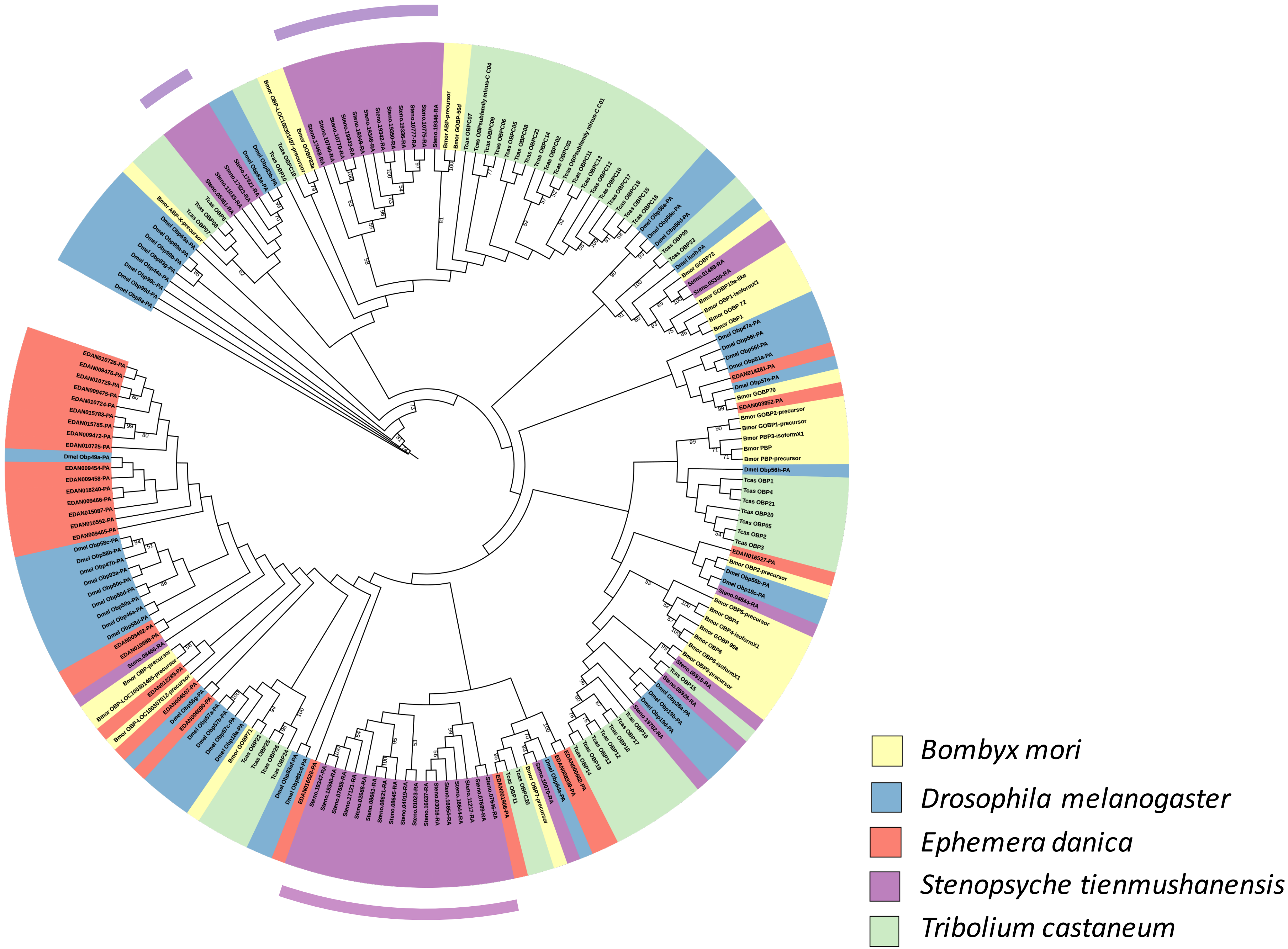

Figure 6 Click here to access/download;Figure;Figure_6_20180925.pdf

(a)

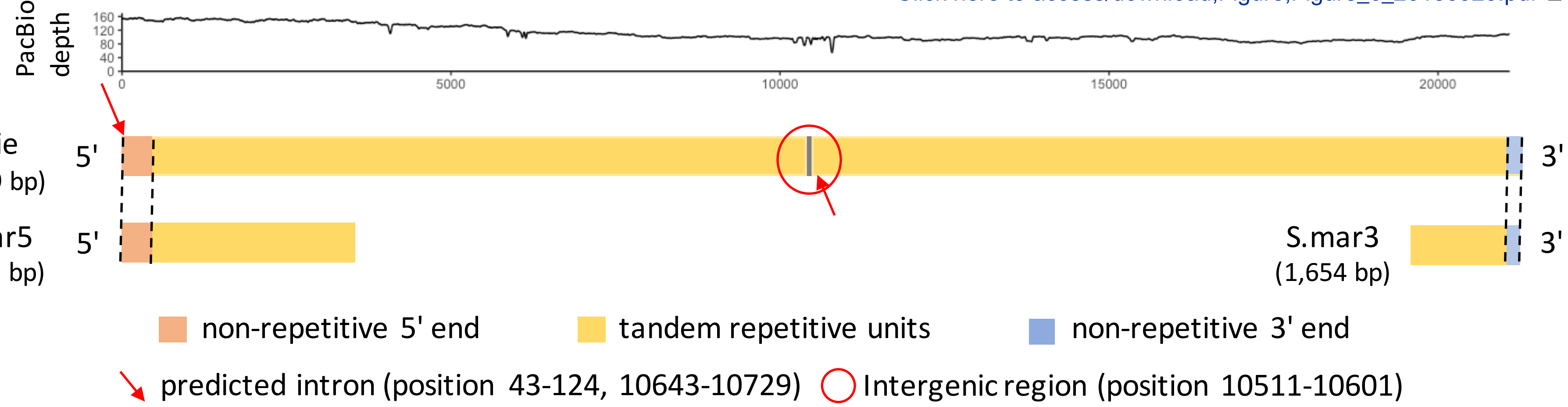

(b) tandem repetitive units

```
S.tie    7841 GPGYYGPGFVGPRRGKASASHSVSVETYVRAPIVRHFSRSGSVSIERPQYYYRPG  8005
S.mar3   525  GPGYYGPGFVGPRRGKASVSHSVSVETYVRAPIVRHFSRSGSVSIERPQYYYRPG  689

S.tie    8006 KISKSKSVSFEQVYVPPVVQHVKKSGSVSVERPQYFYRPGKVSVSRSYSYERVVR  8170
S.mar3   690  KISKSRSISVEQVYVPPVIQHVKKSGSVSVERPRYFYRPGKVSVSRSYSYERVVR  854

S.tie    8171 PARVFNRVSHSASVSVERPRHFVRPAVVARSGSFSAEGGWGRGSYGPGGLLG     8326
S.mar3   855  PARVFNRVSHSASVSVERPRHFVRPAVIARSASFSAEGGWGHGPYGHGGLLG     1010
```

(c) non-repetitive 5' end

```
S.tie    MRAVLFLILFCSLQIHLTGACNKPGKNVIGKLENFLSHGHLNPHVGLHEKILQGDDRIEANSRGDLIEKIISRKEILTD
S.mar5   MRAVLFLILFCSLQIHLTGACNKPGKNVIGKLENFLYHGHLNPHVGLHEKILQGDDRIEAKSRGDLIEKIISRKEILTD

S.tie    DDSESFSVSYSDESTEQIIKTITIVQEKPKHGGRAKEKIYEEEVVIKKVGEVPRDTKIAGCKSSEGIAGIGGIRRAWAS
S.mar5   DDSESFSVSYSDESTEQIIKTITIVQEKPKHGGRAKEKIYEEEVVIKKVGEVPRDTKIAGCKSSEGIAGIGGVRRAWAA
```

(d) non-repetitive 3' end

```
S.tie    DDDVVGGWAPSYGGSVVPFVDGGVSSVGGYGSIPQAVVYTRHPDPRTVRSPCKTSPFQLLINVGNSRKRAGNC
S.mar3   IFDVGAQLPSAYGGSVAPLVYGGVSSLGGYGSIPQAVVYTRHPDPRTVRSPCKSSPFHLLINVGNSRKRAGNC
```

Click here to access/download
Supplementary Material
SOM_20180925.docx

Table S3

Click here to access/download
Supplementary Material
Table S3.xlsx

Response to reviewers
Click here to access/download;Personal Cover;Response_to_reviewers_20180925.docx

September 25th, 2018
Dr. Scott C. Edmunds
Executive Editor
GigaScience

Dear Dr. Edmunds,

We are submitting the revised version of the manuscript "*The genome of an underwater architect, the caddisfly Stenopsyche tienmushanensis Hwang (Insecta: Trichoptera)*" by Luo *et al.* (GIGA-D-18-00136). Thank you very much for handling our previous submission and giving us the opportunity to revise this manuscript.

We appreciate the constructive comments from both reviewers and herein address all questions raised. Briefly, in the revised version, we have clarified key analytical steps and conducted additional analyses. We have also re-organized the writing to make it more clear. The major revisions include: (1) We re-organized the "Genome assembly and polishing" and "Transcriptome analysis" sections to clarify why and how the key steps were conducted, as suggested by both reviewers. (2) We updated caddisfly genome information in Table 1 and the main TEXT following suggestions from Reviewer #1. (3) To improve the quality of the genome assembly and annotation, we added the "Heterozygosity estimation" section, and filtered for potential heterozygous and contaminated contigs with programs suggested by Reviewer #2. We also updated all relevant genome statistics throughout the manuscript. (4) We conducted estimations for genome size and heterozygosity using GenomeScope as suggested by Reviewer #2.

We also polished the writing and corrected typos that we could identify, and made changes to address the concerns of both reviewers. We marked all major revisions in RED in the TEXT. At last, we included a point-to-point response to both reviewers. We believe the revised manuscript has been significantly improved, thanks to both reviewers and editors of *GigaScience*. We hope this revision is satisfactory and we are looking forward to hearing further updates.

Sincerely yours,

Xin Zhou, on behalf of all authors
Beijing Advanced Innovation Center for Food Nutrition and Human Health &
College of Plant Protection,
China Agricultural University, Beijing 100193, China
Email: xinzhou@cau.edu.cn

Point-to-point response to the reviewers
(Line numbers mentioned in the response may not coincide with the original line numbers.)

Reviewer #1: Luo et al. present a high-quality genome assembly for the caddisfly Stenopsyche tienmushanensis, a first for the entire insect order Trichoptera. This is an exciting, important advance that greatly improves existing genomic resources for the group. In their assembly, the authors integrate short-read and long-read data for both DNA and RNA to improve the contiguity of their assembly and their gene annotations. In addition to a high-quality genome, the authors incorporate analyses that assess possible gene family expansions and contractions in caddisflies with a specific focus on cytochrome P450 and olfactory binding proteins. They also present the first full sequence of the H-fibroin gene which is the key factor underlying the unique, aquatic silk produced by caddisflies in their case-making activities.

The generated data and subsequent analyses both appear robust for the task at hand. My most overarching critique, and the general theme in my comments below, is one of clarity. Both the writing style and content of the paper regarding individual analyses and results, need to be substantially improved in the revision. My general comments focus on sections that are particularly deficient or problematic in this regard and I follow with more specific comments on a line-by-line basis.

Simply put, my review can be summarized as follows: This is an important, timely paper that needs additional polish.

Response: We would like to thank you for the positive feedback on our paper, and the great effort in formulating a nice summary and constructive comments to improve our work.

General comments:
Writing style/clarity:
I won't belabor this point any more than I already have but the paper is riddled with missing words and confusing grammar which makes it difficult to read and, more importantly, difficult to understand in several key areas. A few minor examples include: Line 25 -- there's no need for "the" after "adaptations to", Line 26 -- pluralizing "architectures" is odd in this context, Line 95 - "prestine" should be "pristine", etc. Active versus passive voice also varies and being consistent in this regard should be considered while revising. Personally, I prefer active voice. More major examples include a general lack of satisfactory detail in important sections like the "Genome assembly methods."

Response: Thanks for the careful scrutiny on our paper. In the new version, we have changed the sentence on line 26 to "Among the numerous adaptations to aquatic habitats" and replaced "architectures" on line 27 to "composite structures" and

corrected “pristine” on line 100. We have also re-written the analysis step by step to make it clear in the TEXT, and changed most sentences to active voice in the revised manuscript. We have also corrected any grammar errors that we managed to find during the revision.

Genome assembly methods:
Since the manuscript is centered upon this part of the process, that is, how the genome assembly was produced, it's essential that this section be improved. The authors should revise with a focus on briefly explaining each step, why it was performed, and how it fits into the broader picture. For instance, reads from both Stie1 and Stie2 were used for both PacBio and Illumina sequencing, correct? If so, why specify Stie1 and Stie2 on Line 137 if there are no other options? What does it mean to "rectify" a genome assembly with Arrow? Why map your short reads back to your PacBio only assembly? Just to be sure they map at a high rate? You discuss "another revision using Pilon"? What does this mean? Where is the first revision and what was its purpose? Beginning in the 2nd paragraph of this section (Line 144), it's unclear to me what the "corrected, intermediate assembly" is. For the transcriptome sequencing, there was no assembly, correct? You took complete PacBio reads and consider them to be your transcripts. If this is true, what role did the Illumina short-reads from RNA sequencing data play in the process?

Response: We have re-written the corresponding sections to clarify these details. Briefly, we pooled DNA from Stie1 and Stie2 for PacBio sequencing. Additionally, we sequenced Stie1 and Stie2 separately with Illumina using the remaining DNA. We specified Stie1 and Stie2 on Line 164 to show the different mapping rates of the two individuals to the initial genome assembly (based on PacBio).

To assemble the genome, we firstly produced an initial assembly of 510.7 Mb with PacBio data using Falcon. Then we conducted genome assembly polishing. Polishing seeks to resolve base calling errors and indels introduced during the sequencing or assembly, aiming to improve the genome assembly. In case of PacBio sequencing, which have higher error rates, we employed both self-correction and Illumina-based correction (which had much lower error rates in sequencing). After the *de novo* assembly, we conducted a first round of genome polishing using PacBio subreads to improve the accuracy of the assembly. We used BLASR in SMRTlink 4.0 to map all reads to the assembly, then used Arrow (part of the SMRT analysis suite) to produce consensus sequences. As successor of Quiver, Arrow employs an improved consensus model based on a more straight-forward hidden Markov model approach. To further rectify the high error rate from PacBio sequence data, we conducted two additional rounds of polishing with Illumina data using Pilon. Firstly, we used the output bam files from one individual that had higher coverage (Stie2, 99.20%, compared to 97.07% in Stie1) to polish the Arrow-corrected genome assembly. For the second round of polishing, we mapped all Illumina reads (from both Stie1 and Stie2) to the Pilon and

Arrow corrected assembly using bwa-mem. We then ran the resulting bam file through Pilon again, producing an assembly of 512.7 Mb.

In the new version, we added a “Transcriptome analysis” section to explain how we produced the non-redundant, full-length transcriptome from PacBio Iso-Seq data. We followed the PacBio RS_IsoSeq protocol to generate 118,776 full-length transcripts with PacBio raw data (details described in the TEXT). The analysis protocol includes three main steps (http://files.pacb.com/software/smrtanalysis/2.3.0/doc/smrtportal/help/Webhelp/CS_Prot_RS_IsoSeq.htm): 1) Classify: Extract insert reads from PacBio movies; then classify insert reads into chimeric or non-chimeric, and full-length or non-full-length reads. 2) Cluster: Predict de novo consensus isoforms of transcripts from the classified reads using the Iteratively Clustering and Error Correction (ICE) algorithm. Then polish the isoforms with PacBio Iso-Seq data using Arrow. We also used llumina RNA-seq data to correct/polish the PacBio-generated full-length transcript after the Clustering step. These Illumina RNA-seq data were only used in the polishing step. 3) Map: Retain only the transcripts that could be aligned to the intermediate genome assembly with GMAP. We produced 112,307 full-length transcripts and collapsed them to 22,347 consensus transcript isoforms. All details have been added in the TEXT.

Gene prediction methods:

Similar to my comments about the genome assembly methods. This paragraph/section needs to be more clear/thorough. For instance, are there no parameters to specify in Augustus? Did you use defaults? Why 1,000 transcripts ab initio training? You need to report the number of transcripts recovered (Line 130), not just the total length of them. Particularly the number of "full length" transcripts since that appears to be what you used in lieu of any transcript assembly method. I would also like to see a min./max. of the range of transcript sizes. Also, how is training Augustus with transcripts not RNA-seq-assisted? You begin by mentioning three methods of gene prediction, then never mention the "RNA-seq-assisted" again. The reference for EVM should be moved to the end of the sentence as it also includes PASA (which I also think should be spelled out in parentheses, "Program to Assemble Spliced Alignments"). But, aside from specific comments, please revise this section with a focus on clarity. What exactly did you do and why?

Response: We have modified it as suggested. In the gene prediction section, we used three methods, *ab initio* (AUGUSTUS), homology-based (GeneWise) and RNA-seq-assisted (PASA and transdecoder), and then combined the three results using EVM. We have modified the sentences to make the three methods more clear, and the spelling of the PASA and the EVM references have also been corrected in the revised version as suggested.

The steps in AUGUSTUS include model training and *ab initio* gene prediction based on the trained model parameters. AUGUSTUS may use transcriptomes of the focal species to train the model. Alternatively, the program would use a built-in model from a known species that is most closely related to the focal taxon, when transcriptomes of the latter are not available. In our case, we applied full-length transcriptomes of the sequenced caddisfly to train more accurate model parameters. We selected 1,000 non-redundant full-length transcripts from the 22,347 unique transcript isoforms identified from the PacBio Iso-Seq. We have added more details explaining the transcriptome assembly and provided the number of recovered transcripts (22,347 unique transcript isoforms) in the TEXT. We have also added “The mean length of all resulting transcripts was 2,881 bp, ranging from 274 to 13,820 bp” in “Transcriptome analysis” section as suggested.

Gene orthology analysis and phylogenetic tree:
"The region with low quality was filtered with Gblocks" (Lines 247-248). Please clarify this statement. This is minor but I would re-phrase wording like "is closely related to OBP4a" to "is most closely-related to OBP4a" because you aren't giving any quantified value of how closely related they are, just that the D. melanogaster gene family is the most similar one in the database but for all your reader knows, it may also be one of the only options in the database making "close" kind of meaningless.

<u>Response</u>: We have changed the sentence to “We used Gblocks (version 0.91b, with parameters -b5=h) [62, 63] to filter out poorly aligned positions.” We also re-phrased the wording as “was most closely related to OBP84a”.

Concluding remarks:
I would like to see a couple more references in this section. For example, "among the strongest supported ordinal relationships" (Line 322). Also, I think the closing sentence could be stronger. Is the publication of a caddisfly genome only suited to understanding how insects adapted to terrestrial habitats? I would expand this to include aquatic vs. terrestrial lifestyles, the evolution of case-making (the coolest thing about caddisflies in my opinion!), and the evolution of a robust, aquatic silk. The concluding remarks should be summarizing the entire study, not only the paper's most recent line of thinking.

<u>Response</u>: We appreciate that you agree with us on the importance of constructing high-quality genomes for the caddisflies. We also share your feeling that the caddisflies are indeed fascinating underwater architects. Their aquatic lifestyle, case-making behavior, and the strong aquatic silk are worthy of deep investigation. We have added several references: “With a known diversity of over 16,000 species, caddisflies are important members of freshwater ecological communities and their species have been shown to be effective indicators of freshwater health [33, 77, 78]”, and “While Trichoptera and Lepidoptera are reciprocally monophyletic and among the

strongest supported ordinal level relationships within insects [2, 79],". The relevant references are listed below.

2. Misof B, Liu S, Meusemann K, et al. Phylogenomics resolves the timing and pattern of insect evolution. Science 2014;346(6210):763-7.
33. Weigand H, Weiss M, Cai H, et al. Fishing in troubled waters: Revealing genomic signatures of local adaptation in response to freshwater pollutants in two macroinvertebrates. Sci Total Environ 2018;633:875-91.
77. Jehamalar EE, Gloda D, Kiruba S, et al. Trichopterans as a bioindicators of a stream ecosystem. J Basic Applied Biol 2010;4:86-90.
78. Schmidt-Kloiber A, Neu PJ, Malicky M, et al. Aquatic biodiversity in Europe: a unique dataset on the distribution of Trichoptera species with important implications for conservation. Hydrobiologia 2017;797(1):11-27.
79. Kristensen NP. Phylogeny of endopterygote insects, the most successful lineage of living organisms. Eur J Entomol 1999;96:237-54.

Also, we have added sentences at the end of the paper to address the importance of our project "The addition of a high-quality trichopteran genome has the potential to deliver insights into the genetic basis of diverse strategies of insects to adapt to divergent habitats and to uncover the genomic differences between aquatic and terrestrial lifestyles. In particular, the caddisfly genome may help to gain deeper understanding of the evolution of the fascinating case-making behaviors and the robust aquatic silk of these underwater architects.

Supporting data:
The final genome assembly, gene models, and transcripts should be submitted to GenBank and linked with the existing BioProject/SRA archive. Accessioning it on the GigaScience database (GigaDB), while useful, is not sufficient in my opinion.

Response: The final genome assembly, gene models and transcriptomes will become available in a BioProject on Genbank upon acceptance of the manuscript.

Figure 3 - I think you can remove the statement about single-copy orthologs from the legend. Or, if you want to include it, please make its definition less circular. The definition for species-specific paralogs also appears incorrect - or do you mean these are in fact genes with multiple copies in only one species?

Response: We have removed the statement about single-copy orthologs from the legend as suggested. "Species-specific paralogs" refers to gene group uniquely present in only one species, including those with multiple copies or just a single copy.

Figures 4 & 5 - Please make the colorations of species represented in both figures match one another (e.g., S. tienmushanesis & T. castaneum should have the same coloration in both figures).

Response: Done.

Figure 6 - This figure and its legend needs to be clarified. A/B/C for the breakouts would help. And, is it correct that you're saying the gene is 21,089 bp long with only one ~80 bp intron? It is also unclear what the various measurements (3,543 and 1,654 bp) refer to exactly. Also, why is S. marmorata abbreviated as "S.mar5" and not just "S.mar"?

Response: The H-fibroin protein is indeed very large, with multiple repetitive blocks in the middle. Before this study, researchers have only cloned a small proportion of the H-fibroin genes of *Stenopsyche marmorata*, including the non-repetitive 5' and 3' ends (denoted as S.mar5 and S.mar3 in the original figure, respectively). The cloned S.mar5 is 3,543 bp long, containing a non-repetitive 5' end and some adjacent repetitive blocks. Similarly, the cloned S.mar3 is 1,654 bp long, containing a non-repetitive 3' end and some adjacent repetitive blocks. To better present these findings, we have organized the new figure into 4 panels (a-d). Fig. 6a shows the difference in the completeness of the H-fibroin genes described for *S. tienmushanensis* and *S. marmorata*. Fig. 6b-d show the alignments of the representative tandem repetitive unit, non-repetitive 5' end, and non-repetitive 3' end, respectively, between the two species.

By aligning our genome annotation results with the known 5' and 3' ends of the H-fibroin from *S. marmorata*, we were able to identify the precise start and end points of the H-fibroin gene complex for *S. tienmushanensis.* The total length between the start and end is 21,089 bp. The complete H-fibroin gene complex includes two similarly sized predicted genes with a short intergenic region. PacBio sequencing results show a coverage depth of > 100x with many reads spanning across large proportions of the gene range, including the intergenic region, assuring the validity of the assembly (Fig. 6a). From the gene annotation results, two introns were predicted in the region of the H-fibroin gene complex. One 82bp-intron was inferred near the 5' end of the gene complex of *S. tienmushanensis* (Fig. 6a, first arrow), positioned between sequences coding for the 14th and 15th amino acids of the N-terminus of the first predicted protein (Fig. 6c). The other 87bp-intron (position: 10643-10729) was identified near the 5' end of the second predicted gene (Fig. 6a, second arrow), positioned between the second and third position in the codon for the 14th amino acid of the second predicted protein.

Since the H-fibroin gene structure was predicted based on homology, the exact structure of the H-fibroin genes needs to be confirmed using full-length transcript of the larval silk gland, which is being planned for next year when matured larvae become available. We added "For future studies, transcriptome and gene expression analysis from larval silk glands will help elucidate additional structural details of H-fibroin." We also added relevant information of the two predicted introns in the legend of Figure 6.

Table 1 - Please add the accession information for the L. lunatus and G. pellucidus genomes. You can make this a new row in the table. Please also report the contig N50 in kb and add additional detail to the BUSCO results for your reader. You do not need to include the number of genes searched as that is included in the text. I would only report complete (with percentage in parentheses), fragmented (and %), and missing (and %). These can all be their own rows. As is, your audience doesn't know what C/S/D/etc. mean. You should also consider adding the Sericostoma sp. assembly on GenBank (https://www.ncbi.nlm.nih.gov/assembly/GCA_003003475.1) to the table (with corresponding BUSCO results and update any references in the text - if there are any - to four caddisfly genomes, not three.

Response: Thank you for informing us of the newest caddisfly genomes and the suggestions about Table 1. We have added two new caddisfly genomes (*Glossosoma conforme* and *Sericostoma* sp.) in our analysis, with relevant accession information and references. In the new Table 1, the "Assembly accession" row has been added. The N50 is now shown in kb. The results of BUSCO have been updated, and the details are shown in several rows. The meanings of C/S/D/F/M have been added at the bottom of the table. We still keep "1,658", the gene number used in BUSCO calculation in Table 1 for easy data access to readers.

Table 2 - Please provide a more informative legend specifying, briefly, what categories like "Other" and "Unknown" mean. Also, I would prefer the totals for DNA to Unknown to be in order from highest to lowest percentage of the genome. And, a line separating "Total" from the others would be useful since it is a summation and not a category per se like the rest.

Response: We have added the explanations for the "Other" and "Unknown" categories at the bottom of the revised Table 2. The line separating "Total" from the others has been added as suggested. We would prefer to use the original type order, as it is based on the classification of transposon types, such as DNA transposon, LINE, LTR, SINE, the other classified type and unknown type. This classification is based on structures of transposable elements and is more biologically relevant.

Table S1 - Was the short-read RNA data from Stie3 used in any way in the manuscript? If so, this needs to be described in the text (though it's possible I have overlooked it).

Response: The short-read RNA-seq data from Stie3 was used to polish the full-length transcripts from PacBio long-read RNA data. We have mentioned it in the earlier response, and please see "Transcriptome analysis" section in the revised version.

Table S3 - Please include a more informative legend. "Number" for Total examined sequences = 557 - this is the number of contigs, correct? Is this useful? It just says you examined everything in the "Final" assembly. And are the other values numbers or sizes?

Response: Thanks for the suggestion. The original Table S3 did not have enough information to suffice a table. We have deleted the original Table S3 and added relevant information in TEXT: “In total, we identified 91,564 simple sequence repeats (SSR, 4,217 with compound format) with the MIcroSAtellite identification tool (MISA, v1.0, RRID:SCR_010765) [37] using default parameters (see Table S4 for types of SSR)”.

Table S5 - Again, this needs more information in the legend and "Total" should be broken out from the other categories. Also, what does "Total" mean exactly? It's unclear from the listed categories.

Response: We have added a line separating “Total” from the others. The data shown in Table S5 described the results from different methods. The category “Total” refers to the combined repeat sequences from all five methods listed above. We have added relevant information in Table S5.

Specific comments:
Lines 30-31 - I would prefer these values be stated as the number of reads or removed entirely and discussed elsewhere. In the Abstract, I think it's alright to just say that you leveraged both short- and long-reads for the assembly.

Response: Thanks for the suggestion. In genome sequencing projects, data size is usually relevant to the quality of the genome, with deeper sequencing generally assuring better genome assembly and annotation. Although there are many other aspects that would affect the final genome quality, this trend is clearly shown in the caddisfly genomes we have examined in our paper. Additionally, library types and insert lengths are both key parameters to consider in genome sequencing and these vary between genome projects (shown in Table S1). It is important that this information is made available to the readers. Therefore, we have provided the sequencing information in both number formats (read number and total base pairs) “In total, 601.2 M Illumina reads (90.2 Gb), and 16.9 M PacBio subreads (89.0 Gb) were generated”

Lines 48-49 - Break this into two sentences. Right now, it implies that if you include Antarctica there are more diverse insect orders. Of course you don't mean that!

Response: We have modified the sentence to “Comprising >16,000 species and distributed worldwide except for Antarctica, caddisflies (Insecta: Trichoptera) are the most diverse of the strictly aquatic insect orders”.

Line 51 - The Misof et al. (2014) paper should be cited after the divergence timing estimate.

Response: We have moved the citation to the suggested place.

Line 86 - I would prefer "aquatic" over "watery."

Response: We have replaced "watery" with "aquatic".

Line 92-93 - I don't think the historical note about Chinese taxonomy is relevant. It's distribution in China is relevant though.

Response: *S. tienmushanensis* was one of the first caddisfly species ever described by a Chinese entomologist (C-L Hwang 1958). Sixty years from its original description, we now have the opportunity to understand its genome feature, an evidence for the development of Trichopterology in China. Therefore, we would prefer to maintain this note, which was written intentionally as our salute to a fellow pioneer Chinese trichopterologist.

Line 105 - I would like a bit more detail about the DNA extraction procedure given that high-molecular weight DNA is crucial to long-read sequencing. I know you gave the Fu et al. reference, but a bit more detail here without having to track down another reference would be useful for the reader.

Response: Thanks for the suggestion. The DNA was extracted with SDS and proteinase K. The protocols could be viewed from Protocols.io. The sentence has been modified as "DNA was extracted with SDS and proteinase K using the same protocol developed by Hu et al. (https://www.protocols.io/view/dna-extraction-procedure-using-sds-jg4cjyw) [17]."

Line 111 - Capitalize "ten" in the name of the sequencer.

Response: Changed to "HiSeq X Ten" in the TEXT and Table S1.

Line 112 - What does a "genome survey was carried out" mean? What was the point of using jellyfish on your data?

Response: A genome survey is a standard procedure for genome size estimation before the formal assembly using Illumina sequence data. One of the commonly used approaches is the *k*-mer profile analysis. The genome size is calculated by $G=K_{num}/K_{depth}$ (Lander & Waterman 1988, PMID: 3294162), where K_{num} is the total counts of *k*-mer, and K_{depth} is the *k*-mer depth. Jellyfish is used to calculate the distribution of *k*-mers. In the revised manuscript, we also used a newly published software GenomeScope (Vurture et al., 2017, PMC5870704) to estimate the genome size (Figure S3), as recommended by reviewer #2. Instead of using Poisson distribution, GenomeScope uses a mixed negative binomial model. We have removed this jargon in the revised text.

Lines 115-116 - How was a heterozygosity rate inferred from Arabidopsis? Reference for this method?

Response: The *k*-mer profile can reflect the complexity of the genome: homozygous genomes have a simple Poisson profile, while heterozygous genomes have a characteristic bimodal profile (*k*-mer distribution with two peaks, Kajitani et al., 2014, PMC4120091). The results from the original Figure S1 indicate that the genome of *S. tienmushanensis* is heterozygous. To estimate the heterozygosity rate, we used simulated genome data of *Arabidopsis thaliana* with varied heterozygosities and appropriate depths, and plotted the *k*-mer profiles. By comparing the *k*-mer profiles among different simulated heterozygous genome data and the empirical data from *S. tienmushanensis*, we could estimate the level of heterozygosity for *S. tienmushanensis* (Figure S4). This heterozygosity estimation method is used in many genome projects (Kajitani et al., 2014, PMC4120091, Glanzmann et al., 2016, PMC5142436). In the new version of the manuscript, we also estimated the heterozygosity using GenomeScope (Figure S3), which produced a similar result.

Line 121 - This is redundant. You already stated that Stie3 was used for RNA sequencing.

Response: We have modified the sentence as "We sequenced RNA samples using the Illumina HiSeq X Ten platform (insert-size of 180 bp, 150PE) and the PacBio Sequel system (Iso-Seq, library size 0.5-6k), which produced 9.72 Gb and 10.31 Gb data, respectively (Table S1)."

Line 127 - What is the "ICE" algorithm? Reference?

Response: The full name of "ICE" is Iteratively Clustering and Error Correction. The ICE algorithm is used in the SMRT Analysis software provided by PacBio company. There is no relevant published reference yet.

Line 130 - I would prefer to know how many transcripts this refers to. You can put the total basepairs in parentheses if you'd like to keep it included.

Response: In the "Transcriptome analysis" section, we have listed the transcript number and total bases in the TEXT: "resulting in 272,511,198 bp of 118,776 full-length transcripts."

*Line 153 - As currently written, this implies there are three existing caddisfly genomes, there aren't. There are two (actually three, Sericostoma sp. - https://www.ncbi.nlm.nih.gov/assembly/GCA_003003475.1) and your new one. I would re-phrase to something like "a comparison between the S. tienmushanensis and *three publicly available caddisfly genomes (names and references) is provided in Table 1."*

Response: Thanks for the information on these new genomes. We followed your advice and checked all available caddisfly genome sequences. We had added the new genomes in the TEXT: “The comparisons among the five available Trichoptera genome assemblies (including: *Glossosoma conforme* [33], *Glyphotaelius pellucidus* [34], *Limnephilus lunatus* provided by i5K [35] and *Sericostoma* sp. HW-2014 [36]) are shown in Table 1”.

Line 185 - You spell out non-coding RNAs but nothing else in this paragraph. I would revise to spell them all out before using abbreviations.

Response: We have modified the subheading as “Repeat analysis and non-coding RNA (ncRNA) annotation”

Line 214 - No need for "The source of genome assemblies:" - you can simply list the species and their genome accession information.

Response: This phrase is now removed.

Line 231 - Add generation after phylogenetic tree? This subheading needs at least one more word.

Response: We have modified the subheading as “Gene orthology analysis and phylogenetic tree reconstruction”.

Line 236 - Strange wording in the parentheses. Just say something like "See Table S8 for additional details".

Response: The sentence has been modified as suggested.

Lines 269-270 - You stated this was species-specific at the beginning of the sentence, no need to say it again after the Fig. S4 reference.

Response: We have deleted the second “species-specific”, and the new sentence is “For the species-specific paralogs of *S. tienmushanensis* revealed by the OrthoMCL analysis, GO enrichment (Fig. S8) revealed gene expansions of the odorant binding proteins (OBPs).”

Line 273 - "PBP_GOBP" - clarify what exactly this is and consider re-structuring this sentence.

Response: The PBP_GOBP family is a protein family listed in Pfam. This family composed of pheromone binding proteins (PBP) and general odorant binding proteins (GOBP). We have modified the sentence as “The PBP_GOBP family (PF01395 in Pfam), including pheromone binding proteins (PBP) and general odorant binding proteins

(GOBP), was used to search for OBPs in the mayfly genome obtained from the i5K project…".

Lines 280-283 - I don't mind speculation in a genome note but I'd like to see some references for the first sentence of this statement, followed by some evidence that caddisflies are particularly adept at finding mates versus other short-lived aquatic insects. Or, perhaps, you could further develop the argument that this may be generally true for aquatic insects. But don't mayflies have very short adult lifespans? Often shorter than caddisflies? If your hypothesis is correct, why don't they have greatly expanded OBP gene families?

Response: We appreciate your critical thinking. Although a convergent genomic mechanism might be responsible for the elevated efficiency in finding mates in short-lived insects, it is not likely the case for caddisflies and mayflies. Many mayflies typically form mating swarms, which may effectively increase the rates of successful copulation. But such a phenomenon has not been reported in *Stenopsyche* caddisflies. Although *Stenopsyche* adults emerge in a synchronized pattern, the population density is far lower than that observed in mayflies. It would not be unreasonable to presume that *Stenopsyche* adults (perhaps many other caddisflies as well) are under selective pressure to complete the mating process in a timely fashion, considering that reproduction is the sole role for adult caddisflies (most caddisflies don't feed as adults due to simplified mouthpart structures). On the other hand, even if mayflies do not form mating swarms, they may not converge on the same genomic adaptation as caddisflies do. Indeed, our analysis showed no evidence for expansion in mayfly OBPs that are thought to be related to sexual attraction (although another group of OBPs was seen expanded). Clearly, this observation remains mostly a speculation, which needs further confirmation from additional genomes of key taxa (e.g., other groups with short-lived adults, caddisflies that do form swarms, etc.), as well as laboratory experiments. It was not our intention to overly elaborate on this note.

Line 312 - I think you can do a better job selling the relevance of the study here. You present the first high-quality caddisfly genome, period. I would ditch the "welcome addition" part in the next sentence, it's a bit casual and implies you speak for the caddisfly community. (It is welcome though from this researcher's perspective!)

Response: We certainly feel encouraged by this comment and thank you very much for that. It is always rewarding to know that our work is being appreciated by the community. We have deleted "welcome addition". At the end of the "Concluding remarks", we also add a few words to emphasize the significance of our work: "The addition of a high-quality trichopteran genome has the potential to deliver insights into the genetic basis of diverse strategies of insects to adapt to divergent habitats and to uncover the genomic differences between aquatic and terrestrial lifestyles. In particular, the caddisfly genome may help to gain deeper understanding of the

evolution of the fascinating case-making behaviors and the robust aquatic silk of these underwater architects."

Can the authors provide any thoughts - speculation is quite alright - about why caddisfly genomes appear to be pretty variable in size? We now have four estimates and they range from S. tienmushanensis at ~0.45 Gb to L. lunatus at ~1.3 Gb, with two others in the middle of the range. How does this compare to, say, genome variation in Lepidoptera?

Response: This is a very important question. But we don't have a clear answer. Genome sizes may vary between and within insect groups, which seems to be a common theme for this diverse lineage. For instance, of the 78 Lepidoptera genomes reported till 2017 (Triant et al., 2017, PMID: 29602369), the genome sizes range from ~246-809M, which is roughly similar to what we have observed in caddisflies in terms of variation magnitudes. The evolution of genome sizes may be determined by various factors, e.g., ancestral states of the group, genome re-organization, selective pressure, etc. Unfortunately, it is unclear to us which one, or how many of these factors may have led to the variation in caddisflies. Comparative genomics between Lepidoptera and Trichoptera might be helpful in this regard. However, the available genomes for these sister groups are highly unbalanced in number, with many key caddisfly groups remain unknown (e.g., Hydroptilidae, Rhyacophilidae, Hydrobiosidae, etc.). With our *Stenopsyche* genome representing the first from the suborder Annulipalpia, we hope the increasing number of caddisfly genomes will eventually help to elucidate this question.

Reviewer #2: Review of GIGA-D-18-00136: "The genome of an underwater architect, the caddisfly Stenopsyche tienmushanensis Hwang (Insecta: Trichoptera)" – Luo et al. 2018

Overall the manuscript is well written and presented, and the work seems to be well performed. Certainly the final assembly results are very encouraging. I have no doubt that the genome of this caddisfly does add to the genome resource for insects and will be useful to other researchers in the future. I have only a couple of comments I would like to see addressed (and some minor suggestions). I do not envisage that either of these issues raised will significantly affect the results, but nonetheless I think they should be addressed here if nothing but to assure readers of the high quality of this work.

Response: We would like to express our gratitude to the positive feedback from the reviewer. We highly appreciate the efforts and the professional suggestions.

Comments:

- ***Potential coassembly of diverged homologous regions due to combined DNA from 2 wild-caught individuals and/or heterozygosity.*** *Presumably Stie1 and Stie2 individuals were unrelated (or at least their relationship unknown) – this could have implications on the genome assembly if DNA from multiple individuals is combined and subsequently coassembled: it could lead to duplicated contigs derived from diverged homologous regions from either individual. This is addition to the problem of coassembling heterozygous regions from the same individual, which may be an issue given the reasonably high level of heterozygosity indicated from the kmer plots (Fig S1). The authors may have already addressed these issues by removing contigs that are similar (>50% identity over >80% length) to other contigs (lines 144-148), but additional details will be useful to allow the reader to fully assess whether to be worried about this or not. For example, it would be useful to know how many contigs were excluded using the thresholds above, and what the distribution of % identity of these "redundant" contigs is? From the BUSCO analysis, it does not appear that duplication is a problem (only 3.6% BUSCO genes present in more than 1 copy) – so I don't expect uncollapsed heterozygosity to be a major issue. However, the authors should make more efforts to address this explicitly in the text to satisfy readers that this is the case. Note that tools such as Redundans (https://github.com/lpryszcz/redundans) are designed directly to deal with such issues, I would recommend the authors might use this here and in the future.*

Response: We appreciate the reviewer's constructive suggestions. Since we had to use two wild-caught individuals in PacBio sequencing in order to generate enough data, we agree that the co-assembling problem should be handled carefully. We have added a new section with the subheading "Heterozygous estimation" in the revised manuscript to address this issue.

To address the coassembly problem, we conducted analysis with LAST with different combinations of identity and overlap among all the contigs in order to devise an appropriate threshold for heterozygosity filtering. After checking the main genome assembly features (such as N50, reads mapping rates, BUSCO et al.), we chose the redundant threshold as ≥ 80% identity and ≥ 50% overlap. We removed 1,472 entire contigs in the step. We have added the details in the main TEXT.

We ran the Redundans program to double-check the heterozygosity issue, with the same thresholds that we used in LAST (80% identity and 50% overlap). The results from both methods were similar (a total of 1,474 redundant contigs identified, of which 1,471 were shared between the LAST and Redundans results). This new result confirmed that our initial genome assembly excluded most redundant contigs generated by heterozygosity either caused by allelic variations or pooling of multiple individuals. In the new version, we adopt a more liberal strategy by removing all redundant contigs identified by either LAST or Redundans. But if a redundant contig identified in the previous step was mapped with distinct full-length transcriptome

sequences and only aligned to other contigs at ≤ 90% identity, it was then considered as a real contig with expressed transcripts and was subsequently added back to the final genome assembly. Also we removed the short contigs (< 1,000 bp) from the genome assembly. In total, 1,498 redundant contigs were removed from the genome assembly in this step. BUSCO results based on the new assembly show that only 3.6% of the BUSCO single-copy genes have duplications, suggesting that heterozygous sequences are not a major issue for the new assembly.

- ***Potential contamination in the sequencing reads.*** *There appears to be no assessment of potential contamination from non-target organisms in the sequencing reads. Again, there is no indication from the assembly results that this is an issue, but it should be addressed before the assembled contigs are submitted to public repositories. I suggest the authors use Blobtools (https://drl.github.io/blobtools/) (or similar; there are quite a few tools for this now) to perform an analysis of taxonomic partitioning and final QC on their assembly scaffolds. The output plots should be included as a Supplementary figure. This analysis, which plots taxonomically annotated scaffolds based on %GC and coverage, is also useful to assess some of the points raised above regarding potential uncollapsed heterozygosity.*

Response: We followed this suggestion. After filtering out possible heterozygous contigs, we plotted the Taxon-Annotated GC-Coverage (TAGC) graph using Blobtools. We then used the following criteria to remove putative contaminations. As we have written in the modified TEXT "We screened for potential contamination in the genome assembly with Taxon-Annotated GC-Coverage (TAGC) plots using Blobtools (v1.0) [31]. To identify contaminated contigs, we followed the process outlined in Fu et al. 2017 [17]. In short, we marked a contig as a contaminant if it had all three of the following characteristics: (1) had a best hit to a reference sequence from non-Arthropoda, (2) had no mapping of full-length transcripts, and (3) contained no homologous insect genes from the Benchmarking Universal Single-Copy Orthologs (BUSCO v3.0, RRID:SCR_015008) [32]. Four contigs met these characteristics, and were subsequently removed from the assembly (TAGC plots for the final assembly shown in Fig. S6, Table S3).". This procedure removed four contigs from the final assembly, comprising a total of 1,594,341 bp. We have updated all relevant genome statistics throughout the manuscript.

Minor suggestions:

- *Line 25: "adaptations to the aquatic habitats"*

Response: We have deleted the word "the" in the new version.

-
- *Line 27: see above*

Response: We have deleted the word "the" in the new version.

- *Line 51: reference indicating the split between Leps and caddisflies?*

Response: References added.

- *Line 64: might want to explain more fully that "silk dope" is the liquid form of the silk before it is spun (I had to Google it)*

Response: We have modified the sentence as: "Unlike terrestrial silks, caddisworm silk is adapted to be spun from silk dope, the liquid form of the silk stored in silk glands, into tough viscoelastic fibers while fully submerged in water."

- *Line 94-96: this sentence doesn't quite make sense… also define "lotic"*

Response: We have modified the sentence as: "The larvae inhabit lotic environments (living in flowing waters) and are adapted to a wide range of micro-habitats, ...".

- *Line 96: typo "pullutants"→ pollutants*

Response: Done.

- *Line 101: remove colloquial "till"→ "until"*

Response: We have replaced "till" with "until" in the TEXT.

- *Line 104: remove "And"*

Response: Done.

- *Line 107: "Taxonomic identification was made using male morphology by XZ" – I don't understand what is meant by "XZ"? a reference to heterogametic sex?*

Response: "XZ" here stands for the author Xin Zhou. We have used his full name in the sentence in the modified version.

- *Line 114: Fig S1 – typo on Y-axes "Frequence"→ "Frequency"*

Response: Corrected.

- *Line 114: is there an independent estimate of genome size for this species?*

Response: The genome size is estimated using a *k*-mer profile analysis, which is a common practice in most genome sequencing projects. Unfortunately, we were not able to conduct an independent estimate using flow cytometry as the facility was not

available to the authors. Following the reviewer's suggestion, we confirmed the genome-size estimation using GenomeScope.

- *Line 114: how was the genome size estimated from the kmer distribution? This should be explained (perhaps in the S1 legend) as it might be useful to other readers.*

Response: Genome-size estimation based on *k*-mer distribution is a widely adopted method seen in many recent genome sequencing projects. In the *k*-mer profile approach, the genome is calculated by $G=K_{num}/K_{depth}$ (Lander & Waterman 1988, PMID: 3294162), where K_{num} is the total counts of *k*-mer and K_{depth} is the *k*-mer depth. We calculated the total number of *k*-mer reads, and determined K_{depth} from the distribution of *k*-mers. We have added relevant information in the TEXT and figure legend of Figure S2. We also estimated the genome size with the recommended program GenomeScope, and the relevant figures have been added in Figure S3.

- *Line 115: estimation of heterozygosity should also be better explained here. I think the authors have compared to a simulated A. thaliana genome, but it is not 100% clear. I would also point the authors to a useful tool at http://qb.cshl.edu/genomescope/ for genome size and heterozygosity estimation directly from kmer histograms*

Response: The *k*-mer profile can reflect the complexity of the genome: homozygous genomes have a simple Poisson profile while heterozygous genomes have a characteristic bimodal profile (Kajitani et al., 2014, PMC4120091). The results from the original Figure S2 indicated that the genome of *S. tienmushanensis* was heterozygous. To estimate the heterozygosity level, we used simulated genome data of *Arabidopsis thaliana* with different levels of heterozygosity and appropriate depth, and plotted the *k*-mer profiles. By comparing the *k*-mer profiles among simulated genome data with various pre-set heterozygosity and the empirical data from *S. tienmushanensis*, we could estimate the level of heterozygosity for *S. tienmushanensis* (Figure S2). This approach has been applied in many genome projects (Kajitani et al., 2014, PMC4120091, Glanzmann et al., 2016, PMC5142436).

In the new version of the manuscript, we also estimated the genome size and heterozygosity with the recommended program, GenomeScope. GenomeScope applies a mixture model of four evenly spaced negative binomial distributions to the *k*-mer profile to measure the relative abundances of heterozygous sequences instead of the simplified Poisson *k*-mer profile (Vurture et al., 2017, PMC5870704). We have added the new estimation results based on GenomeScope to the main TEXT and Figure S3.

- *Lines 121-130: this paragraph seems to jump around, from RNA to PacBio then back to RNA… suggest it's restructured to be clearer. See also comment below.*

Response: In the new version, we have re-organized the “Genome and transcriptome sequencing” and “Genome assembly and polishing” sections. Additionally, we have added two new sections, “Transcriptome analysis” and “Heterozygosity estimation”, in order to better explain these processes.

- *Lines 127-129: there is no indication what any of these programs (ICE, Arrow, LoRDEC) are actually doing to the data. This section needs more work to improve clarity*

Response: The programs ICE, Arrow, and LoRDEC are used in the transcriptome analysis (updated in the new section “Transcriptome analysis”). We followed the PacBio RS_IsoSeq protocol to generate non-redundant full-length transcripts with the raw data from PacBio. The details have been described in the TEXT and here. The analysis protocol includes three main steps (http://files.pacb.com/software/smrtanalysis/2.3.0/doc/smrtportal/help/Webhelp/CS_Prot_RS_IsoSeq.htm): 1) Classify: Extract insert reads from PacBio movies; then classify insert reads into chimeric or non-chimeric, and full-length or non-full-length reads. 2) Cluster: Predict de novo consensus isoforms of transcripts from the classified reads using the Iteratively Clustering and Error Correction (ICE) algorithm. Then polish the isoforms with PacBio Iso-Seq data using Arrow. We also used llumina RNA-seq data to correct/polish the PacBio-generated full-length transcript after the Clustering step. These Illumina RNA-seq data were only used in the polishing step. 3) Map: Retain only the transcripts that could be aligned to the intermediate genome assembly with GMAP. We produced 112,307 full-length transcripts and collapsed them to 22,347 consensus transcript isoforms.

Line 125: “excluding nearly half of the reads”?

Response: The relevant sentence has been removed in the new version. Corresponding contents have been re-organized in the new section “Transcriptome analysis”.

- *Line 148: how many contigs (and what span) were removed using this analysis? I would recommend the tool Redundans for this procedure (also uses LAST): https://github.com/lpryszcz/redundans*

Response: We removed 1,472 contigs with LAST. We have added the details in the main TEXT and Figure S5. We ran Redundans to double-check the heterozygosity issue, with the same thresholds that we used in LAST (80% identity and 50% overlap). The results were very similar (a total of 1,474 redundant contigs identified, of which 1,471 were shared between the LAST and Redundans results). This new result confirmed that our initial genome assembly excluded most redundant contigs either caused by allelic variations (heterozygosity) or pooling of multiple individuals. In the new version, we adopt a more liberal strategy by removing all redundant contigs identified by either

LAST or Redundans. However, if a redundant contig identified in the previous step was mapped with distinct full-length transcriptome sequences and only aligned to other contigs at ≤ 90% identity, it was then considered as a real contig with expressed transcripts and was subsequently added back to the final genome assembly. In total, 1,498 redundant contigs were removed from the genome assembly in this step. BUSCO results based on the new assembly show that only 3.6% of the BUSCO single-copy genes have duplications, suggesting that heterozygous sequences are not a major issue for the new assembly.

- *Line 177: "In total"*

Response: We have replaced "Totally" with "In total".

- *Line 213: it would be useful to perform BUSCO analysis on these predicted proteins. Quick and simple, add the results to Table 1.*

Response: We did the BUSCO analysis on the predicted proteins (compared against 1,658 insecta_odb9 genes). The results are: C:89.3% [S:81.1%, D:8.2%], F:3.8%, M:6.9%. The value of the BUSCO analysis on the predicted proteins were lower than the analysis on the nucleotides. Similar comparison pattern was shown in one beetle genome (complete BUSCO: 92.59% versus 86.40%, from Wu et al., 2018, PMC5905561). The different result was related to the gene model combination step with EVM. Although more BUSCO genes were predicted from the nucleotides with three different methods (*ab initio*, homology-based, RNA-seq assisted), not all of them would be retained by EVM. The EVM software combines *ab initio* gene predictions and protein and transcript alignments into weighted consensus gene structures. The weight value varies among different methods and are related to the alignment quality. So the value of complete BUSCOs on predicted proteins is lower than that on the nucleotides. For the other sequenced caddisfly genomes, we did not find the predicted proteins from Genbank or related literatures. So we did not include the BUSCO results on the predicted proteins in Table 1.

- *Line 224: "In total"*

Response: We have replaced "Totally" with "In total".

- *Line 242: see above*

Response: We have replaced "Totally" with "In total".

- *Line 260: typo "changs" → "changes"*

Response: Done.

- *Line 260: "showed a significant change in size"*

Response: We have modified the sentence as "Sixty-six gene families showed a significant change in size in *S. tienmushanensis*".

- *Line 289: "fleshed out" odd phrase – maybe 'explored', 'investigated'?*

Response: We have used "explored" as suggested.

- *Table 1: the genome of Limnephilus lunatus is >twice the size of S. tienmushanensis – is there any evidence this is a real difference?*

Response: This is a very important question. But we don't have a clear answer. Genome sizes may vary between and within insect groups, which seems to be a common theme for this diverse lineage. For instance, of the 78 Lepidoptera genomes reported up to 2017 (Triant et al., 2017, PMID: 29602369), the genome sizes range from ~246-809M, which is roughly similar to what we have observed in caddisflies in terms of variation magnitudes. The evolution of genome sizes may be determined by various factors, e.g., ancestral states of the group, genome re-organization, selective pressure, etc. Unfortunately, it is unclear to us which one, or how many of these factors may have led to the variation in caddisflies. Comparative genomics between Lepidoptera and Trichoptera might be helpful in this regard. However, the available genomes for these sister groups are highly unbalanced in number, with many key caddisfly groups remain unknown (e.g., Hydroptilidae, Rhyacophilidae, Hydrobiosidae, etc.). With our Stenopsyche genome representing the suborder Annulipalpia, we hope the increasing number of caddisfly genomes will eventually help to elucidate this question.

- *Table 1: the genome of Glyphotaelius pellucidus appears to be very poor and is barely worth comparing against.*

Response: In the revised Table 1, we have updated information on all sequenced caddisfly genomes. The five sequenced caddisflies so far provide an interesting record how genomics have developed in the study of Trichoptera. Therefore, even though the genome of *Glyphotaelius pellucidus* is of poor quality, we think it is still worth listing in the comparison.

- *Figure 1: I quite like the illustration but surely a picture of the actual animal is more informative? And no less beautiful I'm sure.*

Response: We would like to use the original illustration for Figure 1, and provide a photograph of the live *S. tienmushanensis* in Figure S1. As the photograph demonstrates, the picture barely shows any additional information about the insect. In fact, wing patterns are of very limited use in caddisfly taxonomy and rarely

informative for *Stenopsyche* spp. Perhaps more importantly, it was our intention to include a classic ink drawing of the insect in a genome paper to "marry" genomics to taxonomy. And we would still very much like to include this in the ms.

- *Figure 4 and 5: I'm not actually sure what the purpose of these phylogenies is – what do they show that is relevant or interesting? Perhaps this could be made clearer in the text.*

Response: Comparisons of gene expansion and contraction for *S. tienmushanensis* point out some potentially interesting genomic features of the sequenced caddisfly, which might be relevant to its adaptation to freshwater life. For instance, the P450 gene family, which is typically associated with metabolism of endogenous and exogenous chemicals in model systems, is among the obviously significantly expanded groups. The main purpose of Figure 4 was to show the expanded P450 group in *S. tienmushanensis*, and to infer the possible functions by comparing them to the P450 families in model species with known functions.

Similarly, the main purpose of Figure 5 was to investigate the phylogenetic relationship of the OBP genes in *S. tienmushanensis*, and to infer the possible functions of different OBPs by comparing them to the well-annotated OBPs from representative insect species (*D. melanogaster*, *T. castaneum* and *B. mori* were used here). Interestingly, the caddisfly possesses expansions in an OBP group that is most closely related to corresponding OBP genes in *D. melanogaster* known to be responsible for sexual attraction. To check the possible correlation between the OBPs and the aquatic lifestyle of freshwater insects, we also included the mayfly *Ephemera danica* in the analysis. These two insects both have relatively short life-span as adults, which leads to a speculation that they might share some common genomic features that help them find mates more efficiently. However, their life histories are distinctly different, where mayflies form mating swarms that may effectively increase their chances in finding mates. Not surprisingly, we did not find similar expanded OBP gene groups in the mayfly as the *S. tienmushanensis*.

We have modified the relevant sentences to make the part clearer. "For the species-specific paralogs of *S. tienmushanensis* revealed by the OrthoMCL analysis, GO enrichment (Fig. S4) revealed gene expansions of the odorant binding proteins (OBPs). A phylogeny of the OBPs from *S. tienmushanensis*, *D. melanogaster* [71], *T. castaneum* [72], and *B. mori* [73] (genome data sources shown in Table S8) indicated potential functional relevance of these expansions in the caddisfly genome. Of the expanded OBP gene groups in *S. tienmushanensis*, one was most closely related to OBP83a and OBP83b from *D. melanogaster* (Fig. 5), which are also known as OS-F and OS-E with putative roles in detection of volatile pheromones [71, 74, 75]; and another was most closely related to OBP84a from *D. melanogaster*, which is also known as PBPRP-4 (pheromone-binding protein related protein gene) [71]. These uniquely expanded OBPs in *S. tienmushanensis* may be an adaptive genomic feature associated with sex

attraction. Because most adult caddisflies do not feed due to reduced mouthpart structures, they are obliged to complete reproduction in a more efficient way, in the relatively short adult-stage. Therefore, the OBP expansions in *S. tienmushanensis* may reflect their adaptation in effective mate finding. It is worth noting that OBP expansion is probably not the only mechanism that helps to facilitate reproduction. We examined the mayfly (*Ephemera danica*) genome and did not find convergence on the OBPs. The PBP_GOBP family (PF01395 in Pfam), including pheromone binding proteins (PBP) and general odorant binding proteins (GOBP), was used to search for OBPs in the mayfly genome obtained from the i5K project [35] using HMMER (v3.1b2, RRID: SCR_005305) [76]. Although the mayflies are also known to have short life-span as adults, they may effectively increase their chances in finding mates by forming mating swarms. This behavioral adaptation may explain the discrepancy observed in the genomic features of their OBP genes."

- *Figure 4: the cytochrome P450 copies from the caddisfly appear to be paraphyletic here – is this potentially interesting?*

Response: Thanks for this careful remark. The paraphyly of the examined P450 may have been caused by varied reasons. For instance, the P450 genes included in our analysis came from 10 insect species (Fig. 4), but not all P450 genes were included. The incomplete sampling (both on gene choices and taxon inclusion) may have affected the topology of the gene phylogeny, in particular at the basal parts of the tree (the results on tree tips mostly hold true). Alternatively, some annotated P450 genes may not share homology, even though they show superficial similarity in nucleotide sequences. Without functional examination through laboratory experiments, most observations based on genomics would remain largely speculation. Unfortunately, these are difficult questions to answer, given our limited knowledge on many of the putative gene family members across organisms. Therefore, we are not in a good position to address the question further.
