# Supplementary material for: The genome of an underwater architect, the caddisfly Stenopsyche tienmushanensis Hwang (Insecta: Trichoptera)
Source: Gigascience. 2018 Nov 23;7(12):giy143. doi: 10.1093/gigascience/giy143 (PMC6302954; doi:10.1093/gigascience/giy143)
Supplement: giga-d-18-00136_revision_2.pdf [file giy143_giga-d-18-00136_revision_2.pdf]

GigaScience

The genome of an underwater architect, the caddisfly Stenopsyche tienmushanensis Hwang (Insecta: Trichoptera)

--Manuscript Draft--

**Manuscript Number:**	GIGA-D-18-00136R2
**Full Title:**	The genome of an underwater architect, the caddisfly Stenopsyche tienmushanensis Hwang (Insecta: Trichoptera)
**Article Type:**	Data Note
**Funding Information:**	National Natural Science Foundation of China (31772493) — Dr. Xin Zhou
	Chinese Universities Scientific Fund (2017QC114) — Dr. Xin Zhou
	Chinese Universities Scientific Fund (2018QC133) — Dr. Xin Zhou
**Abstract:**	Background: Caddisflies (Insecta: Trichoptera) are a highly adapted freshwater group of insects split from a common ancestor with Lepidoptera. They are the most diverse (> 16,000 species) of the strictly aquatic insect orders and are widely employed as bio-indicators in water quality assessment and monitoring. Among the numerous adaptations to aquatic habitats, caddisfly larvae use silk and materials from the environment (stones, sticks, leaf matter and etc.) to build composite structures such as fixed retreats and portable cases. Understanding how caddisflies have adapted to aquatic habitats will help explain the evolution and subsequent diversification of the group.<br><br>Findings: We sequenced a retreat-builder caddisfly Stenopsyche tienmushanensis Hwang and assembled a high-quality genome from both Illumina and PacBio sequencing. In total, 601.2 M Illumina reads (90.2 Gb), and 16.9 M PacBio subreads (89.0 Gb) were generated. The 451.5 Mb assembled genome has a contig N50 of 1.29 M, a longest contig of 4.76 Mb, and covers 97.65% of the 1,658 insect single-copy genes as assessed by Benchmarking Universal Single-Copy Orthologs (BUSCO). The genome comprises 36.76% repetitive elements. A total of 14,672 predicted protein-coding genes were identified. The genome revealed gene expansions in specific groups of the cytochrome P450 family and olfactory binding proteins, suggesting potential genomic features associated with pollutant tolerance and mate finding. In addition, the complete gene complex of the highly repetitive H-fibroin, the major protein component of caddisfly larval silk, was assembled.<br><br>Conclusions: We report the draft genome of Stenopsyche tienmushanensis, the highest quality caddisfly genome so far. The genome information will be an important resource for the study of caddisflies, and may shed light on the evolution of aquatic insects.
**Corresponding Author:**	Xin Zhou<br><br>CHINA
**Corresponding Author Secondary Information:**	
**Corresponding Author's Institution:**	
**Corresponding Author's Secondary Institution:**	
**First Author:**	Shiqi Luo
**First Author Secondary Information:**	
**Order of Authors:**	Shiqi Luo
	Min Tang

Powered by Editorial Manager® and ProduXion Manager® from Aries Systems Corporation

	Paul B. Frandsen
	Russell J. Stewart
	Xin Zhou
**Order of Authors Secondary Information:**	
**Response to Reviewers:**	Please see the attached file "response_to_reviewers_20181102.docx"
**Additional Information:**	
**Question**	**Response**
Are you submitting this manuscript to a special series or article collection?	No
**Experimental design and statistics**<br><br>Full details of the experimental design and statistical methods used should be given in the Methods section, as detailed in our Minimum Standards Reporting Checklist. Information essential to interpreting the data presented should be made available in the figure legends.<br><br>Have you included all the information requested in your manuscript?	Yes
**Resources**<br><br>A description of all resources used, including antibodies, cell lines, animals and software tools, with enough information to allow them to be uniquely identified, should be included in the Methods section. Authors are strongly encouraged to cite Research Resource Identifiers (RRIDs) for antibodies, model organisms and tools, where possible.<br><br>Have you included the information requested as detailed in our Minimum Standards Reporting Checklist?	Yes
**Availability of data and materials**<br><br>All datasets and code on which the conclusions of the paper rely must be either included in your submission or	Yes

deposited in publicly available repositories (where available and ethically appropriate), referencing such data using a unique identifier in the references and in the "Availability of Data and Materials" section of your manuscript.<br><br>Have you have met the above requirement as detailed in our Minimum Standards Reporting Checklist?	

Powered by Editorial Manager® and ProduXion Manager® from Aries Systems Corporation

Manuscript
Click here to access/download;Manuscript;Main_text_20181102.docx
Click here to view linked References

The genome of an underwater architect, the caddisfly *Stenopsyche tienmushanensis* Hwang (Insecta: Trichoptera)

Shiqi Luo[1], Min Tang[1], Paul B. Frandsen[2,3], Russell J. Stewart[4], and Xin Zhou[1*]

[1] Beijing Advanced Innovation Center for Food Nutrition and Human Health, College of Plant Protection, China Agricultural University, Beijing, China 100193

[2] Department of Plant and Wildlife Sciences, Brigham Young University, Provo, UT 84602 USA

[3] Data Science Lab, Smithsonian Institution, Washington, DC, 20002 USA

[4] Department of Biomedical Engineering, University of Utah, Salt Lake City, UT 84112 USA

Shiqi Luo: shiqi_luo@cau.edu.cn, http://orcid.org/0000-0002-0506-2230

Min Tang: mintang_bio@outlook.com, http://orcid.org/0000-0002-6021-7282

Paul B. Frandsen: paul_frandsen@byu.edu, http://orcid.org/0000-0002-4801-7579

Russell J. Stewart: russell.stewart@utah.edu, https://orcid.org/0000-0002-8389-8877

Xin Zhou: xinzhoucaddis@icloud.com, http://orcid.org/0000-0002-1407-7952

*Correspondence should be addressed to XZ (xinzhoucaddis@icloud.com)

Abstract

Background: Caddisflies (Insecta: Trichoptera) are a highly adapted freshwater group of insects split from a common ancestor with Lepidoptera. They are the most diverse (> 16,000 species) of the strictly aquatic insect orders and are widely employed as bio-indicators in water quality assessment and monitoring. Among the

numerous adaptations to aquatic habitats, caddisfly larvae use silk and materials from the environment (stones, sticks, leaf matter and etc.) to build composite structures such as fixed retreats and portable cases. Understanding how caddisflies have adapted to aquatic habitats will help explain the evolution and subsequent diversification of the group. **Findings:** We sequenced a retreat-builder caddisfly *Stenopsyche tienmushanensis* Hwang and assembled a high-quality genome from both Illumina and PacBio sequencing. In total, 601.2 M Illumina reads (90.2 Gb), and 16.9 M PacBio subreads (89.0 Gb) were generated. The 451.5 Mb assembled genome has a contig N50 of 1.29 M, a longest contig of 4.76 Mb, and covers 97.65% of the 1,658 insect single-copy genes as assessed by Benchmarking Universal Single-Copy Orthologs (BUSCO). The genome comprises 36.76% repetitive elements. A total of 14,672 predicted protein-coding genes were identified. The genome revealed gene expansions in specific groups of the cytochrome P450 family and olfactory binding proteins, suggesting potential genomic features associated with pollutant tolerance and mate finding. In addition, the complete gene complex of the highly repetitive H-fibroin, the major protein component of caddisfly larval silk, was assembled. **Conclusions:** We report the draft genome of *Stenopsyche tienmushanensis*, the highest quality caddisfly genome so far. The genome information will be an important resource for the study of caddisflies, and may shed light on the evolution of aquatic insects.

Keywords

caddisworm, caddisfly, aquatic insect, freshwater adaptation, silk, H-fibroin, PacBio

Data Description

Comprising >16,000 species and distributed worldwide except for Antarctica, caddisflies (Insecta: Trichoptera) are the most diverse of the strictly aquatic insect orders [1]. This highly adapted freshwater group split from a common ancestor with lepidopterans (moths and butterflies) more than 200 mya [2]. The transition between terrestrial and aquatic (freshwater) habitat has occurred multiple times independently within insects, with caddisflies representing one of the most recent examples [2]. Presumably, this radical transition required numerous adaptations in morphological, physiological and molecular traits. Understanding these adaptations will help explain how insects, in general, have evolved as one of the most successful and abundant class of animals on the planet, and how caddisflies, in particular, have adapted to a wide range of freshwater and marine habitats. Identifying the genomic underpinnings of the adaptive mechanisms of caddisflies will improve our knowledge of these thriving aquatic insects that, as major contributors to freshwater biodiversity, have been widely employed as bio-indicators in water quality assessment and monitoring [3].

In addition, caddisflies are of technological interest because, like their terrestrial moth and butterfly relatives, their larvae (caddisworms) spin silk. Unlike terrestrial silks, caddisworm silk is adapted to be spun into tough viscoelastic fibers while fully submerged in water. Caddisworms use their silk as an adhesive tape to construct a wide variety of composite structures using stones, sticks, leaf matter, and other sediment gathered from the benthos of freshwater rivers, lakes, streams, and marine tidal pools [4]. The larval architectures are suborder dependent, and include transportable tube cases that provide camouflage and physical protection (suborder Integripalpia), stationary fixed retreats with silk nets for capturing food (suborder Annulipalpia), and rigid silk cases for pupation (suborder “Spicipalpia”) [5]. The

distinct and varied deployments of their underwater silk are responsible, in large part, for the penetration of caddisworms into diverse aquatic habitats.

The major protein component of caddisworm silk is H-fibroin, a high molecular weight protein with a blocky, highly repetitive primary sequence. Caddisworm H-fibroins are extensively phosphorylated on repeating serine-rich motifs with the sequence $(_pSX)_n$, where pS is phosphoserine, X is a hydrophobic amino acid, and n=2-6 [6, 7]. The $(_pSX)_n$ motifs form divalent metal ion-stabilized β-domains that are responsible for the strength, toughness, and energy-dissipating self-recovery of caddisworm silk [8-10]. Currently, only incomplete caddisfly H-fibroin sequences are available through a GenBank search because it has not been possible to obtain full-length sequences from cDNAs [11, 12] , or to assemble the highly repetitive sequence *de novo* from short-read RNA-seq data [13] in the absence of long-read sequences.

As both an underwater adhesive and a tough fully hydrated metallofiber, caddisworm silk may provide new insights into the mimetic design of tough adhesive materials for use in aquatic environments. The high-quality draft genome of a caddisfly, which includes the full assembly of the H-fibroin gene complex, will be invaluable for further identifying and characterizing the enzymes [14] and structural protein components of caddisworm silks.

Sampling, taxonomy and sample preparation

The caddisfly *Stenopsyche tienmushanensis* Hwang 1957 (Fig. 1, Fig. S1, NCBI:txid1560151) is only found in China, representing one of the first caddisfly species described by Chinese taxonomists [15]. The distribution range of the species was recently reviewed and is confined to the Central China Region [16]. The larvae inhabit lotic environments (living in flowing waters) and are adapted to a wide range

of micro-habitats, from pristine creeks to disturbed streams, displaying tolerance to various levels of pollutants.

Adult caddisfly specimens were collected using a light trap by the Yongding River, at Yanchi Town in Beijing, China (altitude 292m, 40.03° N, 115.48° E) in 2017. This collecting site is the most northern record for the species. All specimens used in this study were collected at the same site on the same night. Specimens were kept alive on ice, flash frozen and transferred into a -80 °C freezer until extraction. Two female *Stenopsyche* adults (Stie1, Stie2) were used for genome sequencing because the quantity of DNA from a single specimen was not sufficient for PacBio sequencing. A third female individual (Stie3) was extracted for RNA and transcriptome sequencing. The guts were dissected and the remaining whole bodies were used for DNA and RNA extractions. DNA was extracted with SDS and proteinase K using the protocol developed by Hu *et al.* (https://www.protocols.io/view/dna-extraction-procedure-using-sds-jg4cjyw) [17]. Total RNA was extracted with TRIzol following the manufacturer's instructions (Thermo Fisher). Taxonomic identification was conducted by Xin Zhou using male morphology and confirmed by Cytochrome *c* Oxidase Subunit I (COI) barcodes.

Genome and transcriptome sequencing

Separate 400 bp insert-libraries were created from Stie1 and Stie2 DNA. We generated a total of ca. 270 millon 150 bp paired-end (PE) reads, 80.45 Gb in total, using the Illumina HiSeq X Ten sequencing platform at WuXi AppTec (Shanghai, China) (Table S1). We then combined and sequenced the remaining DNA from Stie1 and Stie2 using 12 PacBio Sequel SMRT cells 1M v2 (PacBio p/n101-008-000), with one movie of 600 min at the Genome Center of Nextomics (Wuhan, China). We

produced 78.72 Gb of subreads resulting in a mean subread length of 7.6 kb (Table S1).

We sequenced RNA samples using the Illumina HiSeq X Ten platform (insert-size of 180 bp, 150 PE reads) and the PacBio Sequel system (Iso-Seq, library size 0.5-6k), which produced 9.72 Gb and 10.31 Gb data, respectively (Table S1). We used the PacBio RNA sequences to obtain full-length transcripts and the Illumina RNA sequences to polish/correct sequencing errors for the PacBio reads (see "Transcriptome analysis" section).

For DNA reads sequenced with Illumina, we trimmed three and two bases at the 5' and 3' ends, respectively, using fastp (v 0.18.0) [18]. These termini showed higher fluctuation in per base quality scores, which were reported in the fastq files. For RNA reads from Illumina, we filtered the raw data using fastp with default parameters. For the PacBio data, the subreads (basecalls from a single pass of the insert DNA template) of poor quality were filtered out based on the signal to noise ratio with default parameters. This analytical step is part of the integrative data processing procedure and is performed automatically when the raw data are produced during sequencing.

Genome assembly and polishing

Before genome assembly, we estimated the genome size by *k*-mer analyses of the Illumina DNA data. The genome size is calculated using the formula: $G=K_{num}/K_{depth}$ [19], where K_{num} is the total counts of *k*-mer and K_{depth} is the *k*-mer depth. We generated a *k*-mer profile with Jellyfish (v2.1.3, RRID:SCR_005491) [20], which calculates the *k*-mer number and distribution. We then used two different models to generate estimates of genome size. The first method assumes a Poisson distribution for the *k*-mers. When multiple peaks are observed, the peak with lower *k*-mer frequencies is considered as the result of heterozygosity. The second method, which is

integrated into the program GenomeScope, uses a mixed negative binomial model, granting more flexibility in genome size estimation [21]. Using the distribution frequency of 17-mers (Fig. S2), the genome sizes were estimated as 453.2 Mb and 445.5 Mb for Stie1 and Stie2, respectively, when K_{depth} was calculated based on a Poisson distribution; while the genome sizes estimated in GenomeScope were 407.6 Mb and 406.8 Mb, respectively (Fig. S3).

De novo genome assembly conducted with Falcon on the PacBio data (v1.8.7, length_cutoff = 8 kb, length_cutoff_pr = 10 kb, max_diff = 60, max_cov = 75) [22] produced an initial assembly of 510.7 Mb, with a contig N50 of 1.16 Mb (Table S2). After the *de novo* assembly, a first round of genome polishing using PacBio subreads improved the accuracy of the assembly. BLASR in SMRTlink 4.0 (https://github.com/PacificBiosciences/SMRT-Link) mapped all subreads to the initial assembly with the following parameters: "--bestn 5 --minMatch 18 --minSubreadLength 1000 --minAlnLength 500 --minPctSimilarity 70 --minPctAccuracy 70 --hitPolicy randombest". Then Arrow (a fucntion of the SMRT analysis suite) [23] defined consensus sequences. Arrow reaches improved consensus when compared with the legacy Quiver algorithm and is based on a more straightforward hidden Markov model approach [23]. This analysis corrected 2,556,035 insertions, 519,440 deletions and 1,302,397 substitutions in the draft genome assembly.

To further correct errors in the PacBio only assembly, the Arrow-corrected genome was polished for two additional rounds with Illumina data using Pilon (v1.20, RRID:SCR_014731) [24]. First, we mapped reads from each individual, separately, to the Arrow-corrected assembly with bwa-mem using default parameters (Version 0.7.12-r1039, RRID:SCR_010910 [25]). Then, we used the output bam file from the

individual with higher coverage (Stie2, 99.20%, compared to 97.07% in Stie1) for the first round of polishing with Pilon (--mindepth 20), which corrected 87,535 insertions, 44,308 deletions and 46,678 substitutions. For the second round of polishing, we mapped all Illumina reads (from both Stie1 and Stie2) to the Pilon- and Arrow-corrected assembly with bwa-mem using default parameters. We then ran the resulting bam file through Pilon (--mindepth 20) again, producing an assembly of 512.7 Mb and correcting an additional 71,259 insertions, 123,506 deletions and 223,395 substitutions.

Transcriptome analysis

We identified full-length transcripts from the PacBio RNA-sequencing data following the PacBio IsoSeq analysis pipeline, which includes three steps: classifying, clustering and mapping. After filtering the low-quality subreads based on signal to noise ratio with default parameters, we used SMRT Link to convert raw sequences into a BAM file and retained reads of insert with high quality (minimal full pass: 1, minimal predicted accuracy: 0.8), producing circular consensus sequences (CCS), which were then classified into two classes: full-length reads (those that contained both the 5' primer and 3' primer with poly-As, 76.43% of all subreads) and non-full-length reads. Next, we conducted isoform clustering with full-length and non-full-length reads using the Iteratively Clustering and Error Correction (ICE) algorithm in the SMRT analysis software, followed by polishing using the Arrow function [23]. To correct sequencing errors from PacBio, we polished the consensus sequences with Illumina transcriptome sequences using LoRDEC [26] (v0.6, RRID: SCR_015814, with parameters: -k 19, -s 3), resulting in 272,511,198 bp of 118,776 full-length transcripts. Further, we retained only the transcripts that could be aligned to the intermediate genome assembly with GMAP (-n 1) [27], then collapsed them with the python script

collapse_isoforms_by_sam.py from SMRT Link package, producing a final set with 22,347 non-redundant transcriptome isoforms. The mean length of all resulting transcripts was 2,881 bp, ranging from 274 to 13,820 bp.

Heterozygosity estimation

We estimated the heterozygosity from the *k*-mer profile via a comparison to a series of simulated heterozygosities of a model genome (*Arabidopsis thaliana*) [28]. The estimated heterozygosities were 1.10% and 1.06% for Stie1 and Stie2, respectively (Fig. S4), which were similar to the results obtained from GenomeScope (1.08% and 1.05%, respectively, Fig. S3).

Although the estimated heterozygosity of *S. tienmushanensis* is within the normal range for non-model insects with a published genome, the pooling of DNA from two wild-caught caddisfly adults represents a potential source for inflated heterozygosity. To address this potential issue, we used LAST (v852, RRID:SCR_006119) [29] and Redundans (v 0.14a) [30] to identify redundant contigs in the intermediate assembly. Contigs of the corrected intermediate assembly were aligned against themselves using LAST (v852, RRID:SCR_006119) [29] and Redundans (v 0.14a) [30]. Those contigs with ≥ 50% of their length overlapping with others at a ≥ 80% identity were considered redundant; the shorter of the pair was removed from the genome assembly. As a result, a total of 1,472 and 1,474 contigs were identified as redundant by LAST and Redundans, respectively, with 1,471 contigs identified by both programs. The distribution of identity of the redundant contigs identified by LAST (Fig. S5) indicated that most had > 90% similarity and overlaps with other contigs. We then compared candidate redundant contigs identified by either LAST or Redundans with the full-length transcripts. If a particular candidate was mapped with distinct full-length transcript sequences and also aligned with other

contigs at ≤ 90% identity, it was considered a true contig with unique expressed transcripts and added back into the assembly. We additionally removed short contigs (< 1,000 bp) from the genome assembly. In total, 1,498 redundant contigs were removed from the genome assembly in this step.

We screened for potential contamination in the genome assembly with Taxon-Annotated GC-Coverage (TAGC) plots using Blobtools (v1.0) [31]. To identify contaminated contigs, we followed the process outlined by Fu et al. 2017 [17]. In short, we marked a contig as a contaminant if it had all three of the following characteristics: (1) had a best hit to a reference sequence from non-Arthropoda, (2) had no mapping of full-length transcripts, and (3) contained no homologous insect genes from the Benchmarking Universal Single-Copy Orthologs (BUSCO v3.0, RRID:SCR_015008) [32]. Four contigs met these characteristics, and were subsequently removed from the assembly (TAGC plots for the final assembly shown in Fig. S6, Table S3).

The final genome assembly of *S. tienmushanensis* is 451.5 Mb, with a contig N50 of 1.29 Mb and a longest contig of 4.76 Mb (Table S2). The size of the final assembly is very close to those estimated based on *k*-mer distributions using the Poisson distribution method (453.2 Mb and 445.5 Mb for Stie1 and Stie2, respectively), but larger than those estimated by GenomeScope (407.6 Mb and 406.8 Mb). This discrepancy may reflect the differences in the two algorithms, or it may imply possible redundant contigs that were not identified by our filtering procedures. The comparisons among the five available Trichoptera genome assemblies (including: *Glossosoma conforme* [33], *Glyphotaelius pellucidus* [34], *Limnephilus lunatus* provided by i5K [35] and *Sericostoma* sp. HW-2014 [36]) are shown in Table 1.

The completeness of the assembly was assessed using BUSCO (v3.0, RRID:SCR_015008) [32] and the insecta_odb9 gene set [37]. Overall, 97.65% of 1,658 single-copy genes were completely recovered in the full genome assembly, representing a significant improvement over existing caddisfly genomes (Table 1). The high completeness of the assembly is likely due to deep long-read sequencing, which enables the assembly of long and complex regions of the genome.

Repeat analysis and non-coding RNA (ncRNA) annotation

In total, we identified 91,564 simple sequence repeats (SSR, 4,217 with compound format) with the MIcroSAtellite identification tool (MISA, v1.0, RRID:SCR_010765) [38] using default parameters (see Table S4 for types of SSR). We identified 1,749,004 bp (0.39% of the genome size) of sequence as full-length long terminal repeat (LTR) transposons, using LTR_finder (v1.06, RRID: SCR_015247) [39] with the parameter "–a ps_scan". We also identified 3,579,704 tandem repeats, accounting for 0.79% of the genome size, using Tandem Repeats Finder (TRF, v4.09) [40] with the following parameters "Match = 2, Mismatch = 7, Delta = 7, PM = 80, PI = 10, Minscore = 20, MaxPeriod =2000". Next, we used RepeatModeler (v1.0.4, RRID:SCR_015027, http://www.repeatmasker.org) to generate a *de novo* repeat library from the genome (searching engine: rmblast, using default parameters), followed by RepeatMasker (v4.0.7, RRID:SCR_012954) [41] with parameters "-nolow –norna –q –no_is" to search for TEs from the known Repbase TE library (Repbase21.08) [42] and the *de novo* repeat library we built. In total, we annotated 46,773,887 bp (10.36%) and 156,642,282 bp (34.69%) from RepeatMasker with the Repbase TE library and the *de novo* repeat library, respectively. We also annotated 30,030,332 bp (6.65%) of TE sequences in the genome by similarity using the TE protein reference libraries in RepeatProteinMasker (v4.0.7, P < 0.0001,

RRID:SCR_012954) [41] using parameters "-noLowSimple -pvalue 0.0001". Overall, 36.76% of the genome were masked as repeats (Table 2, results from different programs in Table S5), with those classified as DNA transposons as the most abundant type (17.81% of the genome size).

We annotated rRNA using RNAmmer (v1.2) [43] with default parameters. In addition, we aligned our RNA-seq data to all caddisfly rRNA sequences available in Genbank using BLASTN (identity > 90%, mapping length for 18s and 28s rRNA > 400 bp). We predicted tRNA using tRNAscan-SE (v1.3.1, with default parameters) [44] and annotated snRNA and miRNA using Rfam 11.0 [45] and BLAST with default parameters. In total, we predicted 150 rRNAs (four 28S rRNA genes, one 18s rRNA gene and 145 5S rRNA genes), 644 tRNAs, 75 snRNAs and 89 miRNAs.

Gene prediction

We predicted gene models using three different strategies: *ab initio*, homology-based, and RNA-seq-assisted predictions. We chose one thousand non-redundant full-length transcripts, each of which contained more than one exon with translated amino acids at < 80% identity from each other, for parameter training in the *ab initio* prediction (AUGUSTUS v3.2.2, RRID:SCR_008417) [46] with other parameters as "--UTR = off --gff3 = on --genemodel = complete --strand = both --min_intron_len = 15". For homology-based gene prediction, we aligned the genome to insect proteins obtained from the uniref90 database [47] using TBLASTN with an E-value cutoff of 1e-5, and defined gene structures using GeneWise (v2.4.1, RRID:SCR 015054) [48] with parameters "-genesf -gff -sum -trev/tfor". For RNA-seq-assisted predictions, we used the Program to Assemble Spliced Alignment (PASA) (v2.0.2, RRID:SCR_014656) [49] with the parameter "--ALIGNERS BLAT" to align the transcriptomes to genome sequences with BLAT. We then predicted ORFs from the

resulting PASA gff file using Transdecoder (v5.0.2) [50] with default parameters. Finally, we used EvidenceModeler (EVM, v1.1.1, RRID:SCR_014659) to combine gene models from all three different methods (using the following weights for different types of evidence types: 3, 5 and 10 for *ab initio*, homology-based, and RNA-seq-assisted predictions, respectively), followed by PASA (run with the default parameters) to update the final results, including alternative splicing, UTRs, and additional genes missed but predicted by PASA (Table S6) [51]. All predicted genes were aligned with known transposons by Transposon PSI (http://transposonpsi.sourceforge.net/) to remove putative transposon sequences (E-value ≤ 1e-5). In total, 14,672 genes were annotated for *S. tienmushanensis*. Comparisons of the new Trichoptera annotation with four sequenced lepidopterans (*B. mori* : ASM15162 v.1 [52], *D. plexippus* v.3 [53], *H. melpomene* Hmel2.5 [54, 55] , *P. xylostella* DBM_FJ_V1.1 [56]) suggested that gene numbers and exon lengths were similar among all species (Table S7).

Functional annotation of protein-coding genes

Gene functions were assigned based on best match of the predicted proteins to SwissProt and TrEMBL [47] using BLASTP (with E-value ≤ 1e-5), and Kyoto Encyclopedia of Genes and Genomes (KEGG) databases using KAAS [57]. Of the 15,658 annotated proteins encoded by 14,672 genes, including those from alternative splicing, 10,441 (66.68%), 12,661 (80.86%) and 5,602 (35.78%) had significant hits with proteins catalogued in SwissProt, TrEMBL and KEGG, respectively. In total, 10,302 (65.79%) annotated proteins included motifs/domains identified by InterProScan (v5.21, RRID:SCR 005829) [58] when searched against InterPro databases. Of these, 7,842 genes were assigned to Gene Ontology (GO) [59] IDs with a corresponding InterPro entry (top 20 terms of GO pathway analysis shown in Fig.

S7). In summary, 12,805 annotated proteins encoded by 11,838 genes were assigned with at least one related function, accounting for 80.68% of the total identified genes in *S. tienmushanensis* (Fig. 2).

Gene orthology analysis and phylogenetic tree reconstruction

We constructed a phylogeny using genome-scale orthologous genes from 12 species, including *S. tienmushanensis*, 10 additional insects (*Acyrthosiphon pisum*, *Apis mellifera*, *Bombyx mori*, *Clunio marinus*, *Danaus plexippus*, *Drosophila melanogaster*, *Heliconius melpomene*, *Tribolium castaneum*, *Pediculus humanus* and *Plutella xylostella*) and a crustacean (*Daphnia pulex*) as the outgroup (see Table S8 for additional details). Gene orthology was identified using OrthoMCL (version v2.0.9, RRID: SCR_007839) [60] with default parameters. We excluded transcripts from alternative splicing and retained only the longest transcript for each gene. Orthologous proteins from the 12 species were aligned against each other using BLASTP (E-value ≤ 1e-5). Then we used the Markov Clustering Algorithm (MCL) to perform a graph clustering of protein orthologs from above. In total, 18,834 gene family clusters were identified, including 1,263 single-copy orthologous genes (Fig. 3).

We used these 1,263 orthologous single-copy genes from the 12 species to construct a phylogenetic tree. Multiple sequence alignments were conducted with MAFFT (version 7.058beta, RRID: SCR_011811) [61] with default parameters, and the protein alignment was transformed to a coding sequence (CDS) alignment. We used Gblocks (version 0.91b, with the parameter -b5=h) [62, 63] to filter out poorly aligned positions. The phylogenetic tree was constructed using RAxML (version v8.0.19, RRID: SCR_006086) [64] with the GTRGAMMA model and 100 bootstrap replicates. The divergence times among different lineages were estimated with the

MCMCTREE package from PAML (version 4.6, RRID: SCR_014932) [65], using parameters "clock = 2, RootAge ≤ 5.30, model = 7, BDparas = 110, kappa_gamma = 62, alpha_gamma = 11, rgene_gamma = 13.7, sigma2_gamma = 11.03". The phylogenetic tree (Fig. 3) confirmed that *S. tiemushanesis* was the sister lineage to Lepidoptera. The divergence time between *S. tienmushanensis* and the three representative Lepidoptera species was generally consistent with earlier results [2].

Based on the phylogeny, we conducted analyses on gene family expansions and contractions using CAFE (version 3.1) [66] with default parameters. Compared with sister taxa from Lepidoptera, *S. tienmushanensis* possessed a larger number of contracted gene families and lower number of expanded gene families from the common ancestor (Fig. 3). Among all expanded/contracted groups, 66 gene families showed a significant change in size in *S. tienmushanensis* ($P < 0.05$), in which 63 gene families were significantly expanded. These included cytochrome P450, HSP20, insect cuticle protein, and Histone-lysine N-methyltransferase SETMAR, which is related to DNA double-strand break repair [67, 68]. The expanded cytochrome P450 in the caddisfly was most closely related to the CYP9 family from *D. melanogaster* (Fig. 4), which are functional in the metabolism of insect hormones and in the breakdown of insecticides [69, 70]. We speculate that this expansion may play a role in the adaptation of *S. tienmushanensis* to a wide range of freshwaters with varied pollutants, although further investigations are needed to prove this hypothesis.

For the species-specific paralogs of *S. tienmushanensis* revealed by the OrthoMCL analysis, GO enrichment (Fig. S8) revealed gene expansions of the odorant binding proteins (OBPs). A phylogeny of the OBPs from *S. tienmushanensis*, *D. melanogaster* [71], *T. castaneum* [72], and *B. mori* [73] (genome data sources shown in Table S8) indicated potential functional relevance of these expansions in the

caddisfly genome. Of the expanded OBP gene groups in *S. tienmushanensis*, one was most closely related to OBP83a and OBP83b from *D. melanogaster* (Fig. 5), which are also known as OS-F and OS-E with putative roles in detection of volatile pheromones [71, 74, 75]; and another was most closely related to OBP84a from *D. melanogaster*, which is also known as PBPRP-4 (pheromone-binding protein related protein gene) [71]. These uniquely expanded OBPs in *S. tienmushanensis* may be an adaptive genomic feature associated with sex attraction. Because most adult caddisflies do not feed due to reduced mouthpart structures, they are obliged to complete reproduction in a more efficient way, in the relatively short adult-stage. Therefore, the OBP expansions in *S. tienmushanensis* may reflect their adaptation in effective mate finding. It is worth noting that OBP expansion is probably not the only mechanism that helps to facilitate reproduction. We examined the mayfly (*Ephemera danica*) genome and did not find convergence on the OBPs. The PBP_GOBP family (PF01395 in Pfam), including pheromone binding proteins (PBP) and general odorant binding proteins (GOBP), was used to search for OBPs in the mayfly genome obtained from the i5K project [35] using HMMER (v3.1b2, RRID: SCR_005305) [76] with default parameters. Although the mayflies are also known to have short life-span as adults, they may effectively increase their chances in finding mates by forming mating swarms. This behavioral adaptation may explain the discrepancy observed in the genomic features of their OBP genes when compared with the trichopteran genome.

H-fibroin gene analysis

Previous research on caddisworm silk has revealed that phosphorylation of serines in the H-fibroin protein and the incorporation of multivalent metal ions is responsible for its unique mechanical properties [6, 10]. However, while these features have been

revealed as important functional features of caddisworm silk, the genetic underpinnings of silk production have not been fully explored. For example, only partial sequences of the H-fibroin gene have been assembled in previously sequenced transcriptomes [13], presumably due to the inadequacy of short read technologies in resolving complex genomic features rich in repeats. Here, using long read PacBio sequencing, we report the first full assembly of the H-fibroin gene complex of a retreat-building caddisworm.

The genome assembly included a 21 kb region, which was identified as the complete H-fibroin gene complex, including two similarly sized H-fibroin genes separated by a short intergenic region. PacBio sequencing results show a coverage depth of > 100x with many reads spanning across large proportions of the gene range, including the intergenic region, assuring the validity of the assembly (Fig. 6a). The coding regions harbor multiple conserved tandem units with high similarity to a previously reported H-fibroin gene fragment from *Stenopsyche marmorata*, a retreat-making caddisfly from the same genus [7] (GenBank accession number BAM84281, 479 aa in length). The conserved units code typical short H-fibroin repeats, including GGX, SXSXSX and GPGX, with varied sequences and lengths (Fig. 6b). In addition, the identified region contained both non-repetitive N- and C-termini, homologous to the termini of *S. marmorata* H-fibroin [12] (Fig. 6c, 6d), further confirming complete assembly of the gene complex. Interestingly, the N-terminus was found at the beginning of the first gene and the C-terminus was found at the end of the second gene with the intergenic region occurring between the repetitive regions (Fig. 6a). Both genes encode proteins with the expected molecular mass (M_m) of H-fibroin, ~350 kg mol^{-1}. This gene structure had not been previously reported, and was only possible to determine with the full assembly using long-read sequencing. The

assembly of the complete H-fibroin region in our study provides a significant expansion over existing genetic resources on caddisfly H-fibroin genes, which will be important for studying caddisworm silk structure and adaptation to aquatic environments. For future studies, transcriptome and gene expression analysis from larval silk glands will help elucidate additional structural details of H-fibroin.

Concluding remarks

The genome presented here is the first high-quality draft genome of a retreat-building caddisfly. With a known diversity of over 16,000 species, caddisflies are important members of freshwater ecological communities and their species have been shown to be effective indicators of freshwater health [33, 77, 78]. There exists a host of researchers in freshwater biology and entomology whose research will be positively impacted by the availability of a high-quality draft genome.

In addition to the genome, we present a set of 14,672 annotated genes. This will enable large scale comparisons with existing genomes, especially those in Lepidoptera. While Trichoptera and Lepidoptera are reciprocally monophyletic and among the strongest supported ordinal level relationships within insects [2, 79], they have highly divergent life histories with Lepidoptera being primarily terrestrial, while the Trichoptera egg, larval, and pupal stages are entirely aquatic. The addition of a high-quality trichopteran genome has the potential to deliver insights into the genetic basis of diverse strategies of insects to adapt to divergent habitats and to uncover the genomic differences between aquatic and terrestrial lifestyles. In particular, the caddisfly genome may provide a deeper understanding of the evolution of the fascinating case-making behaviors and the underwater silk of these aquatic architects.

Availability of supporting data

All raw sequencing reads have been deposited in the Short Read Archive (SRA) under the project PRJNA436868. The raw sequencing reads, genome assembly, gene models and other supporting data are available via the *GigaScience* database, GigaDB[80].

Abbreviations

BUSCO: Benchmarking Universal Single-Copy Orthologs; CCS: circular consensus sequence; COI: Cytochrome c Oxidase Subunit I; GO: Gene Ontology; KEGG: Kyoto Encyclopedia of Genes and Genomes; ICE: Iteratively Clustering and Error Correction; LTR: long terminal repeat; MCL: Markov Clustering Algorithm; OBP: odorant binding protein; SMRT: single molecular real time; SRA: Short Read Archive; SSR: simple sequence repeats; TAGC: Taxon-Annotated GC-Coverage; TE: transposable elements; TRF: tandem repeat finder.

Competing interests

The authors declare that there are no competing interests.

Funding

XZ is supported by the National Science Foundation of China (31772493) , Beijing Advanced Innovation Center for Food Nutrition and Human Health, and the Chinese Universities Scientific Fund (2017QC114 and 2018QC133) through China Agricultural University.

Author contributions

XZ designed the study. SL, MT and PBF conducted genome analysis and assembly. XZ, SL, PBF and MT collected the specimens. PBF and RJS led analysis of the H-fibroin genes. All authors participated in writing and proofed the manuscript.

Acknowledgements

XZ thank Dr. Tingting Zhang from Shandong Agricultural University for her contribution in preparing the illustration of the caddisfly. Drs. Meng Yang, Ruixue Li,

Hui Zhang and Hua Peng from NextOmics provided important expertise and assistance in genome sequencing and analysis.

References

1. Morse JC. The Trichoptera world checklist. Zoosymposia 2011;**5**(1):372-80.
2. Misof B, Liu S, Meusemann K, et al. Phylogenomics resolves the timing and pattern of insect evolution. Science 2014;**346**(6210):763-7.
3. Resh VH and Unzicker JD. Water quality monitoring and aquatic organisms: the importance of species identification. J Water Pollut Control Fed 1975;**47**(1):9-19.
4. Holzenthal R, Blahnik R, Kjer K, et al. An update on the phylogeny of caddisflies (Trichoptera). In: *Proceedings of the 12th International Symposium on Trichoptera The Caddis Press, Columbus, Ohio* 2007, pp.143-53.
5. Holzenthal RW, Thomson RE and Ríos-Touma B. Order Trichoptera. Thorp and Covich's Freshwater Invertebrates (Fourth Edition). Elsevier; 2015. p. 965-1002.
6. Stewart RJ and Wang CS. Adaptation of caddisfly larval silks to aquatic habitats by phosphorylation of H-fibroin serines. Biomacromolecules 2010;**11**(4):969-74.
7. Ohkawa K, Miura Y, Nomura T, et al. Long-range periodic sequence of the cement/silk protein of *Stenopsyche marmorata*: purification and biochemical characterisation. Biofouling 2013;**29**(4):357-67.
8. Addison JB, Ashton NN, Weber WS, et al. β-Sheet nanocrystalline domains formed from phosphorylated serine-rich motifs in caddisfly larval silk: a solid state NMR and XRD study. Biomacromolecules 2013;**14**(4):1140-8.
9. Ashton NN and Stewart RJ. Self-recovering caddisfly silk: energy dissipating, Ca^{2+}-dependent, double dynamic network fibers. Soft Matter 2015;**11**(9):1667-76.
10. Ashton NN, Pan H and Stewart RJ. Connecting caddisworm silk structure and mechanical properties: combined infrared spectroscopy and mechanical analysis. Open Biol 2016;**6**(6):160067.
11. Yonemura N, Mita K, Tamura T, et al. Conservation of silk genes in Trichoptera and Lepidoptera. J Mol Evol 2009;**68**(6):641-53.
12. Wang Y, Sanai K, Wen H, et al. Characterization of unique heavy chain fibroin filaments spun underwater by the caddisfly *Stenopsyche marmorata* (Trichoptera; Stenopsychidae). Mol Biol Rep 2010;**37**(6):2885-92.
13. Ashton NN, Roe DR, Weiss RB, et al. Self-tensioning aquatic caddisfly silk: Ca^{2+}-dependent structure, strength, and load cycle hysteresis. Biomacromolecules 2013;**14**(10):3668-81.
14. Wang CS, Ashton NN, Weiss RB, et al. Peroxinectin catalyzed dityrosine crosslinking in the adhesive underwater silk of a casemaker caddisfly larvae, *Hysperophylax occidentalis*. Insect Biochem Mol Biol 2014;**54**:69-79.

15. Hwang CL. Descriptions of Chinese caddis flies (Trichoptera). Acta Zool sin 1958;**10**:279-85.
16. Xu JH, Wang BX and Sun CH. The *Stenopsyche simplex* species group from China with descriptions of three new species (Trichoptera: Stenopsychidae). Zootaxa 2014;**3785**(2):217-30.
17. Fu X, Li J, Tian Y, et al. Long-read sequence assembly of the firefly *Pyrocoelia pectoralis* genome. Gigascience 2017;**6**(12):1-7.
18. Chen S, Zhou Y, Chen Y, et al. fastp: an ultra-fast all-in-one FASTQ preprocessor. Bioinformatics 2018;**34**(17):i884-90.
19. Lander ES and Waterman MS. Genomic mapping by fingerprinting random clones: a mathematical analysis. Genomics 1988;**2**(3):231-9.
20. Marçais G and Kingsford C. A fast, lock-free approach for efficient parallel counting of occurrences of k-mers. Bioinformatics 2011;**27**(6):764-70.
21. Vurture GW, Sedlazeck FJ, Nattestad M, et al. GenomeScope: fast reference-free genome profiling from short reads. Bioinformatics 2017;**33**(14):2202-4.
22. Chin CS, Peluso P, Sedlazeck FJ, et al. Phased diploid genome assembly with single-molecule real-time sequencing. Nat Methods 2016;**13**(12):1050-4.
23. Chin CS, Alexander DH, Marks P, et al. Nonhybrid, finished microbial genome assemblies from long-read SMRT sequencing data. Nat Methods 2013;**10**(6):563-9.
24. Walker BJ, Abeel T, Shea T, et al. Pilon: an integrated tool for comprehensive microbial variant detection and genome assembly improvement. PLoS One 2014;**9**(11):e112963.
25. Li H and Durbin R. Fast and accurate short read alignment with Burrows–Wheeler transform. Bioinformatics 2009;**25**(14):1754-60.
26. Salmela L and Rivals E. LoRDEC: accurate and efficient long read error correction. Bioinformatics 2014;**30**(24):3506-14.
27. Wu TD and Watanabe CK. GMAP: a genomic mapping and alignment program for mRNA and EST sequences. Bioinformatics 2005;**21**(9):1859-75.
28. Kajitani R, Toshimoto K, Noguchi H, et al. Efficient *de novo* assembly of highly heterozygous genomes from whole-genome shotgun short reads. Genome Res 2014;**24**(8):1384-95.
29. Kiełbasa SM, Wan R, Sato K, et al. Adaptive seeds tame genomic sequence comparison. Genome Res 2011;**21**(3):487-93.
30. Pryszcz LP and Gabaldón T. Redundans: an assembly pipeline for highly heterozygous genomes. Nucleic Acids Res 2016;**44**(12):e113.
31. Kumar S, Jones M, Koutsovoulos G, et al. Blobology: exploring raw genome data for contaminants, symbionts and parasites using taxon-annotated GC-coverage plots. Front Genet 2013;**4**:237.
32. Simão FA, Waterhouse RM, Ioannidis P, et al. BUSCO: assessing genome assembly and annotation completeness with single-copy orthologs. Bioinformatics 2015;**31**(19):3210-2.

33. Weigand H, Weiss M, Cai H, et al. Fishing in troubled waters: Revealing genomic signatures of local adaptation in response to freshwater pollutants in two macroinvertebrates. Sci Total Environ 2018;**633**:875-91.

34. Ferguson L, Marlétaz F, Carter JM, et al. Ancient expansion of the Hox cluster in Lepidoptera generated four homeobox genes implicated in extra-embryonic tissue formation. PLoS Genet 2014;**10**(10):e1004698.

35. i5K Consortium. The i5K Initiative: advancing arthropod genomics for knowledge, human health, agriculture, and the environment. J Hered 2013;**104**(5):595-600.

36. Weigand H, Weiss M, Cai H, et al. Deciphering the origin of mito-nuclear discordance in two sibling caddisfly species. Mol Ecol 2017;**26**(20):5705-15.

37. Zdobnov EM, Tegenfeldt F, Kuznetsov D, et al. OrthoDB v9.1: cataloging evolutionary and functional annotations for animal, fungal, plant, archaeal, bacterial and viral orthologs. Nucleic Acids Res 2016;**45**(D1):D744-9.

38. Thiel T, Michalek W, Varshney R, et al. Exploiting EST databases for the development and characterization of gene-derived SSR-markers in barley (*Hordeum vulgare* L.). Theor Appl Genet 2003;**106**(3):411-22.

39. Xu Z and Wang H. LTR_FINDER: an efficient tool for the prediction of full-length LTR retrotransposons. Nucleic Acids Res 2007;**35**(suppl_2):W265-8.

40. Benson G. Tandem repeats finder: a program to analyze DNA sequences. Nucleic Acids Res 1999;**27**(2):573-80.

41. Tarailo-Graovac M and Chen N. Using RepeatMasker to identify repetitive elements in genomic sequences. Curr Protoc Bioinformatics 2009:4.10.1-4.

42. Kapitonov VV and Jurka J. A universal classification of eukaryotic transposable elements implemented in Repbase. Nat Rev Genet 2008;**9**(5):411-2.

43. Lagesen K, Hallin P, Rødland EA, et al. RNAmmer: consistent and rapid annotation of ribosomal RNA genes. Nucleic Acids Res 2007;**35**(9):3100-8.

44. Lowe TM and Eddy SR. tRNAscan-SE: a program for improved detection of transfer RNA genes in genomic sequence. Nucleic Acids Res 1997;**25**(5):955.

45. Burge SW, Daub J, Eberhardt R, et al. Rfam 11.0: 10 years of RNA families. Nucleic Acids Res 2012;**41**(D1):D226-32.

46. Fu H and Dooner HK. Intraspecific violation of genetic colinearity and its implications in maize. Proc Natl Acad Sci U S A 2002;**99**(14):9573-8.

47. UniProt Consortium. UniProt: a hub for protein information. Nucleic Acids Res 2015;**43**(D1):D204-12.

48. Birney E and Durbin R. Using GeneWise in the *Drosophila* annotation experiment. Genome Res 2000;**10**(4):547-8.

49. Haas BJ, Delcher AL, Mount SM, et al. Improving the *Arabidopsis* genome annotation using maximal transcript alignment assemblies. Nucleic Acids Res 2003;**31**(19):5654-66.

50. Haas BJ, Papanicolaou A, Yassour M, et al. *De novo* transcript sequence reconstruction from RNA-Seq: reference generation and analysis with Trinity. Nat Protoc 2013;**8**(8):1494.
51. Haas BJ, Salzberg SL, Zhu W, et al. Automated eukaryotic gene structure annotation using EVidenceModeler and the program to assemble spliced alignments. Genome Biol 2008;**9**(1):R7.
52. Duan J, Li R, Cheng D, et al. SilkDB v2. 0: a platform for silkworm (*Bombyx mori*) genome biology. Nucleic Acids Res 2009;**38**(suppl_1):D453-6.
53. Zhan S, Merlin C, Boore JL, et al. The monarch butterfly genome yields insights into long-distance migration. Cell 2011;**147**(5):1171-85.
54. Dasmahapatra KK, Walters JR, Briscoe AD, et al. Butterfly genome reveals promiscuous exchange of mimicry adaptations among species. Nature 2012;**487**(7405):94-8.
55. Davey JW, Chouteau M, Barker SL, et al. Major improvements to the *Heliconius melpomene* genome assembly used to confirm 10 chromosome fusion events in 6 million years of butterfly evolution. G3 2016;**6**(3):695-708.
56. You M, Yue Z, He W, et al. A heterozygous moth genome provides insights into herbivory and detoxification. Nat Genet 2013;**45**(2):220-5.
57. Moriya Y, Itoh M, Okuda S, et al. KAAS: an automatic genome annotation and pathway reconstruction server. Nucleic Acids Res 2007;**35**(suppl_2):W182-5.
58. Jones P, Binns D, Chang HY, et al. InterProScan 5: genome-scale protein function classification. Bioinformatics 2014;**30**(9):1236-40.
59. Ashburner M, Ball CA, Blake JA, et al. Gene ontology: tool for the unification of biology. Nat Genet 2000;**25**(1):25-9.
60. Li L, Stoeckert CJ and Roos DS. OrthoMCL: identification of ortholog groups for eukaryotic genomes. Genome Res 2003;**13**(9):2178-89.
61. Katoh K and Standley DM. MAFFT multiple sequence alignment software version 7: improvements in performance and usability. Mol Biol Evol 2013;**30**(4):772-80.
62. Talavera G and Castresana J. Improvement of phylogenies after removing divergent and ambiguously aligned blocks from protein sequence alignments. Syst Biol 2007;**56**(4):564-77.
63. Castresana J. Selection of conserved blocks from multiple alignments for their use in phylogenetic analysis. Mol Biol Evol 2000;**17**(4):540-52.
64. Stamatakis A. RAxML version 8: a tool for phylogenetic analysis and post-analysis of large phylogenies. Bioinformatics 2014;**30**(9):1312-3.
65. Yang Z. PAML 4: phylogenetic analysis by maximum likelihood. Mol Biol Evol 2007;**24**(8):1586-91.
66. De Bie T, Cristianini N, Demuth JP, et al. CAFE: a computational tool for the study of gene family evolution. Bioinformatics 2006;**22**(10):1269-71.
67. Fnu S, Williamson EA, De Haro LP, et al. Methylation of histone H3 lysine 36 enhances DNA repair by nonhomologous end-joining. Proc Natl Acad Sci U S A 2011;**108**(2):540-5.

68. Lee SH, Oshige M, Durant ST, et al. The SET domain protein Metnase mediates foreign DNA integration and links integration to nonhomologous end-joining repair. Proc Natl Acad Sci U S A 2005;**102**(50):18075-80.
69. Li X, Schuler MA and Berenbaum MR. Molecular mechanisms of metabolic resistance to synthetic and natural xenobiotics. Annu Rev Entomol 2007;**52**:231-53.
70. Feyereisen R. Insect CYP genes and P450 enzymes. Insect molecular biology and biochemistry. Elsevier; 2012. p. 236-316.
71. Hekmat-Scafe DS, Scafe CR, McKinney AJ, et al. Genome-wide analysis of the odorant-binding protein gene family in *Drosophila melanogaster*. Genome Res 2002;**12**(9):1357-69.
72. Dippel S, Oberhofer G, Kahnt J, et al. Tissue-specific transcriptomics, chromosomal localization, and phylogeny of chemosensory and odorant binding proteins from the red flour beetle *Tribolium castaneum* reveal subgroup specificities for olfaction or more general functions. BMC Genomics 2014;**15**:1141.
73. Gong DP, Zhang HJ, Zhao P, et al. The odorant binding protein gene family from the genome of silkworm, *Bombyx mori*. BMC Genomics 2009;**10**:332.
74. Pikielny CW, Hasan G, Rouyer F, et al. Members of a family of drosophila putative odorant-binding proteins are expressed in different subsets of olfactory hairs. Neuron 1994;**12**(1):35-49.
75. McKenna MP, Hekmat-Scafe DS, Gaines P, et al. Putative *Drosophila* pheromone-binding proteins expressed in a subregion of the olfactory system. J Biol Chem 1994;**269**(23):16340-7.
76. Eddy SR. Accelerated profile HMM searches. PLoS Comp Biol 2011;**7**(10):e1002195.
77. Jehamalar EE, Gloda D, Kiruba S, et al. Trichopterans as a bioindicators of a stream ecosystem. J Basic Applied Biol 2010;**4**:86-90.
78. Schmidt-Kloiber A, Neu PJ, Malicky M, et al. Aquatic biodiversity in Europe: a unique dataset on the distribution of Trichoptera species with important implications for conservation. Hydrobiologia 2017;**797**(1):11-27.
79. Kristensen NP. Phylogeny of endopterygote insects, the most successful lineage of living organisms. Eur J Entomol 1999;**96**:237-54.
80. Luo S; Tang M; Frandsen PB; Stewart RJ; Zhou X (2018): Supporting data for "The genome of an underwater architect, the caddisfly *Stenopsyche tienmushanensis* Hwang (Insecta: Trichoptera)" GigaScience Database. http://dx.doi.org/10.5524/100538

Figure legends

Figure 1: An illustration of the adult caddisfly *Stenopsyche tienmushanensis* in its typical habitat.

Figure 2: Functional gene annotations using four databases.

Figure 3: The phylogenetic tree and gene expansion/contraction of 12 arthropod taxa. Multiple-copy orthologs represent the gene groups present in all species with a gene number > 1 in at least one species. Species-specific paralogs represent genes uniquely present in only one species. Other types of orthologs represent the gene groups that are absent in some species and not species-specific paralogs. Numbers of expanded gene families are marked in green, while numbers of contracted gene families are marked in red. MRCA: most recent common ancestor. The number below MRCA is the total group numbers from the OrthoMCL analysis. Note that only some of the gene expansions/contractions are significant.

Figure 4: The phylogenetic relationship of the significantly expanded gene groups of cytochrome P450 family in ten insect species. The phylogeny was constructed using Maximum Likelihood, showing significant expansions in *S. tienmushanensis*. The bootstrap values are marked on the nodes.

Figure 5: The Maximum Likelihood tree of odorant-binding proteins (OBPs) in five insect species. The bootstrap values are marked on the nodes. The expanded OBP groups in *S. tienmushanensis* are most closely related to those potentially responsible for pheromone detection in *Drosophila*.

Figure 6: The H-fibroin gene complex in *S. tienmushanensis*. The sequences of H-fibroin gene fragments previously reported from *S. marmorata* are referred from [7, 12]. (a) The comparison of H-fibroin genes between *S. tienmushanensis* and *S. marmorata*. The depth of PacBio read coverage is shown in the line plot (smoothed by

a sliding window average of 25 bps). The H-fibroin alignment of one representative tandem repetitive unit, non-repetitive 5' end, and non-repetitive 3' end between *S. tienmushanensis* and *S. marmorata* was shown in panels (b-d). Identical amino acids in alignment between *S. tienmushanensis* and *S. marmorata* were marked in grey shadow. The start and end positions of the nucleotides were shown in the alignment of the repetitive units. Amino acids in the black box represent the typical motifs of short repeat unit. S.tie: H-fibroin gene complex in *S. tienmushanensis*; S.mar5/S.mar3: the 5'/3' end nucleotides of H-fibroin mRNA fragments in *S. marmorata*. The marked intron near the 5' end of the gene complex (position: 43-124) was inferred from the alignment between the non-repetitive 5' end between *S. tienmushanensis* and *S. marmorata*, positioned between sequences coding for the 14th and 15th amino acids of the N-terminus of the first predicted protein. The other marked intron (position: 10643-10729) was identified near the 5' end of the second predicted gene, positioned between the second and third position in the codon for the 14th amino acid of the second predicted protein.

Tables

Table 1 Comparison of genome assemblies among five caddisfly genomes

Species	*Stenopsyche tienmushanensis*	*Glossosoma conforme*	*Glyphotaelius pellucidus*	*Limnephilus lunatus*	*Sericostoma* sp. HW-2014
Platform	PacBio + Illumina	Illumina	Illumina	Illumina	Illumina
Aseembly accession	v1	ASM334726v1	-	Llun_2.0	ASM300347v1
Sequencing depth	153 × + 150 ×	53.0 ×	8.12 ×	80.1 ×	43.0 ×
Total length (bp)	451,494,475	604,293,666	757,289,448	1,369,180,260	1,015,727,762
Scaffold N50 (kb)	1,297	16.7	1.47	69.1	3.1
BUSCO (n=1,658)	C:97.6% (S:94.0%,D:3.6%) F:1.1% M:1.3%	C:85.2% (S:84.2%,D:1.0%) F:11.6% M:3.2%	C:22.3% (S:22.1%,D:0.2%) F:39.9% M:37.8%	C:86.7% (S:80.9%,D:5.8%) F:7.6% M:5.7%	C:37.4% (S:37.0%,D:0.4%) F:38.8% M:23.8%

The genome source: *G. conforme* [33], *G. pellucidus* [34], *L. lunatus* from i5K project [35], and *Sericostoma* sp. HW-2014 [36]. BUSCO annotation: C: complete BUSCOs; S: Complete and single-copy BUSCOs; D: Complete and duplicated BUSCOs; F: Fragmented BUSCOs; M: Missing BUSCOs.

Table 2 Summary of annotated repeats

Type	Combined TEs Length (bp)	% of genome
DNA	80,400,946	17.81
LINE	20,688,303	4.58
LTR	1,914,131	0.42
SINE	7,687	0.00
Other	15,267,487	3.38
Unknown	47,696,381	10.56
Total	165,974,935	36.76

“Other” represents a TE that is classified, but does not belong to one of our chosen classes. “Unknown” represents TE that could not be classified.

Click here to access/download;Figure;Figure_1_20180925.pdf

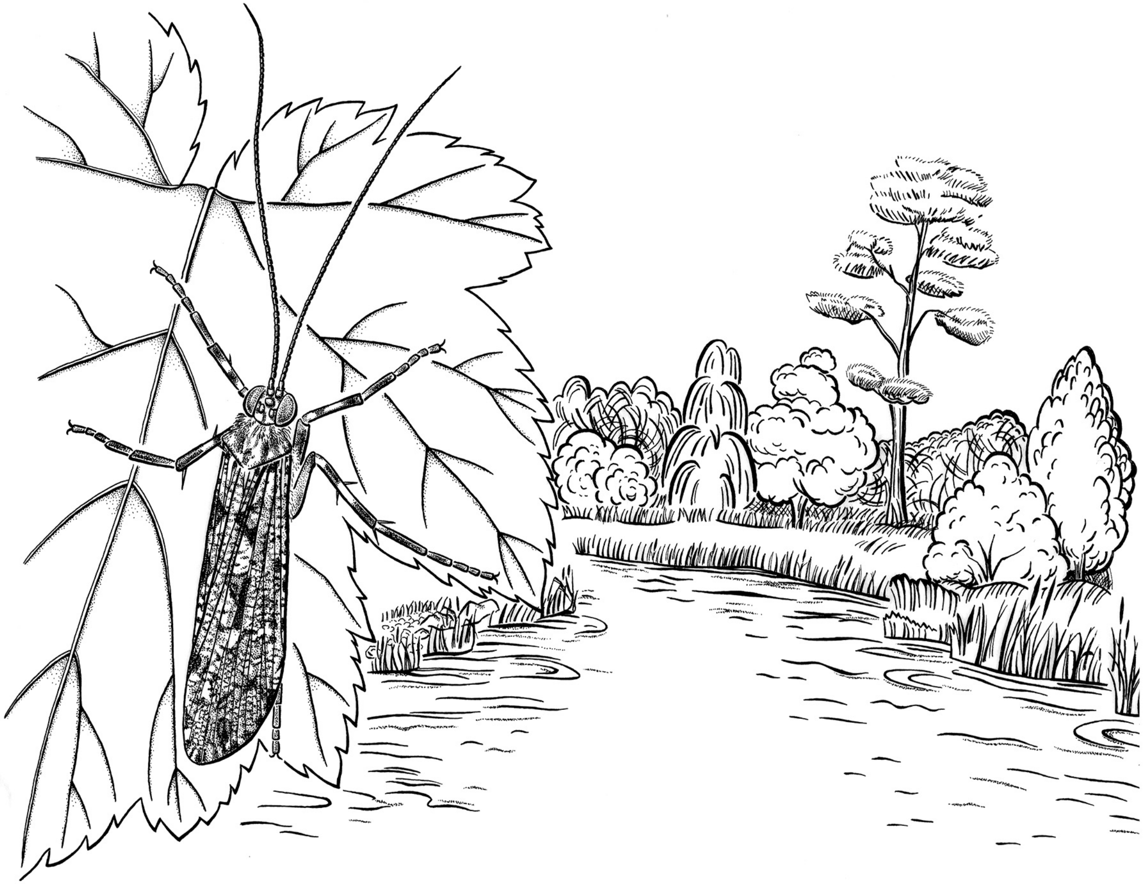

Figure 2

Click here to access/download;Figure;Figure_2_20180925.pdf

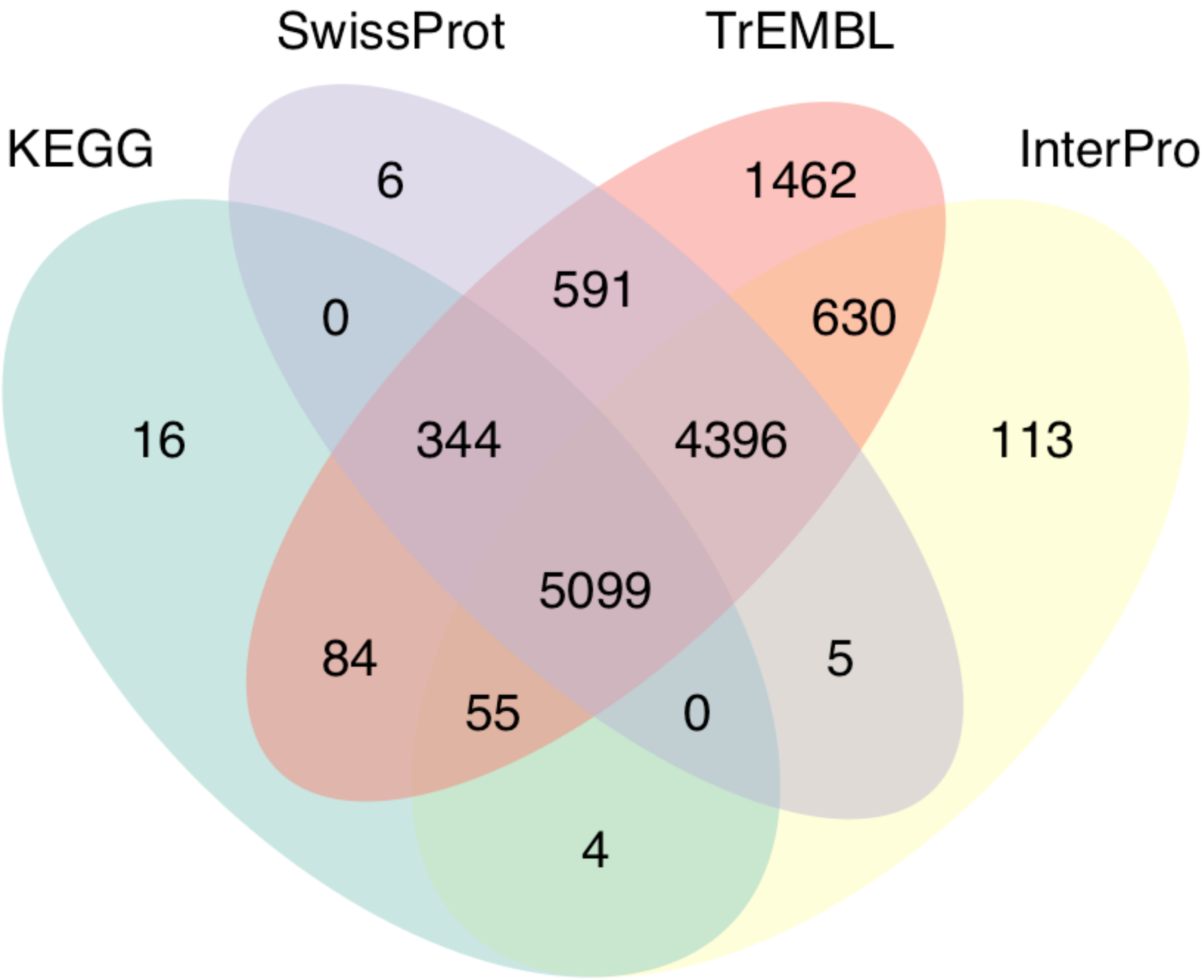

Figure 3 Click here to access/download;Figure;Figure_3_20180925.pdf

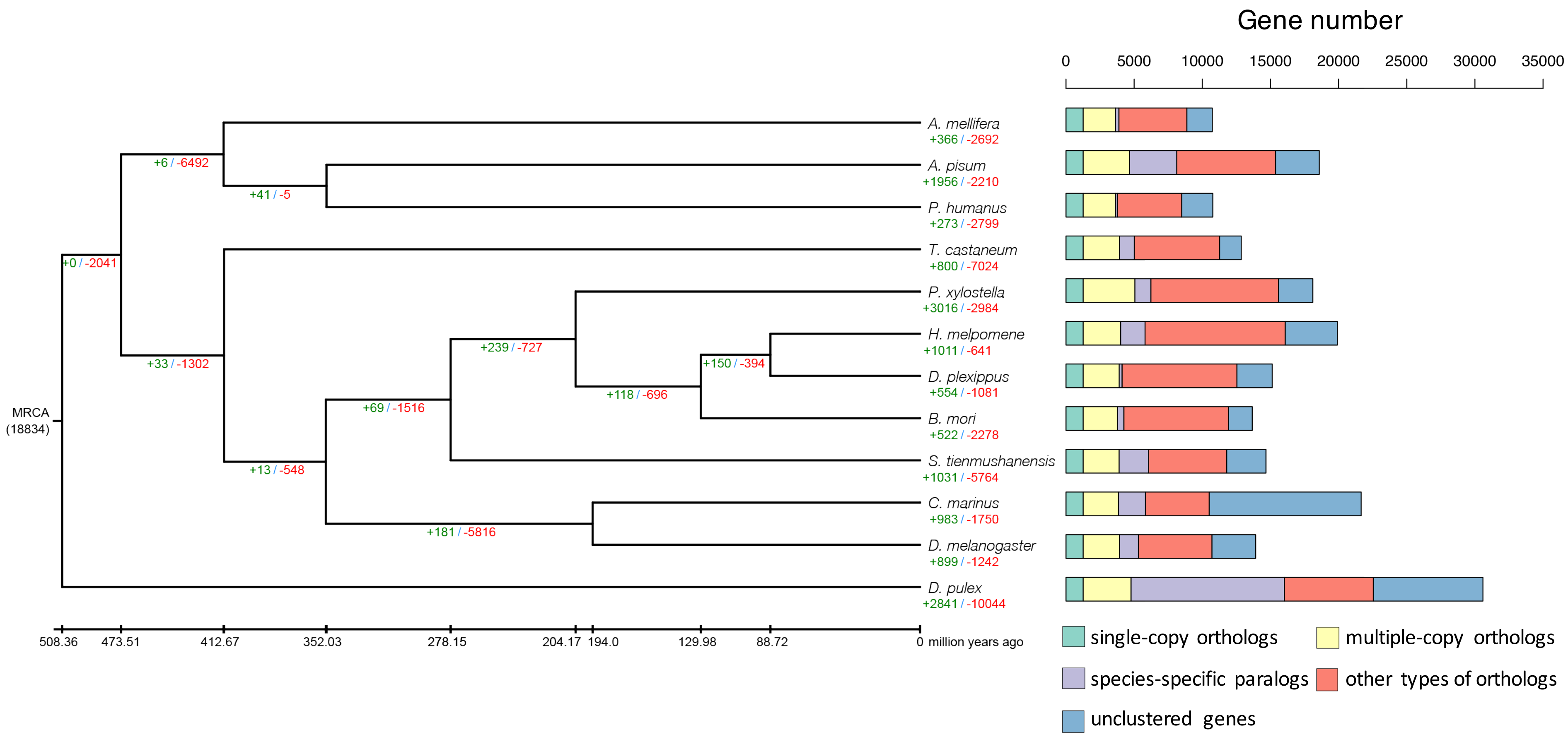

Figure 4

Click here to access/download;Figure;Figure_4_20180925.pdf

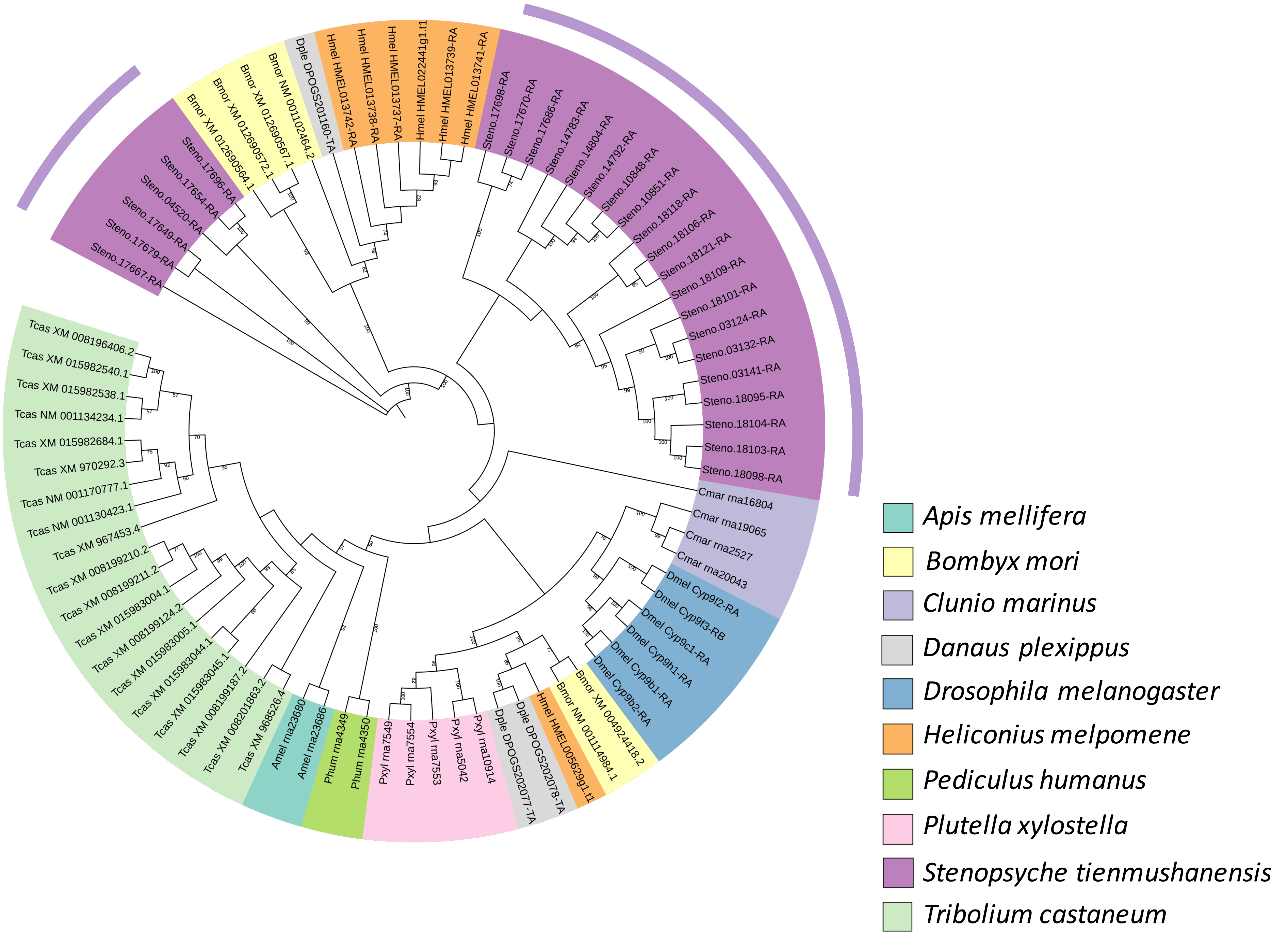

Figure 5 Click here to access/download;Figure;Figure_5_20180925.pdf

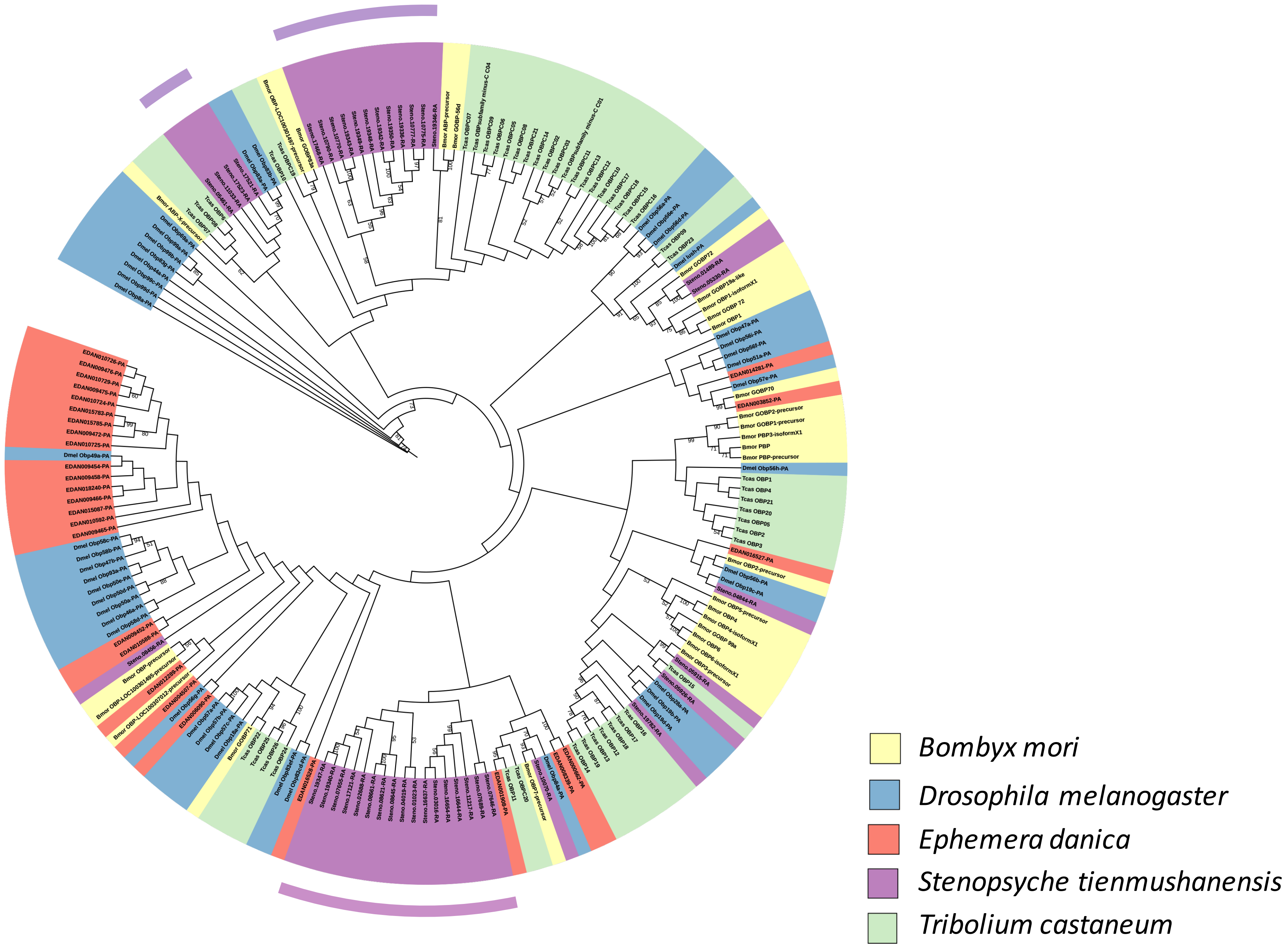

Figure 6 Click here to access/download;Figure;Figure_6_20180925.pdf

(a)

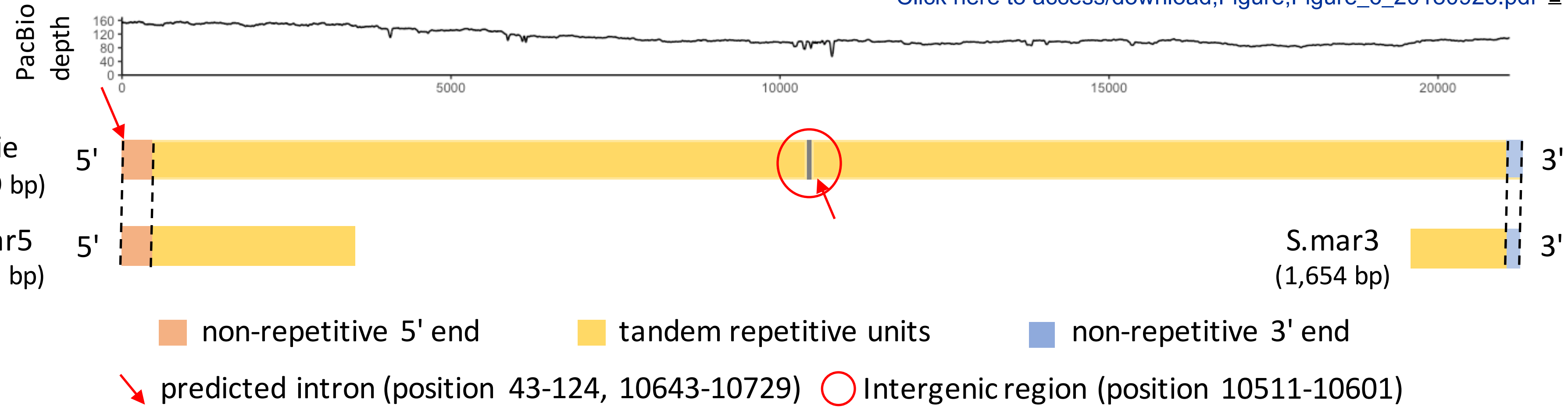

(b) tandem repetitive units

```
S.tie    7841 GPGYYGPGFVGPRRGKASASHSVSVETYVRAPIVRHFSRSGSVSIERPQYYYRPG 8005
S.mar3   525  GPGYYGPGFVGPRRGKASVSHSVSVETYVRAPIVRHFSRSGSVSIERPQYYYRPG 689

S.tie    8006 KISKSKSVSFEQVYVPPVVQHVKKSGSVSVERPQYFYRPGKVSVSRSYSYERVVR 8170
S.mar3   690  KISKSRSISVEQVYVPPVIQHVKKSGSVSVERPRYFYRPGKVSVSRSYSYERVVR 854

S.tie    8171 PARVFNRVSHSASVSVERPRHFVRPAVVARSGSFSAEGGWGRGSYGPGGLLG 8326
S.mar3   855  PARVFNRVSHSASVSVERPRHFVRPAVIARSASFSAEGGWGHGPYGHGGLLG 1010
```

(c) non-repetitive 5' end

```
S.tie   MRAVLFLILFCSLQIHLTGACNKPGKNVIGKLENFLSHGHLNPHVGLHEKILQGDDRIEANSRGDLIEKIISRKEILTD
S.mar5  MRAVLFLILFCSLQIHLTGACNKPGKNVIGKLENFLYHGHLNPHVGLHEKILQGDDRIEAKSRGDLIEKIISRKEILTD

S.tie   DDSESFSVSYSDESTEQIIKTITIVQEKPKHGGRAKEKIYEEEVVIKKVGEVPRDTKIAGCKSSEGIAGIGGIRRAWAS
S.mar5  DDSESFSVSYSDESTEQIIKTITIVQEKPKHGGRAKEKIYEEEVVIKKVGEVPRDTKIAGCKSSEGIAGIGGVRRAWAA
```

(d) non-repetitive 3' end

```
S.tie   DDDVVGGWAPSYGGSVVPFVDGGVSSVGGYGSIPQAVVYTRHPDPRTVRSPCKTSPFQLLINVGNSRKRAGNC
S.mar3  IFDVGAQLPSAYGGSVAPLVYGGVSSLGGYGSIPQAVVYTRHPDPRTVRSPCKSSPFHLLINVGNSRKRAGNC
```

Click here to access/download
Supplementary Material
SOM_20180925.docx

Table S3

Click here to access/download
Supplementary Material
Table S3.xlsx

response to reviewers Click here to access/download;Personal Cover;response_to_reviewers_20181102.docx

November 2nd, 2018
Dr. Scott C. Edmunds
Executive Editor
GigaScience

Dear Dr. Edmunds,

We are submitting the revised version of the manuscript "*The genome of an underwater architect, the caddisfly Stenopsyche tienmushanensis Hwang (Insecta: Trichoptera)*" by Luo *et al.* (GIGA-D-18-00136R1). Thank you very much for handling our previous submission and giving us the opportunity to revise this manuscript.

We appreciate the constructive comments from the reviewer. In the revised version, we have provided additional details in the analytical methods section of the TEXT, with additional information, including specific parameters, about the programs marked by the reviewer. We also polished the writing and corrected typos that we could identify, and made changes to address the concerns of the reviewer. We had two native English speakers review the TEXT and marked all major revisions in RED. At last, we included a point-to-point response to the reviewer. We believe the revised manuscript has been improved, thanks to the reviewer and editors of *GigaScience*. We hope this revision is satisfactory and we are looking forward to hearing further updates.

Sincerely yours,

Xin Zhou, on behalf of all authors
Beijing Advanced Innovation Center for Food Nutrition and Human Health &
College of Plant Protection,
China Agricultural University, Beijing 100193, China
Email: xinzhou@cau.edu.cn

Point-to-point response to the reviewers
(Line numbers mentioned in the response may not coincide with the original line numbers.)

Reviewer reports:
Reviewer #1: The revised manuscript is a substantial improvement over the previous version. However, my two overarching critiques of the previous version - (1) that the Methods were generally lacking in clarity (and specifics) and (2) that the writing would be improved by close copy-editing still stand. If the authors are worried about bogging down the manuscript with program details, they should consider a supplementary document that includes all of the programs used with the appropriate flags/usage details listed. A GitHub repository with the code to reproduce their analyses would be an even better solution.

That said, the authors have done a nice job improving the manuscript and I am confident further consideration of both clarity and reproducibility would improve it even more. Below, I have noted some specific, largely minor, critiques, many which reflect from my general criticisms above. My view that this resource is important to the field and will serve as a valuable resource for future studies of caddisfly evolution remains unchanged.

Response: We would like to thank you for the positive feedback and the careful scrutiny on our paper. We have provided additional details in the analytical methods as suggested to improve our work.

Specific comments:
Line 35 - Re-word this sentence. I would split apart the repeat percentage and number of genes into separate sentences as they don't make grammatical sense as is.

Response: We have modified the sentence to “The genome comprises 36.76% repetitive elements. A total of 14,672 predicted protein-coding genes were identified.”

Line 36 - The genome reveals this. Not the "genome sequences" or, perhaps they do, but that's an odd way to put it!

Response: We have deleted the word “sequences” in the sentence.

Lines 86-87 - Was the complete genome the key or the long-read sequences? I would argue the latter.

Response: We have modified the sentence to “… or to assemble the highly repetitive sequence *de novo* from short-read RNA-seq data [13] in the absence of long-read sequences.”

Line 110 - How were the "guts" removed? I assume dissection but this should be stated.

Response: We have modified the sentence to "The guts were dissected and the remaining whole bodies were used for DNA and RNA extractions."

Line 113 - The RNA extraction methods are not sufficient.

Response: Total RNA was extracted with TRIzol reagent using standard protocols provided by the manufacturer. We have specified it in the modified version: "Total RNA was extracted with TRIzol following the manufacturer's instructions (Thermo Fisher)."

Line 121 - How many SMRT cells were sequenced? I don't follow the "movie length" definition of sequencing effort. Or perhaps both the number of SMRT cells and the movie length should be reported if they are relevant to different aspects of the sequencing process.

Response: We used 12 SMRT cells in PacBio DNA sequencing, and the number of cells has been added in the TEXT. PacBio SMRT sequencing technology is single molecule real time sequencing (Rhoads & Au 2015, doi: 10.1016/j.gpb.2015.08.002). Four fluorescent-labeled nucleotides are added to the SMRT cell. The replication processes of single molecule template in zero-mode waveguides (ZMWs) of a SMRT cell are recorded by a "movie" of light pulses, and the pulses corresponding to each ZMW can be interpreted to be a sequence of bases (called a continuous long read). Our sequencing with PacBio Sequel produces sequencing movies of 600min (10 hr) in length. Choosing a movie time depends on many factors, such as the size of the insert, barcoding of samples, etc. Longer movies tend to result in increased read lengths and output, but there are diminishing returns. These technical details are part of the standard PacBio sequencing protocols and too lengthy to be reported in our manuscript.

Line 127 - "full length transcriptomes"? I assume this should be transcripts?

Response: We used "transcripts" as suggested.

Lines 130-133 - Is this all of the filtering that was performed? How did you determine those 5 bases needed to be trimmed? For PacBio, are "subreads" the same as reads? Why call them "subreads"? After some googling, it appears there is a bit of difference between reads/subreads. I think this should be stated briefly in the paper, your audience may not be aware of it.

Response: We adjusted the text to read: "For DNA reads sequenced with Illumina, we trimmed three and two bases at the 5' and 3' ends, respectively, using fastp (v 0.18.0) [18]. These termini showed higher fluctuation in per base quality scores, which were reported in the fastq files. For RNA reads from Illumina, we filtered the raw data using fastp with default parameters."

The generation of subreads in PacBio sequencing is described in Rhoads & Au (2015, doi: 10.1016/j.gpb.2015.08.002). Briefly, the SMRTbell, the template in PacBio

sequencing, is a closed, single-stranded circular DNA that is created by ligating hairpin adaptors to both ends of a target double-stranded DNA (dsDNA) molecule. Because the SMRTbell forms a closed circle, after the polymerase replicates one strand of the target dsDNA, it can continue incorporating bases of the adapter and then the other strand. If the lifetime of the polymerase is long enough, both strands can be sequenced multiple times (called ‘‘passes”) in a single continuous long read (CLR). The CLR can be split to multiple reads (called “subreads”) by recognizing and cutting out the adaptor sequences. We have added a brief definition for the term “subreads” in the modified version: “For the PacBio data, the subreads (basecalls from a single pass of the insert DNA template) of poor quality were filtered out based on the signal to noise ratio with default parameters. This analytical step is part of the integrative data processing procedure and is performed automatically when the raw data are produced during sequencing.” The detailed description of the sequencing procedure, however, is not included in the text.

Line 173 - "Transcriptomes" should be transcripts?

Response: We used “transcripts” as suggested.

Line 175 - "After filtering the low-quality reads" - how was this done?

Response: The filtering of the PacBio polymerase reads and subreads (both DNA and RNA sequencing) was based on the signal to noise ratio (SNR) with default parameters, and was conducted by programs (such as high quality region finder [HQRF]) pre-installed on the sequencing instrument. This filtering step is part of the integrative data processing procedure and is performed automatically when the raw data are produced during sequencing. We have addressed this issue in the “Genome and transcriptome sequencing” section. To avoid ambiguity, we have modified the sentence to “After filtering the low-quality subreads based on signal noise ratio with default parameters, we used SMRT Link…”

Lines 173-188 - There are many instances of "Program X was run..." then "We ran Program Y..." with very few details about these analyses. The authors need to provide more detail for how sequencing errors were corrected (Lines 181-182), how polishing was performed (Line 181), etc. And, to be clear, this comment applies beyond these ~15 lines to much of the paper. Running a program without specific details of how it was performed is a common theme here and perhaps the most significant weakness of the study in its current form.

Response: We have added additional details in the “Transcriptome analysis” section. After filtering low-quality subreads based on signal to noise ratio with default parameters, we defined high-quality reads of insert as having a minimal full pass of 1 and minimal prediction accuracy of 0.8, then produced circular consensus sequences (CCS). These steps are conducted with the PacBio IsoSeq analysis pipeline, and the details have been added in the new version.

The subsequent analysis is also based on the PacBio IsoSeq analysis pipeline, including three steps: classifying (command “pbtranscript classify” in the pipeline), clustering (command “pbtranscript cluster” in the pipeline) and mapping (polishing with LoRDEC and mapping with GMAP). Arrow (a function embedded with the PacBio IsoSeq analysis pipeline) is the successor of the Quiver algorithm, set with one parameter in the clustering step (“pbtranscript cluster --quiver”). LoRDEC is not part of the PacBio IsoSeq analysis pipeline, and we have added the key parameters for LoRDEC in the revised version.

Line 224 - You can merely reference Table S2. No need for "Statistics of the genome assembly in". There are a couple of other places where the writing can be tightened in this way as well.

Response: We have modified the references to tables in three places: (1) “Statistics of the genome assembly in Table S2” is modified to “Table S2” as suggested. (2) “80.45 Gb in total for the two samples, details shown in Table S1” is modified to “80.45 Gb in total for the two samples, Table S1”. (3) “details in Table S6” is modified to “Table S6”.

Line 240 and the rest of the paragraph - Again, lots of programs being used with few details. These would be very hard, if not impossible, analyses to replicate.

Response: We have added specific parameters in the corresponding section “Repeat analysis and non-coding RNA (ncRNA) annotation”, including those for programs LTR_finder, TRF, RepeatMasker, RepeatProteinMasker and RNAmmer.

Line 266 and the rest of the section - Same comment as above. GeneWise, ASA, BLAT, Transdecoder, EvidenceModeler - all ran with default parameters? Any changes?

Response: We have added parameters in programs used in the “Gene prediction” section, including AUGUSTUS, GeneWise, PASA, Transdecoder and EvidenceModeler. BLAT was used in PASA as one parameter “--ALIGNERS BLAT”.

Line 393 - "Mm" means "molecular mass", right? I would spell this out and then give the acronym (if needed).

Response: Yes. In the modified version, we have modified it to “molecular mass (M_m)” as suggested.
