# Supplementary material for: The genome of an underwater architect, the caddisfly Stenopsyche tienmushanensis Hwang (Insecta: Trichoptera)
Source: Gigascience. 2018 Nov 23;7(12):giy143. doi: 10.1093/gigascience/giy143 (PMC6302954; doi:10.1093/gigascience/giy143)
Supplement: reviewer_2_original_submission_(attachment).docx [file giy143_reviewer_2_original_submission_(attachment).docx]

**Review of GIGA-D-18-00136: “The genome of an underwater architect, the caddisfly Stenopsyche tienmushanensis Hwang (Insecta: Trichoptera)” – Luo et al. 2018**

Overall the manuscript is well written and presented, and the work seems to be well performed. Certainly the final assembly results are very encouraging. I have no doubt that the genome of this caddisfly does add to the genome resource for insects and will be useful to other researchers in the future. I have only a couple of comments I would like to see addressed (and some minor suggestions). I do not envisage that either of these issues raised will significantly affect the results, but nonetheless I think they should be addressed here if nothing but to assure readers of the high quality of this work.

Comments:

- **Potential coassembly of diverged homologous regions due to combined DNA from 2 wild-caught individuals and/or heterozygosity.** Presumably Stie1 and Stie2 individuals were unrelated (or at least their relationship unknown) – this could have implications on the genome assembly if DNA from multiple individuals is combined and subsequently coassembled: it could lead to duplicated contigs derived from diverged homologous regions from either individual. This is addition to the problem of coassembling heterozygous regions from the same individual, which may be an issue given the reasonably high level of heterozygosity indicated from the kmer plots (Fig S1). The authors may have already addressed these issues by removing contigs that are similar (>50% identity over >80% length) to other contigs (lines 144-148), but additional details will be useful to allow the reader to fully assess whether to be worried about this or not. For example, it would be useful to know how many contigs were excluded using the thresholds above, and what the distribution of % identity of these “redundant” contigs is? From the BUSCO analysis, it does not appear that duplication is a problem (only 3.6% BUSCO genes present in more than 1 copy) – so I don’t expect uncollapsed heterozygosity to be a major issue. However, the authors should make more efforts to address this explicitly in the text to satisfy readers that this is the case. Note that tools such as Redundans (<https://github.com/lpryszcz/redundans>) are designed directly to deal with such issues, I would recommend the authors might use this here and in the future.
- **Potential contamination in the sequencing reads.** There appears to be no assessment of potential contamination from non-target organisms in the sequencing reads. Again, there is no indication from the assembly results that this is an issue, but it should be addressed before the assembled contigs are submitted to public repositories. I suggest the authors use Blobtools (<https://drl.github.io/blobtools/>) (or similar; there are quite a few tools for this now) to perform an analysis of taxonomic partitioning and final QC on their assembly scaffolds. The output plots should be included as a Supplementary figure. This analysis, which plots taxonomically annotated scaffolds based on %GC and coverage, is also useful to assess some of the points raised above regarding potential uncollapsed heterozygosity.

Minor suggestions:

- Line 25: “adaptations to ~~the~~ aquatic habitats"
- Line 27: see above
- Line 51: reference indicating the split between Leps and caddisflies?
- Line 64: might want to explain more fully that “silk dope” is the liquid form of the silk before it is spun (I had to Google it)
- Line 94-96: this sentence doesn’t quite make sense… also define “lotic”
- Line 96: typo “pullutants” 🡪 pollutants
- Line 101: remove colloquial “till” 🡪 “until”
- Line 104: remove “And”
- Line 107: “Taxonomic identification was made using male morphology by XZ” – I don’t understand what is meant by “XZ”? a reference to heterogametic sex?
- Line 114: Fig S1 – typo on Y-axes “Frequence” 🡪 “Frequency”
- Line 114: is there an independent estimate of genome size for this species?
- Line 114: how was the genome size estimated from the kmer distribution? This should be explained (perhaps in the S1 legend) as it might be useful to other readers.
- Line 115: estimation of heterozygosity should also be better explained here. I think the authors have compared to a simulated A. thaliana genome, but it is not 100% clear. I would also point the authors to a useful tool at <http://qb.cshl.edu/genomescope/> for genome size and heterozygosity estimation directly from kmer histograms
- Lines 121-130: this paragraph seems to jump around, from RNA to PacBio then back to RNA… suggest it’s restructured to be clearer. See also comment below.
- Lines 127-129: there is no indication what any of these programs (ICE, Arrow, LoRDEC) are actually doing to the data. This section needs more work to improve clarity
- Line 125: “excluding nearly half **of** **the** reads”?
- Line 148: how many contigs (and what span) were removed using this analysis? I would recommend the tool Redundans for this procedure (also uses LAST): <https://github.com/lpryszcz/redundans>
- Line 177: “In total”
- Line 213: it would be useful to perform BUSCO analysis on these predicted proteins. Quick and simple, add the results to Table 1.
- Line 224: “In total”
- Line 242: see above
- Line 260: typo “changs” 🡪 “changes”
- Line 260: “showed **a** significant **change in size**”
- Line 289: “fleshed out” odd phrase – maybe ‘explored’, ‘investigated’?
- Table 1: the genome of *Limnephilus lunatus* is >twice the size of *S. tienmushanensis* – is there any evidence this is a real difference?
- Table 1: the genome of *Glyphotaelius pellucidus* appears to be very poor and is barely worth comparing against.
- Figure 1: I quite like the illustration but surely a picture of the actual animal is more informative? And no less beautiful I’m sure.
- Figure 4 and 5: I’m not actually sure what the purpose of these phylogenies is – what do they show that is relevant or interesting? Perhaps this could be made clearer in the text.
- Figure 4: the cytochrome P450 copies from the caddisfly appear to be paraphyletic here – is this potentially interesting?
