# Supplementary material for: The genome of an underwater architect, the caddisfly Stenopsyche tienmushanensis Hwang (Insecta: Trichoptera)
Source: Gigascience. 2018 Nov 23;7(12):giy143. doi: 10.1093/gigascience/giy143 (PMC6302954; doi:10.1093/gigascience/giy143)
Supplement: Supplemental Files [file giy143_supplemental_files.zip › SOM_20180925.docx]

**
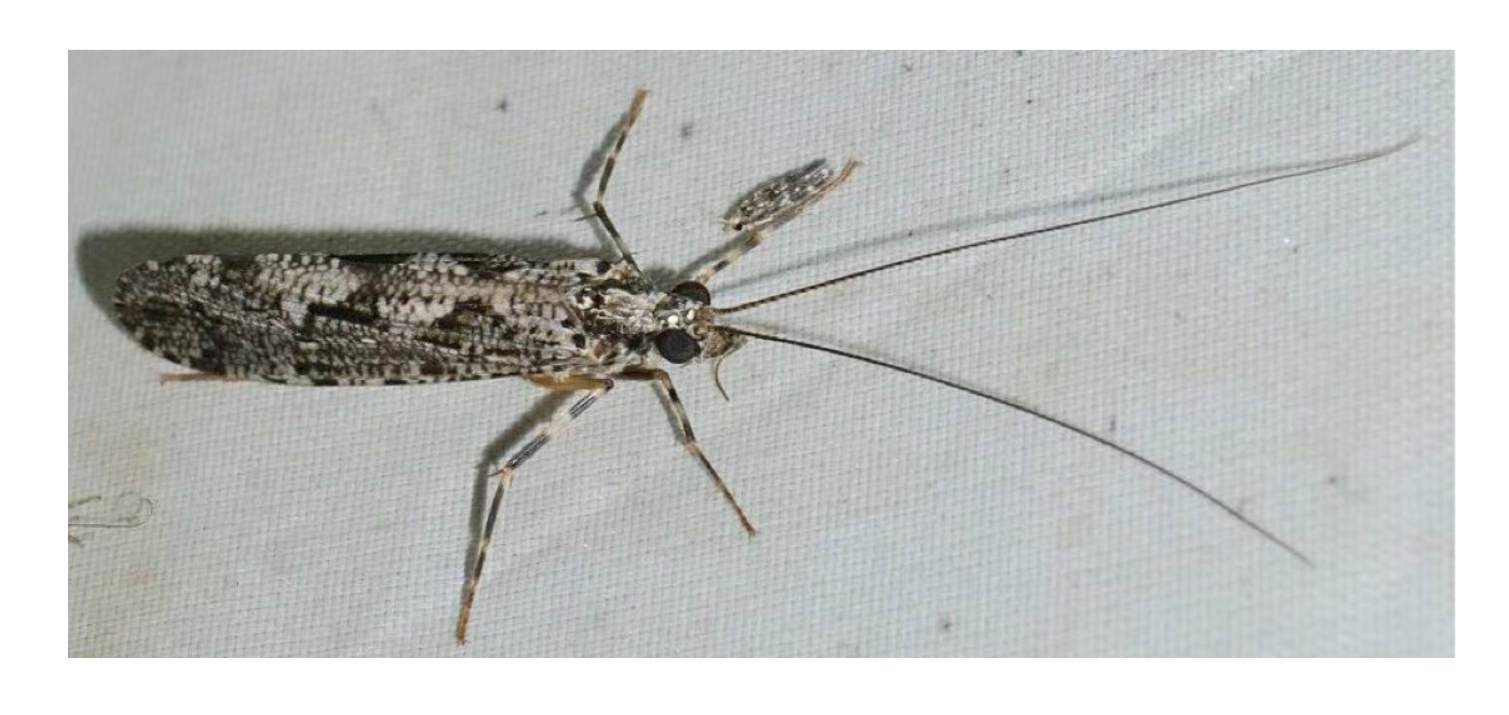
**

**Figure S1. An adult *Stenopsyche tienmushanensis* attracted to light trap (Photo credit: Mr. Jiahui Hu, China Agricultural University).** By comparison, an adult Hydroptilidae is shown resting next to the left foreleg of *Stenopsyche*.

**
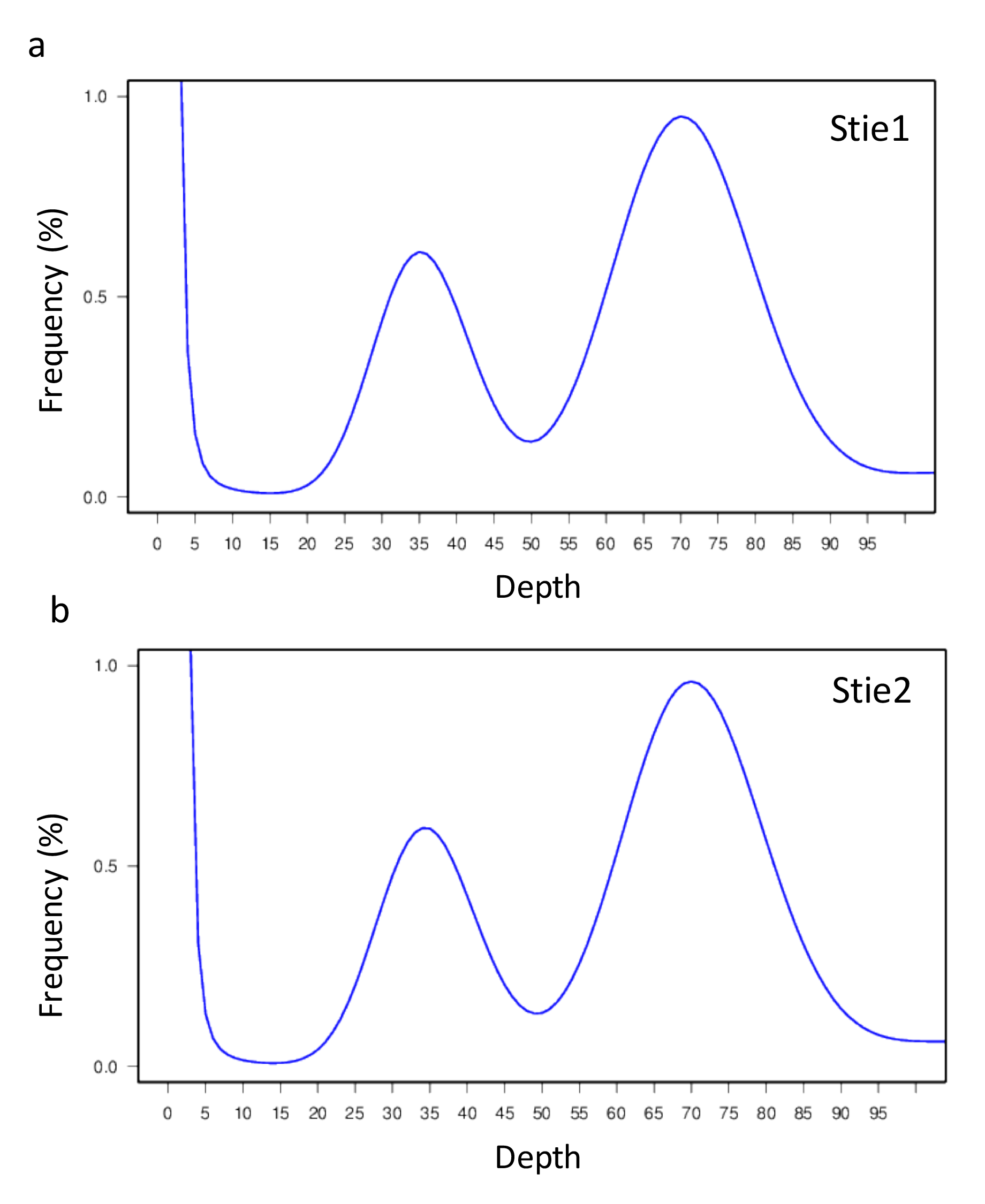
**

**Figure S2. Distributions of Illumina 17-mers for samples Stie1 (a) and Stie2 (b).** The first peak on the left (depth=35) is a heterozygous peak, the second peak is the main peak (depth=70).

**
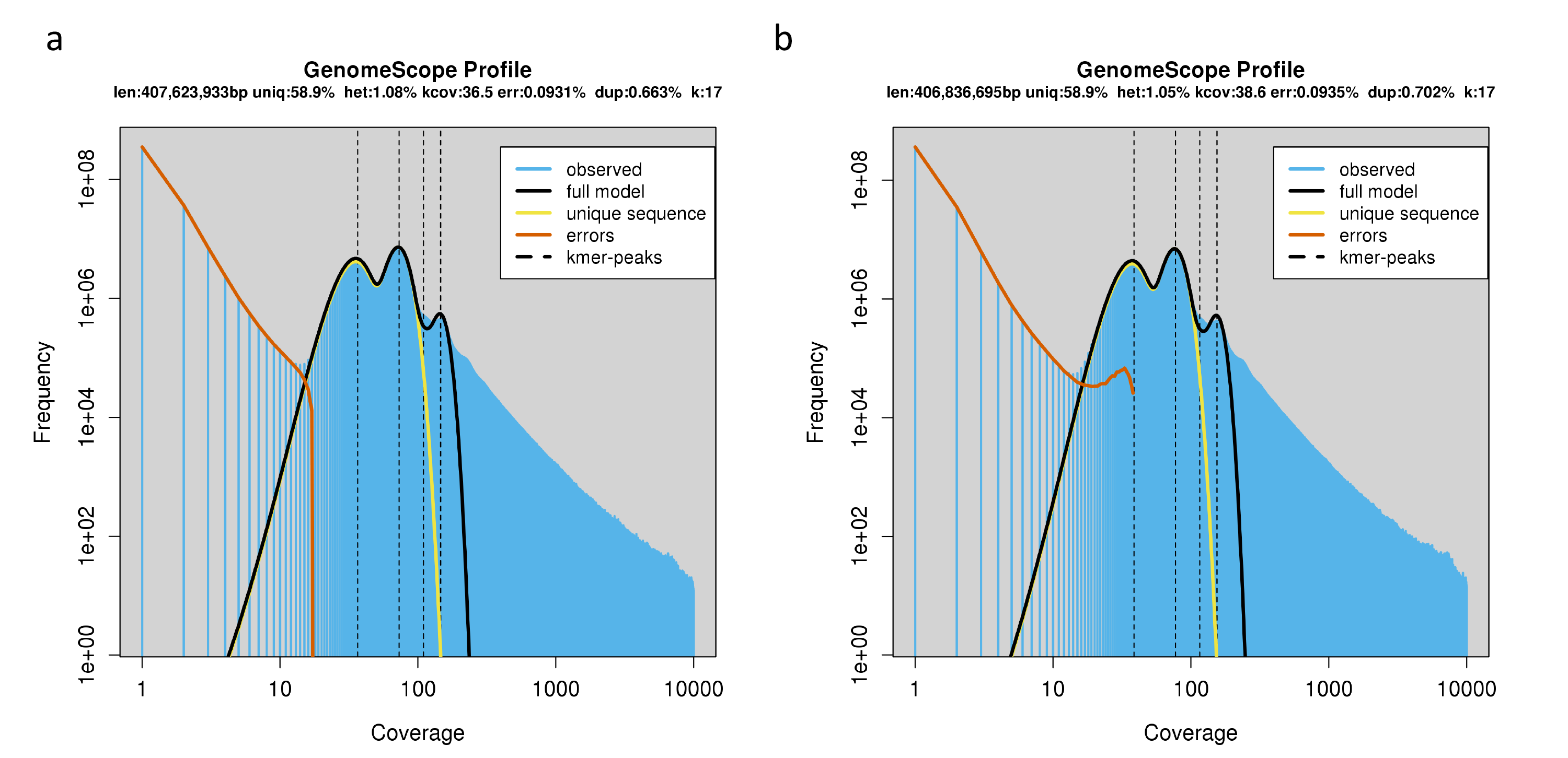
**

**Figure S3. GenomeScope 17-mer profile plots for Stie1 (a) and Stie2 (b), showing the fitting of the GenomeScope model (black) to the observed k-mer frequencies (blue).**

**
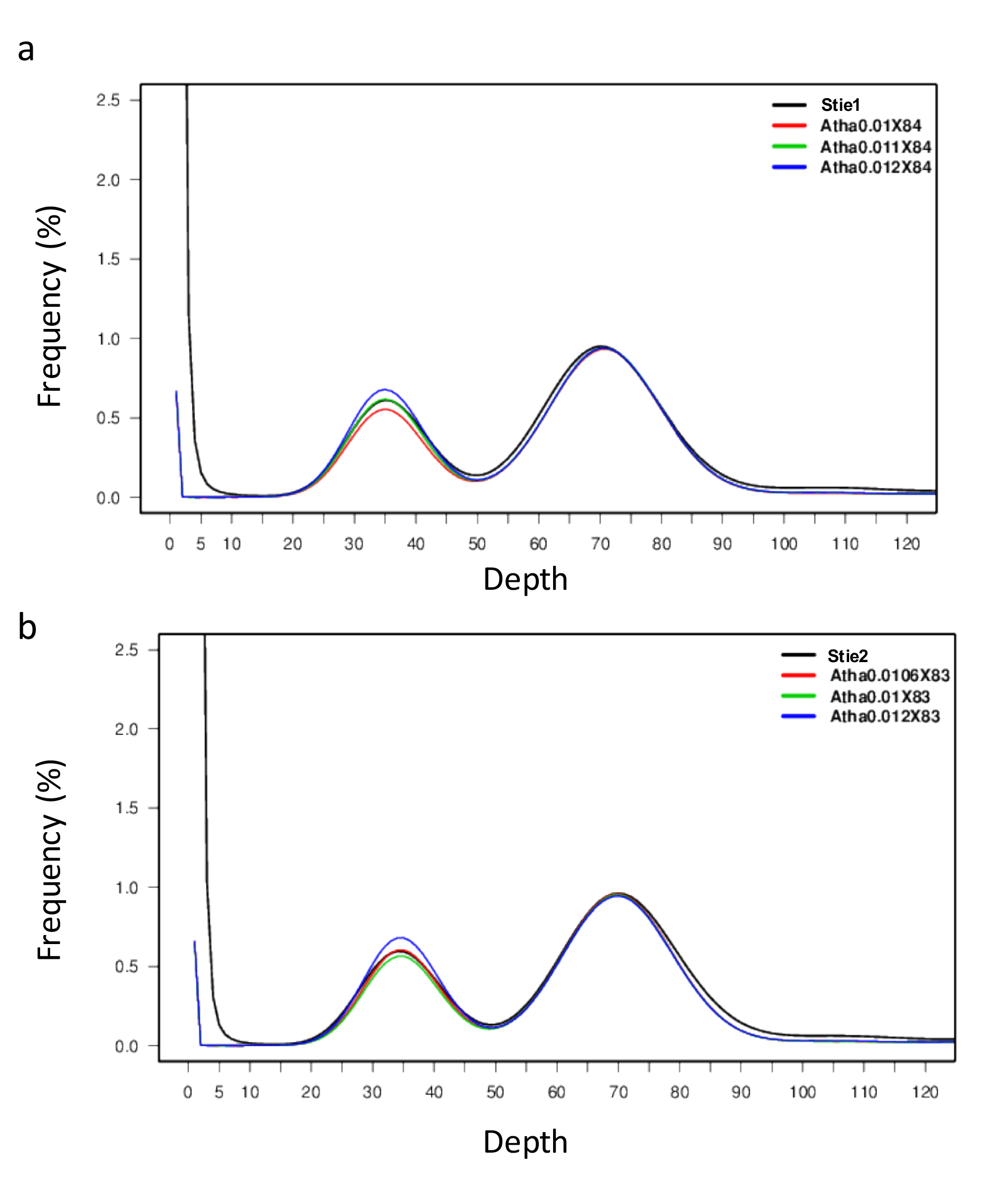
**

**Figure S4. Comparison of 17-mer depth distributions of samples Stie1 (a) and Stie2 (b) with a series of simulated heterozygosities of a model genome (*Arabidopsis thaliana*).** The simulated genome data of *A. thaliana* with different heterozygosity (number before “X” in the key) and appropriate depth (number after “X” in the key) was used.

**
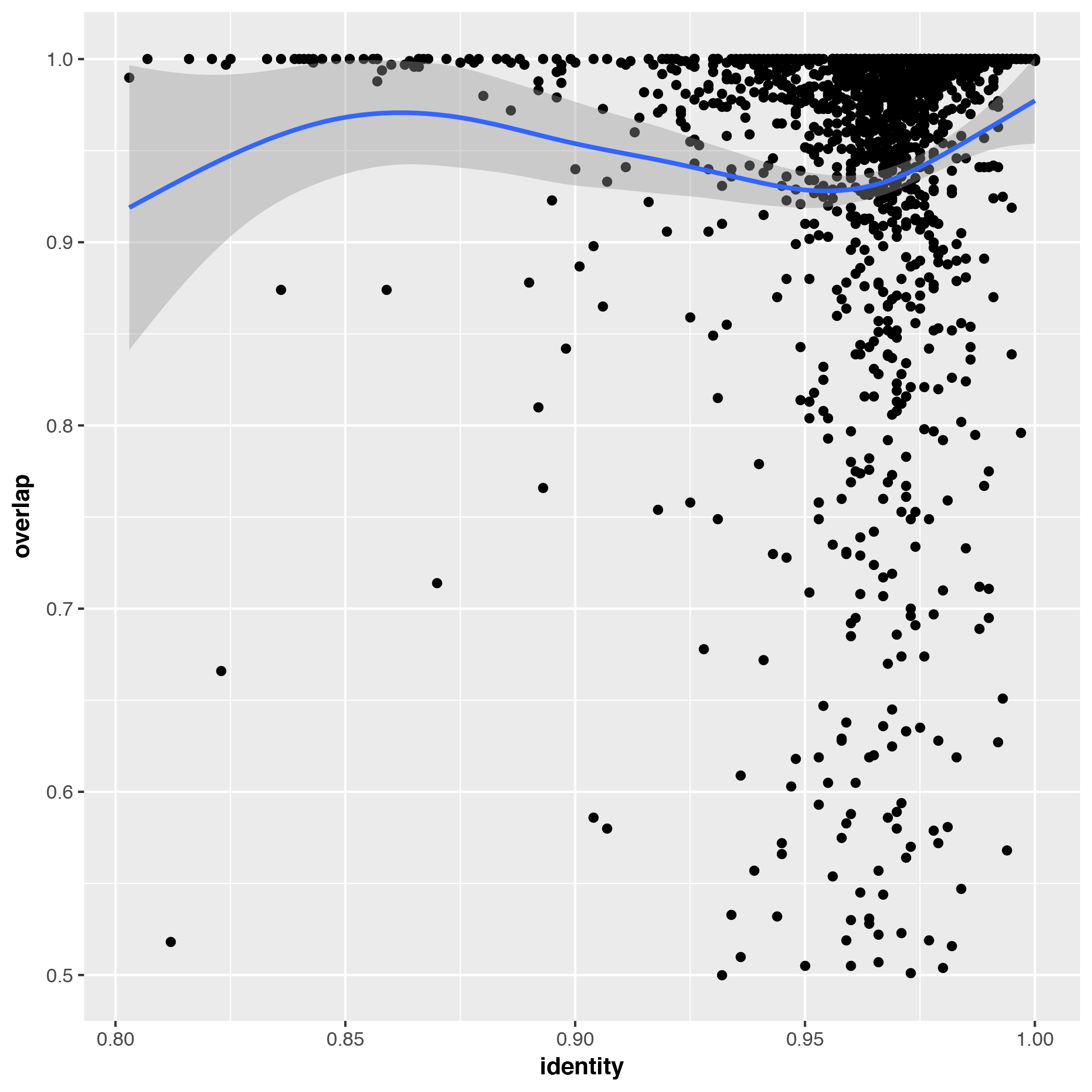
**

**Figure S5. The distribution of the 1,472 redundant contigs identified by LAST.**

**
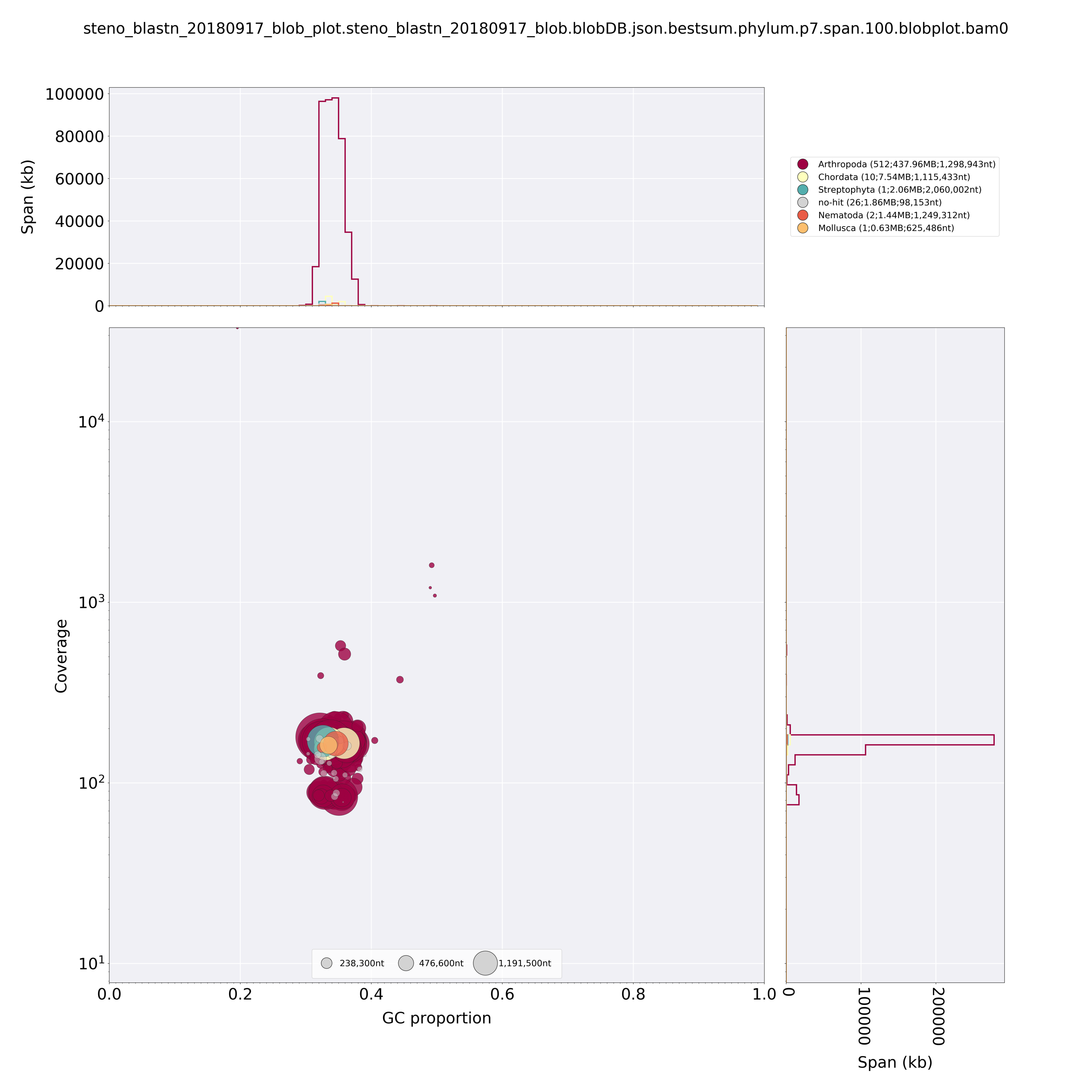
**

**Figure S6. Taxon-annotated GC-coverage (TAGC) plots for the final genome assembly of *Stenopsyche tienmushanensis*.** Each circle represents one contig in the assembly, with different colors based on the best match to the corresponding taxonomic annotation. The upper- and right- panels show the distribution of the total span (kb) of contigs for a given GC proportion or coverage.

**
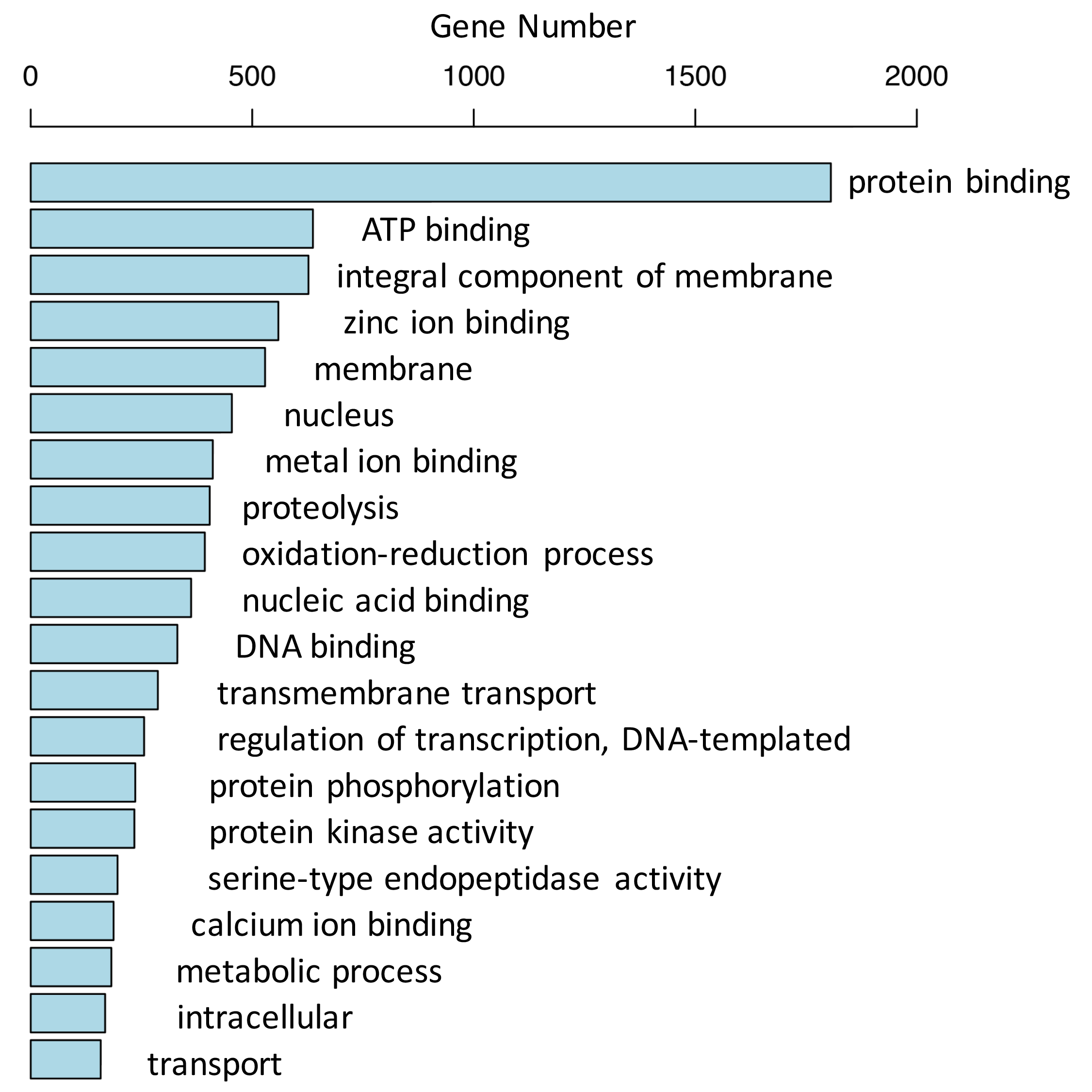
**

**Figure S7. Top 20 terms in the GO pathway analysis**

**
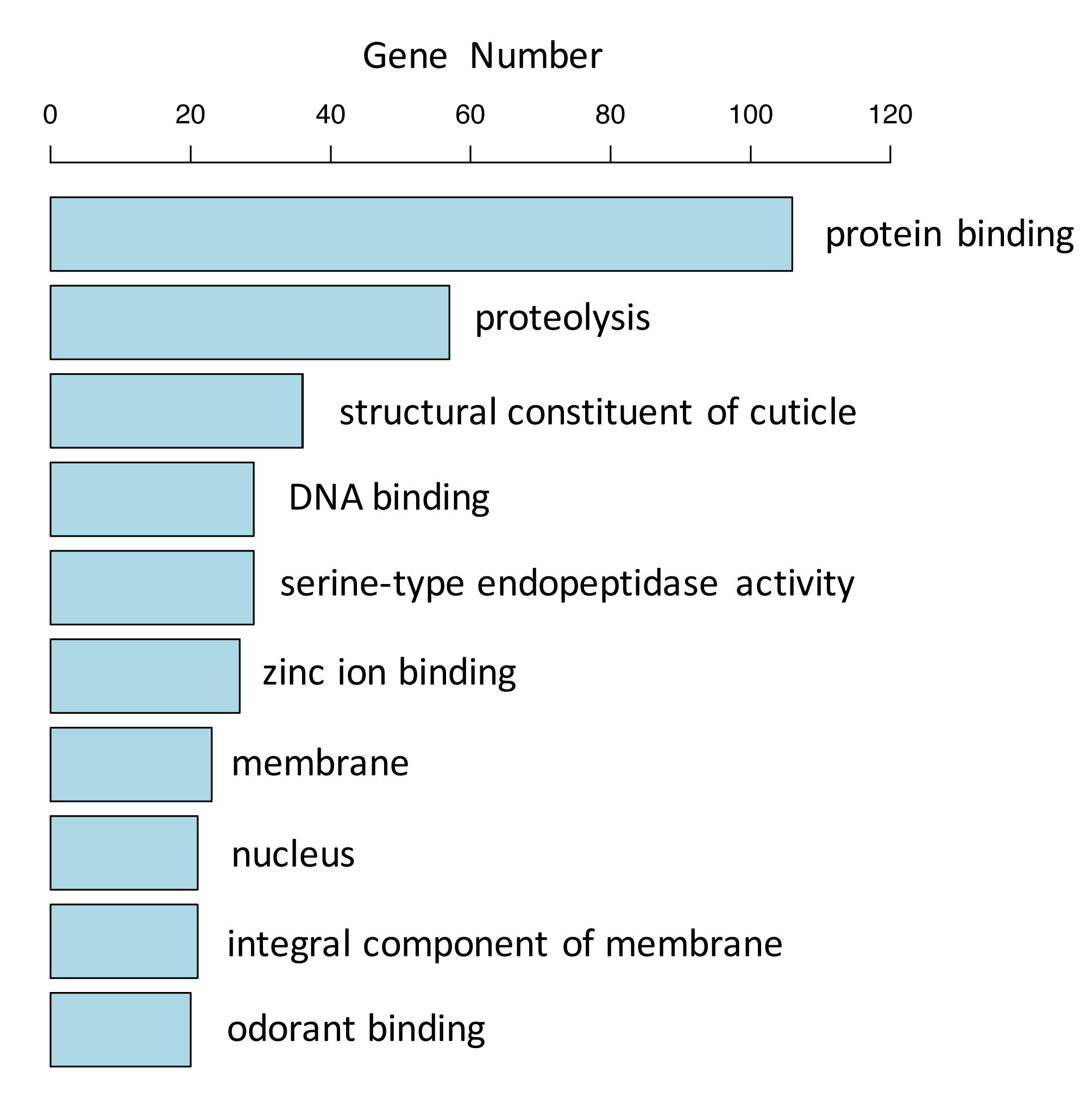
**

**Figure S8. Top 10 terms in the GO pathway analysis of the species-specific paralog genes**

**Supplementary Tables**

**Table S1 Sequencing data counts**

| Sample | Source | Library type | Platform | Read length | Insert size (bp) | Total base (bp) | Number of reads |
| --- | --- | --- | --- | --- | --- | --- | --- |
| Stie1 | DNA | short insert | Illumina  HiSeq X Ten | 150 bp paired-end | 400 | 40,679,119,800 | 271,194,132 |
| Stie2 | DNA | short insert | Illumina  HiSeq X Ten | 150 bp paired-end | 400 | 39,775,849,200 | 265,172,328 |
| Stie1+Stie2 | DNA | long read | PacBio Sequel | 7.6 kb (mean) | 20 k | 78,720,333,957 | 10,321,897 |
| Stie3 | RNA | short insert | Illumina  HiSeq X Ten | 150 bp paired-end | 180 | 9,723,314,700 | 64,822,098 |
| Stie3 | RNA | long read | PacBio Sequel | 1.1 kb (mean) | 0.5-6 k | 10,305,012,637 | 6,578,615 |

The Read length and Number of reads of PacBio sequencing results are based on the statistics of subreads.

**Table S2 Statistics of the initial and final genome assemblies**

| Stat Type | Initial |  | Final |  |
| --- | --- | --- | --- | --- |
|  | Contig  length | Contig number | Contig  length | Contig number |
| N50 | 1,160,110 | 134 | 1,296,863 | 110 |
| N60 | 933,486 | 183 | 1,109,837 | 147 |
| N70 | 674,154 | 248 | 884,073 | 192 |
| N80 | 411,166 | 344 | 657,931 | 252 |
| N90 | 164,125 | 531 | 415,262 | 337 |
| Longest | 4,744,952 | 1 | 4,765,805 | 1 |
| Total | 510,726,241 | 2,054 | 451,494,475 | 552 |
| Length ≥ 1kb | 510,710,601 | 2005 | 451,494,475 | 552 |
| Length ≥ 2kb | 1,160,110 | 134 | 451,493,386 | 551 |
| Length ≥ 5kb | 933,486 | 183 | 451,489,603 | 550 |

**Table S3 Statistics of Taxon-annotated GC-coverage (TAGC) analysis (see Table S3.xlsx).** “Iso-Seq” represents contigs mapped by full-length transcripts sequenced by PacBio Iso-Seq. “BUSCO” represents contigs containing insect homologous genes categorized in BUSCO.

**Table S4 Numbers of different types of Simple Sequence Repeat (SSR)**

| Type | Unit size (repeat number) | Number |
| --- | --- | --- |
| p1 | 1( ≥ 10) | 68,598 |
| p2 | 2( ≥ 6) | 18,327 |
| p3 | 3( ≥ 5) | 3,957 |
| p4 | 4( ≥ 5) | 465 |
| p5 | 5( ≥ 5) | 204 |
| p6 | 6( ≥ 5) | 13 |

Type: p1-6: repeats consisting of 1 to 6 nucleotides, respectively. Unit size: the number of nucleotides in a repetitive unit of SSR.

**Table S5 Annotated repeat sequences from different methods**

| Methods | Repeat Size (bp) | % of genome |
| --- | --- | --- |
| LTR_finder | 1,749,004 | 0.39 |
| TRF | 3,579,704 | 0.79 |
| RepeatMasker + Repbase library | 46,773,887 | 10.36 |
| RepeatMasker + RepeatModeler *de novo* library | 156,642,282 | 34.69 |
| RepeatProteinMasker | 30,030,332 | 6.65 |
| Total | 165,974,935 | 36.76 |

“Total” represents the sum of repeat sequences identified by different methods.

**Table S6 Statistics of gene prediction based on three methods**

| Method | | Software | Total number of genes | Average gene length (bp) | Average CDS length (bp) | Average exon number per gene | Average exon length (bp) | Average intron number per gene | Average intron length (bp) |
| --- | --- | --- | --- | --- | --- | --- | --- | --- | --- |
| *De novo* | AUGUSTUS | | 20,480 | 8,026.13 | 1,345.17 | 5.31 | 253.17 | 4.31 | 1,548.93 |
| Homology | GeneWise | | 13,626 | 5,448.77 | 1,065.62 | 4.20 | 253.99 | 3.20 | 1,371.82 |
| cDNA | PASA | | 9,498 | 13,777.65 | 1,490.54 | 6.87 | 216.94 | 5.87 | 2,092.92 |
| Final set | EVM | | 14,672 | 11,209.47 | 1,510.83 | 6.43 | 234.90 | 5.43 | 1,785.52 |

**Table S7 Comparison of gene annotations with representative lepidopterans**

| Species | Total number of genes | Average gene length (bp) | Average CDS length (bp) | Average exon number per gene | Average exon length (bp) | Average intron length (bp) |
| --- | --- | --- | --- | --- | --- | --- |
| *Stenopsyche tienmushanensis* | 14,672 | 11,209.47 | 1,510.83 | 6.43 | 234.90 | 1,785.52 |
| *Bombyx mori* | 13,663 | 14,654.48 | 1,429.25 | 6.79 | 210.52 | 2,284.46 |
| *Danaus plexippus* | 15,130 | 6,002.37 | 1,382.82 | 6.71 | 205.97 | 808.51 |
| *Heliconius melpomene* | 19,908 | 5,254.51 | 1,217.58 | 5.49 | 221.88 | 899.61 |
| *Plutella xylostella* | 18,106 | 9,657.24 | 1,406.53 | 7.07 | 198.87 | 1,358.68 |

The source of genome assemblies: *B. mori* : ASM15162 v.1 [1], *D. plexippus* v.3 [2], *H. melpomene* Hmel2.5 [3, 4]，*P. xylostella* DBM_FJ_V1.1 [5].

**Table S8 Genome data sources of the 11 arthropod species used in evolutionary analysis**

| Species | Data source |
| --- | --- |
| *Apis mellifera* | ftp://ftp.ncbi.nlm.nih.gov/genomes/all/GCF/000/002/195/GCF_000002195.4_Amel_4.5 |
| *Acyrthosiphon pisum* | ftp://ftp.ncbi.nlm.nih.gov/genomes/all/GCF/000/142/985/GCF_000142985.2_Acyr_2.0 |
| *Pediculus humanus* | ftp://ftp.ncbi.nlm.nih.gov/genomes/all/GCF/000/006/295/GCF_000006295.1_JCVI_LOUSE_1.0 |
| *Tribolium castaneum* | ftp://ftp.ncbi.nlm.nih.gov/genomes/all/GCF/000/002/335/GCF_000002335.3_Tcas5.2 |
| *Plutella xylostella* | ftp://ftp.ncbi.nlm.nih.gov/genomes/all/GCF/000/330/985/GCF_000330985.1_DBM_FJ_V1.1 |
| *Heliconius melpomene* | http://www.butterflygenome.org/sites/default/files/Hmel2.5_Release_2017-10-05.tar.gz |
| *Danaus plexippus* | [http://monarchbase.umassmed.edu/download/Dp_geneset_OGS2*](http://monarchbase.umassmed.edu/download/Dp_geneset_OGS2_*.fasta.gz) |
| *Bombyx mori* | <http://download.lepbase.org/v4/sequence/Bombyx_mori_ASM15162v1*> |
| *Clunio marinus* | ftp://ftp.ncbi.nlm.nih.gov/genomes/all/GCA/900/005/825/GCA_900005825.1_CLUMA_1.0/ |
| *Drosophila melanogaster* | ftp://ftp.flybase.net/genomes/Drosophila_melanogaster/dmel_r6.18_FB2017_05 |
| *Daphnia pulex* | ftp://ftp.ncbi.nlm.nih.gov/genomes/all/GCA/000/187/875/GCA_000187875.1_V1.0 |

References

1. Duan J, Li R, Cheng D, et al. SilkDB v2. 0: a platform for silkworm (*Bombyx mori*) genome biology. Nucleic Acids Res 2009;**38**(suppl_1):D453-6.

2. Zhan S, Merlin C, Boore JL, et al. The monarch butterfly genome yields insights into long-distance migration. Cell 2011;**147**(5):1171-85.

3. Dasmahapatra KK, Walters JR, Briscoe AD, et al. Butterfly genome reveals promiscuous exchange of mimicry adaptations among species. Nature 2012;**487**(7405):94-8.

4. Davey JW, Chouteau M, Barker SL, et al. Major improvements to the *Heliconius melpomene* genome assembly used to confirm 10 chromosome fusion events in 6 million years of butterfly evolution. G3 2016;**6**(3):695-708.

5. You M, Yue Z, He W, et al. A heterozygous moth genome provides insights into herbivory and detoxification. Nat Genet 2013;**45**(2):220-5.
