# Supplementary material for: The genome of an underwater architect, the caddisfly Stenopsyche tienmushanensis Hwang (Insecta: Trichoptera)
Source: Gigascience. 2018 Nov 23;7(12):giy143. doi: 10.1093/gigascience/giy143 (PMC6302954; doi:10.1093/gigascience/giy143)
Supplement: response_to_reviewer_comments_original_submission.pdf [file giy143_response_to_reviewer_comments_original_submission.pdf]

Author's Response To Reviewer Comments

Close

Please check the file "Response_to_reviewers_20180925.docx" uploaded as "Personal cover"

Close
