# Supplementary material for: The genome of an underwater architect, the caddisfly Stenopsyche tienmushanensis Hwang (Insecta: Trichoptera)
Source: Gigascience. 2018 Nov 23;7(12):giy143. doi: 10.1093/gigascience/giy143 (PMC6302954; doi:10.1093/gigascience/giy143)
Supplement: reviewer_1_report_(original_submission) -- Scott Hotaling — 7/19/2018 Reviewed [file giy143_reviewer_1_report_(original_submission).pdf]

Reviewer Report

Title: **The genome of an underwater architect, the caddisfly Stenopsyche tienmushanensis Hwang (Insecta: Trichoptera)**

Version: **Original Submission** **Date:** 7/19/2018

Reviewer name **Scott Hotaling**

Reviewer Comments to Author:

Luo et al. present a high-quality genome assembly for the caddisfly Stenopsyche tienmushanensis, a first for the entire insect order Trichoptera. This is an exciting, important advance that greatly improves existing genomic resources for the group. In their assembly, the authors integrate short-read and long-read data for both DNA and RNA to improve the contiguity of their assembly and their gene annotations. In addition to a high-quality genome, the authors incorporate analyses that assess possible gene family expansions and contractions in caddisflies with a specific focus on cytochrome P450 and olfactory binding proteins. They also present the first full sequence of the H-fibroin gene which is the key factor underlying the unique, aquatic silk produced by caddisflies in their case-making activities.

The generated data and subsequent analyses both appear robust for the task at hand. My most overarching critique, and the general theme in my comments below, is one of clarity. Both the writing style and content of the paper regarding individual analyses and results, need to be substantially improved in the revision. My general comments focus on sections that are particularly deficient or problematic in this regard and I follow with more specific comments on a line-by-line basis.

Simply put, my review can be summarized as follows: This is an important, timely paper that needs additional polish.

General comments:
Writing style/clarity:
I won't belabor this point any more than I already have but the paper is riddled with missing words and confusing grammar which makes it difficult to read and, more importantly, difficult to understand in several key areas. A few minor examples include: Line 25 -- there's no need for "the" after "adaptations to", Line 26 -- pluralizing "architectures" is odd in this context, Line 95 - "prestine" should be "pristine", etc. Active versus passive voice also varies and being consistent in this regard should be considered while revising. Personally, I prefer active voice. More major examples include a general lack of satisfactory detail in important sections like the "Genome assembly methods."

Genome assembly methods:
Since the manuscript is centered upon this part of the process, that is, how the genome assembly was produced, it's essential that this section be improved. The authors should revise with a focus on briefly explaining each step, why it was performed, and how it fits into the broader picture. For instance, reads from both Stie1 and Stie2 were used for both PacBio and Illumina sequencing, correct? If so, why specify Stie1 and Stie2 on Line 137 if there are no other options? What does it mean to "rectify" a genome assembly with Arrow? Why map your short reads back to your PacBio only assembly? Just to be sure they map at a high rate? You discuss "another revision using Pilon"? What does this mean? Where is the first revision and what was its purpose? Beginning in the 2nd paragraph of this section (Line 144), it's unclear to me what the "corrected, intermediate assembly" is. For the transcriptome sequencing, there was no assembly, correct? You took complete PacBio reads and consider them to be your transcripts. If this is true, what role did the Illumina short-reads from RNA sequencing data play in the process?

Gene prediction methods:

Similar to my comments about the genome assembly methods. This paragraph/section needs to be more clear/thorough. For instance, are there no parameters to specify in Augustus? Did you use defaults? Why 1,000 transcripts ab initio training? You need to report the number of transcripts recovered (Line 130), not just the total length of them. Particularly the number of "full length" transcripts since that appears to be what you used in lieu of any transcript assembly method. I would also like to see a min./max. of the range of transcript sizes. Also, how is training Augustus with transcripts not RNA-seq-assisted? You begin by mentioning three methods of gene prediction, then never mention the "RNA-seq-assisted" again. The reference for EVM should be moved to the end of the sentence as it also includes PASA (which I also think should be spelled out in parentheses, "Program to Assemble Spliced Alignments"). But, aside from specific comments, please revise this section with a focus on clarity. What exactly did you do and why?

Gene orthology analysis and phylogenetic tree:
"The region with low quality was filtered with Gblocks" (Lines 247-248). Please clarify this statement. This is minor but I would re-phrase wording like "is closely related to OBP4a" to "is most closely-related to OBP4a" because you aren't giving any quantified value of how closely related they are, just that the D. melanogaster gene family is the most similar one in the database but for all your reader knows, it may also be one of the only options in the database making "close" kind of meaningless.

Concluding remarks:
I would like to see a couple more references in this section. For example, "among the strongest supported ordinal relationships" (Line 322). Also, I think the closing sentence could be stronger. Is the publication of a caddisfly genome only suited to understanding how insects adapted to terrestrial habitats? I would expand this to include aquatic vs. terrestrial lifestyles, the evolution of case-making (the coolest thing about caddisflies in my opinion!), and the evolution of a robust, aquatic silk. The concluding remarks should be summarizing the entire study, not only the paper's most recent line of thinking.

Supporting data:
The final genome assembly, gene models, and transcripts should be submitted to GenBank and linked with the existing BioProject/SRA archive. Accessioning it on the GigaScience database (GigaDB), while useful, is not sufficient in my opinion.

Figure 3 - I think you can remove the statement about single-copy orthologs from the legend. Or, if you want to include it, please make its definition less circular. The definition for species-specific paralogs also appears incorrect - or do you mean these are in fact genes with multiple copies in only one species?

Figures 4 & 5 - Please make the colorations of species represented in both figures match one another (e.g., S. tienmushanesis & T. castaneum should have the same coloration in both figures).

Figure 6 - This figure and its legend needs to be clarified. A/B/C for the breakouts would help. And, is it correct that you're saying the gene is 21,089 bp long with only one ~80 bp intron? It is also unclear what the various measurements (3,543 and 1,654 bp) refer to exactly. Also, why is S. marmorata abbreviated as "S.mar5" and not just "S.mar"?

Table 1 - Please add the accession information for the L. lunatus and G. pellucidus genomes. You can make this a new row in the table. Please also report the contig N50 in kb and add additional detail to the BUSCO results for your reader. You do not need to include the number of genes searched as that is included in the text. I would only report complete (with percentage in parentheses), fragmented (and %), and missing (and %). These can all be their own rows. As is, your audience doesn't know what C/S/D/etc. mean. You should also consider adding the Sericostoma sp. assembly on GenBank (https://www.ncbi.nlm.nih.gov/assembly/GCA_003003475.1) to the table (with corresponding BUSCO results and update any references in the text - if there are any - to four caddisfly genomes, not three.

Table 2 - Please provide a more informative legend specifying, briefly, what categories like "Other" and "Unknown" mean. Also, I would prefer the totals for DNA to Unknown to be in order from highest to lowest

percentage of the genome. And, a line separating "Total" from the others would be useful since it is a summation and not a category per se like the rest.

Table S1 - Was the short-read RNA data from Stie3 used in any way in the manuscript? If so, this needs to be described in the text (though it's possible I have overlooked it).

Table S3 - Please include a more informative legend. "Number" for Total examined sequences = 557 - this is the number of contigs, correct? Is this useful? It just says you examined everything in the "Final" assembly. And are the other values numbers or sizes?

Table S5 - Again, this needs more information in the legend and "Total" should be broken out from the other categories. Also, what does "Total" mean exactly? It's unclear from the listed categories.

Specific comments:
Lines 30-31 - I would prefer these values be stated as the number of reads or removed entirely and discussed elsewhere. In the Abstract, I think it's alright to just say that you leveraged both short- and long-reads for the assembly.

Lines 48-49 - Break this into two sentences. Right now, it implies that if you include Antarctica there are more diverse insect orders. Of course you don't mean that!

Line 51 - The Misof et al. (2014) paper should be cited after the divergence timing estimate.

Line 86 - I would prefer "aquatic" over "watery."

Line 92-93 - I don't think the historical note about Chinese taxonomy is relevant. It's distribution in China is relevant though.

Line 105 - I would like a bit more detail about the DNA extraction procedure given that high-molecular weight DNA is crucial to long-read sequencing. I know you gave the Fu et al. reference, but a bit more detail here without having to track down another reference would be useful for the reader.

Line 111 - Capitalize "ten" in the name of the sequencer.

Line 112 - What does a "genome survey was carried out" mean? What was the point of using jellyfish on your data?

Lines 115-116 - How was a heterozygosity rate inferred from Arabidopsis? Reference for this method?

Line 121 - This is redundant. You already stated that Stie3 was used for RNA sequencing.

Line 127 - What is the "ICE" algorithm? Reference?

Line 130 - I would prefer to know how many transcripts this refers to. You can put the total basepairs in parentheses if you'd like to keep it included.

Line 153 - As currently written, this implies there are three existing caddisfly genomes, there aren't. There are two (actually three, Sericostoma sp. - https://www.ncbi.nlm.nih.gov/assembly/GCA_003003475.1) and your new one. I would re-phrase to something like "a comparison between the S. tienmushanensis and *three publicly available caddisfly genomes (names and references) is provided in Table 1."

Line 185 - You spell out non-coding RNAs but nothing else in this paragraph. I would revise to spell them all out before using abbreviations.

Line 214 - No need for "The source of genome assemblies:" - you can simply list the species and their genome accession information.

Line 231 - Add generation after phylogenetic tree? This subheading needs at least one more word.

Line 236 - Strange wording in the parentheses. Just say something like "See Table S8 for additional details".

Lines 269-270 - You stated this was species-specific at the beginning of the sentence, no need to say it again after the Fig. S4 reference.

Line 273 - "PBP_GOBP" - clarify what exactly this is and consider re-structuring this sentence.

Lines 280-283 - I don't mind speculation in a genome note but I'd like to see some references for the first sentence of this statement, followed by some evidence that caddisflies are particularly adept at finding mates versus other short-lived aquatic insects. Or, perhaps, you could further develop the argument that this may be generally true for aquatic insects. But don't mayflies have very short adult lifespans? Often shorter than caddisflies? If your hypothesis is correct, why don't they have greatly expanded OBP gene families?

Line 312 - I think you can do a better job selling the relevance of the study here. You present the first high-quality caddisfly genome, period. I would ditch the "welcome addition" part in the next sentence, it's a bit casual and implies you speak for the caddisfly community. (It is welcome though from this researcher's perspective!)

Can the authors provide any thoughts - speculation is quite alright - about why caddisfly genomes appear to be pretty variable in size? We now have four estimates and they range from S. tienmushanensis at ~0.45 Gb to L. lunatus at ~1.3 Gb, with two others in the middle of the range. How does this compare to, say, genome variation in Lepidoptera?

Level of Interest

Please indicate how interesting you found the manuscript: Choose an item.

Quality of Written English

Please indicate the quality of language in the manuscript: Choose an item.

Declaration of Competing Interests

Please complete a declaration of competing interests, considering the following questions:

- Have you in the past five years received reimbursements, fees, funding, or salary from an organisation that may in any way gain or lose financially from the publication of this manuscript, either now or in the future?
- Do you hold any stocks or shares in an organisation that may in any way gain or lose financially from the publication of this manuscript, either now or in the future?
- Do you hold or are you currently applying for any patents relating to the content of the manuscript?

- Have you received reimbursements, fees, funding, or salary from an organization that holds or has applied for patents relating to the content of the manuscript?
- Do you have any other financial competing interests?
- Do you have any non-financial competing interests in relation to this paper?

If you can answer no to all of the above, write 'I declare that I have no competing interests' below. If your reply is yes to any, please give details below.

I declare that I have no competing interests

I agree to the open peer review policy of the journal. I understand that my name will be included on my report to the authors and, if the manuscript is accepted for publication, my named report including any attachments I upload will be posted on the website along with the authors' responses. I agree for my report to be made available under an Open Access Creative Commons CC-BY license (http://creativecommons.org/licenses/by/4.0/). I understand that any comments which I do not wish to be included in my named report can be included as confidential comments to the editors, which will not be published.

I agree to the open peer review policy of the journal

To further support our reviewers, we have joined with Publons, where you can gain additional credit to further highlight your hard work (see: https://publons.com/journal/530/gigascience). On publication of this paper, your review will be automatically added to Publons, you can then choose whether or not to claim your Publons credit. I understand this statement. Yes
