# Supplementary material for: The genome of an underwater architect, the caddisfly Stenopsyche tienmushanensis Hwang (Insecta: Trichoptera)
Source: Gigascience. 2018 Nov 23;7(12):giy143. doi: 10.1093/gigascience/giy143 (PMC6302954; doi:10.1093/gigascience/giy143)
Supplement: reviewer_1_report_revsion_1 -- Scott Hotaling — 10/28/2018 Reviewed [file giy143_reviewer_1_report_revsion_1.pdf]

Reviewer Report

Title: The genome of an underwater architect, the caddisfly Stenopsyche tienmushanensis Hwang (Insecta: Trichoptera)

Version: Revision 1 Date: 10/28/2018

Reviewer name Scott Hotaling

Reviewer Comments to Author:

The revised manuscript is a substantial improvement over the previous version. However, my two overarching critiques of the previous version - (1) that the Methods were generally lacking in clarity (and specifics) and (2) that the writing would be improved by close copy-editing still stand. If the authors are worried about bogging down the manuscript with program details, they should consider a supplementary document that includes all of the programs used with the appropriate flags/usage details listed. A GitHub repository with the code to reproduce their analyses would be an even better solution.

That said, the authors have done a nice job improving the manuscript and I am confident further consideration of both clarity and reproducibility would improve it even more. Below, I have noted some specific, largely minor, critiques, many which reflect from my general criticisms above. My view that this resource is important to the field and will serve as a valuable resource for future studies of caddisfly evolution remains unchanged.

Specific comments:
Line 35 - Re-word this sentence. I would split apart the repeat percentage and number of genes into separate sentences as they don't make grammatical sense as is.

Line 36 - The genome reveals this. Not the "genome sequences" or, perhaps they do, but that's an odd way to put it!

Lines 86-87 - Was the complete genome the key or the long-read sequences? I would argue the latter.

Line 110 - How were the "guts" removed? I assume dissection but this should be stated.

Line 113 - The RNA extraction methods are not sufficient.

Line 121 - How many SMRT cells were sequenced? I don't follow the "movie length" definition of sequencing effort. Or perhaps both the number of SMRT cells and the movie length should be reported if they are relevant to different aspects of the sequencing process.

Line 127 - "full length transcriptomes"? I assume this should be transcripts?

Lines 130-133 - Is this all of the filtering that was performed? How did you determine those 5 bases needed to be trimmed? For PacBio, are "subreads" the same as reads? Why call them "subreads"? After some googling, it appears there is a bit of difference between reads/subreads. I think this should be stated briefly in the paper, your audience may not be aware of it.

Line 173 - "Transcriptomes" should be transcripts?

Line 175 - "After filtering the low-quality reads" - how was this done?

Lines 173-188 - There are many instances of "Program X was run..." then "We ran Program Y..." with very

few details about these analyses. The authors need to provide more detail for how sequencing errors were corrected (Lines 181-182), how polishing was performed (Line 181), etc. And, to be clear, this comment applies beyond these ~15 lines to much of the paper. Running a program without specific details of how it was performed is a common theme here and perhaps the most significant weakness of the study in its current form.

Line 224 - You can merely reference Table S2. No need for "Statistics of the genome assembly in". There are a couple of other places where the writing can be tightened in this way as well.

Line 240 and the rest of the paragraph - Again, lots of programs being used with few details. These would be very hard, if not impossible, analyses to replicate.

Line 266 and the rest of the section - Same comment as above. GeneWise, ASA, BLAT, Transdecoder, EvidenceModeler - all ran with default parameters? Any changes?

Line 393 - "Mm" means "molecular mass", right? I would spell this out and then give the acronym (if needed).

Level of Interest

Please indicate how interesting you found the manuscript: Choose an item.

Quality of Written English

Please indicate the quality of language in the manuscript: Choose an item.

Declaration of Competing Interests

Please complete a declaration of competing interests, considering the following questions:

- Have you in the past five years received reimbursements, fees, funding, or salary from an organisation that may in any way gain or lose financially from the publication of this manuscript, either now or in the future?
- Do you hold any stocks or shares in an organisation that may in any way gain or lose financially from the publication of this manuscript, either now or in the future?
- Do you hold or are you currently applying for any patents relating to the content of the manuscript?
- Have you received reimbursements, fees, funding, or salary from an organization that holds or has applied for patents relating to the content of the manuscript?
- Do you have any other financial competing interests?
- Do you have any non-financial competing interests in relation to this paper?

If you can answer no to all of the above, write 'I declare that I have no competing interests' below. If your reply is yes to any, please give details below.

I declare that I have no competing interests

I agree to the open peer review policy of the journal. I understand that my name will be included on my report to the authors and, if the manuscript is accepted for publication, my named report including any attachments I upload will be posted on the website along with the authors' responses. I agree for my report to be made available under an Open Access Creative Commons CC-BY license (http://creativecommons.org/licenses/by/4.0/). I understand that any comments which I do not wish to be included in my named report can be included as confidential comments to the editors, which will not be published.

I agree to the open peer review policy of the journal

To further support our reviewers, we have joined with Publons, where you can gain additional credit to further highlight your hard work (see: https://publons.com/journal/530/gigascience). On publication of this paper, your review will be automatically added to Publons, you can then choose whether or not to claim your Publons credit. I understand this statement. Yes
